# A modular and divergent approach to spirocyclic pyrrolidines†

**DOI:** 10.1039/d0sc03676e

**Published:** 2020-08-07

**Authors:** Benjamin D. A. Shennan, Peter W. Smith, Yusuke Ogura, Darren J. Dixon

**Affiliations:** Department of Chemistry, Chemistry Research Laboratory, University of Oxford 12 Mansfield Road Oxford UK darren.dixon@chem.ox.ac.uk

## Abstract

An efficient three-step sequence to afford a valuable class of spirocyclic pyrrolidines is reported. A reductive cleavage/Horner–Wadsworth–Emmons cascade facilitates the spirocyclisation of a range of isoxazolines bearing a distal β-ketophosphonate. The spirocyclisation precursors are elaborated in a facile and modular fashion, *via* a [3 + 2]-cycloaddition followed by the condensation of a phosphonate ester, introducing multiple points of divergence. The synthetic utility of this protocol has been demonstrated in the synthesis of a broad family of 1-azaspiro[4,4]nonanes and in a concise formal synthesis of the natural product (±)-cephalotaxine.

## Abstract 2

A three-step, modular and divergent sequence accessing challenging spirocyclic pyrrolidines has been developed, featuring a novel reductive spirocyclization cascade.

## Graphical abstract

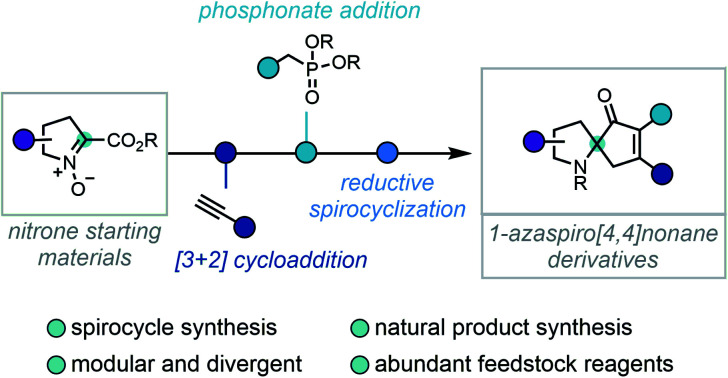

## Introduction

The pyrrolidine ring system is common to numerous pharmaceutical compounds and natural products of structural and biological importance. This is highlighted in its ranking as the 5^th^ most commonly occurring nitrogen-containing heterocycle in FDA-approved pharmaceuticals.^1^ Furthermore, there is a widespread abundance of the pyrrolidine scaffold within the architecturally complex polycyclic ring systems of natural products.^2^

**Scheme 1:**
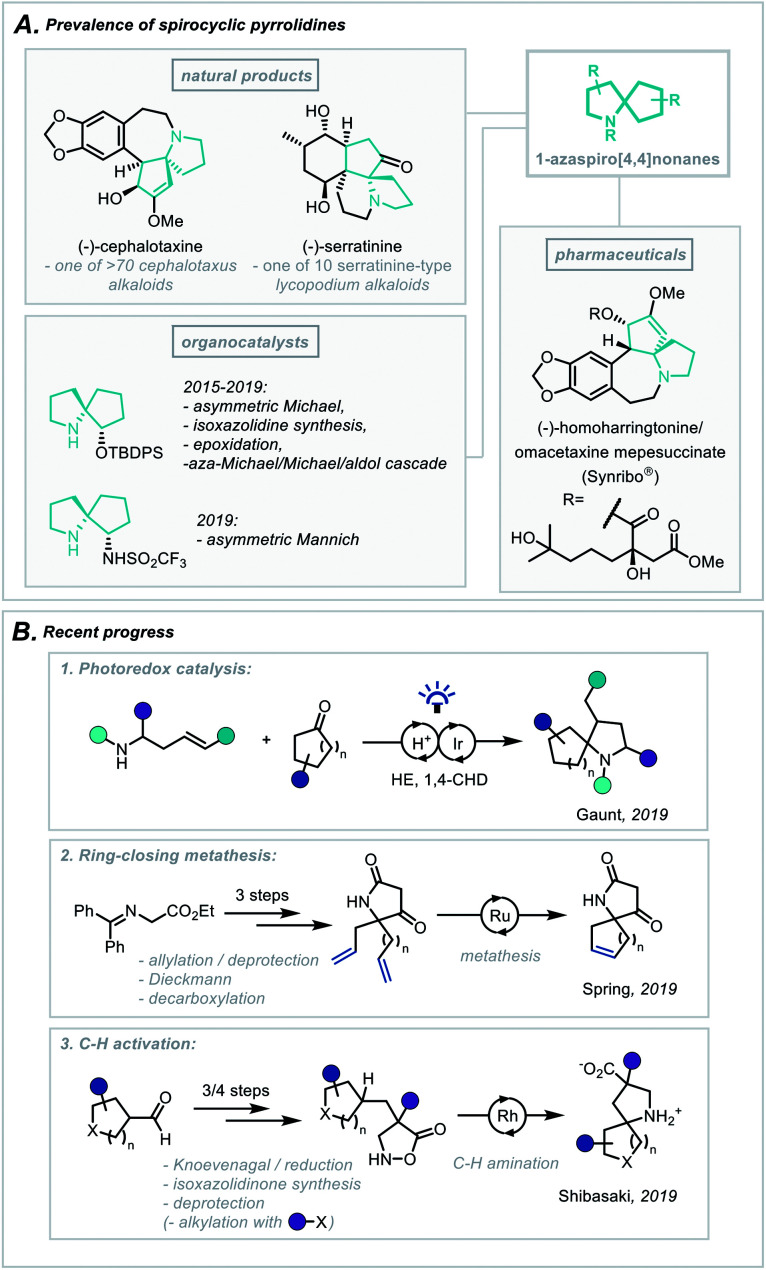
(A) Prevalence of spirocyclic pyrrolidines in natural products, pharmaceuticals and organocatalysis. (B) Recent progress in the synthesis of spirocyclic pyrrolidines.

In particular, the *Cephalotaxus* alkaloids (for example cephalotaxine, Scheme 1A) are characterised by a complex spirocyclic pyrrolidine scaffold, namely the 1-azaspiro[4,4]nonane framework, and as such have experienced a continuous wave of synthetic attention since their isolation.^3^ This has been catalysed by the development of homoharringtonine, one member of the family, into an FDA-approved medication (Synribo®) for treatment of chronic myeloid leukaemia in patients resistant to the more standard tyrosine kinase inhibitors.^4^

More recently, there has been a concerted effort in medicinal chemistry programmes to move towards fragment libraries comprising of more sp^3^-rich motifs due to their advantageous physicochemical attributes.^5^ Furthermore, spirocyclic scaffolds possess uniquely rigid structures, not only enabling greater predictability of conformation using *in silico* docking but also contributing to a less severe decrease in conformational freedom, and therefore entropy, upon protein binding.^6^

For these reasons, the development of efficient, broad-scope strategies for the construction of spirocyclic pyrrolidine systems, such as the 1-aza-spiro[4,4]nonane framework (Scheme 1A), has become a target of significant importance in synthetic method development.^7^ In this context, Gaunt and co-workers recently disclosed a general spirocyclisation method employing photoredox catalysis to generate α-amino radicals capable of 5-*exo*-trig/dig cyclisations (Scheme 1B(1)).^8^ From another access point, and focussing on the role these scaffolds play in fragment-based drug discovery, Spring and co-workers reported the use of a ring-closing metathesis strategy to afford a broad class of spirocyclic structures.^9^ The group went on to highlight qualitatively the molecules' favourable physicochemical properties as well as the diverse 3D chemical space these products occupy (Scheme 1B(2)). Additionally, an elegant report from Shibasaki and coworkers employed a Rh-catalysed intramolecular C–H amination of isoxazolidin-5-ones to construct a series of β-proline-derived spirocycles (Scheme 1B(3)).^10^

In a continuation of our programme for the development of new approaches towards structurally complex, sp^3^-rich nitrogen-containing architectures,^7*e*,11^ we recognised the importance of divergent and expedient access to this burgeoning chemical space. We reasoned that a modular reaction design, incorporating commercial or readily accessible building blocks, would provide multiple points of diversity, enabling an efficient and complementary route to the 1-azaspiro[4,4]nonane framework. Such a strategy could serve as the key complexity-building sequence in the synthesis of natural products,^12^ organocatalysts,^13^ or pharmaceutically relevant structures, and herein we wish to report our findings.

## Results and discussion

**Scheme 2:**
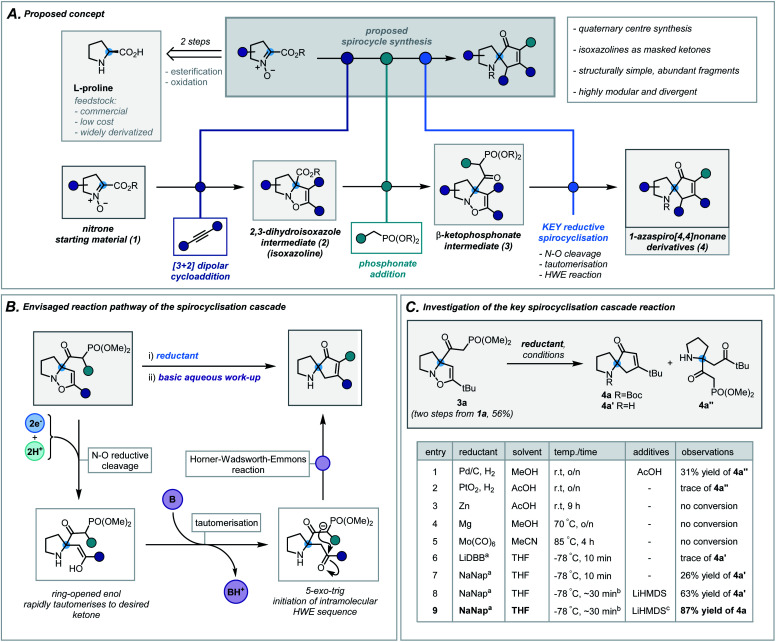
(A) Proposed concept. (B) Envisaged reaction pathway of the spirocyclisation cascade. (C) Investigation of the key spirocyclisation cascade reaction. ^a^ ∼1 M solution in THF, LiDBB = lithium 4,4′-di-*tert*-butylbiphenylide, NaNap = sodium naphthalenide; ^b^ reductant was titrated against reaction mixture to determine accurate endpoint, typically taking 30 minutes (see ESI for more details†); ^c^ Boc_2_O in THF was added following basic work up.

We envisaged exploiting an intramolecular Horner–Wadsworth–Emmons (HWE) reaction of a suitable β-ketophosphonate moiety with a pendant ketone as the key spirocyclisation reaction.^14^ The high exergonicity of such a transformation could allow the formation of hindered and highly-substituted cyclopentenones. In order to construct this privileged HWE precursor, we predicted the reductive ring cleavage of a 2,3-dihydroisoxazole moiety (referred to henceforth as isoxazolines) would efficiently reveal the desired ketone (Scheme 2B). This would be attractive due to the ready accessibility of such isoxazolines (**2**) *via* [3 + 2] dipolar cycloadditions between proline-derived nitrones (**1**) and terminal alkynes – a well-documented and reliable method for the incorporation of the desired quaternary centre.^15^ Furthermore, the use of proline-derived feedstocks, as well as simple terminal alkynes, would serve as a low-cost and widely available framework on which to develop and apply our methodology. The requisite β-ketophosphonate functionality would arise from the condensation of a suitable phosphonate anion with the pendant ester in **2**. Notably, numerous phosphonate esters are commercially available or are otherwise rapidly accessed from Michaelis–Arbuzov reactions and alkylation of dimethyl methylphosphonate. By exploiting these simple and dependable transformations, one could afford a broad range of ketophosphonate precursors (**3**). However, the feasibility and generality of this cycloaddition/phosphonate condensation sequence was unknown and would need to be probed in order to assess fully the functional group tolerance of the subsequent spirocyclisation reaction. In addition, while related N–O cleavage reactions are well-documented,^16^ this ambitious reductive cleavage/HWE cascade on such a densely functionalised isoxazoline is, to the best of our knowledge, unreported. In order to orchestrate the desired cascade sequence successfully, a carefully chosen reductant and set of reaction conditions would need to be established.

Nitrone **1a**, derived from l-proline benzyl ester, was chosen as a model substrate and its cycloaddition with phenylacetylene was investigated. After a concise solvent screen (ESI, Table S1†), effective conditions were established to afford isoxazoline **2d** in 67% yield as a single regioisomer.^15*c*^ Subsequent condensation of dimethyl methylphosphonate with the ester proceeded in high yield (70%) using LiHMDS as a base – a notable result given the considerable steric repulsion arising from the α-quaternary centre (ESI, Table S2†). Despite this progress, anticipating that the conjugated system in aryl-substituted isoxazolines may impair the desired reductive cleavage, ketophosphonate **3a** (R = *t*Bu) was chosen as a *simpler* framework on which to investigate the spirocyclisation cascade (Scheme 2C). A brief scouting of precedented N–O reducing agents and electron transfer reagents resulted in no desired spirocyclisation product (Scheme 2C, entries 1–5).^17^ However, standard Pd/C hydrogenation conditions afforded the intermediate ring-opened ketone in 31% yield (Scheme 2C, entry 1). Inspired by reported reductions of other weak bonds,^18^ radical anions of aromatic compounds (*e.g.* naphthalene and 4,4′-di-*tert*-butylbiphenylide) were investigated (Scheme 2C, entries 6 and 7). Despite typically being employed in detosylation, dehalogenation and similar transformations, there are scattered reports of alkali metal naphthalenides effecting N–O cleavage reactions in related systems.^19^ Pleasingly, sodium naphthalenide (NaNap) in THF afforded the desired spirocycle **4a′** in 26% yield, following an alkaline work-up to trigger the HWE reaction (Scheme 2C, entry 7). Transient protection of the ketophosphonate moiety as its anion using one equivalent of LiHMDS and careful control of sodium naphthalenide equivalents (see ESI for details†) improved the yield to 63% (entry 8). Due to the inherent instability of the resulting secondary amines and their challenging purification, a di-*tert*-butyl dicarbonate (Boc_2_O) “quench” was successfully implemented at the end of the sequence allowing isolation of desired *N*-Boc-protected spirocycle **4a** in 87% (entry 9).

**Scheme 3:**
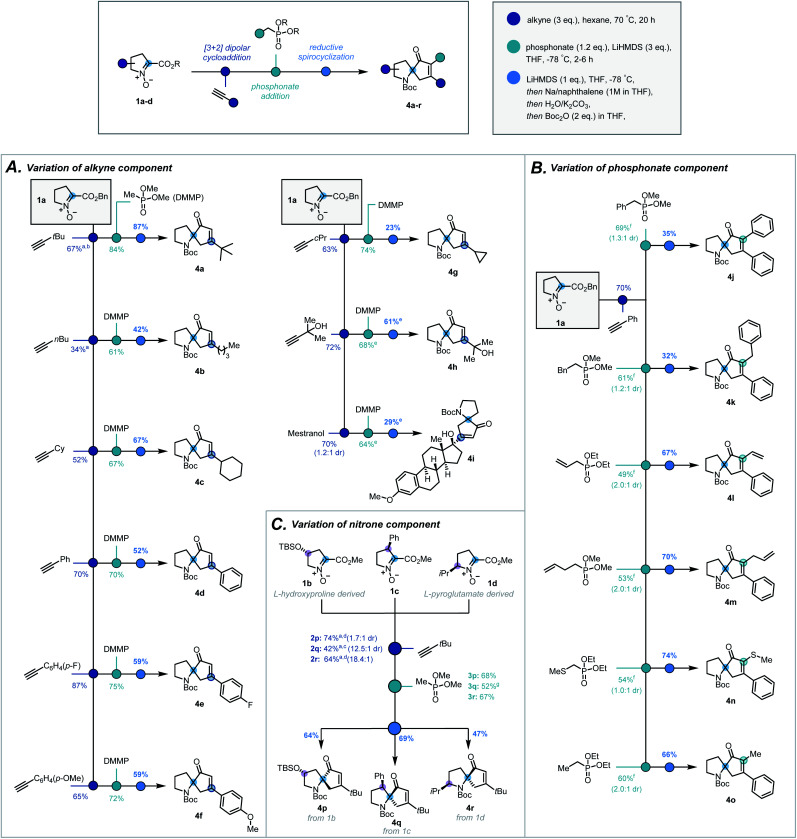
Scope of the three-step sequence with respect to (A) the alkyne, (B) the phosphonate ester and (C) the nitrone. ^a^ 6 eq. of alkyne, ^b^ reaction time = 3 days, ^c^ reaction time = 4 days and toluene as solvent, ^d^ reaction time = 2 days ^e^ extra eq. of base used, ^f^ modification to general procedure used (see ESI†), ^g^ extra equivalent of phosphonate and reaction temperature held at −78 °C for 7 h.

With optimal conditions established, the modularity and the scope of the sequence with respect to the terminal alkyne, the substitution on the phosphonate, and the substitution of the pyrrolidine ring, were investigated. Variation in the terminal alkyne enabled introduction of a range of functionalities and carbon frameworks at the 5-position^20^ of the isoxazoline (**2a–i**, Scheme 3A). Condensation of dimethyl methylphosphonate successfully afforded the corresponding ketophosphonates (**3a–i**) in moderate to good yields (61–84%). From cycloadduct **2d**, variation at the α-position of the ketophosphonate was introduced by the condensation of α-substituted phosphonates (Scheme 3B). Despite a more challenging addition, phenyl (**3j**), benzyl (**3k**), vinyl (**3l**), allyl (**3m**), thiomethyl (**3n**) and methyl (**3o**) groups were all introduced in synthetically viable yields.^21^

To investigate how substitution of the pyrrolidine backbone would affect the reaction sequence, nitrones suitably substituted at the 3-, 4- and 5-positions^20^ were synthesised *via* modified literature procedures (**1b–d**, ESI, Scheme S2–S4†). Notably, Bull's directed C–H activation of l-proline was employed to arylate the 3-position of the pyrrolidine ring.^22^

Utilising *tert*-butylacetylene as the alkyne, these substituted nitrones were submitted to the cycloaddition which proceeded in a diastereoselective fashion (1.7 : 1–18.4 : 1 dr, Scheme 3C, **2p–r**). For 4- and 5-substituted bicycles, the standard procedure was followed to afford ketophosphonates **3p** and **3r** successfully. In contrast, given the more hindered ester moiety, the 3-substituted bicycle required longer reaction times and greater excess of phosphonate to improve the yield of ketophosphonate **3q**.

**Scheme 4:**
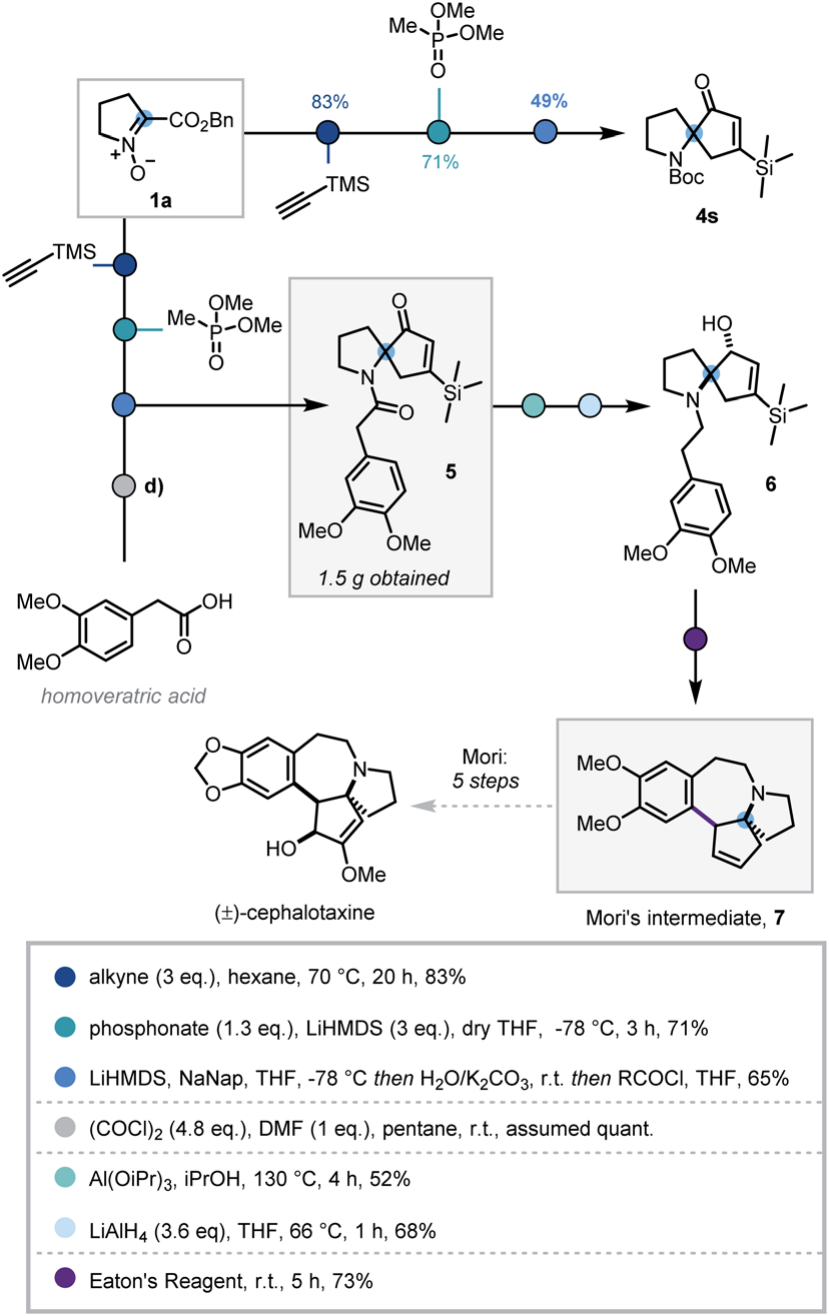
Synthesis of spirocycle **4s** and formal synthesis of (±)-cephalotaxine.

With a broad library of ketophosphonates in hand, the scope of the spirocyclisation was investigated. Following successful spirocyclisation of **4a**, substrates bearing alkyl side chains were initially submitted to the reaction conditions (Scheme 3A, **4a–c**, **4g**). Pleasingly the cyclised product was observed in all cases, with yields highest for bulkier alkyl side chains (*e.g. tert*-butyl, cyclohexyl). Aryl-substituted isoxazolines proceeded in appreciable, though somewhat reduced, yields (**4d–f**). This decrease in yield from alkyl substrates is rationalized by the electron-accepting properties of the isoxazoline moiety when conjugated with an aryl group, potentially leading to undesired side reactions. Unsuccessful results with strongly electron-withdrawing aryl substituents support this rationalisation.^23,24^ Further varying the isoxazoline substitution, a free tertiary alcohol (**3h**) was successfully implemented by using an additional equivalent of base prior to NaNap addition. This enabled construction of **4h** and structurally-complex spiroenone **4i** derived from mestranol, an FDA-approved hormone therapy.^25^

α-Substituted ketophosphonates delivered desired spiroenones **4j–4o** (Scheme 3B) with the highest yields obtained for substrates having relatively small α-side chains. This demonstrates the method's applicability for the synthesis of highly congested cyclopentenones. Furthermore, incorporation of allyl and vinyl groups could facilitate downstream functionalisation at the α-position of the cyclopentenone.

Encouragingly, substitution of the pyrrolidine backbone had no detrimental effect on the efficacy of the spirocyclisation, with spiroenones **4p–4r** all elaborated in synthetically practical yields (Scheme 3C). As such, the absolute stereochemical configuration of the quaternary carbon may be set to afford the spirocycles in single diastereomeric series – a particularly pertinent result given the application of 1-azaspiro[4,4]nonane derivatives in asymmetric organocatalytic manifolds.^13^

In order to demonstrate the synthetic utility of this three-step sequence, its application towards the synthesis of (±)-cephalotaxine was investigated. The *Cephalotaxus* alkaloids remain valuable synthetic targets and novel strategies to this class of molecules are desirable.^26^ It was envisaged that the use of trimethylsilylacetylene in the cycloaddition would introduce a substituent at the 5-position of the isoxazoline that could later be removed *via* protodesilylation, thereby intercepting synthetic intermediates reported by Mariano and Mori.^27^

Deploying the standard conditions, spiroenone **4s** was rapidly synthesized, thus confirming the facile incorporation of the desired silyl moiety (Scheme 4). Following this success, a modified acylative quench was investigated in which an acid chloride derived from homoveratric acid was employed to introduce the remaining core carbon atoms, *via* the pyrrolidine nitrogen. Pleasingly this modification was implemented with no issue, allowing key spirocycle **5** to be elaborated in 65% yield. This procedure could be carried out on a larger scale with only a modest depreciation in yield to 56%, affording 1.5 g of **5**.

Due to low reactivity inhibiting the desired protodesilylation,^28^ spirocycle **5** was reduced to the corresponding allylic alcohol, utilising Mariano's Meerwein–Ponndorf–Verley conditions, prior to amide reduction affording tertiary amine **6** in moderate yields.^27*b*^ Given the close structural similarity to Mori's Friedel–Crafts cyclisation precursor and the instability of vinyl silanes to protodesilylation in acidic media, **6** was submitted to Mori's Friedel–Crafts conditions. To our delight, **6** was indeed converted to the desilylated cyclisation product **7**, thus completing the formal synthesis of (±)-cephalotaxine.^27*a*^ However, this use of polyphosphoric acid provided inconsistent results and was operationally challenging. As such alternative acids and dehydrating reagents were investigated (see Table S4†) and Eaton's reagent (7.7 wt% P_2_O_5_ in MsOH)^29^ emerged as the most efficient, affording **7** in 73% after 5 h stirring at room temperature. This 7-step sequence from commercially available l-proline benzyl ester hydrochloride significantly expedites synthetic access to Mori's tetracyclic intermediate **7**.^27*b*,30^

## Conclusion

A concise and divergent approach to 1-azaspiro[4,4]nonane derivatives, which features a novel sodium naphthalenide-mediated reductive-HWE cascade reaction, has been developed. By variation of the alkyne, the phosphonate ester, and the pyrrolidine backbone, a large class of highly substituted and densely functionalised spirocyclic pyrrolidines was constructed, significantly broadening the synthetic access to this chemical space. We believe the modularity of this sequence lends itself well to applications in medicinal chemistry and natural product synthesis alike. To this end, the utility of the reaction pathway was demonstrated by its successful application in a formal synthesis of (±)-cephalotaxine, accessing Mori's tetracyclic intermediate in only 7 steps. Furthermore, this work demonstrates and opens up the underexploited paradigm of deploying isoxazolines as masked ketone equivalents in reductive cascade sequences.

## Conflicts of interest

There are no conflicts to declare.

## Supplementary Material

SC-011-D0SC03676E-s001
