# Supplementary material for: A modular and divergent approach to spirocyclic pyrrolidines
Source: Chem Sci. 2020 Aug 7;11(38):10354–60. doi: 10.1039/d0sc03676e (PMC8162384; doi:10.1039/d0sc03676e)
Supplement: SC-011-D0SC03676E-s001 [file SC-011-D0SC03676E-s001.pdf]

Electronic Supplementary Material (ESI) for Chemical Science.
This journal is © The Royal Society of Chemistry 2020

Supporting Information

for

A Modular and Divergent Approach to Spirocyclic Pyrrolidines

Benjamin D. A. Shennan, Peter W. Smith, Yusuke Ogura, and Darren J. Dixon*

Department of Chemistry, Chemistry Research Laboratory

University of Oxford, Mansfield Road, Oxford OX1 3TA (UK)

E-mail: darren.dixon@chem.ox.ac.uk

1. General Information ... 2
2. General Procedures for the Synthesis of 1-Azaspiro[4,4]non-8-en-9-ones ... 4
3. Optimization of Cycloaddition and Phosphonate Condensation ... 8
4. Nitrone Syntheses ... 10
5. Synthesis of Phosphonates ... 20
6. Synthesis and Characterisation of Cycloadducts ... 22
7. Synthesis and Characterisation of Ketophosphonates ... 29
8. Synthesis and Characterisation of Spirocycles ... 41
9. Formal Synthesis of (±)-Cephalotaxine ... 54
10. Challenging Substrates and Unsuccessful Intermediates ... 62
NMR Spectra for Novel Compounds ... 63
References ... 139

1. General Information

Bulk solutions were evaporated under reduced pressure using a Büchi rotary evaporator. All reagents were obtained from commercial suppliers and used without further purification. Where solvent dryness was important, CH_2Cl_2, Et_2O, THF or toluene were obtained from: dry solvent bottles with a septa (Aldrich or Acros) or an MBRAUN-SPS solvent purification system. All reactions were carried out under a nitrogen atmosphere.

Flash column chromatography was carried out using Merck Silicagel 60, particle size 40-63 μm. All reactions were followed by thin layer chromatography (TLC) when practical, using Merck aluminium-backed Silicagel 60 F_{254} fluorescent treated silica which was visualised under UV light (254 nm or 365 nm). In addition, TLC plates were stained with aq. basic potassium permanganate or ethanolic acidic vanillin.

^{1}H , **^{13}C**, **^{19}F** and **^{31}P NMR** spectra were recorded using Bruker AVIII HD 400 and Bruker AVII 500 spectrometers. Chemical shifts (δ) are quoted in parts per million (ppm) relative to tetramethylsilane (δ=0) and are referenced to the solvent residual peak. Coupling constants

(J) are quoted in Hertz (Hz), rounded to the nearest 0.1 Hz. The ^{1}H NMR spectra are reported as follows: ppm (multiplicity, coupling constants, number of protons). NMR assignments, where present, use numbering independent from IUPAC, using two dimensional (COSY, HSQC, HMBC) NMR spectroscopy to assist the assignment. Where distinction between major and minor diastereomers/rotamers has been possible the notation maj/min has been employed after each resonance. **Infrared (IR) spectra** were recorded on a Bruker Tensor 27 FT-IR spectrometer. Absorption maxima (ν_{max}) are reported in wavenumbers (cm^{-1}). Only selected absorption maxima are reported. **Low resolution mass spectra (LRMS)** were recorded on a Agilent single quadrupole with CTC-PAL mass spectrometer operating in positive ionisation mode. **High resolution mass spectra (HRMS)** were recorded on a Bruker Daltonics µTOF mass spectrometer. **Melting points (MP)** were recorded in degrees Celsius (°C), using a Leica Galen III hotstage microscope apparatus.

2. General Procedures for the Synthesis of 1-Azaspiro[4,4]non-8-en-9-ones

Oxidation Procedure A

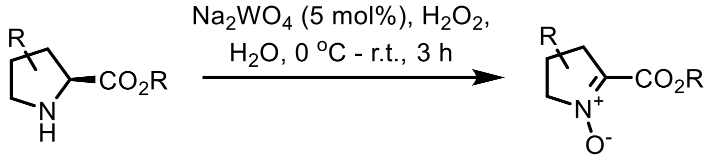

Following a modified literature procedure,[1] The secondary amine hydrochloride salt was dissolved in a minimum amount of H_2O, basified to > pH 10 with sat. aq. K_2CO_3 and then extracted with CH_2Cl_2. The combined organics were dried with Na_2SO_4, filtered and the solvent was removed under reduced pressure. To the resulting free amine was added H_2O (0.5 M), and the solution was cooled to 0 °C before adding Na_2WO_4 (5 mol%). H_2O_2 (30% aq., 2.2 eq.) was then added dropwise over 30 min and this was biphasic mixture was stirred under a N_2 atmosphere for 3 h, at which point it was cooled to 0 °C and excess H_2O_2 was quenched via addition of sat. aq. Na_2SO_3. The resulting aqueous mixture was extracted with CH_2Cl_2 and the combined organics were dried with Na_2SO_4, filtered and the solvent was removed under reduced pressure. The resulting crude oil was purified by FCC.

Oxidation Procedure B

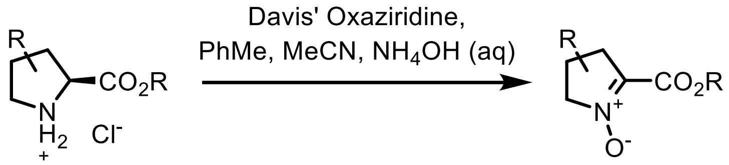

Following a literature procedure,[2] to a suspension of secondary amine hydrochloride salt in MeCN (0.09 M) was added 35% ammonia solution (2.5 eq.). This was stirred under a N_2 atmosphere for 5 minutes before adding a solution of Davis' oxaziridine (2.5 eq.) in toluene (0.7 M) dropwise over 45 minutes. The reaction was stirred at r.t. until starting material was

consumed as determined by TLC, at which point brine was introduced. The aqueous phase was extracted with EtOAc and the organic layers were combined and washed with brine. The organic phase was dried with $MgSO_4$ then filtered before removing the solvent under reduced pressure. The crude product was purified as in Oxidation Procedure A.

Note: During the course of this work, different nitrone oxidation methodologies were investigated. The two detailed in this S.I. offer complementary approaches to this challenging transformation: Oxidation Procedure A is typically lower yielding but uses far cheaper reagents – all of which are commercially available – and is more amenable to scale-up, hence, it was used for less elaborate starting materials where chemical yield was less important; Oxidation Procedure B tended to give a higher yield however faces difficulties associated with using superstoichiometric Davis' Oxaziridine, it was therefore used on smaller scales where material was more precious. Additional oxidation procedures investigated included using methyl trioxorhenium/UHP, mCPBA, and Oxone however were inferior to the two detailed above for the substrates in question.[3]

Cycloadduct Preparation: General Procedure A

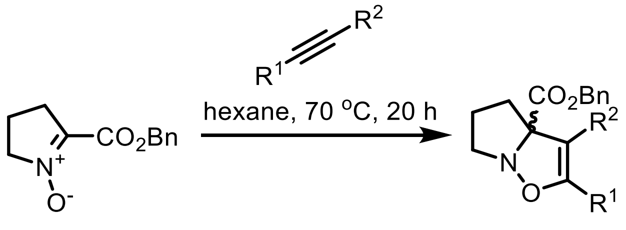

In an oven-dried sealable tube flushed with N_2, nitrone **1a** (100 mg, 0.456 mmol) was mixed with the respective alkyne (1.369 mmol) in hexane (4 mL) and the tube was sealed and stirred at 70 °C for 20 h - or until the starting material was consumed as determined by TLC. The hexane was removed under a stream of N_2 and the crude oil that resulted was purified by FCC.

Ketophosphonate Preparation I: General Procedure B

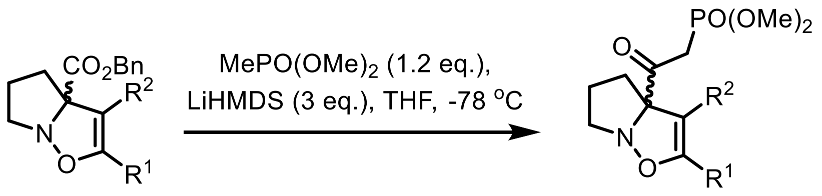

A solution of cycloadduct starting material (~0.156 mmol) and dimethyl methylphosphonate (1.2 eq.) in dry degassed THF (0.2 M) was added dropwise to 1M LiHMDS in THF (3 eq.) at -78 °C under an argon atmosphere. The reaction mixture was stirred until the starting material was consumed as determined by TLC and then quenched by the addition of sat. aq. NH_4Cl (2 mL). After dilution with EtOAc (5 mL) and H_2O (5 mL), the aqueous phase was extracted with EtOAc (3 x 20 mL) and the organic layers were combined, dried with $MgSO_4$ and filtered. The solvent was removed under reduced pressure to yield a yellow oil that was purified by FCC (EtOAc) to yield the ketophosphonates as light yellow oils.

Ketophosphonate Preparation II: Modification to General Procedure B

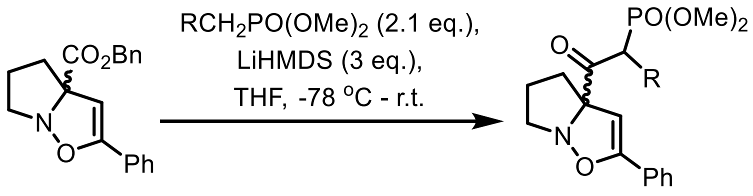

A solution of cycloadduct **2d** starting material (0.156 mmol) and the respective phosphonate (2.1 eq.) in dry degassed THF (0.2 M) was stirred at -78 °C under an argon atmosphere. 1M LiHMDS in THF (467 µL, 0.467 mmol) was added dropwise over 15 minutes and the reaction mixture was allowed to slowly warm to room temperature. After stirring at room temperature for 1 h, the solution was quenched with sat. aq. NH_4Cl (2 mL). The aqueous layer was diluted with H_2O (5 mL) and extracted with EtOAc (3 x 20 mL). The organic layers were combined, dried with $MgSO_4$ and filtered. The solvent was removed under reduced

pressure to yield yellow oils. Purification by FCC (2:1 EtOAc:pentane) yielded the substituted ketophosphonates as colourless oils.

Note: In some of the more challenging condensations, it was observed that holding the temperature at -78 °C for longer, e.g 4 hours, improved the yield.

Synthesis of N-Boc-Spiroamines: General Procedure C

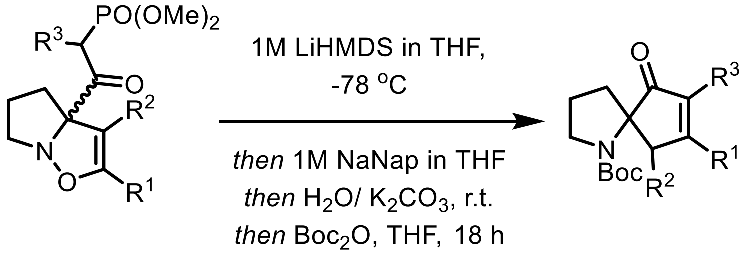

To a fully purged round-bottomed flask under argon, a solution of ketophosphonate in dry THF (0.05 M) was added and cooled to -78 °C. 1M LiHMDS in THF (1 eq.) was added dropwise and then stirred for 20 min. 1M sodium naphthalenide (NaNap) in THF (~3 eq.) was added dropwise until complete consumption of starting material was observed by TLC (9:1 EtOAc:MeOH). H_2O (0.5 mL) was then added, followed by addition of sat. aq. K_2CO_3 (1 mL) and the biphasic mixture was stirred for 1 h before addition of a solution of Boc_2O (2 eq.) in THF (1 mL). After stirring at r.t. for 18 h, the aqueous phase was diluted (2 mL H_2O) and then extracted with EtOAc (3 x 20 mL) and the organic layers were combined, dried with $MgSO_4$ and filtered before removal of the solvent under reduced pressure. The resulting crude material was purified by FCC.

Note: In the early stages of the dropwise addition of the NaNap solution to the reaction mixture, the dark green colour of the NaNap was observed to rapidly disappear upon reaction with the starting material. As the addition continued, the colour was observed to linger and at

this point TLC analysis was carried out to confirm consumption of starting material was proceeding.

Note: Early experimentation with this method highlighted a detrimental effect in using a significant excess of NaNap. As such care must be taken in the later stage of the addition with more frequent TLC analyses.

Note: Upon completion the reaction mixture was typically a light brown solution. Over-addition of NaNap was evident from the solution remaining dark green or often an insoluble dark solid would form, presumably precipitated NaNap caused by the cryogenic temperatures. This solid would slowly dissolve/react upon warming to room temperature.

Preparation of 1M Sodium Naphthalenide (NaNap)

To a solution of naphthalene (1.28 g, 10 mmol) in dry THF (10 mL) was added sodium metal (0.345 g, 15 mmol) and the reaction was stirred under an atmosphere of N_2 for 16 h. The dark green solution that resulted was used in General Procedure C without further purification.

3. Optimization of Cycloaddition and Phosphonate Condensation

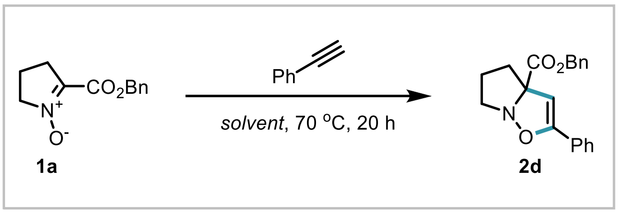

entry	solvent	yield
1	hexane	81%[a] (70%[b])
2	PhMe	73%[a]
3	THF	59%[a]
4	$CHCl_3$	47%[a]

Table S1. Solvent screen for model cycloaddition. [a] ^{1}H NMR yield using trimethoxybenzene as standard. [b]Isolated yield.

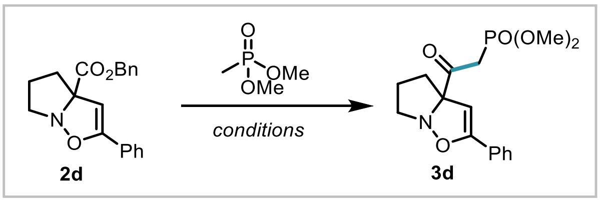

entry	base	phosphonate equivalents	[**2d**] (M)	yield
1[a]	*n*-BuLi (3 eq.)	3	0.022	14%
2[b]	*n*-BuLi (2 eq.)	3	0.022	56%
3[c]	*n*-BuLi (2 eq.)	2.3	0.022	*trace*
4[b]	*n*-BuLi (2.1 eq.)	2.3	0.2	60%
5[b]	LiHMDS (3 eq.)	1.2	0.2	67%
6[d]	LiHMDS (3 eq.)	1.2	0.2	70%

Table S2. Results from the phosphonate condensation optimisation. [a]Cycloadduct in THF added to Li-phosphonate solution. [b]THF degassed, Li-phosphonate formation stirred for 1 h, -78 °C maintained for an additional 2 h. [c]Reaction stirred overnight. [d] THF Solution of **2d** and phosphonate added dropwise to base.

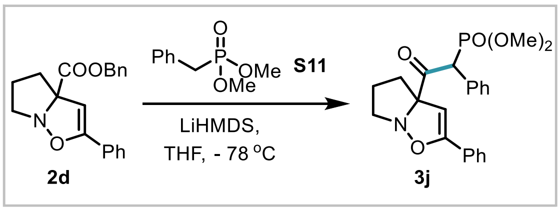

entry	base	phosphonate equivalents	[**2d**] (M)	yield
1	LiHMDS (3 eq.)	1.2	0.2	27%
2	LiHMDS (2 eq.)	2	0.2	39%
3	LiHMDS (2 eq.)	5	0.2	40%
4[a]	LDA (2.1 eq.)	1	0.2	*trace*
5[b]	LiHMDS (3 eq.)	2	0.2	60%

Table S3. Results from the optimisation of the synthesis of α-substituted β-ketophosphonates. [a]-5 °C to 0 °C.[4] [b]Reverse addition (base added to cycloadduct-phosphonate solution), rigorously anhydrous conditions.

4. Nitrone Syntheses

Synthesis of Nitrone 1a

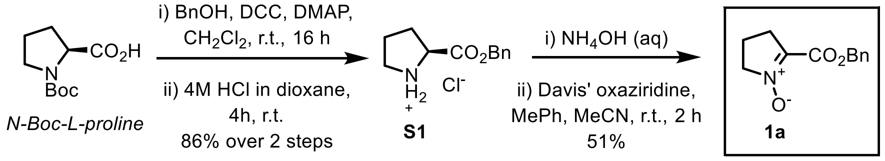

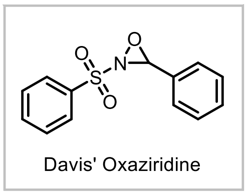

Scheme S1. Synthesis of Nitrone **1a**

(S)-1-((benzyloxy)carbonyl)pyrrolidin-5-ium chloride (S1)

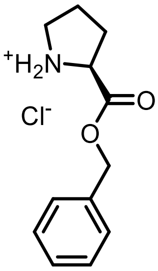

To a flask of N-Boc-proline (21.5 g, 100 mmol) dissolved CH_2Cl_2 (200 mL) was added DCC (22.7 g, 110 mmol), DMAP (1.2 g, 10 mmol) and benzyl alcohol (10.3 mL, 100 mmol). The reaction flask was stirred at r.t. for 16 h under a N_2 atmosphere before being cooled to -10 °C and filtered in *vacuo*. The filtrate was concentrated under reduced pressure before being purified by FCC (3:1 pentane:EtOAc) to afford the benzyl ester of N-Boc-proline as a light yellow oil. The yield was assumed quantitative and the product was stirred in 4M HCl in dioxane (100 mL, 400 mmol) under a N_2 atmosphere for 4 h. Et_2O was added to the reaction mixture until a white solid was observed at which point the reaction was cooled to 0 °C. The mixture was filtered in *vacuo* to yield the title compound as a white solid (20.4 g, 85% *over 2 steps*). **MP** 148-149 °C (lit. 141-143 °C)[5]; **^{1}H NMR** (400 MHz, $CDCl_3$) δ 10.60 (br s, 1H), 9.24 (br s, 1H), 7.35 – 7.27 (m, 5H), 5.25 – 5.07 (m, 2H), 4.48 (m, 1H), 3.59 – 3.37 (m, 2H), 2.41 – 2.28 (m, 1H), 2.20 – 1.90 (m, 3H); **^{13}C NMR** (101 MHz, $CDCl_3$) δ 168.7, 134.5, 128.7, 128.7, 128.5, 68.3, 59.3, 45.9, 28.7, 23.5; **LRMS** (ESI) mass calculated for [M+H]+ ($C_{12}H_{16}NO_2$) requires *m/z* 206.1, found *m/z* 206.2. Data are consistent with the literature.[5]

1-((Benzyloxy)carbonyl)-3,4-dihydro-2H-pyrrole 5-oxide (1a)

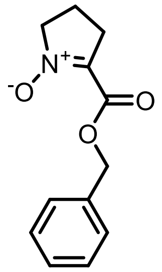

Using Oxidation Procedure A, the hydrochloride salt of proline benzyl ester **S1** (7.8 g, 32 mmol) was submitted to the reaction conditions. FCC purification (19:1 EtOAc:MeOH) afforded nitrone **1a** (2.57 g, 37%) as a colourless oil.

Using Oxidation Procedure B, hydrochloride salt **S1** (1.00 g, 4.15 mmol) was submitted to the reaction conditions. FCC purification (19:1 EtOAc:MeOH) afforded nitrone **1a** (463 mg, 51%) as a colourless oil.

IR (thin film) ν_{max}/cm^{-1} 2953 (C-H), 1723 (C=O), 1696, 1553 (C=N), 1221 (N-O); **^{1}H NMR** (400 MHz, $CDCl_3$) δ 7.47 – 7.31 (m, 5H), 5.31 (app. d, *J* = 1.1 Hz, 2H), 4.27 – 4.15 (m, 2H), 3.13 – 3.02 (m, 2H), 2.25 – 2.14 (m, 2H);**^{13}C NMR** (101 MHz, $CDCl_3$) δ 159.3, 135.3, 133.8, 128.6, 128.4, 128.4, 66.8, 66.7, 29.7, 16.8; **HRMS** (ESI) mass calculated for [M+H]+ ($C_{12}H_{14}O_3N$) requires *m/z* 220.0968, found *m/z* 220.0971.

Synthesis of Nitrone 1b

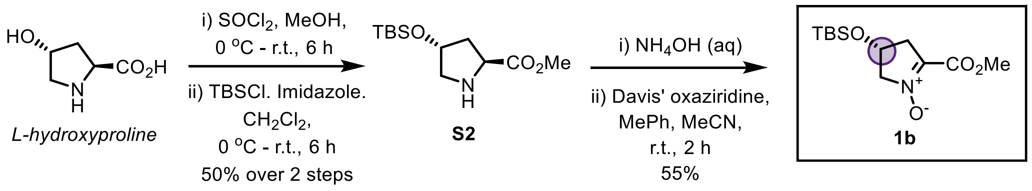

Scheme S2. Synthesis of Nitrone 1b

Methyl (1S,3R)-3-((tert-butyldimethylsilyl)oxy)pyrrolidine-1-carboxylate (S2)

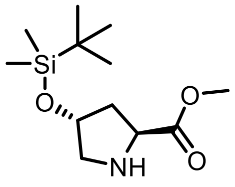

Following adapted literature procedures,[6,7,8] $SOCl_2$ (2.0 mL, 27 mmol) was added dropwise to a stirred and cooled (0 °C) solution of trans-4-hydroxy-L-proline (3.0 g, 23 mmol) in MeOH (38 mL). After 30 min, the

ice bath was removed and stirring was continued for 6 h. Evaporation of the solvent gave the ester hydrochloride as an off white solid (yield assumed quant.).

To a solution of the crude product and imidazole (9.4 g, 138 mmol) in CH_2Cl_2 (48 mL), was added TBSCl (4.1 g, 27 mmol) at 0 °C under N_2 atmosphere. After stirring for 6 h at r.t., the reaction was quenched with water (30 mL) and extracted with CH_2Cl_2 (2 x 50 mL). The combined organic layers were dried with $MgSO_4$, filtered and concentrated under reduced pressure. The residue was purified by FCC (pentane:EtOAc 1:1) to give the title compound as a colourless oil (2.97 g, 50%). **^{1}H NMR** (400 MHz, $CDCl_3$) δ 4.42 – 4.33 (m, 1H), 3.99 (t, *J* = 8.0 Hz, 1H), 3.72 (s, 3H), 3.10 (dd, *J* = 11.5, 4.5 Hz, 1H), 2.84 (app. ddt, *J* = 11.4, 2.3, 1.1 Hz, 1H), 2.05 (m, 2H), 1.95 (ddd, *J* = 13.2, 8.0, 5.4 Hz, 1H), 0.87 (s, 9H), 0.06 (s, 3H), 0.05 (s, 3H); **^{13}C NMR** (101 MHz, $CDCl_3$) δ 175.5, 73.0, 58.7, 55.9, 52.1, 40.1, 25.8, 18.1, -4.8; **LRMS** (ESI) mass calculated for [M+H]+ ($C_{12}H_{25}NO_3Si$) requires *m/z* 260.2, found *m/z* 260.4. All data are consistent with the literature.[8]

(R)-3-((Tert-butyldimethylsilyl)oxy)-1-(methoxycarbonyl)-3,4-dihydro-2H-pyrrole 5-oxide (1b)

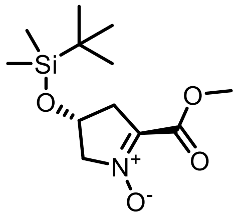

Oxidation Procedure B was followed using **S2** (1.0 g, 4.0 mmol). The reaction was determined to be complete by TLC after stirring at r.t. for 5 h. Purification by FCC (2:1 pentane: EtOAc) yielded the title compound as a white solid (575 mg, 55%). **MP** 88-89 °C; **IR** (thin film) ν_{max}/cm^{-1} 2954 (C-H), 1751 (C=O), 1698, 1552 (C=N), 1252 (N-O), 1086 (C-O); **^{1}H NMR** (400 MHz, $CDCl_3$) δ 4.53 (tt, *J* = 6.2, 2.1 Hz, 1H), 4.33 (ddt, *J* = 14.9, 6.0, 1.4 Hz, 1H), 3.99 (ddt, *J* = 14.9, 2.5, 1.4 Hz, 1H), 3.86 (s, 3H), 3.27 (ddt, *J* = 18.4, 6.5, 1.4 Hz, 1H), 2.95 (app. dq, *J* = 18.3, 1.4 Hz, 1H), 0.88 (s, 9H), 0.09 (s, 3H), 0.08 (s, 3H); **^{13}C NMR** (101 MHz, $CDCl_3$) δ 159.9, 132.4, 75.1,

63.6, 52.3, 40.8, 25.6, 17.9, -4.8, -4.9; **HRMS** (ESI) mass calculated for [M+H]+ ($C_{12}H_{24}O_4NSi$) requires *m/z* 274.1469, found *m/z* 274.1465.

Synthesis of Nitrone 1c

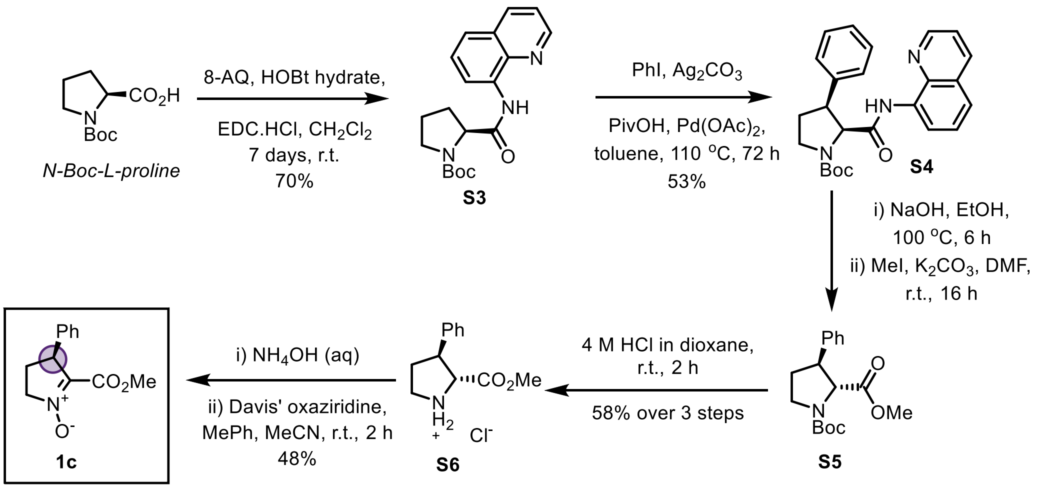

Scheme S3. Synthesis of Nitrone 1c

Tert-butyl (S)-1-(quinolin-8-ylcarbamoyl)pyrrolidine-5-carboxylate (S3)

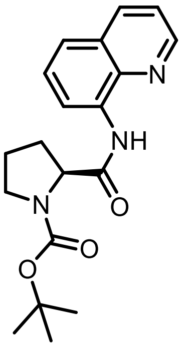

Following a literature procedure,[9] HOBt hydrate (2.76 g, 15 mmol) was added to a solution of N-Boc-proline (3.23 g, 15 mmol) and 8-aminoquinoline (2.60 g, 18 mmol) in CH_2Cl_2 (60 mL) at r.t., and was stirred for 5 min. EDC.HCl (3.45 g, 18 mmol) was then added and the solution was stirred for 7 days. The reaction was diluted with CH_2Cl_2 (100 mL) and sat. aq. $NaHCO_3$ (100 mL) was added. The aqueous layer was extracted with CH_2Cl_2 (3 x 100 mL) and the combined organic extracts were dried with Na_2SO_4, filtered and the solvent was removed under reduced pressure. The resulting brown solid was purified by FCC (grading 9:1 to 4:1 pentane:EtOAc) to yield the title compound as an off-white solid (3.60 g, 70%). The product was characterised as a mixture of rotamers. **MP** 147-149 °C (lit. 136-139 °C)[9]; **^{1}H NMR** (400 MHz, DMSO-d_6) δ 10.41 (br s, 0.4H), 10.26 (br s, 0.6H), 8.88 (s, 1H), 8.70 – 8.57 (m, 1H), 8.39 (dd, *J* = 8.3, 1.7 Hz, 1H), 7.70 – 7.52 (m, 3H), 4.48 (dd, *J* = 8.5, 4.1 Hz,

1H), 3.53 – 3.37 (m, 2H), 2.31 – 2.11 (m, 1H), 2.02 (d, *J* = 22.5 Hz, 1H), 1.92 – 1.77 (m, 2H), 1.43 (s, 4H), 1.21 (s, 5H); **^{13}C NMR** (101 MHz, DMSO-d_6) δ 172.0, 171.3, 153.9, 149.4, 138.4, 137.1 , 134.5, 128.3, 127.5, 122.7, 122.4, 116.5, 79.8, 79.5, 61.7, 47.2, 31.4, 30.2, 28.6, 28.3, 24.5, 23.9; **LRMS** (ESI) mass calculated for [M+Na]+ ($C_{19}H_{23}N_3NaO_3$) requires *m/z* 364.2, found *m/z* 364.2. The data were consistent with the literature.[9*]

Tert-butyl (2S,3S)-2-phenyl-1-(quinolin-8-ylcarbamoyl)pyrrolidine-5-carboxylate (S4)

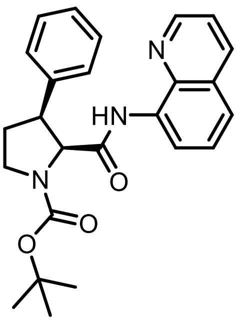

Following a literature procedure,[9] a screw-cap tube was charged with tert-butyl (S)-2-(quinolin-8-ylcarbamoyl)pyrrolidine-1-carboxylate **S3** (1.00 g, 2.9 mmol), Ag_2CO_3 (810 mg, 2.9 mmol), pivalic acid (90 mg, 0.88 mmol), phenyl iodide (980 µL, 8.8 mmol), $Pd(OAc)_2$ (66 mg, 0.29 mmol), and toluene (10 mL). The reaction vessel was flushed with argon, sealed and placed in an oil bath (preheated to 110 °C) and stirred for 72 h. The reaction mixture was cooled to r.t. and EtOAc (50 mL) was added. The resulting solution was filtered through a pad of Celite®, eluting with further EtOAc (2 x 50 mL). The solvent was removed under reduced pressure to yield a brown oil. Purification by FCC (grading 1% to 5% MeCN/ CH_2Cl_2) yielded the title compound as an off-white solid (642 mg, 53%). The product was characterised as a mixture of rotamers. **MP** 125-129 °C (lit 118-120 °C)[9]; **^{1}H NMR** (400 MHz, DMSO-d_6) δ 9.72 (br s, 0.4H), 9.68 (br s, 0.5H), 8.83 (d, *J* = 5.1 Hz, 1H), 8.36 – 8.27 (m, 2H), 7.61 – 7.53 (m, 2H), 7.47 (t, *J* = 7.9 Hz, 1H), 7.33 (t, *J* = 7.8 Hz, 2H), 7.09 (t, *J* = 7.2 Hz, 2H), 6.96 (t, *J* = 7.4 Hz, 1H), 4.86 (d, *J* = 8.5 Hz, 1H), 3.86 – 3.74 (m, 2H), 3.55 – 3.38 (m, 1H), 2.57 (app. q, *J* = 10.9 Hz, 1H), 2.11 (app. dd, *J* = 10.1, 4.4 Hz, 1H), 1.43 (s, 3H), 1.22 (s, 6H); **^{13}C NMR** (101 MHz, DMSO-d_6) δ 169.9, 153.7, 149.1, 138.2, 137.7, 136.9, 134.2, 128.5, 128.4, 128.0, 127.3, 127.2, 122.5, 122.1, 116.4, 79.4, 65.7, 47.9, 46.3,

[*] Literature NMR data were taken at 373 K hence the lack of observable rotamers previously documented.

28.6, 28.3, 27.7; **LRMS** (ESI) mass calculated for [M+H]+ ($C_{25}H_{28}N_3O_3$) requires *m/z* 418.2, found *m/z* 418.7. All data were consistent with the literature.[9*]

(1R, 2S)-1-(Methoxycarbonyl)-2-phenylpyrrolidin-5-ium chloride (S6)

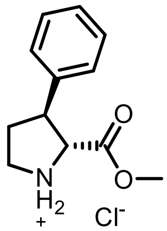

Following a modified literature procedure,[10] a screw-cap tube was charged with **S4** (930 mg, 2.2 mmol), sodium hydroxide (890 mg, 22 mmol) and then dissolved in EtOH (12 mL). The tube was heated to 100 °C for 6 h before cooling to r.t.. The mixture was then diluted with EtOAc (12 mL) and 1M aq. NaOH (12 mL). The aqueous layer was collected and the organic layer was extracted with 1 M aq. NaOH (3 x 60 mL). All the aqueous layers were combined and washed with EtOAc (120 mL), then acidified to pH 2-3 with conc. HCl. This acidic layer was then extracted with EtOAc (4 x 240 mL). The combined organics were dried with Na_2SO_4, filtered and the solvent was removed under reduced pressure. The resulting brown oil was then triturated with Et_2O then pentane to yield a pale red solid which was used without purification. The crude carboxylic acid was dissolved in DMF (6 mL) and anhydrous K_2CO_3 (405 mg, 2.9 mmol) was added at 0 °C and the mixture was stirred for 10 min. MeI (180 µL, 2.9 mmol) was added and the reaction mixture was stirred for 18 h at r.t.. This was then filtered and the filtrate was dissolved in H_2O (20 mL). The aqueous layer was extracted with EtOAc (3 x 40 mL) and the organics were combined and washed with brine (20 mL). The organics were then dried with Na_2SO_4, filtered and the solvent was removed under reduced pressure to yield the crude product as a dark brown oil. Purification by FCC (3:1 pentane: EtOAc) yielded the Boc-protected methyl ester **S5**. The resulting colourless oil was treated with 4 M HCl in dioxane (1.5 mL) under a N_2 atmosphere for 2 h. Et_2O was then introduced until precipitation of an off-white solid which was collected by filtration. Washing with pentane yielded the title

[*] Literature NMR data were taken at 373 K hence the lack of observable rotamers in previous spectra.

compound as an off-white solid (305 mg, 58% over 3 steps). **MP** 168-172 °C; **IR** (thin film) ν_{max}/cm^{-1} 3427 (N-H), 3031 (C-H), 1744 (C=O), 909; **^{1}H NMR** (500 MHz, $CDCl_3$) δ 11.17 (br s, 1H), 9.58 (br s, 1H), 7.43 – 7.36 (m, 4H), 7.33 – 7.29 (m, 1H), 4.53 (d, *J* = 9.5 Hz, 1H), 3.86 – 3.79 (m, 1H), 3.74 (s, 3H), 3.65 (2H), 2.54 (dtd, *J* = 13.8, 7.0, 2.8 Hz, 1H), 2.37 (dtd, *J* = 13.2, 10.5, 7.9 Hz, 1H); **^{13}C NMR** (126 MHz, $CDCl_3$) δ 168.8, 138.1, 129.1, 127.9, 127.6, 64.5, 53.44, 48.6, 46.2, 34.0; **HRMS** (ESI) mass calculated for [M-Cl]+ ($C_{12}H_{16}O_2N$) requires *m/z* 206.1176, found *m/z* 206.1175

(S)-5-(Methoxycarbonyl)-4-phenyl-3,4-dihydro-2H-pyrrole 1-oxide (1c)

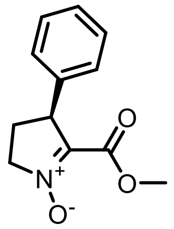

Oxidation Procedure B was followed using **S6** (200 mg, 0.83 mmol). The reaction was determined to be complete by TLC after stirring at r.t. for 6 h. Purification by FCC (EtOAc) yielded the title compound as an off-white solid (87 mg, 48%). **MP** 116-118 °C; **IR** (thin film) ν_{max}/cm^{-1} 3028 (C-H), 1722 (C=O), 1697, 1544 (C=N), 1222 (N-O); **^{1}H NMR** (400 MHz, $CDCl_3$) δ 7.42 – 7.31 (m, 2H), 7.31 – 7.24 (m, 1H, 7.24 – 7.18 (m, 2H), 4.58 – 4.48 (m, 1H), 4.40 (dddd, *J* = 14.5, 9.1, 7.7, 2.1 Hz, 1H), 4.21 (dddd, *J* = 14.6, 9.8, 5.5, 1.2 Hz, 1H), 3.69 (s, 3H), 2.73 (app. dtd, *J* = 13.2, 9.6, 7.7 Hz, 1H), 2.10 (dddd, *J* = 13.3, 9.1, 5.5, 4.3 Hz, 1H); **^{13}C NMR** (101 MHz, $CDCl_3$) δ 159.8, 141.4, 136.5, 129.0, 127.5, 126.8, 65.6, 52.1, 48.1, 27.5; **HRMS** (ESI) mass calculated for [M+H]+ ($C_{12}H_{14}O_3N$) requires *m/z* 220.0968, found *m/z* 220.0969.

Synthesis of Nitrone 1d

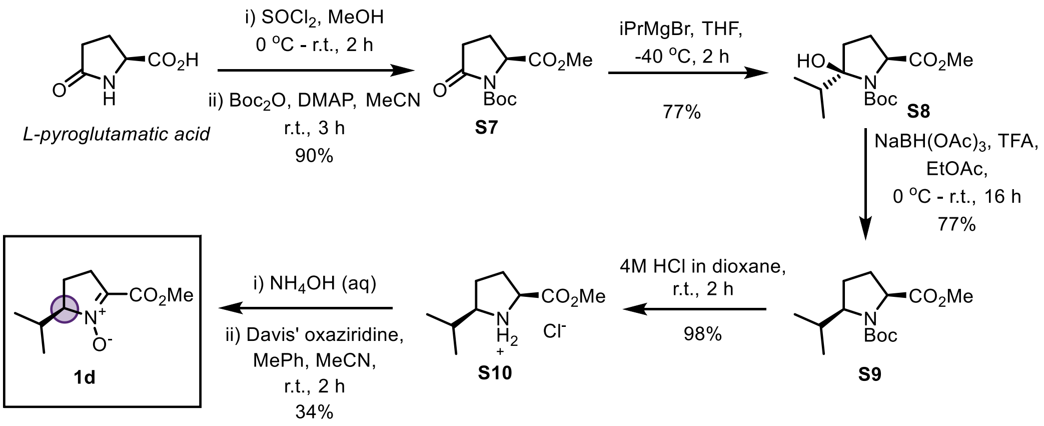

Scheme S4. Synthesis of Nitrone **1d**

1-(Tert-butyl) 2-methyl (S)-5-oxopyrrolidine-1,2-dicarboxylate (S7)

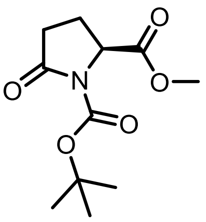

Following a literature procedure,[11] to a stirred solution of L-pyroglutamic acid (2.0 g, 15 mmol) in MeOH (24 mL) at 0 °C was added $SOCl_2$ (2.26 mL, 31 mmol) dropwise over 5 min. The mixture was allowed to warm to r.t. and stirred for 2 h. The reaction mixture was concentrated under reduced pressure and the resulting yellow oil was dissolved in CH_2Cl_2 (50 mL). The organic solution was washed with sat. aq. $NaHCO_3$ (25 mL) and brine (25 mL). The combined aqueous layers were further extracted with CH_2Cl_2 (5 x 25 mL). The combined organics were then dried over $MgSO_4$, filtered and concentrated under reduced pressure.

The resulting yellow oil was dissolved in MeCN (4 mL) and Boc_2O (3.93 g, 18 mmol) and DMAP (183 mg, 1.5 mmol) was added. After 3 h the reaction mixture was concentrated to a dark orange oil. Purification by FCC (4:1 CH_2Cl_2: EtOAc) followed by recrystallization (EtOAc/Et_2O) yielded the desired product as white crystals (3.39 g, 90%). **^{1}H NMR** (400 MHz, $CDCl_3$) δ 4.60 (dd, J = 9.4, 3.0 Hz, 1H), 3.77 (s, 3H), 2.68 – 2.56 (m, 1H), 2.48 (ddd, J = 17.5, 9.4, 3.7 Hz, 1H), 2.31 (ddt, J = 13.4, 10.1, 9.4 Hz, 1H), 2.02 (dddd, J = 13.3, 9.6, 3.6, 3.0 Hz, 1H), 1.47 (s, 9H); **^{13}C NMR** (101 MHz, $CDCl_3$) δ 173.2, 171.8, 149.3, 83.6, 58.8,

52.5, 31.1, 27.9, 21.5; **LRMS (ESI)** mass calculated for [M+Na]+ ($C_{11}H_{17}NNaO_5$) requires *m/z* 266.1, found *m/z* 266.0. All data are consistent with the literature.[11]

1-(Tert-butyl) 2-methyl (2S,5R)-5-hydroxy-5-isopropylpyrrolidine-1,2-dicarboxylate (S8)

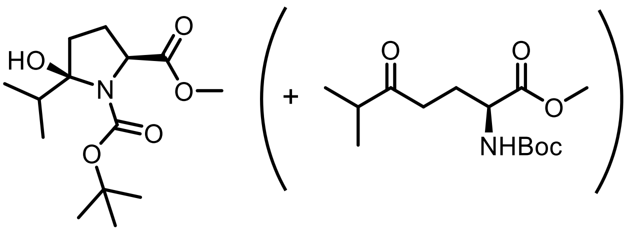

Following a literature procedure,[12] iPrMgBr (2 mL, 3 M in 2-MeTHF, 6.2 mmol) was added at -40 °C to a stirred solution of **S7** (1.0 g, 4.1 mmol) in anhydrous THF (12 mL). After 2 h, the reaction was quenched with sat. aq. NH_4Cl (15 mL). The resulting aqueous layer was extracted with CH_2Cl_2 (3 x 60 mL). The combined organic layers were dried with $MgSO_4$, filtered and concentrated under reduced pressure. Purification by FCC (4:1 to 1:1 pentane:EtOAc) yielded the title compound as a colourless oil (913 mg, 77%) with 10% existing as the open chain isomeric ketone. As such, the mixture was carried forward and not fully characterised. **LRMS** (ESI) mass calculated for [M+Na]+ ($C_{14}H_{25}NO_5$) requires *m/z* 310.2, found *m/z* 310.5.

1-(Tert-butyl) 2-methyl (2S,5R)-5-isopropylpyrrolidine-1,2-dicarboxylate (S9)

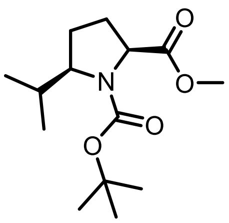

Following a literature procedure,[12] $NaBH(OAc)_3$ (816 mg, 3.9 mmol) was added at 0 °C to a stirred solution of **S8** (850 mg, 3.0 mmol) in EtOAc (12 mL). After 10 min, TFA (750 µL, 9.8 mmol) was added dropwise over a period of 40 min. The mixture was stirred at 0 °C for 2 h and then at r.t. for 16 h. Sat. aq. $NaHCO_3$ (35 mL) was added and most of the organic solvent was removed under reduced pressure. CH_2Cl_2 (35 mL) was added and the layers were separated. The organic layer was washed with sat. aq. $NaHCO_3$ (2 x 35 mL). The combined aqueous layers were re-extracted with CH_2Cl_2 (2 x 50 mL). The combined organic layers were then

washed with brine (35 mL), dried with $MgSO_4$, filtered and the solvent removed under reduced pressure. The resulting yellow oil was purified by FCC (6:1 pentane:EtOAc) to yield the desired product as a colourless oil (627 mg, 77%). The product was characterised as a mixture of rotamers. **^{1}H NMR** (400 MHz, $CDCl_3$) δ 4.28 (d, *J* = 8.8 Hz, 0.4H), 4.24 – 4.13 (m, 0.6H), 3.68 (dd, *J* = 3.3, 1.6 Hz, 3.6H), 3.59 (m, 0.4H), 2.27 – 2.10 (m, 1H), 2.00 (m, 0.4H), 1.91 – 1.83 (m, 1.4H), 1.78 (dq, *J* = 7.2, 3.4 Hz, 2H), 1.44 – 1.39 (m, 4H), 1.38 – 1.33 (m, 5H), 0.95 – 0.91 (m, 3H), 0.87 (m, 3H). **^{13}C NMR** (101 MHz, $CDCl_3$) δ 173.9, 155.1, 154.4, 79.8, 79.7, 64.3, 64.1, 60.4, 59.9, 51.9, 51.7, 31.4, 31.2, 29.1, 28.3, 28.2, 27.4, 26.4, 20.0, 19.8, 18.5, 18.1. **LRMS** (ESI) mass calculated for [M+Na]+ ($C_{14}H_{25}NO_4Na$) requires *m/z* 294.2, found *m/z* 294.2. All data are consistent with the literature.[12]

(2R,5S)-2-Isopropyl-5-(methoxycarbonyl)pyrrolidin-1-ium chloride (S10)

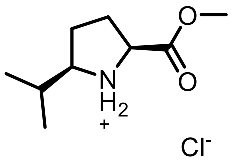

4M HCl in dioxane (2 mL, 7.38 mL) was added to **S9** (500 mg, 1.85 mmol) and stirred at r.t. for 2 h. Et_2O was added dropwise until a white solid was formed. The white solid was collected by filtration and washed with pentane to yield the desired product as a white solid (376 mg, 98%). **MP** 155-156 °C**;** **IR** (thin film) ν_{max}/cm^{-1} 3426 (N-H), 2962 (C-H), 1748 (C=O), 1236; **^{1}H NMR** (400 MHz, $CDCl_3$) δ 11.88 (br s, 1H), 7.38 (br s, 1H), 4.50 (app. ddt, *J* = 9.9, 6.9, 3.8 Hz, 1H), 3.79 (s, 3H), 3.52 – 3.41 (m, 1H), 2.38 – 2.26 (m, 1H), 2.21 (ddt, *J* = 13.6, 7.3, 3.1 Hz, 1H), 2.09 (dddd, *J* = 13.4, 9.0, 5.9, 2.4 Hz, 2H), 1.65 (dtd, *J* = 13.2, 11.1, 7.4 Hz, 1H), 1.16 (d, *J* = 6.6 Hz, 3H), 0.96 (d, *J* = 6.7 Hz, 3H); **^{13}C NMR** (101 MHz, $CDCl_3$) δ 170.1, 67.5, 59.1, 53.8, 30.8, 28.7, 27.8, 20.7, 19.2; **HRMS (ESI)** mass calculated for [M-Cl]+ ($C_9H_{18}O_2N$) requires *m/z* 172.1332, found *m/z* 172.1332.

(R)-2-Isopropyl-5-(methoxycarbonyl)-3,4-dihydro-2H-pyrrole 1-oxide (1d)

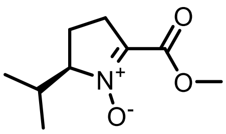

Oxidation Procedure B was followed using **S10** (250 mg, 1.21 mmol). The reaction was determined to be complete by TLC after stirring at r.t. for 7 h. Purification by FCC (1:19 MeOH:EtOAc) yielded the title compound as a white solid (75 mg, 34%) **MP** 40-42 °C; **IR** (thin film) ν_{max}/cm^{-}1 2961 (C-H), 1726 (C=O), 1694, 1545 (C=N), 1223 (N-O), 1195; **^{1}H NMR** (400 MHz, $CDCl_3$) δ 4.16 – 4.07 (m, 1H), 3.83 (s, 3H), 2.97 – 2.80 (m, 2H), 2.63 (app. ddp, *J* = 10.8, 6.9, 3.8 Hz, 1H), 2.11 (dtd, *J* = 13.4, 9.4, 5.9 Hz, 1H), 1.94 (ddt, *J* = 13.2, 9.3, 6.5 Hz, 1H), 0.96 (d, *J* = 7.1 Hz, 3H), 0.83 (d, *J* = 6.7 Hz, 3H); **^{13}C NMR** (101 MHz, $CDCl_3$) δ 160.4, 133.2, 81.7, 52.2, 28.6, 28.0, 18.7, 17.0, 14.7; **HRMS** (ESI) mass calculated for [M+H]+ ($C_9H_{16}O_3N$) requires *m/z* 186.1125, found *m/z* 186.1125.

5. Synthesis of Phosphonates

Dimethyl benzylphosphonate (S11)

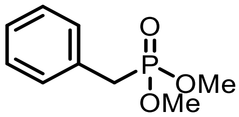

Following an adapted literature procedure,[13] to a solution of trimethylphosphite (5.89 ml, 50 mmol) in an equal volume of toluene was added benzyl bromide (0.595 mL, 5 mmol) and the mixture was heated to 110 °C for 20 h. The solvent and excess trimethyl phosphite were then removed under reduced pressure to yield the crude product as a colourless oil. This was purified by FCC (EtOAc) (95:5 EtOAc:MeOH) to yield the desired product with 11% impurity of trimethylphosphite. This was re-columned in the same conditions to afford the pure title compound as a colourless oil (498 mg, 50%). **^{1}H NMR** (400 MHz, $CDCl_3$) δ 7.32 – 7.17 (m, 5H), 3.62 (d, *J* = 10.8 Hz, 6H), 3.12 (d, *J* = 21.8 Hz, 2H); **^{13}C NMR** (101 MHz, $CDCl_3$) δ 131.3 (d, *J* = 9.4 Hz), 129.7 (d, *J* = 6.4 Hz), 128.6 (d, *J* = 3.2 Hz), 127.0 (d, *J* = 3.4 Hz), 52.9 (d, *J* = 6.4 Hz), 32.9 (d, *J* = 138.3 Hz); **^{31}P NMR** (162 MHz, $CDCl_3$) δ 28.81; **LRMS (ESI)** mass calculated for [M+Na]+

($C_9H_{13}NaO_3P$) requires *m/z* 223.1, found *m/z* 223.0. All data were consistent with the literature.[14]

Alkylation Procedure

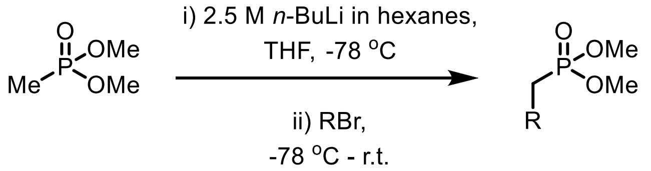

Following an adapted literature procedure,[15] a solution of dimethyl methylphosphonate (540 µL, 5.0 mmol) in dry THF (10 mL) was cooled to –78 °C under a N_2 atmosphere before adding 2.5 M solution of *n*-BuLi in hexanes (2.4 mL, 6.0 mmol). This was stirred for 20 min before addition of the alkyl halide (1.3 eq.). The reaction was allowed to slowly warm to r.t. and then stirred for 20 h. Reaction completion was determined by TLC at which point it was quenched by brine (20 mL). The aqueous phase was extracted with Et_2O (3 x 50 mL). The combined organics were dried with $MgSO_4$, filtered and the solvent was removed under reduced pressure. The crude product was purified by FCC (2% MeOH in EtOAC as eluent) to yield the phosphonates as oils.

Dimethyl phenethylphosphonate (S12)

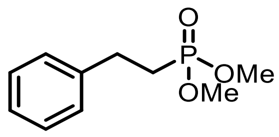

The general alkylation procedure was followed using benzyl bromide (770 µL, 6.5 mmol) to afford the title compound as a light yellow oil (687 mg, 64%). **^{1}H NMR** (400 MHz, $CDCl_3$) δ 7.44 – 7.32 (m, 2H), 7.32 – 7.20 (m, 3H), 3.80 (app. dd, *J* = 10.8, 0.7 Hz, 6H), 3.05 – 2.92 (m, 2H), 2.22 – 2.08 (m, 2H); **^{13}C NMR** (101 MHz, $CDCl_3$) δ 140.8 (d, *J* = 17.4 Hz), 128.6, 128.0, 126.4, 52.3 (d, *J* = 7.0 Hz), 28.5 (d, *J* = 4.6 Hz), 26.6 (d, *J* = 139.1 Hz); **^{31}P NMR** (162 MHz, $CDCl_3$) δ 33.38; **LRMS** (ESI) mass calculated for [M+Na]+ ($C_{10}H_{15}NaO_3P$) requires *m/z* 237.0, found *m/z* 237.0. All data were consistent with the literature.[16]

Dimethyl but-3-en-1-ylphosphonate (S13)

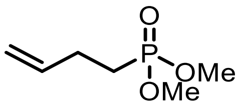

The general alkylation procedure was followed using allyl bromide (560 μL, 6.5 mmol) to afford the title compound as a colourless oil (470 mg, 57%). **^{1}H NMR** (400 MHz, $CDCl_3$) δ 5.91 – 5.78 (m, 1H), 5.13 – 4.99 (m, 2H), 3.75 (app. dq, *J* = 10.8, 1.0 Hz, 6H), 2.40 – 2.30 (m, 2H), 1.92 – 1.78 (m, 2H); **^{13}C NMR** (101 MHz, $CDCl_3$) δ 137.0 (d, *J* = 17.3 Hz), 115.2, 52.3 (d, *J* = 6.4 Hz), 26.4 (d, *J* = 4.4 Hz), 24.0 (d, *J* = 140.8 Hz); **^{31}P NMR** (162 MHz, $CDCl_3$) δ 34.07; **LRMS** (ESI) mass calculated for [M+Na]+ ($C_6H_{13}NaO_3P$) requires *m/z* 187.0, found *m/z* 187.0. All data were consistent with the literature.[17]

6. Synthesis and Characterisation of Cycloadducts

Synthesis of **2a**

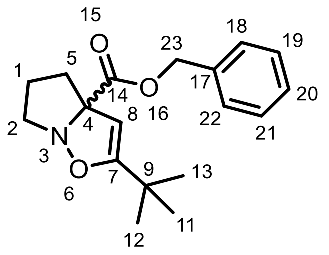

General Procedure A was followed using *t*-Bu-acetylene (334 μL, 2.74 mmol) and stirred for 74 h. The resulting oil was purified by FCC (4:1 pentane:EtOAc) to give the desired product as an amorphous white solid (92 mg, 67%). **IR** (thin film) ν_{max}/cm^{-1} 2965 (C-H), 1735 (C=O), 1663 (C=C), 1235; **^{1}H NMR** (400 MHz, $CDCl_3$) δ 7.4 – 7.3 (m, 5H, 18-22), 5.2 (d, *J* = 12.5 Hz, 1H, 23), 5.2 (d, *J* = 12.6 Hz, 1H, 23’), 4.4 (s, 1H, 8), 3.4 – 3.2 (m, 2H, 2), 2.1 (ddd, *J* = 12.6, 8.3, 7.5 Hz, 1H, 5), 2.0 – 1.9 (m, 1H, 5’), 1.9 – 1.7 (m, 2H, 1), 1.1 (s, 9H, 11-13); **^{13}C NMR** (101 MHz, $CDCl_3$) δ 173.2 (14), 165.3 (7), 136.0 (17), 128.5 (19, 21), 128.1 (20), 127.8 (18, 22), 92.0 (8), 82.2 (4), 66.6 (23), 60.0 (2), 36.9 (5), 31.3 (9), 28.1 (11-13), 23.0 (1); **HRMS** (ESI) mass calculated for [M+H]+ ($C_{18}H_{24}O_3N$) requires *m/z* 302.1751, found *m/z* 302.1747.

Synthesis of **2b**

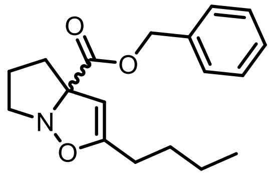

General Procedure A was followed, however 6 equivalents of alkyne were used. Thus 1-hexyne (310 μL, 2.74 mmol) was added and the reaction mixture was stirred for 20 h. The crude oil that resulted was purified by FCC (4:1 pentane:EtOAc) to give the title compound as a pale yellow oil (47 mg, 34%). **IR** (thin film) ν_{max}/cm^{-1} 3033 (C-H), 1737 (C=O), 1250, 843; **^{1}H NMR** (400 MHz, $CDCl_3$) δ 7.40 – 7.28 (m, 5H), 5.20 (app. d, *J* = 1.0 Hz, 2H), 4.50 (t, *J* = 1.1 Hz, 1H), 3.36 – 3.29 (m, 2H), 2.19 – 2.09 (m, 3H), 2.00 (dddd, *J* = 12.5, 6.6, 4.8, 0.7 Hz, 1H), 1.90 – 1.76 (m, 2H), 1.52 – 1.43 (m, 2H), 1.38 – 1.27 (m, 2H), 0.89 (t, *J* = 7.3 Hz, 3H); **^{13}C NMR** (101 MHz, $CDCl_3$) δ 173.2, 157.6, 136.0, 128.5, 128.1, 127.9, 94.4, 82.4, 66.7, 6.13, 36.9, 28.8, 25.5, 23.2, 22.2, 13.8; **HRMS** (ESI) mass calculated for [M+H]+ ($C_{18}H_{24}O_3N$) requires *m/z* 302.1751, found *m/z* 302.1750.

Synthesis of **2c**

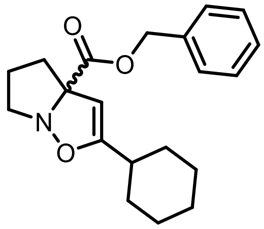

General Procedure A was followed using cyclohexylacetylene (360 μL) and stirred for 22 h. The resulting oil was purified by FCC (4:1 pentane:EtOAc) to give the title compound as a colourless oil (71 mg, 52%). **IR** (thin film) ν_{max}/cm^{-1} 3034 (C-H), 1735 (C=O), 1172, 1016; **^{1}H NMR** (400 MHz, $CDCl_3$) δ 7.37 – 7.28 (m, 5H), 5.24 – 5.15 (m, 2H), 4.45 (s, 1H), 3.30 (dd, *J* = 7.7, 5.8 Hz, 2H), 2.13 (m, 2H), 2.03 – 1.94 (m, 1H), 1.92 – 1.75 (m, 4H), 1.75 – 1.60 (m, 2H), 1.33 – 1.11 (m, 6H); **^{13}C NMR** (101 MHz, $CDCl_3$) δ 173.2, 161.9, 136.0, 128.5, 128.1, 127.8, 92.6, 82.2, 66.6, 60.1, 36.9, 35.3, 30.7, 30.5, 26.0, 25.7, 23.0; **HRMS** (ESI) mass calculated for [M+H]+ ($C_{20}H_{26}O_3N$) requires *m/z* 328.1907, found *m/z* 328.1903.

Synthesis of **2d**

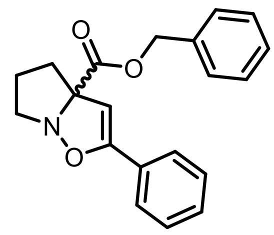

General Procedure A was followed using phenylacetylene (150 µL) as the dipolarophile and the reaction was left for 21 h. The crude oil was purified by FCC (2:1 pentane:EtOAc) to give the desired product as a yellow oil (103 mg, 70%). **IR** (thin film) ν_{max}/cm^{-1} 2980 (C-H), 1734 (C=O), 1382, 1154; **^{1}H NMR** (400 MHz, $CDCl_3$) δ 7.56 – 7.46 (m, 2H), 7.39 – 7.28 (m, 8H), 5.23 (app. d, *J* = 0.8 Hz, 3H), 3.53 – 3.39 (m, 2H), 2.24 (ddd, *J* = 12.7, 8.7, 7.3 Hz, 1H), 2.15 (dddd, *J* = 12.8, 6.9, 4.8, 1.1 Hz, 1H), 1.98 – 1.81 (m, 2H); **^{13}C NMR** (101 MHz, $CDCl_3$) δ 172.8, 154.5, 135.8, 129.3, 128.6, 128.4, 128.2, 128.1, 128.0, 125.8, 94.8, 83.0, 67.0, 60.3, 37.2, 23.3; **HRMS** (ESI) mass calculated for [M+H]+ ($C_{20}H_{20}O_3N$) requires *m/z* 322.1438, found *m/z* 322.1436.

Synthesis of **2e**

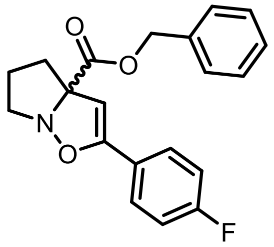

General Procedure A was followed using 4-fluorophenylacetylene (160 mg) and stirred for 20 h. The resulting yellow oil was purified by FCC (2:1 pentane:EtOAc) to give the title compound as a yellow oil (134 mg, 87%). **MP** 54-56 °C; **IR** (thin film) ν_{max}/cm^{-1} 2980 (C-H), 1735 (C=O), 1158; **^{1}H NMR** (400 MHz, $CDCl_3$) δ 7.52 – 7.46 (m, 2H), 7.42 – 7.27 (m, 5H), 7.07 – 6.99 (m, 2H), 5.23 (d, 12.7 Hz, 1H), 5.22 (d, 12.7 Hz, 1H), 5.17 (s, 1H), 3.53 – 3.38 (m, 2H), 2.23 (ddd, *J* = 12.7, 8.5, 7.4 Hz, 1H), 2.17 – 2.09 (m, 1H), 1.97 – 1.83 (m, 2H); **^{13}C NMR** (101 MHz, $CDCl_3$) δ 172.7, 163.3 (d, *J* = 249.2 Hz), 153.6, 135.8, 128.6, 128.2, 128.0, 127.7 (d, *J* = 8.3 Hz), 124.4 (d, *J* = 3.1 Hz), 115.5 (d, *J* = 21.9 Hz), 94.5, 83.1, 67.0, 60.3, 37.1, 23.3; **^{19}F NMR** (376 MHz, $CDCl_3$) δ -111.11 (app. tt, *J* = 8.6, 5.3 Hz); **HRMS** (ESI) mass calculated for [M+H]+ ($C_{20}H_{19}O_3NF$) requires *m/z* 340.1344, found *m/z* 340.1341.

Synthesis of **2f**

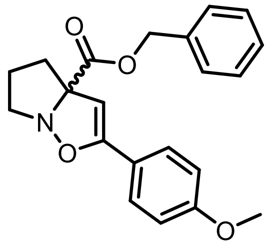

General Procedure A was followed using 4-methoxyphenylacetylene (180 μL) and stirred for 24 h. The resulting crude oil was purified by FCC (3:1 pentane:EtOAc) to give the title compound as a white solid (103 mg, 65%). **MP** 71-72 °C; **IR** (thin film) ν_{max}/cm^{-1} 2976 (C-H), 1725 (C=O), 1708 (C=C), 1258, 1068, 1030 (C-O); **^{1}H NMR** (400 MHz, $CDCl_3$) δ 7.48 – 7.41 (m, 2H), 7.39 – 7.27 (m, 5H), 6.89 – 6.83 (m, 2H), 5.22 (d, 12.6 Hz, 1H), 5.22 (d, 12.6 Hz, 1H), 5.09 (s, 1H), 3.81 (s, 3H), 3.51 – 3.38 (m, 2H), 2.27 – 2.18 (m, 1H), 2.13 (dddd, *J* = 12.6, 6.9, 4.8, 1.0 Hz, 1H), 1.96 – 1.81 (m, 2H); **^{13}C NMR** (101 MHz, $CDCl_3$) δ 172.9, 160.4, 154.3, 135.9, 128.5, 128.2, 128.0, 127.3, 120.8, 113.8, 93.0, 83.0, 66.9, 60.3, 55.3, 37.1, 23.2; **HRMS** (ESI) mass calculated for [M+H]+ ($C_{21}H_{22}O_4N$) requires *m/z* 352.1543, found *m/z* 352.1540

Synthesis of **2g**

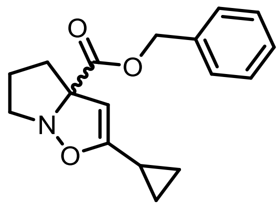

General Procedure A was followed using cyclopropylacetylene (120 μL) and stirred for 19 h. The resulting oil was purified by FCC (4:1 pentane:EtOAc) to give the title compound as a colourless oil (81 mg, 63%). **IR** (thin film) ν_{max}/cm^{-1} 3012 (C-H), 1734 (C=O), 1175; **^{1}H NMR** (400 MHz, $CDCl_3$) δ 7.38 – 7.27 (m, 5H), 5.19 (d, *J* = 1.6 Hz, 2H), 4.51 (s, 1H), 3.33 – 3.26 (m, 2H), 2.13 (ddd, *J* = 12.6, 8.5, 7.3 Hz, 1H), 2.03 – 1.94 (m, 1H), 1.90 – 1.74 (m, 2H), 1.48 – 1.38 (m, 1H), 0.76 – 0.68 (m, 3H), 0.65 (dddd, *J* = 8.8, 5.2, 3.7, 1.6 Hz, 1H); **^{13}C NMR** (101 MHz, $CDCl_3$) δ 173.1, 158.5, 136.0, 128.5, 128.1, 127.9, 92.9, 82.4, 66.7, 60.0, 36.9, 23.1, 7.0, 6.0, 5.7; **HRMS** (ESI) mass calculated for [M+H]+ ($C_{17}H_{20}O_3N$) requires *m/z* 286.1438, found *m/z* 286.1436.

Synthesis of **2h**

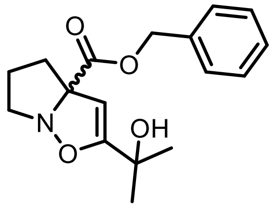

General Procedure A was followed using 2-methyl-3-butyn-2-ol (130 μL) and stirred for 6 h. The resulting oil was purified by FCC (2:1 pentane:EtOAc) to give the title compound as a colourless oil (99 mg, 72%). **IR** (thin film) ν_{max}/cm^{-1} 3399 (O-H), 2977 (C-H), 1733 (C=O), 1227 (C-O), 1172 (C-O); **^{1}H NMR** (400 MHz, $CDCl_3$) δ 7.38 – 7.28 (m, 5H), 5.24 – 5.15 (m, 2H), 4.72 (s, 1H), 3.35 (dd, *J* = 7.1, 5.7 Hz, 2H), 2.22 – 2.11 (m, 1H), 2.02 (m, 2H), 1.90 – 1.77 (m, 2H), 1.40 (s, 6H); **^{13}C NMR** (101 MHz, $CDCl_3$) δ 172.8, 162.1, 135.8, 128.5, 128.2, 127.9, 93.5, 82.4, 68.2, 66.8, 60.1, 36.8, 28.2, 28.1, 23.1; **HRMS** (ESI) mass calculated for [M+H]+ ($C_{17}H_{22}O_4N$) requires *m/z* 304.1543, found *m/z* 304.1541.

Synthesis of **2i**

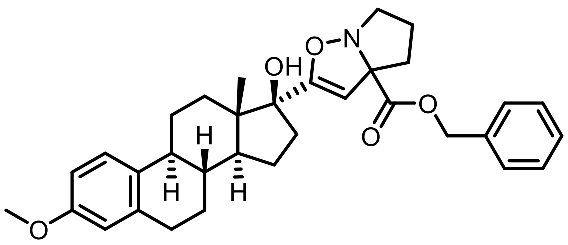

General Procedure A was followed using nitrone **1a** (75 mg, 0.342 mmol) and mestranol (140 mg, 0.445 mmol) and stirred for 24 h. The resulting oil was purified by FCC (3:2 pentane:EtOAc) to give the both diastereomers of the title compound as white solids (127 mg, 70%, 1.2:1 dr).* **Major: MP** 66-68 °C; **IR** (thin film) ν_{max}/cm^{-1} 3509 (O-H), 2934 (C-H), 2866 (C-H), 1733 (C=O), 1254; **^{1}H NMR** (500 MHz, $CDCl_3$) δ 7.34 – 7.28 (m, 2H), 7.23 (dd, *J* = 8.4, 6.8 Hz, 2H), 7.17 – 7.11 (m, 2H), 6.71 (dd, *J* = 8.6, 2.8 Hz, 1H), 6.62 (d, *J* = 2.7 Hz, 1H), 5.22 – 5.13 (m, 2H), 4.68 (s, 1H), 3.78 (s, 3H), 3.44 – 3.33 (m, 2H), 2.89 – 2.73 (m, 2H), 2.28 (ddd, *J* = 12.7, 8.6, 7.1 Hz, 1H), 2.19 – 2.04 (m, 3H), 2.00 – 1.77 (m, 5H), 1.72 – 1.62 (m, 2H), 1.53 (td, *J* = 11.3, 7.3 Hz, 1H), 1.45 – 1.33 (m, 4H), 1.17 (app. qd, *J* = 11.9, 6.9 Hz, 1H), 0.88 (s, 3H); **^{13}C NMR** (126 MHz, $CDCl_3$) δ 172.9, 160.4, 157.4, 137.8, 135.6, 132.6, 128.5, 128.3, 128.2, 126.4, 113.7, 111.4, 97.4, 82.6, 82.4, 67.2, 60.2, 55.2, 48.8, 47.5,

* Investigations into producing a single crystal of either diastereomer were unsuccesful.

43.4, 39.2, 36.3, 33.2, 29.8, 27.4, 26.3, 23.4, 23.0, 14.0; **HRMS** (ESI) mass calculated for [M+H]+ ($C_{33}H_{40}O_5N$) requires *m/z* 530.2901, found *m/z* 530.2901. **Minor: MP** 65-70 °C; **IR** (thin film) ν_{max}/cm^{-1} 3491 (O-H), 2990 (C-H), 1731 (C=O), 1254, 1237; **^{1}H NMR** (500 MHz, $CDCl_3$) δ 7.42 – 7.29 (m, 5H), 7.18 – 7.14 (m, 1H), 6.71 (dd, *J* = 8.6, 2.8 Hz, 1H), 6.62 (d, *J* = 2.8 Hz, 1H), 5.22 (app. d, *J* = 1.7 Hz, 2H), 4.78 (s, 1H), 3.78 (s, 3H), 3.45 (dddd, *J* = 12.3, 6.0, 4.6, 1.1 Hz, 1H), 3.35 (ddd, *J* = 12.6, 9.1, 5.8 Hz, 1H), 2.92 – 2.73 (m, 2H), 2.27 – 2.07 (m, 5H), 1.97 – 1.83 (m, 4H), 1.78 – 1.67 (m, 2H), 1.67 – 1.58 (m, 1H), 1.51 – 1.29 (m, 5H), 0.90 (s, 3H); **^{13}C NMR** (126 MHz, $CDCl_3$) δ 172.7, 161.0, 157.4, 138.0, 135.8, 132.7, 128.6, 128.3, 128.1, 126.3, 113.8, 111.4, 96.8, 82.6, 82.4, 67.0, 60.3, 55.2, 48.8, 47.5, 43.3, 39.4, 36.9, 36.8, 33.0, 29.9, 27.4, 26.3, 23.7, 23.5, 14.1; **HRMS** (ESI) mass calculated for [M+H]+ ($C_{33}H_{40}O_5N$) requires *m/z* 530.2901, found *m/z* 530.2896.

Synthesis of **2p**

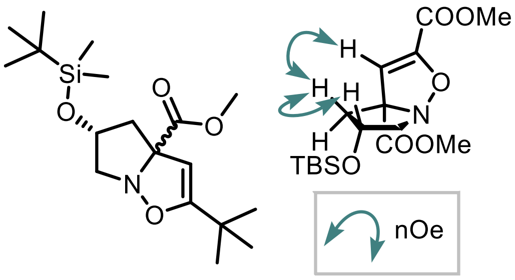

Nitrone **1d** (140 mg, 0.51 mmol) and *t*-Bu-acetylene (390 µL, 3.1 mmol) were stirred in hexane (4 mL) for 2 days at 70 °C. The hexane was removed under a stream of N_2 and the resulting oil was purified by FCC (9:1 pentane:Et_2O) to give the desired product as a colourless oil (134 mg, 74%, 1.7:1 dr). **Major: IR** (thin film) ν_{max}/cm^{-1} 2956 (C-H), 1736 (C=O), 1252, 1118, 836, 776; **^{1}H NMR** (500 MHz, $CDCl_3$) δ 4.50 – 4.35 (m, 2H), 3.75 (s, 3H), 3.39 – 3.21 (m, 2H), 2.25 – 2.19 (m, 2H), 1.11 (s, 9H), 0.86 (s, 9H), 0.05 (s, 3H), 0.04 (s, 3H); **^{13}C NMR** (126 MHz, $CDCl_3$) δ 173.4, 165.0, 92.8, 80.8, 70.1, 66.1, 52.6, 45.3, 31.3, 28.0, 25.7, 18.0, -4.9; **HRMS** (ESI) mass calculated for [M+H]+ ($C_{18}H_{34}O_4NSi$) requires *m/z* 356.2252, found *m/z* 356.2246. **Minor: IR** (thin film) ν_{max}/cm^{-1} 2956 (C-H), 1735 (C=O), 1252, 1109, 836, 777; **^{1}H NMR** (500 MHz, $CDCl_3$) δ 4.58 (s, 1H), 4.32 (qd, *J* = 6.6, 5.2 Hz, 1H), 3.73 (s, 3H),

3.65 – 3.60 (m, 1H), 3.07 (ddd, *J* = 10.6, 6.8, 0.8 Hz, 1H), 2.49 (ddd, *J* = 13.1, 6.6, 0.9 Hz, 1H), 2.05 (ddd, *J* = 13.1, 6.3, 0.9 Hz, 1H), 1.12 (s, 9H), 0.87 (s, 9H), 0.05 (s, 3H), 0.05 (s, 3H); **^{13}C NMR** (126 MHz, $CDCl_3$) δ 173.8, 163.6, 93.7, 80.3, 69.2, 65.5, 52.6, 44.2, 31.0, 28.0, 25.8, 18.1, -4.8, -4.9; **HRMS** (ESI) mass calculated for [M+H]+ ($C_{18}H_{34}O_4NSi$) requires *m/z* 356.2252, found *m/z* 356.2245.

Synthesis of **2q**

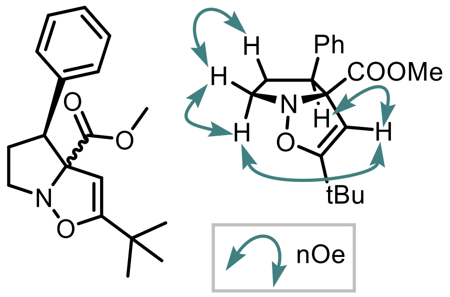

Nitrone **1c** (38 mg, 0.17 mmol) and *t*-Bu-acetylene (130 µL, 1.0 mmol) were stirred in toluene (2 mL) for 4 days at 70 °C. The toluene was removed under a stream of N_2 and the resulting oil was purified by FCC (9:1 pentane:Et_2O) to give the title compound as a colourless oil (22 mg, 42%, 12.5:1 dr). **Major: IR** (thin film) ν_{max}/cm^{-1} 2968 (C-H), 1739 (C=O), 1234, 1118; **^{1}H NMR** (400 MHz, $CDCl_3$) δ 7.31 – 7.25 (m, 2H), 7.23 – 7.20 (m, 1H), 7.20 – 7.15 (m, 2H), 4.78 (s, 1H), 3.88 (dt, *J* = 10.3, 5.1 Hz, 1H), 3.48 (d, *J* = 8.1 Hz, 1H), 3.20 (m, 4H), 2.18 (td, *J* = 7.9, 4.9 Hz, 2H), 1.16 (s, 9H); **^{13}C NMR** (101 MHz, $CDCl_3$) δ 171.9 , 164.0, 139.2, 128.3, 127.9, 127.1, 92.9, 86.1, 58.4, 56.2, 51.5, 31.3, 28.6, 28.1; **HRMS** (ESI) mass calculated for [M+H]+ ($C_{18}H_{24}O_3N$) requires *m/z* 302.1751, found *m/z* 302.1748.*

Synthesis of **2r**

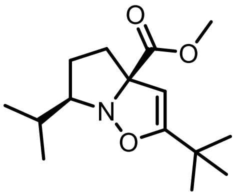

Nitrone **1b** (50 mg, 0.27 mmol) and *t*-Bu-acetylene (200 µL, 1.6 mmol) were stirred in hexane (4 mL) for 2 days at 70 °C. The reaction was determined to be complete by TLC after 2 days and the hexane was

* Minor diastereomer not isolated since only observed in trace quantities

removed under a stream of N_2. Purification by FCC (9:1 pentane:Et_2O) yielded the title compound as a colourless oil (46 mg, 64%, 18.4:1 dr). **Major**[*]**: IR** (thin film) ν_{max}/cm^{-1} 2958 (C-H), 1734 (C=O), 1661, 1244, 1128; **^{1}H NMR** (400 MHz, $CDCl_3$) δ 4.54 (s, 1H), 3.71 (s, 3H), 2.84 (dt, *J* = 11.0, 6.1 Hz, 1H), 2.36 (ddd, *J* = 12.8, 7.9, 2.4 Hz, 1H), 2.00 – 1.83 (m, 2H), 1.74 (dddd, *J* = 12.6, 8.1, 5.9, 2.4 Hz, 1H), 1.53 (dtd, *J* = 12.6, 10.9, 7.9 Hz, 1H), 1.11 (s, 9H), 1.04 (d, *J* = 6.8 Hz, 3H), 0.94 (d, *J* = 6.8 Hz, 3H); **^{13}C NMR** (101 MHz, $CDCl_3$) δ 174.6, 163.7, 93.5, 80.7, 76.5, 52.4, 35.3, 31.1, 31.1, 28.1, 24.2, 20.5, 19.0; **HRMS** (ESI) mass calculated for [M+H]+ ($C_{15}H_{26}O_3N$) requires *m/z* 268.1907, found *m/z* 268.1907.[†]

7. Synthesis and Characterisation of Ketophosphonates

Synthesis of **3a**

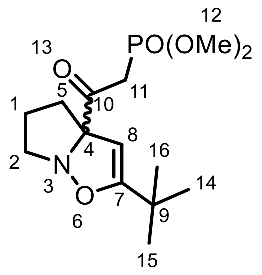

General Procedure B was followed using benzyl ester **2a** (50 mg, 0.17 mmol). FCC afforded the title compound as a colourless oil (45 mg, 84%). **IR** (thin film) ν_{max}/cm^{-1} 2968 (C-H), 1715 (C=O), 1259 (P=O), 1031 (P-O antisymmetric); **^{1}H NMR** (400 MHz, $CDCl_3$) δ 4.27 (s, 1H, 8), 3.79 (d, *J* = 11.2 Hz, 3H, 12), 3.77 (d, *J* = 11.2 Hz, 3H, 12'), 3.62 (dd, *J* = 21.4, 14.8 Hz, 1H, 11), 3.33 – 3.19 (m, 3H, 2', 2, 11'), 2.32 – 2.22 (m, 1H, 5), 1.92 – 1.83 (m, 1H, 5'), 1.83 – 1.66 (m, 2H, 1), 1.12 (s, 9H, 14-16); **^{13}C NMR** (101 MHz, $CDCl_3$) δ 202.3 (d, *J* = 6.6 Hz, 10), 166.4 (7), 92.26 (8), 88.1 (d, *J* = 3.9 Hz, 4), 59.7 (2), 52.9 (d, *J* = 6.3 Hz, 12), 52.8 (d, *J* = 6.3 Hz, 12'), 35.6 (d, *J* = 135.0 Hz, 11), 33.6 (5), 31.4 (9), 28.1 (14-16), 23.0 (1); **^{31}P NMR** (162 MHz, $CDCl_3$) δ 24.20; **HRMS** (ESI) mass calculated for [M+H]+ ($C_{14}H_{25}O_5NP$) requires *m/z* 318.1465, found *m/z* 318.1462.

Synthesis of **3b**

[*] NOESY analysis was inconclusive, however stereochemistry was assigned based on the more decisive NOESY correlations of **3r**.
[†] Minor diastereomer not isolated since only observed in trace quantities.

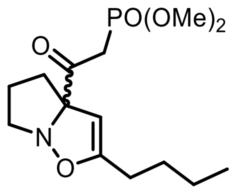

General Procedure B was followed using cycloadduct **2b** (41 mg, 0.136 mmol). Purification by FCC yielded the title compound as a yellow oil (26 mg, 61%). **IR** (thin film) ν_{max}/cm^{-1} 2957 (C-H), 1714 (C=O), 1249 (P=O), 1031 (P-O antisymmetric); **^{1}H NMR** (400 MHz, $CDCl_3$) δ 4.32 (app. d, *J* = 1.1 Hz, 1H), 3.79 (d, *J* = 11.2 Hz, 3H), 3.77 (d, *J* = 11.2 Hz, 3H), 3.65 (dd, *J* = 21.4, 14.9 Hz, 1H), 3.28-3.26 (m, 3H), 2.31 – 2.21 (m, 1H), 2.17 – 2.08 (m, 2H), 1.94 – 1.79 (m, 2H), 1.78 – 1.69 (m, 1H), 1.54 – 1.43 (m, 2H), 1.41 – 1.28 (m, 2H), 0.90 (t, *J* = 7.3 Hz, 3H); **^{13}C NMR** (101 MHz, $CDCl_3$) δ 202.4 (d, *J* = 6.8 Hz), 158.6, 94.5, 88.3 (d, *J* = 3.2 Hz), 59.9, 53.0 (d, *J* = 6.3 Hz), 52.8 (d, *J* = 6.2 Hz), 35.8 (d, *J* = 135.0 Hz), 33.8, 28.8, 25.5, 23.2, 22.2, 13.7; **^{31}P NMR** (162 MHz, $CDCl_3$) δ 24.14; **HRMS** (ESI) mass calculated for [M+H]+ ($C_{14}H_{25}O_5NP$) requires *m/z* 318.1465, found *m/z* 318.1468

Synthesis of **3c**

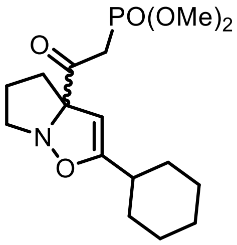

General Procedure B was followed using cycloadduct **2c** (50 mg, 0.153 mmol). Purification by FCC afforded the title compound as a yellow oil (35 mg, 67%). **IR** (thin film) ν_{max}/cm^{-1} 2929 (C-H), 1714 (C=O), 1258 (P=O), 1030 (P-O antisymmetric); **^{1}H NMR** (400 MHz, $CDCl_3$) δ 4.27 (d, *J* = 1.0 Hz, 1H), 3.79 (d, *J* = 11.2 Hz, 3H), 3.77 (d, *J* = 11.2 Hz, 3H), 3.62 (dd, *J* = 21.4, 14.9 Hz, 1H), 3.32 – 3.18 (m, 3H), 2.31 – 2.21 (m, 1H), 2.16 – 2.07 (m, 1H), 1.91 – 1.78 (m, 4H), 1.73 (m, 3H), 1.65 (m, *J* = 11.2, 6.4, 2.5 Hz, 1H), 1.33 – 1.13 (m, 5H); **^{13}C NMR** (101 MHz, $CDCl_3$) δ 202.4 (d, *J* = 6.8 Hz), 163.0, 92.8, 88.1 (d, *J* = 3.2 Hz), 59.8, 53.0 (d, *J* = 6.3 Hz), 52.8 (d, *J* = 6.4 Hz), 35.6 (d, *J* = 134.9 Hz), 35.3, 33.6, 30.7, 30.6, 25.9, 25.7, 23.0; **^{31}P NMR** (162 MHz, $CDCl_3$) δ 24.19; **HRMS** (ESI) mass calculated for [M+H]+ ($C_{16}H_{27}O_5NP$) requires *m/z* 344.1621, found *m/z* 344.1624.

Synthesis of **3d**

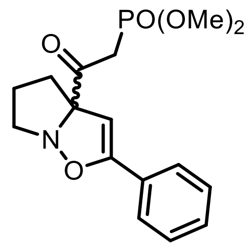

General Procedure B was followed using cycloadduct **2d** (50 mg, 0.16 mmol). FCC afforded the title compound as a yellow oil (37 mg, 70%). **IR** (thin film) ν_{max}/cm^{-1} 3058 (C-H), 1715 (C=O), 1260 (P=O), 1029 (P-O antisymmetric); **^{1}H NMR** (400 MHz, $CDCl_3$) δ 7.51 (dddd, *J* = 6.7, 4.0, 2.6, 1.5 Hz, 2H), 7.38 – 7.33 (m, 3H), 5.06 (s, 1H), 3.78 (d, *J* = 11.2 Hz, 3H), 3.76 (d, *J* = 11.2 Hz, 3H), 3.71 (dd, *J* = 21.6, 14.7 Hz, 1H), 3.45 – 3.39 (m, 2H), 3.34 (dd, *J* = 21.2, 14.7 Hz, 1H), 2.36 (ddd, *J* = 12.9, 8.4, 7.0 Hz, 1H), 2.04 (dddd, *J* = 12.8, 7.0, 4.9, 0.9 Hz, 1H), 1.97 – 1.85 (m, 1H), 1.81 (app. ddq, *J* = 12.5, 7.1, 5.4 Hz, 1H); **^{13}C NMR** (101 MHz, $CDCl_3$) δ 202.1 (d, *J* = 6.8 Hz), 155.3, 129.6, 128.5, 127.7, 125.8, 94.9, 88.9 (d, *J* = 3.2 Hz), 60.1, 52.9 (dd, *J* = 12.6, 6.4 Hz), 36.01 (d, *J* = 134.2 Hz), 34.0, 23.3; **^{31}P NMR** (162 MHz, $CDCl_3$) δ 23.79; **HRMS** (ESI) mass calculated for [M+H]+ ($C_{16}H_{21}O_5NP$) requires *m/z* 338.1152, found *m/z* 338.1148.

Synthesis of **3e**

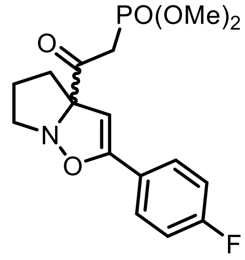

General Procedure B was followed using cycloadduct **2e** (50 mg, 0.147 mmol). Purification by FCC afforded the title compound as a yellow oil (39 mg, 75%). **IR** (thin film) ν_{max}/cm^{-1} 2981 (C-H), 1734 (C=O), 1259 (P-O); **^{1}H NMR** (400 MHz, $CDCl_3$) δ 7.51 – 7.45 (m, 2H), 7.07 – 6.99 (m, 2H), 5.00 (s, 1H), 3.77 (d, *J* = 11.2 Hz, 3H), 3.75 (d, *J* = 11.2 Hz, 3H), 3.70 (dd, *J* = 21.7, 14.7 Hz, 1H), 3.41 (dd, *J* = 7.7, 5.7 Hz, 2H), 3.32 (dd, *J* = 21.3, 14.7 Hz, 1H), 2.35 (ddd, *J* = 12.9, 8.5, 7.1 Hz, 1H), 2.08 – 1.97 (m, 1H), 1.96 – 1.73 (m, 2H); **^{13}C NMR** (101 MHz, $CDCl_3$) δ 202.0 (d, *J* = 6.5 Hz), 163.3 (d, *J* = 249.7 Hz), 154.3, 127.8 (d, *J* = 8.2 Hz), 124.0 (d, *J* = 3.2 Hz), 115.6 (d, *J* = 22.2 Hz), 94.7, 89.0 (d, *J* = 3.2 Hz), 60.1, 53.0 (d, *J* = 6.3 Hz), 52.9 (d, *J* = 6.3 Hz), 35.9 (d, *J* = 134.2 Hz), 34.0, 23.3; **^{19}F NMR** (377 MHz, $CDCl_3$) δ -

110.44 (app. tt, J = 8.5, 5.3 Hz); ^{31}**P NMR** (162 MHz, $CDCl_3$) δ 23.69; **HRMS** (ESI) mass calculated for [M+H]+ ($C_{16}H_{20}O_5NFP$) requires m/z 356.1058, found m/z 356.1059.

Synthesis of **3f**

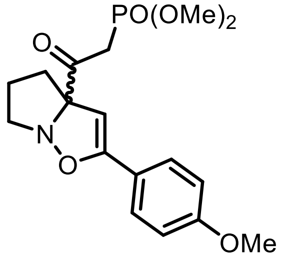

General Procedure B was followed using cycloadduct **2f** (50 mg, 0.141 mmol). Purification by FCC afforded the title compound as a yellow oil (37 mg, 72%). **IR** (thin film) ν_{max}/cm^{-1} 2956 (C-H), 1713 (C=O), 1647, 1251 (P-O), 1030 (P=O antisymmetric); ^{1}**H NMR** (400 MHz, $CDCl_3$) δ 7.46 – 7.41 (m, 2H), 6.89 – 6.84 (m, 2H), 4.91 (s, 1H), 3.81 (s, 3H), 3.77 (d, J = 11.2 Hz, 3H), 3.76 (d, J = 11.2 Hz, 3H), 3.71 – 3.64 (dd, 1H), 3.40 (m, 2H), 3.33 (dd, J = 21.2, 14.7 Hz, 1H), 2.35 (ddd, J = 12.8, 8.4, 7.0 Hz, 1H), 2.02 (dddd, J = 12.7, 7.0, 5.0, 0.8 Hz, 1H), 1.96 – 1.72 (m, 2H); ^{13}**C NMR** (101 MHz, $CDCl_3$) δ 202.2 (d, J = 6.4 Hz), 160.6, 155.1, 127.3, 120.3, 113.9, 93.1, 88.9 (d, J = 3.3 Hz), 60.0, 55.35, 53.0 (d, J = 6.3 Hz), 52.9 (d, J = 5.9 Hz), 35.9 (d, J = 134.3 Hz), 33.9, 23.2; ^{31}**P NMR** (162 MHz, $CDCl_3$) δ 23.95; **HRMS** (ESI) mass calculated for [M+H]+ ($C_{17}H_{23}O_6NP$) requires m/z 368.1258, found m/z 368.1256.

Synthesis of **3g**

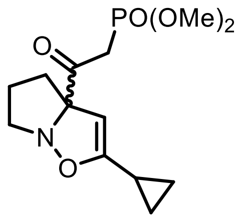

General Procedure B was followed using cycloadduct **2g** (35 mg, 0.123 mmol). Purification by FCC afforded the title compound as a yellow oil (27 mg, 74%). **IR** (thin film) ν_{max}/cm^{-1} 2980 (C-H), 1713 (C=O), 1254 (P=O), 1030 (P-O antisymmetric); ^{1}**H NMR** (400 MHz, $CDCl_3$) δ 4.32 (s, 1H), 3.79 (d, J = 11.2 Hz, 3H), 3.77 (d, J = 11.2 Hz, 3H), 3.61 (dd, J = 21.4, 14.9 Hz, 1H), 3.34 – 3.17 (m, 3H), 2.30 – 2.19 (m, 1H), 1.93 – 1.78 (m, 2H), 1.78 – 1.67 (m, 1H), 1.49 – 1.39 (m, 1H), 0.80 – 0.63 (m, 4H); ^{13}**C NMR** (101 MHz, $CDCl_3$) δ 202.2 (d, J = 6.3 Hz), 159.6, 92.9, 88.3 (d, J = 3.7 Hz), 59.7, 53.0 (d, J = 6.2 Hz), 52.8 (d, J = 6.1 Hz), 35.7 (d, J =

135.0 Hz), 33.6, 23.1, 7.0, 6.2, 5.9; **^{31}P NMR** (162 MHz, $CDCl_3$) δ 24.16; **HRMS** (ESI) mass calculated for [M+H]+ ($C_{13}H_{21}O_5NP$) requires *m/z* 302.1152, found *m/z* 302.1155.

Synthesis of **3h**

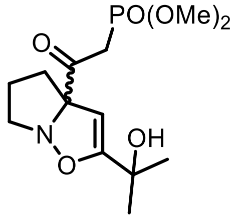

General Procedure B was followed using cycloadduct **2h** (50 mg, 0.166 mmol), and modified by the use of additional $MePO(OMe)_2$ (41 μL, 0.38 mmol) and LiHMDS (680 μL 1M in THF, 0.68 mmol). Purification by FCC afforded the title compound as a colourless oil (36 mg, 68%). **IR** (thin film) ν_{max}/cm^{-1} 3381 (O-H), 2977 (C-H), 1713 (C=O), 1243 (P=O), 1025 (P-O antisymmetric); **^{1}H NMR** (400 MHz, $CDCl_3$) δ 4.54 (d, *J* = 0.8 Hz, 1H), 3.80 – 3.77 (m, 3H), 3.77 – 3.74 (m, 3H), 3.59 – 3.48 (m, 1H), 3.43 – 3.22 (m, 3H), 3.13 (s, 1H), 2.30 (dtd, *J* = 11.6, 6.9, 6.3, 1.4 Hz, 1H), 1.97 – 1.79 (m, 2H), 1.79 – 1.65 (m, 1H), 1.41 (d, *J* = 1.8 Hz, 3H), 1.41 (d, *J* = 1.6 Hz, 3H); **^{13}C NMR** (101 MHz, $CDCl_3$) δ 201.2 (d, *J* = 6.7 Hz), 163.6, 93.6, 88.0 (d, *J* = 2.9 Hz), 67.8, 59.6, 53.2 (d, *J* = 6.4 Hz), 52.9 (d, *J* = 6.4 Hz), 35.9 (d, *J* = 132.7 Hz), 32.8, 28.3, 27.7, 22.9; **^{31}P NMR** (162 MHz, $CDCl_3$) δ 23.52; **HRMS** (ESI) mass calculated for [M+H]+ ($C_{13}H_{23}O_6NP$) requires *m/z* 320.1258, found *m/z* 320.1261.

Synthesis of **3i**

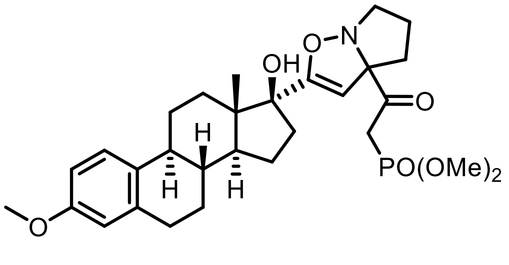

General Procedure B was followed using cycloadduct (Major) **2i** (50 mg, 0.095 mmol), and modified by the use of additional $MePO(OMe)_2$ (24 μL, 0.219 mmol) and LiHMDS (390 μL, 1M in THF, 0.390 mmol). Purification by FCC afforded the title compound as an amorphous white solid (33 mg, 64%). **IR** (thin film) ν_{max}/cm^{-1} 3390 (O-H), 2932 (C-H), 1714 (C=O), 1254 (P=O), 1032 (P-O antisymmetric); **^{1}H NMR** (400 MHz, $CDCl_3$) δ 7.21 – 7.16 (m, 1H), 6.70 (dd, *J* = 8.6, 2.8 Hz, 1H), 6.62 (d, *J* = 2.8 Hz, 1H), 4.61 (s, 1H), 3.77 (s, 3H), 3.77 (d, *J* = 11.2

Hz, 3H), 3.73 (d, *J* = 11.2 Hz, 3H), 3.55 (dd, *J* = 21.0, 14.2 Hz, 1H), 3.49 – 3.39 (m, 1H), 3.38 – 3.30 (m, 2H), 3.13 (br s, 1H), 2.92 – 2.78 (m, 2H), 2.39 – 2.29 (m, 2H), 2.22 (ddd, *J* = 14.2, 9.4, 5.9 Hz, 1H), 2.13 (td, *J* = 10.9, 4.2 Hz, 1H), 2.01 – 1.85 (m, 4H), 1.83 – 1.69 (m, 3H), 1.69 – 1.60 (m, 1H), 1.57 – 1.42 (m, 4H), 1.41 – 1.24 (m, 1H), 0.94 (s, 3H); **^{13}C NMR** (101 MHz, $CDCl_3$) δ 200.6 (d, *J* = 7.0 Hz), 162.7, 157.5, 138.0, 132.4, 126.2, 113.8, 111.5, 97.1, 88.2 (d, *J* = 2.4 Hz), 82.4, 60.0, 55.2, 53.3 (d, *J* = 6.4 Hz), 52.9 (d, *J* = 6.4 Hz), 49.1, 47.3, 43.9, 39.4, 36.4, 36.3 (d, *J* = 132.5 Hz), 33.7, 32.6, 29.8, 27.5, 26.4, 23.5, 23.4, 14.0; **^{31}P NMR** (162 MHz, $CDCl_3$) δ 23.38; **HRMS** (ESI) mass calculated for [M+H]+ ($C_{29}H_{41}O_7NP$) requires *m/z* 546.2615, found *m/z* 546.2612

Synthesis of **3j**

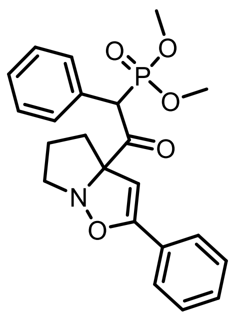

The modification to General Procedure B was followed using cycloadduct **2d** (50 mg, 0.156 mmol) and phosphonate **S11** (65 mg, 0.327 mmol). Purification yielded the title compound as a white solid (45 mg, 69%). The product was isolated as a 1.3:1 mixture of diastereomers. **MP** 135-138 °C; **IR** (thin film) ν_{max}/cm^{-1} 2957 (C-H), 1712 (C=O), 1255 (P=O), 1027 (P=O antisymmetric); **^{1}H NMR** (500 MHz, $CDCl_3$) δ 7.55 – 7.47 (m, $2H_{maj}+2H_{min}$), 7.44 – 7.40 (m, $2H_{maj}$), 7.38 – 7.27 (m, $4H_{maj}+6H_{min}$), 7.24 – 7.18 (m, $2H_{maj}+2H_{min}$), 5.64 (d, *J* = 21.6 Hz, $1H_{maj}$), 5.49 (d, *J* = 19.2 Hz, $1H_{min}$), 5.18 (s, $1H_{min}$), 4.62 (s, $1H_{maj}$), 3.72 (d, *J* = 10.9 Hz, $3H_{maj}$), 3.69 (d, *J* = 11.0 Hz, $3H_{maj}$), 3.67 (d, *J* = 10.8 Hz, $3H_{min}$), 3.62 (d, *J* = 11.0 Hz, $3H_{min}$), 3.47 (dd, *J* = 7.3, 5.5 Hz, $2H_{maj}$), 3.29 (dt, *J* = 11.9, 5.8 Hz, $1H_{min}$), 2.89 (ddd, *J* = 12.4, 8.6, 5.6 Hz, $1H_{min}$), 2.53 – 2.44 (m, $1H_{maj}$), 2.07 - 1.71 (m, $3H_{maj}+3H_{min}$), 1.46 – 1.38 (m, $1H_{min}$); **^{13}C NMR** (126 MHz, $CDCl_3$) δ 203.5 (d, *J* = 5.1 Hz)$_{min}$, 202.6 (d, *J* = 4.8 Hz)$_{maj}$, 155.3$_{maj}$, 154.9$_{min}$, 131.6 (d, *J* = 9.4 Hz)$_{maj}$, 130.7 (d, *J* = 9.0 Hz)$_{min}$, 130.1 (d, *J* = 6.4 Hz)$_{min}$, 129.7 (d, *J* = 6.7 Hz)$_{maj}$, 129.5$_{min}$, 129.5$_{maj}$, 128.6$_{min}$, 128.5$_{maj}$,

128.3$_{min}$, 127.9$_{min}$, 127.8 (d, J = 3.3 Hz)$_{min}$, 127.7$_{maj}$, 127.6 (d, J = 3.1 Hz)$_{maj}$, 125.8$_{maj+min}$, 95.4$_{maj}$, 95.3$_{min}$, 89.5 (d, J = 4.8 Hz)$_{min}$, 89.2 (d, J = 4.3 Hz)$_{maj}$, 60.1$_{maj}$, 59.9$_{min}$, 53.8 (d, J = 6.4 Hz)$_{min}$, 53.7 (d, J = 6.6 Hz)$_{min}$, 53.4 (d, J = 7.0 Hz)$_{maj}$, 53.4 (d, J = 7.0 Hz)$_{maj}$, 52.30 (d, J = 134.7 Hz)$_{min}$, 52.30 (d, J = 134.7 Hz)$_{maj}$, 36.0$_{min}$, 33.9$_{maj}$, 23.4$_{maj}$, 23.1$_{min}$; **^{31}P NMR** (162 MHz, $CDCl_3$) δ 22.46$_{min}$, 22.21$_{maj}$; **HRMS** (ESI) mass calculated for [M+H]+ ($C_{22}H_{25}O_5NP$) requires *m/z* 414.1465, found *m/z* 414.1458.

Synthesis of **3k**

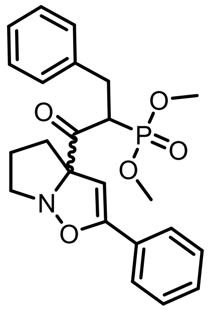

The modification to General Procedure B was followed using cycloadduct **2d** (50 mg, 0.156 mmol) and phosphonate **S12** (70 mg, 0.327 mmol). Purification yielded the title compound as a yellow oil (41 mg, 61%). The product was isolated as a 2.3:1 mixture of diastereomers. **IR** (thin film) ν_{max}/cm^{-1} 2956 (C-H), 1708 (C=O), 1251 (P=O), 1028 (P-O antisymmetric); **^{1}H NMR** (500 MHz, $CDCl_3$) δ 7.48 – 7.44 (m, 2H$_{min}$), 7.35 – 7.27 (m, 3H$_{maj}$+3H$_{min}$), 7.25 – 7.14 (m, 2H$_{maj}$), 7.06 – 7.00 (m, 2H$_{maj}$+2H$_{min}$), 6.94 – 6.88 (m, 3H$_{maj}$+3H$_{min}$), 5.08 (s, 1H$_{min}$), 4.73 (ddd, J = 23.6, 12.0, 3.4 Hz, 1H$_{maj}$), 4.67 (ddd, J = 21.6, 9.0, 6.5 Hz, 1H$_{min}$), 4.01 (s, 1H$_{maj}$), 3.84 (d, J = 11.0 Hz, 6H$_{maj}$), 3.69 (d, J = 11.0 Hz, 3H$_{min}$), 3.68 (d, J = 11.0 Hz, 3H$_{min}$), 3.46 – 3.34 (m, 2H$_{maj}$), 3.30 – 3.20 (m, 1H$_{maj}$+1H$_{min}$), 3.10 (ddd, J = 13.3, 9.1, 3.4 Hz, 1H$_{maj}$), 2.69 (ddd, J = 12.7, 9.4, 5.2 Hz, 1H$_{min}$), 2.30 – 2.23 (m, 1H$_{maj}$+1H$_{min}$), 1.99 (dddd, J = 12.7, 6.3, 5.0, 1.0 Hz, 1H$_{maj}$), 1.85 – 1.72 (m, 2H$_{maj}$), 1.70 – 1.54 (m, 2H$_{min}$), 1.29 – 1.18 (m, 2H$_{min}$); **^{13}C NMR** (126 MHz, $CDCl_3$) δ 206.6 (d, J = 6.1 Hz)$_{min}$, 204.3 (d, J = 5.1 Hz)$_{maj}$, 154.7$_{maj}$, 154.1$_{min}$, 138.9 (d, J = 16.7 Hz)$_{min}$, 138.6 (d, J = 16.8 Hz)$_{maj}$, 129.3$_{maj}$, 129.3$_{min}$, 128.9$_{maj+min}$, 128.5 - 128.4 (app. m)$_{min}$, 128.2$_{maj}$, 128.0$_{maj}$, 126.7$_{min}$, 126.3$_{maj}$, 125.9$_{maj}$, 125.6$_{min}$, 95.7$_{min}$, 93.6$_{maj}$, 89.9 – 89.7 (m)$_{maj}$, 89.1 (d, J = 2.1 Hz)$_{min}$, 60.1$_{min}$, 59.7$_{maj}$, 53.5 (d, J = 6.6 Hz)$_{maj}$, 53.1 (d, J = 6.8, 2.1 Hz)$_{min}$, 48.5 (d, J = 124.1

Hz)$_{maj}$, 47.97 (d, *J* = 127.1 Hz)$_{min}$, 34.8$_{maj}$, 34.6$_{min}$, 34.0 (d, *J* = 4.7 Hz)$_{min}$, 33.6 (d, *J* = 4.9 Hz)$_{maj}$, 23.3$_{maj}$, 22.8$_{min}$; **^{31}P NMR** (162 MHz, $CDCl_3$) δ 24.56$_{maj}$, 24.24$_{min}$; **HRMS** (ESI) mass calculated for [M+H]+ ($C_{23}H_{27}O_5NP$) requires *m/z* 428.1621, found *m/z* 428.1617.

Synthesis of **3l**

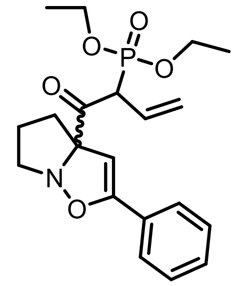

The modification to General Procedure B was followed using cycloadduct **2d** (50 mg, 0.156 mmol) and diethyl allylphosphonate (44 mg, 0.296 mmol). Purification yielded the title compound as a light yellow oil (30 mg, 49%). The product was isolated as a 2:1 mixture of diastereomers. **IR** (thin film) ν_{max}/cm^{-1} 2980, 1642, 1611, 1563, 1243 (P=O), 1020 (P-O antisymmetric); **^{1}H NMR** (400 MHz, $CDCl_3$) δ 7.56 – 7.50 (m, 2H$_{min}$+2H$_{min}$), 7.50 – 7.44 (m, 2H$_{maj}$), 7.34 (qd, *J* = 4.0, 2.0 Hz, 3H$_{maj}$+3H$_{min}$), 5.97 (app. dtdd, *J* = 16.8, 9.7, 6.4, 3.5 Hz, 1H$_{maj}$+1H$_{min}$), 5.38 – 5.25 (m, 2H$_{min}$), 5.21 (s, 1H$_{min}$), 5.20 – 5.11 (m, 2H$_{maj}$), 4.93 (s, 1H$_{maj}$), 4.86 (dd, *J* = 20.8, 9.1 Hz, 1H$_{min}$), 4.23 – 4.10 (m, 4H$_{maj}$+2H$_{min}$), 4.10 – 4.03 (m, 2H$_{min}$), 3.54 – 3.41 (m, 2H$_{maj}$+1H$_{min}$), 3.29 (ddd, *J* = 12.5, 8.7, 5.6 Hz, 1H$_{min}$), 2.38 (ddd, *J* = 12.8, 9.0, 7.0 Hz, 1H$_{maj}$), 2.31 (ddd, *J* = 12.9, 8.7, 7.0 Hz, 1H$_{min}$), 2.10 (dddd, *J* = 12.6, 6.8, 4.4, 1.0 Hz, 1H$_{maj}$), 2.01 (dddd, *J* = 14.2, 7.0, 4.1, 1.9 Hz), 1.96 – 1.80 (m, 2H$_{maj}$+1H$_{min}$), 1.76 (ddq, *J* = 12.3, 7.0, 5.0 Hz, 1H$_{min}$), 1.32 (q, *J* = 7.0 Hz, 6H$_{maj}$), 1.24 (dt, *J* = 13.0, 7.1 Hz, 6H$_{min}$). **^{13}C NMR** (126 MHz, $CDCl_3$) δ 203.8 (d, *J* = 5.8 Hz)$_{maj}$, 202.7 (d, *J* = 6.4 Hz)$_{min}$, 155.2$_{maj}$, 155.0$_{min}$, 129.8 (d, *J* = 11.9 Hz)$_{maj}$, 129.5$_{maj}$, 129.5$_{min}$, 129.3 (d, *J* = 11.7 Hz)$_{min}$, 128.5$_{maj+min}$, 128.0$_{min}$, 127.8$_{maj}$, 125.8$_{maj+min}$, 120.2 (d, *J* = 12.5 Hz)$_{min}$, 119.6 (d, *J* = 12.8 Hz)$_{maj}$, 95.5$_{min}$, 95.1$_{maj}$, 89.4 (d, *J* = 2.3 Hz)$_{maj}$, 89.1 (d, *J* = 2.8 Hz)$_{min}$, 62.93 (app dt, *J* = 29.7, 6.5 Hz)$_{maj+min}$, 60.2$_{min}$, 60.0$_{maj}$, 52.1 (d, *J* = 132.1 Hz)$_{min}$, 52.0 (d, *J* = 128.8 Hz)$_{maj}$, 34.8$_{maj}$, 34.7$_{min}$, 23.4$_{maj}$, 23.4$_{min}$, 16.5 – 16.3 (m)$_{maj+min}$. **^{31}P NMR** (162 MHz, $CDCl_3$) δ 19.52$_{min}$, 19.36$_{maj}$.

Synthesis of **3m**

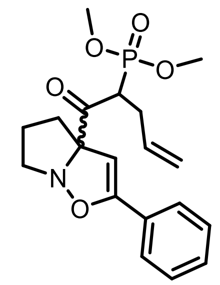

The modification to General Procedure B was followed using cycloadduct **2d** (40 mg, 0.125 mmol) and phosphonate **S13** (43 mg, 0.262 mmol). Purification yielded the title compound as a yellow oil (25 mg, 53%). The product was isolated as a 2:1 mixture of diastereomers. **IR** (thin film) ν_{max}/cm^{-1} 2953 (C-H), 1708 (C=O), 1250 (P=O), 1026 (P=O antisymmetric) **^{1}H NMR** (500 MHz, $CDCl_3$) δ 7.54 – 7.51 (m, $2H_{min}$), 7.49 – 7.46 (m, $2H_{maj}$), 7.35 (dp, *J* = 4.6, 1.5 Hz, $3H_{maj}$+$3H_{min}$), 5.79 – 5.70 (m, $1H_{min}$), 5.55 (dddd, *J* = 16.4, 10.1, 7.6, 6.1 Hz, $1H_{maj}$), 5.19 (s, $1H_{min}$), 5.06 (dd, *J* = 17.0, 1.5 Hz, $1H_{min}$), 5.00 (dd, *J* = 17.0, 1.0 Hz, $1H_{maj}$+$1H_{min}$), 4.90-4.86 (m, $2H_{maj}$), 4.52 (ddd, *J* = 23.4, 10.9, 3.9 Hz, $1H_{maj}$), 4.31 (ddd, *J* = 20.8, 10.3, 4.4 Hz, $1H_{min}$), 3.80 (d, *J* = 11.0 Hz, $3H_{maj}$), 3.80 (d, *J* = 10.9 Hz, $3H_{maj}$), 3.70 (d, *J* = 11.2 Hz, $3H_{min}$), 3.67 (d, *J* = 11.0 Hz, $3H_{min}$), 3.56 – 3.42 (m, $2H_{maj}$+$1H_{min}$), 3.24 (ddd, *J* = 12.6, 9.1, 5.6 Hz, $1H_{min}$), 2.79 – 2.60 (m, $1H_{maj}$+$2H_{min}$), 2.58 – 2.48 (m, $1H_{maj}$), 2.33 (ddd, *J* = 12.8, 8.9, 7.2 Hz, $1H_{maj}$), 2.22 (ddd, *J* = 12.9, 9.1, 6.9 Hz, $1H_{min}$), 2.15 (dddd, *J* = 12.8, 6.8, 4.6, 1.1 Hz, $1H_{maj}$), 2.00 (dddd, *J* = 12.8, 7.0, 4.3, 1.3 Hz, $1H_{min}$), 1.95 – 1.79 (m, $2H_{maj}$+$1H_{min}$), 1.79 – 1.70 (m, $1H_{min}$); **^{13}C NMR** (126 MHz, $CDCl_3$) δ 205.6 (d, *J* = 4.9 Hz)$_{maj}$, 205.4 (d, *J* = 5.7 Hz)$_{min}$, 155.0_{maj}, 154.7_{min}, 135.2 (d, *J* = 15.3 Hz)$_{min}$, 134.9 (d, *J* = 15.9 Hz)$_{maj}$, 129.5_{maj}, 129.4_{min}, $128.5_{maj+min}$, 128.0_{min}, 127.8_{maj}, 125.8_{maj}, 125.7_{min}, 117.2_{min}, 117.0_{maj}, 95.8_{min}, 94.9_{maj}, 89.6_{maj}, 89.1 (d, *J* = 1.9 Hz)$_{min}$, 60.3_{min}, 59.9_{maj}, 53.4 (d, *J* = 6.3 Hz)$_{maj+min}$, 53.1 (d, *J* = 6.6 Hz)$_{maj+min}$, 45.9 (d, *J* = 126.0 Hz)$_{maj+min}$, 35.1_{maj}, 34.5_{min}, 32.7 (d, *J* = 4.9 Hz)$_{min}$, 31.9 (d, *J* = 5.1 Hz)$_{maj}$, 23.4_{min}, 23.3_{maj}; **^{31}P NMR** (162 MHz, $CDCl_3$) δ 24.91_{maj}, 24.90_{min}; **HRMS** (ESI) mass calculated for [M+H]+ ($C_{19}H_{25}O_5NP$) requires *m/z* 378.1465, found *m/z* 378.1463.

Synthesis of **3n**

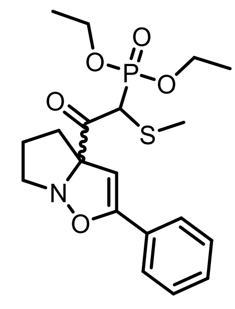

The modification to General Procedure B was followed using cycloadduct **2d** (68 mg, 0.212 mmol) and diethyl methylthiomethylphosphonate (78

μL, 0.445 mmol). Purification yielded the title compound as a light yellow oil (47 mg, 54%). The product was isolated as a 1:1 mixture of diastereomers. **IR** (thin film) ν_{max}/cm^{-1} 2981 (C-H), 1706 (C=O), 1252 (P=O), 1023 (P-O antisymmetric) **^{1}H NMR** (500 MHz, $CDCl_3$) δ 7.45-7.41 (m, $2H_{maj}+2H_{min}$), 7.30-7.27 (m, $3H_{maj}+3H_{min}$), 5.12 (s, $1H_{maj}$), 5.11 (s, $1H_{min}$), 4.86 (d, *J* = 18.9 Hz, $1H_{min}$), 4.66 (d, *J* = 15.2 Hz, $1H_{maj}$), 4.29 – 4.08 (m, $4H_{maj}+4H_{min}$), 3.52 – 3.41 (m, $2H_{maj}+2H_{min}$), 2.38 (ddd, *J* = 12.9, 8.9, 7.0 Hz, $1H_{min}$), 2.30 (m, $4H_{maj}$), 2.21 – 2.09 (m, $1H_{maj}+2H_{min}$), 1.97 – 1.80 (m, $2H_{maj}+2H_{min}$), 1.38 – 1.32 (m, $6H_{min}$), 1.26 (app. dt, *J* = 15.4, 7.1 Hz, $6H_{maj}$); **^{13}C NMR** (126 MHz, $CDCl_3$) δ 202.5_{min}, 199.9_{maj}, 155.1_{min}, 154.9_{maj}, 129.6_{min}, 129.5_{maj}, 128.5_{min}, 128.5_{maj}, 127.8_{maj}, 127.7_{min}, 125.8_{min}, 125.8_{maj}, 95.4_{maj}, 95.3_{min}, 88.7 (d, *J* = 3.9 Hz)$_{min}$, 88.6 (d, *J* = 5.4 Hz)$_{maj}$, 63.6 (d, *J* = 5.9 Hz)$_{min}$, 63.5 (d, *J* = 6.7 Hz)$_{min}$, 63.3 (d, *J* = 6.5 Hz)$_{maj}$, 63.2 (d, *J* = 6.9 Hz)$_{maj}$, 60.2_{maj}, 60.1_{min}, 44.9 (d, *J* = 140.2 Hz)$_{min}$, 43.1 (d, *J* = 148.6 Hz)$_{maj}$, 36.4_{maj}, 35.0_{min}, 23.6_{maj}, 23.4_{min}, 16.5 – 16.3 (m)$_{maj+min}$, 16.1 (d, *J* = 4.7 Hz)$_{min}$, 15.2 (d, *J* = 1.9 Hz)$_{maj}$; **^{31}P NMR** (202 MHz, $CDCl_3$) δ 19.05_{maj}, 18.36_{min}; **HRMS** (ESI) mass calculated for [M+H]+ ($C_{19}H_{27}O_5NPS$) requires *m/z* 412.1342, found *m/z* 412.1334.

Synthesis of **3o**

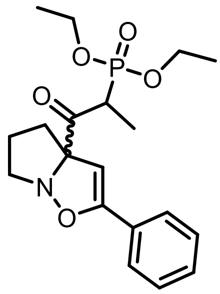

The modification to General Procedure B was followed using cycloadduct **2d** (50 mg, 0.156 mmol) and diethyl ethylphosphonate (53 μL, 0.327 mmol). Purification yielded the title compound as a light brown oil (35 mg, 60%). The product was isolated as a 2.0:1 mixture of diastereomers. **IR** (thin film) ν_{max}/cm^{-1} 2979 (C-H), 1710 (C=O), 1243 (P=O), 1022 (P-O antisymmetric); **^{1}H NMR** (500 MHz, $CDCl_3$) δ 7.56 – 7.51 (m, $2H_{min}$), 7.51 – 7.46 (m, $2H_{maj}$), 7.35 (m, $3H_{maj}+3H_{min}$), 5.24 (s, $1H_{min}$), 5.00 (s, $1H_{maj}$), 4.40 (dq, *J* = 22.2, 7.2 Hz, $1H_{maj}$), 4.21– 3.99 (m, $4H_{maj}+5H_{min}$), 3.55 – 3.42 (m, $2H_{maj}+1H_{min}$), 3.28 (ddd, *J* = 12.3, 8.4,

5.5 Hz, $1H_{min}$), 2.44 – 2.37 (m, $1H_{min}$), 2.32 (ddd, *J* = 12.9, 9.3, 7.1 Hz), 2.13 (dddd, *J* = 12.7, 6.7, 4.1, 1.1 Hz, $1H_{maj}$), 1.98 – 1.82 (m, $2H_{maj}$+$3H_{min}$), 1.43 (dd, *J* = 18.4, 7.1 Hz, $3H_{min}$), 1.33 (app d, *J* = 8.7 Hz, $9H_{maj}$), 1.28 – 1.18 (m, $6H_{min}$); **^{13}C NMR** (126 MHz, $CDCl_3$) δ 207.8 (d, *J* = 5.0 Hz)$_{maj}$, 205.6 (d, *J* = 5.5 Hz)$_{min}$, 155.1$_{min}$, 154.9$_{maj}$, 129.5$_{maj}$, 129.5$_{min}$, 128.5$_{maj+min}$, 127.9$_{min}$, 127.8$_{maj}$, 125.8$_{min}$, 125.8$_{maj}$, 95.7$_{min}$, 95.1$_{maj}$, 89.5 (d, *J* = 2.2 Hz)$_{maj}$, 89.1 (d, *J* = 2.5 Hz)$_{min}$, 62.7 – 62.6 (m)$_{maj}$, 62.5 – 62.3 (m)$_{maj}$, 60.3$_{min}$, 60.2$_{maj}$, 40.9 (d, *J* = 133.0 Hz)$_{min}$, 40.4 (d, *J* = 129.9 Hz)$_{maj}$, 35.2$_{maj}$, 33.8$_{min}$, 23.5$_{min}$, 23.4$_{maj}$, 16.6 – 16.3 (m)$_{maj}$+$_{min}$, 13.6 (d, *J* = 7.0 Hz)$_{min}$, 13.3 (d, *J* = 7.0 Hz)$_{maj}$; **^{31}P NMR** (162 MHz, $CDCl_3$) δ 24.24$_{min}$, 23.98$_{maj}$; **HRMS** (ESI) mass calculated for [M+H]+ ($C_{19}H_{27}O_5NP$) requires *m/z* 380.1621, found *m/z* 380.1616.

Synthesis of **3p**

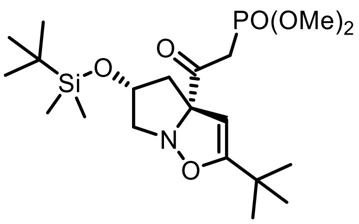

General Procedure B was followed using cycloadduct **2p** (60 mg, 0.169 mmol). Purification by FCC (1:1 pentane:EtOAc) afforded the title compound as a colourless oil (51 mg, 68%). **IR** (thin film) ν_{max}/cm^{-1} 2956 (C-H), 1717 (C=O), 1254 (P=O), 1031 (P-O antisymmetric); **^{1}H NMR** (400 MHz, $CDCl_3$) δ 4.42 – 4.34 (m, 2H), 3.78 (d, *J* = 11.1 Hz, 3H), 3.76 (d, *J* = 11.2 Hz, 3H), 3.47 (d, *J* = 20.2 Hz, 2H), 3.35 (ddd, *J* = 12.0, 4.8, 1.1 Hz, 1H), 3.18 (ddd, *J* = 12.0, 4.9, 1.2 Hz, 1H), 2.31 (ddd, *J* = 13.2, 4.9, 1.1 Hz, 1H), 2.12 (ddd, *J* = 13.2, 5.5, 1.2 Hz, 1H), 1.11 (s, 9H), 0.84 (s, 9H), 0.04 (s, 3H), 0.03 (s, 3H); **^{13}C NMR** (101 MHz, $CDCl_3$) δ 202.6 (d, *J* = 7.0 Hz), 165.5, 92.9, 86.3 (d, *J* = 4.7 Hz), 70.2, 66.2, 53.0 (d, *J* = 6.3 Hz), 52.7 (d, *J* = 6.4 Hz), 43.1, 34.9 (d, *J* = 137.5 Hz), 31.4, 28.0, 25.7, 18.0, -5.0; **^{31}P NMR** (162 MHz, $CDCl_3$) δ 24.61; **HRMS** (ESI) mass calculated for [M+H]+ ($C_{20}H_{39}O_6NPSi$) requires *m/z* 448.2279, found *m/z* 448.2275.

Synthesis of **3q**

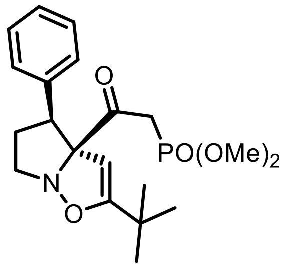

General Procedure B was followed using cycloadduct **2q** (22 mg, 0.073 mmol), 2 equivalents of $MePO(OMe)_2$ and the period at -78 °C was increased to 7 h. Purification by FCC (1:1 pentane:EtOAc) afforded the title compound as a yellow oil (15 mg, 52%). **IR** (thin film) ν_{max}/cm^{-1} 2959 (C-H), 1714 (C=O), 1259 (P=O), 1032 (P-O antisymmetric); **^{1}H NMR** (400 MHz, $CDCl_3$) δ 7.31 – 7.23 (m, 2H), 7.22 – 7.16 (m, 1H), 7.13 – 7.06 (m, 2H), 4.62 (s, 1H), 3.88 – 3.78 (m, 1H), 3.63 (m, 7H), 3.33 – 3.24 (m, 1H), 3.12 (dd, *J* = 20.5, 15.7 Hz, 1H), 2.97 (dd, *J* = 20.7, 15.7 Hz, 1H), 2.32 (app. dq, *J* = 12.6, 7.4 Hz, 1H), 2.00 – 1.89 (m, 1H), 1.16 (s, 9H); **^{13}C NMR** (101 MHz, $CDCl_3$) δ 201.2 (d, *J* = 7.4 Hz), 166.1, 139.8, 128.7, 128.0, 127.0, 93.2, 91.5 (d, *J* = 4.7 Hz), 58.5, 54.0, 52.8 (d, *J* = 30.9 Hz), 52.8 (d, *J* = 30.8 Hz), 37.3 (d, *J* = 136.6 Hz), 31.4, 30.5, 28.0; **^{31}P NMR** (162 MHz, $CDCl_3$) δ 23.53; **HRMS** (ESI) mass calculated for [M+H]+ ($C_{20}H_{29}O_5NP$) requires *m/z* 394.1778, found *m/z* 394.1767.

Synthesis of **3r**

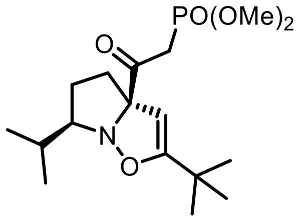

General Procedure B was followed using cycloadduct **2r** (42 mg, 0.157 mmol). FCC (1:1 pentane:EtOAc) afforded the title compound as a colourless oil (38 mg, 67%). **IR** (thin film) ν_{max}/cm^{-1} 2960 (C-H), 1715 (C=O), 1258 (P=O), 1032 (P-O antisymmetric); **^{1}H NMR** (400 MHz, $CDCl_3$) δ 4.37 (s, 1H), 3.77 (d, *J* = 11.2 Hz, 3H), 3.76 (d, *J* = 11.2 Hz, 3H), 3.54 (dd, *J* = 20.6, 15.5 Hz, 1H), 3.23 (dd, *J* = 20.5, 15.5 Hz, 1H), 2.86 (dt, *J* = 11.3, 6.2 Hz, 1H), 2.57 – 2.49 (m, 1H), 1.87 – 1.67 (m, 3H), 1.35 (dtd, *J* = 12.6, 10.9, 7.9 Hz, 1H), 1.11 (s, 9H), 1.01 (d, *J* = 6.8 Hz, 3H), 0.91 (d, *J* = 6.8 Hz, 3H); **^{13}C NMR** (101 MHz, $CDCl_3$) δ 202.4 (d, *J* = 7.0 Hz), 164.9, 93.5, 86.3 (d, *J* = 4.0 Hz), 76.0, 52.8 (d, *J* = 6.0 Hz), 52.7 (d, *J* = 6.1 Hz), 34.8 (d, *J* = 136.9 Hz), 31.6, 31.3, 31.2, 28.0, 24.3, 20.2, 19.0; **^{31}P NMR** (162

MHz, $CDCl_3$) δ 24.84; **HRMS** (ESI) mass calculated for [M+H]+ ($C_{17}H_{31}O_5NP$) requires *m/z* 360.1934, found *m/z* 360.1930.

8. Synthesis and Characterisation* of Spirocycles

Synthesis of **4a**

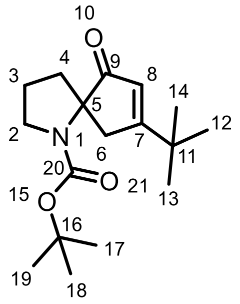

General Procedure C was followed using ketophosphonate **3a** (47 mg, 0.15 mmol). FCC purification (2:1 pentane:EtOAc) afforded the title compound as a colourless oil (38 mg, 87%). **IR** (thin film) ν_{max}/cm^{-1} 2968 (C-H), 1714 (C=O), 1695 (C=O), 1603 (C=C), 1390; **^{1}H NMR** (400 MHz, $CDCl_3$) δ 5.97 (dd, *J* = 2.1, 1.2 Hz, $1H_{min}$, 8_{min}), 5.93 (dd, *J* = 2.1, 1.3 Hz, $1H_{maj}$, 8_{maj}), 3.63 – 3.43 (m, $2H_{maj}$+$2H_{min}$, 2_{maj}, 2_{min}), 3.07 (dd, *J* = 17.0, 2.1 Hz, $1H_{min}$, 6_{min}), 2.91 (dd, *J* = 17.5, 2.0 Hz, $1H_{maj}$, 6_{maj}), 2.57 (dd, *J* = 17.5, 1.4 Hz, $1H_{maj}$, $6'_{maj}$), 2.51 (dd, *J* = 17.1, 1.2 Hz, $1H_{min}$, $6'_{min}$), 2.09 – 1.92 (m, $2H_{maj}$, 3_{maj}, 4_{maj}), 1.90 – 1.78 (m, $1H_{maj}$, $3'_{maj}$), 1.77 – 1.64 (m, $1H_{maj}$, $4'_{maj}$), 1.41 (s, $9H_{min}$, 17_{min}-19_{min}), 1.31 (s, $9H_{maj}$, 17_{maj}-19_{maj}), 1.17 (s, $9H_{maj}$+$9H_{min}$, 12_{maj}-14_{maj}, 12_{min}-14_{min}); **^{13}C NMR** (101 MHz, $CDCl_3$) δ 208.4 (9_{maj}), 208.2 (9_{min}), 185.6 (7_{min}), 185.4 (7_{maj}), 153.2 (20_{min}), 153.1 (20_{maj}), 124.8 (8_{maj}), 124.7 (8_{min}), 80.3 (5_{maj}), 79.6 (5_{min}), 69.4 (16_{min}), 68.9 (16_{maj}), 48.2 (2_{min}), 48.0 (2_{maj}), 42.6 (6_{maj}), 41.4 (6_{min}), 40.2 (4_{maj}), 39.3 (4_{min}), 35.1 (11_{min}), 35.0 (11_{maj}), 28.5 – 28.2 (m, 12_{maj}-14_{maj}, 17_{maj}-19_{maj}, 12_{min}-14_{min}, 17_{min}-19_{min}), 23.9 (3_{min}), 23.3 (3_{maj}); **HRMS** (ESI) mass calculated for [M+Na]+ ($C_{17}H_{27}O_3NNa$) requires *m/z* 316.1883, found *m/z* 316.1882.

Synthesis of **4b**

* NMR spectra of N-Boc-protected spirocycles were recorded as a mixture of rotamers in all cases (labelled maj/min where distinction possible)

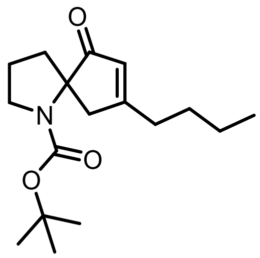

The modification to General Procedure C was followed using ketophosphonate **3b** (20 mg, 0.063 mmol). FCC purification (1:1 pentane:EtOAc) afforded the title compound as a yellow oil (7.7 mg, 42%). **IR** (thin film) ν_{max}/cm^{-1} 2970 (C-H), 1715 (C=O), 1695 (C=O), 1615, 1392, 1161; **^{1}H NMR** (500 MHz, $CDCl_3$) δ 6.00 (t, *J* = 1.5 Hz, $1H_{min}$), 5.94 (t, *J* = 1.7 Hz, $1H_{maj}$), 3.62 – 3.49 (m, $2H_{maj}$+$2H_{min}$), 3.11 – 3.05 (m, $1H_{min}$), 2.95 – 2.88 (m, $1H_{maj}$), 2.45 (dd, *J* = 22.9, 17.4 Hz, $1H_{maj}$+$1H_{min}$), 2.46 – 2.29 (m, $2H_{maj}$+$2H_{min}$), 2.06 (ddd, *J* = 14.9, 10.8, 5.0 Hz, $1H_{maj}$+$1H_{min}$), 1.99 (dddd, *J* = 12.1, 9.0, 6.2, 2.9 Hz, $1H_{maj}$+$1H_{min}$), 1.90 – 1.78 (m, $1H_{maj}$+$1H_{min}$), 1.74 (tdd, *J* = 14.9, 6.3, 3.1 Hz, $1H_{maj}$+$1H_{min}$), 1.61 – 1.51 (m, $2H_{maj}$+$2H_{min}$), 1.42 (s, $9H_{min}$), 1.41 – 1.34 (m, $2H_{maj}$+$2H_{min}$), 1.32 (s, $9H_{maj}$), 0.94 (app. dt, *J* = 10.1, 7.3 Hz, $3H_{maj}$+$3H_{min}$); **^{13}C NMR** (126 MHz, $CDCl_3$) δ $208.0_{maj+min}$, 178.3_{min}, 177.9_{maj}, 153.3_{min}, 153.1_{maj}, $127.1_{maj+min}$, 80.2_{maj}, 79.7_{min}, 68.9_{min}, 68.6_{maj}, 48.1_{min}, 48.0_{maj}, 46.3_{maj}, 45.1_{min}, 40.1_{maj}, 39.1_{min}, 33.4_{min}, 33.3_{maj}, 29.0_{maj}, 28.7_{min}, 28.5_{min}, 28.3_{maj}, 23.9_{min}, 23.3_{maj}, 22.5_{maj}, 22.5_{min}, 13.8_{min}, 13.8_{maj}; **HRMS** (ESI) mass calculated for [M+Na]+ ($C_{17}H_{27}O_3NNa$) requires *m/z* 316.1885, found *m/z* 316.1885.

Synthesis of **4c**

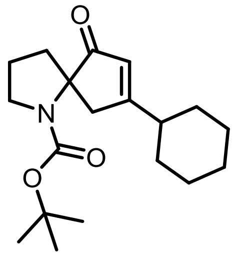

General Procedure C was followed using ketophosphonate **3c** (24 mg, 0.07 mmol). FCC purification (2:1 pentane:EtOAc) afforded the title compound as a colourless oil (15 mg, 67%). **IR** (thin film) ν_{max}/cm^{-1} 2973 (C-H), 2928 (C-H), 1716 (C=O), 1697, 1391, 1161; **^{1}H NMR** (400 MHz, $CDCl_3$) δ 5.96 (dt, *J* = 2.6, 1.3 Hz, $1H_{min}$), 5.91 (dt, *J* = 2.0, 1.3 Hz, $1H_{maj}$), 3.63 – 3.46 (m, $2H_{maj}$+$2H_{min}$), 3.10 – 3.02 (m, $1H_{min}$), 2.92 (dd, *J* = 17.7, 2.0 Hz, $1H_{maj}$), 2.53 – 2.43 (m, $1H_{maj}$+$1H_{min}$), 2.25 (m, $1H_{maj}$+$1H_{min}$), 2.04 (m, $1H_{maj}$+$1H_{min}$), 1.97 (m, $1H_{maj}$+$1H_{min}$), 1.93 – 1.77 (m, $5H_{maj}$+$5H_{min}$), 1.77 – 1.62 (m, $2H_{maj}$+$2H_{min}$), 1.41 (s, $9H_{min}$), 1.31 (s, $9H_{maj}$), 1.38 –

1.15 (m, $4H_{maj}$+$4H_{min}$); **^{13}C NMR** (101 MHz, $CDCl_3$) δ 208.1_{maj}, 208.0_{min}, 182.1_{maj}, 181.9_{min}, 153.3_{min}, 153.1_{maj}, $125.5_{maj+min}$, 80.2_{maj}, 79.6_{min}, 68.8_{min}, 68.4_{maj}, 48.2_{min}, 48.0_{maj}, 44.5_{maj}, 43.2_{min}, 42.0_{maj}, 41.8_{min}, 40.2_{maj}, 39.3_{min}, 31.1_{maj}, 30.9_{maj}, 30.8_{min}, 30.7_{min}, $28.5_{maj+min}$, 26.08 – 25.87 $(m)_{maj+min}$, 23.9_{min}, 23.3_{maj}; **HRMS** (ESI) mass calculated for [M+Na]+ ($C_{19}H_{29}O_3NNa$) requires *m/z* 342.2040, found *m/z* 342.2034.

Synthesis of **4d**

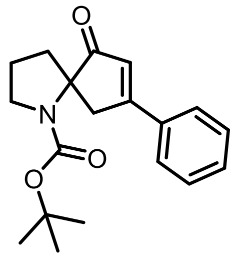

General Procedure C was followed using ketophosphonate **3d** (40 mg, 0.12 mmol). FCC purification (1:1 pentane:EtOAc) afforded the title compound as a white solid (19 mg, 52%). **MP** 128-129 °C; **IR** (thin film) ν_{max}/cm^{-1} 2974 (C-H), 1707 (C=O), 1692 (C=O), 1600 (C=C), 1392, 1161; **^{1}H NMR** (400 MHz, $CDCl_3$) δ 7.67 – 7.59 (m, $2H_{maj}$+$2H_{min}$), 7.52 – 7.38 (m, $3H_{maj}$+$3H_{min}$), 6.64 (dd, *J* = 2.1, 1.3 Hz, $1H_{min}$), 6.56 (t, *J* = 1.7 Hz, $1H_{maj}$), 3.69 – 3.54 (m, $2H_{maj}$+$2H_{min}$), 3.51 (dd, *J* = 17.0, 2.1 Hz, $1H_{min}$), 3.32 (dd, *J* = 17.4, 2.0 Hz, $1H_{maj}$), 2.97 (app. td, *J* = 17.1, 1.5 Hz, $1H_{maj}$+$1H_{min}$), 2.21 – 1.99 (m, $2H_{maj}$+$2H_{min}$), 1.99 – 1.78 (m, $2H_{maj}$+$2H_{min}$), 1.42 (s, $9H_{min}$), 1.28 (s, $9H_{maj}$); **^{13}C NMR** (101 MHz, $CDCl_3$) δ 207.6_{maj}, 207.5_{min}, 168.9_{maj}, 168.8_{min}, 153.4_{min}, 153.1_{maj}, 134.0_{min}, 133.9_{maj}, 131.4_{maj}, 131.2_{min}, 129.0_{maj}, 128.7_{min}, 127.0_{min}, 126.7_{maj}, 125.1_{min}, 125.0_{maj}, 80.3_{maj}, 79.8_{min}, 69.0_{min}, 68.6_{maj}, 48.2_{min}, 48.0_{maj}, 43.7_{maj}, 42.3_{min}, 40.2_{maj}, 39.3_{min}, 28.5_{min}, 28.2_{maj}, 24.0_{min}, 23.3_{maj}; **HRMS** (ESI) mass calculated for [M+Na]+ ($C_{19}H_{23}O_3NNa$) requires *m/z* 336.1570, found *m/z* 336.1567.

Synthesis of **4e**

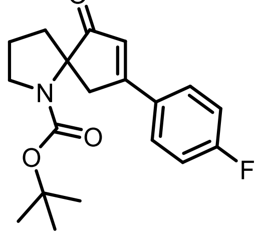

General Procedure C was followed using ketophosphonate **3e** (44 mg, 0.124 mmol). FCC purification (3:1 pentane:EtOAc) afforded the title compound as a white solid (24 mg, 59%). **MP** 162-164 °C; **IR** (thin

film) ν_{max}/cm^{-1} 2975 (C-H), 1707 (C=O), 1689 (C=O), 1602 (C=C),1392, 1161; ^{1}**H NMR** (400 MHz, $CDCl_3$) δ 7.67 – 7.58 (m, $2H_{maj}$+$2H_{min}$), 7.20 – 7.08 (m, $2H_{maj}$+$2H_{min}$), 6.57 (t, *J* = 1.7 Hz, $1H_{min}$), 6.50 (t, *J* = 1.7 Hz, $1H_{maj}$), 3.68 – 3.52 (m, $2H_{maj}$+$2H_{min}$), 3.49 (dd, *J* = 17.0, 2.1 Hz, $1H_{min}$), 3.29 (dd, *J* = 17.4, 1.9 Hz, $1H_{maj}$), 2.93 (app ddd, *J* = 18.3, 17.2, 1.4 Hz, $1H_{maj}$+$1H_{min}$), 2.21 – 2.00 (m, $2H_{maj}$+$2H_{min}$), 1.98 – 1.79 (m, $2H_{maj}$+$2H_{min}$), 1.42 (s, $9H_{min}$), 1.27 (s, $9H_{maj}$); ^{13}**C NMR** (101 MHz, $CDCl_3$) δ 207.4_{maj}, 207.3_{min}, 167.5_{maj}, 167.5_{min}, 164.8 (d, *J* = 253.5 Hz)$_{maj}$, 164.7 (d, *J* = 253.2 Hz)$_{min}$, 153.5_{min}, 153.0_{maj}, 130.4 (d, *J* = 2.9 Hz)$_{min}$, 130.2 (d, *J* = 3.2 Hz)$_{maj}$, 129.1 (d, *J* = 8.7 Hz)$_{min}$, 128.8 (d, *J* = 8.7 Hz)$_{maj}$, 124.9_{min}, 124.8_{maj}, 116.3 (d, *J* = 22.1 Hz)$_{maj}$, 115.9 (d, *J* = 22.0 Hz)$_{min}$, 80.4_{maj}, 79.9_{min}, 69.1_{min}, 68.6_{maj}, 48.2_{min}, 48.0_{maj}, 43.7_{maj}, 42.4_{min}, 40.2_{maj}, 39.3_{min}, 28.5_{min}, 28.2_{maj}, 24.0_{min}, 23.3_{maj}; ^{19}**F NMR** (377 MHz, $CDCl_3$) δ -107.66 (app tt, *J* = 8.4, 5.3 Hz)$_{maj}$, -108.09 (app tt, *J* = 8.4, 5.3 Hz)$_{min}$; **HRMS** (ESI) mass calculated for [M+Na]+ ($C_{19}H_{22}O_3NFNa$) requires *m/z* 354.1476, found *m/z* 354.1476.

Synthesis of **4f**

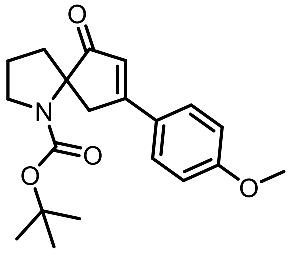

General Procedure C was followed using ketophosphonate **3f** (60 mg, 0.163 mmol). FCC purification (2:1 pentane:EtOAc) afforded the title compound as an amorphous white solid (33 mg, 59%). **IR** (thin film) ν_{max}/cm^{-1} 2984 (C-H), , 1709 (C=O), 1606 (C=C), 1112, 1059; ^{1}**H NMR** (400 MHz, $CDCl_3$) δ 7.64 – 7.55 (m, $2H_{maj}$+$2H_{min}$), 6.99 – 6.95 (m, $2H_{maj}$), 6.95 – 6.88 (m, $2H_{min}$), 6.52 (t, *J* = 1.6 Hz, $1H_{min}$), 6.45 (t, *J* = 1.6 Hz, $1H_{maj}$), 3.87 (s, $3H_{maj}$), 3.85 (s, $3H_{min}$), 3.69 – 3.53 (m, $2H_{maj}$+$2H_{min}$), 3.52 – 3.42 (m, $1H_{min}$), 3.27 (dd, *J* = 17.3, 1.9 Hz, $1H_{maj}$), 2.94 (app td, *J* = 17.2, 1.5 Hz, $1H_{maj}$+$1H_{min}$), 2.21 – 1.98 (m, $2H_{maj}$+$2H_{min}$), 1.97 – 1.77 (m, $2H_{maj}$+$2H_{min}$), 1.42 (s, $9H_{min}$), 1.27 (s, $9H_{maj}$); ^{13}**C NMR** (101 MHz, $CDCl_3$) δ 207.4_{maj}, 207.3_{min}, $168.5_{maj+min}$, 162.3_{maj}, 162.1_{min}, 153.4_{min}, 153.2_{maj},

128.8$_{min}$, 128.5$_{maj}$, 126.8$_{min}$, 126.5$_{maj}$, 123.1$_{min}$, 123.0$_{maj}$, 114.4$_{maj}$, 114.1$_{min}$, 80.2$_{maj}$, 79.7$_{min}$, 69.0$_{min}$, 68.6$_{maj}$, 55.5$_{maj}$, 55.4$_{min}$, 48.2$_{min}$, 48.0$_{maj}$, 43.6$_{maj}$, 42.3$_{min}$, 40.2$_{maj}$, 39.3$_{min}$, 28.5$_{min}$, 28.2$_{maj}$, 24.0$_{min}$, 23.3$_{maj}$; **HRMS** (ESI) mass calculated for [M+Na]+ ($C_{20}H_{25}O_4NNa$) requires *m/z* 366.1676, found *m/z* 366.1675.

Synthesis of **4g**

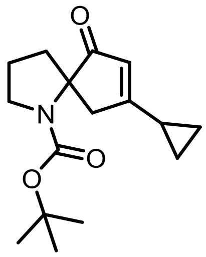

General Procedure C was followed using ketophosphonate **3g** (47 mg, 0.15 mmol). FCC purification (1:1 pentane:EtOAc) afforded the title compound as a yellow oil (10 mg, 23%). **IR** (thin film) ν_{max}/cm^{-1} 2974 (C-H), 1694 (C=O), 1602 (C=C), 1392, 1161; **^{1}H NMR** (400 MHz, $CDCl_3$) δ 5.99 (t, *J* = 1.6 Hz, 1H$_{min}$), 5.91 (t, *J* = 1.7 Hz, 1H$_{maj}$), 3.60 – 3.46 (m, 2H$_{maj}$+2H$_{min}$), 2.94 (dd, *J* = 17.2, 1.9 Hz, 1H$_{min}$), 2.75 (dd, *J* = 17.5, 1.8 Hz, 1H$_{maj}$,), 2.33 (dd, *J* = 17.5, 1.5 Hz, 1H$_{maj}$), 2.25 (dd, *J* = 17.2, 1.4 Hz, 1H$_{min}$), 2.11 – 1.91 (m, 2H$_{maj}$+2H$_{min}$), 1.87 – 1.78 (m, 2H$_{maj}$+2H$_{min}$), 1.78 – 1.64 (m, 1H$_{maj}$+1H$_{min}$), 1.42 (s, 9H$_{min}$), 1.33 (s, 9H$_{maj}$), 1.11 – 0.99 (m, 2H$_{maj}$+2H$_{min}$), 0.95 – 0.78 (m, 2H$_{maj}$+2H$_{min}$); **^{13}C NMR** (101 MHz, $CDCl_3$) δ 207.1$_{maj}$, 206.9$_{min}$, 180.3$_{min}$, 180.0$_{maj}$, 125.1$_{min}$, 124.8$_{maj}$, 80.2$_{maj}$, 79.7$_{min}$, 68.7$_{min}$, 68.2$_{maj}$, 48.2$_{min}$, 47.9$_{maj}$, 43.2$_{maj}$, 41.8$_{min}$, 40.0$_{maj}$, 39.1$_{min}$, 28.5$_{min}$, 28.3$_{maj}$, 23.8$_{min}$, 23.2$_{maj}$, 15.2$_{min}$, 14.9$_{maj}$, 10.11$_{min}$, 9.6$_{maj}$, 9.3$_{maj}$, 9.2$_{min}$; **HRMS** (ESI) mass calculated for [M+Na]+ ($C_{16}H_{23}O_3NNa$) requires *m/z* 300.1570, found *m/z* 300.1570.

Synthesis of **4h**

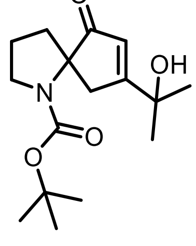

General Procedure C was followed using ketophosphonate **3h** (32 mg, 0.100 mmol) however an additional equivalent of base was used. FCC purification (EtOAc) afforded the title compound as an off-white solid (18 mg, 61%). **MP** 109-111 °C; **IR** (thin film) ν_{max}/cm^{-1} 3437 (O-H), 2976 (C-H), 1698 (C=O), 1611 (C=C), 1394, 1161; **^{1}H NMR** (400 MHz, $CDCl_3$) δ 6.11 (dd, *J* = 2.1,

1.4 Hz, $1H_{min}$), 6.10 (dd, *J* = 2.1, 1.4 Hz, $1H_{maj}$), 3.64 – 3.45 (m, $2H_{maj}$+$2H_{min}$), 3.13 (dd, *J* = 17.6, 2.1 Hz, $1H_{min}$), 2.95 (dd, *J* = 17.7, 2.1 Hz, $1H_{maj}$), 2.61 (td, *J* = 17.6, 1.4 Hz, $1H_{maj}$+$1H_{min}$), 2.50 – 2.42 (m, $1H_{min}$), 2.14 – 2.10 (m, $1H_{maj}$), 2.09 – 1.93 (m, $2H_{maj}$+$2H_{min}$), 1.91 – 1.80 (m, $1H_{maj}$+$1H_{min}$), 1.80 – 1.67 (m, $1H_{maj}$+$1H_{min}$), 1.46 (s, $3H_{maj}$+$3H_{min}$), 1.45 (s, $3H_{maj}$+$3H_{min}$), 1.40 (s, $9H_{min}$), 1.32 (s, $9H_{maj}$); **^{13}C NMR** (101 MHz, $CDCl_3$) δ 207.9_{maj}, 182.2_{min}, 181.9_{maj}, 153.4_{min}, 153.1_{maj}, 125.0_{min}, 124.9_{maj}, 80.4_{maj}, 79.8_{min}, 71.5_{maj}, 71.2_{min}, 69.3_{min}, 69.1_{maj}, 48.2_{min}, 48.0_{maj}, 42.4_{maj}, 41.2_{min}, 40.2_{maj}, 39.1_{min}, 28.8_{min}, 28.7_{min}, 28.4_{min}, 28.4_{maj}, 23.9_{min}, 23.3_{maj}; **HRMS** (ESI) mass calculated for [M+Na]+ ($C_{16}H_{25}O_4NNa$) requires *m/z* 318.1676, found *m/z* 318.1676.

Synthesis of **4i**

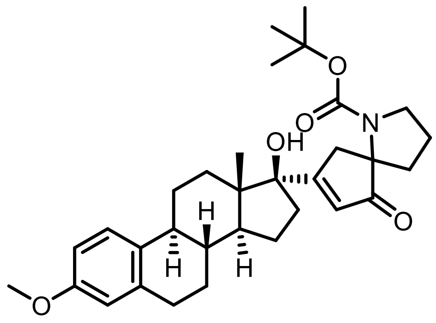

General Procedure C was followed using ketophosphonate **3i** (30 mg, 0.055 mmol) however an additional equivalent of base was used. FCC purification (2:1 pentane:EtOAc) afforded the title compound as a white solid (8.4 mg, 29%). **MP** 105-111 °C; **IR** (thin film) ν_{max}/cm^{-1} 3474 (O-H), 2929 (C-H), 1696 (C=O), 1608, 1394, 1162; **^{1}H NMR** (500 MHz, $CDCl_3$) δ 7.14 (t, *J* = 9.0 Hz, +$1H_{min}$), 6.70 (ddd, *J* = 11.9, 8.5, 2.8 Hz, $1H_{maj}$+$1H_{min}$), 6.63 (dd, *J* = 5.7, 2.7 Hz, $1H_{maj}$+$1H_{min}$), 6.06 (app d, *J* = 1.7 Hz, $1H_{min}$), 6.02 (dd, *J* = 1.1 Hz, $1H_{maj}$), 3.77 (s, $3H_{min}$), 3.77 (s, $3H_{maj}$), 3.65 – 3.58 (m, $1H_{maj}$), 3.58 – 3.45 (m, $1H_{maj}$+$2H_{min}$), 3.06 – 2.99 (m, $1H_{min}$), 2.95 (dd, *J* = 14.2, 1.7 Hz, $1H_{maj}$), 2.91 – 2.81 (m, $3H_{maj}$+$3H_{min}$), 2.32 – 2.16 (m, $3H_{maj}$+$2H_{min}$), 2.13 (app q, *J* = 10.5, 8.7 Hz, $1H_{min}$), 2.08 – 1.95 (m, $3H_{maj}$+$3H_{min}$), 1.95 – 1.83 (m, $3H_{maj}$+$3H_{min}$), 1.83 – 1.70 (m, $2H_{maj}$+$2H_{min}$), 1.70 – 1.31 (m, $8H_{maj}$+$8H_{min}$), 1.40 (s, $9H_{min}$), 1.36 (s, $9H_{maj}$), 1.02 (s, $3H_{maj}$), 0.99 (s, $3H_{min}$); **^{13}C NMR** (126 MHz, $CDCl_3$) δ 207.9_{min}, 207.0_{maj}, 181.7_{min}, 181.3_{maj}, 157.6_{maj}, 157.4_{min}, 153.2_{maj}, 153.1_{min}, 138.0_{min},

137.8$_{maj}$, 132.7$_{min}$, 132.1$_{maj}$, 129.2$_{maj}$, 129.1$_{min}$, 126.2$_{maj}$, 126.1$_{min}$, 113.9$_{maj}$, 113.7$_{min}$, 111.5$_{maj}$, 111.4$_{min}$, 85.7$_{min}$, 85.6$_{maj}$, 80.7$_{maj}$, 79.6$_{min}$, 69.1$_{maj}$, 68.2$_{min}$, 55.2$_{min}$, 55.2$_{maj}$, 49.0$_{maj}$, 48.6$_{maj}$, 48.6$_{min}$, 48.1$_{min}$, 48.1$_{min}$, 47.7$_{maj}$, 44.6$_{min}$, 44.5$_{maj}$, 43.0$_{maj}$, 42.9$_{min}$, 41.0$_{maj}$, 39.7$_{maj}$, 39.5$_{maj}$, 39.4$_{min}$, 39.1$_{min}$, 38.9$_{min}$, 33.1$_{maj}$, 32.7$_{min}$, 29.8$_{min}$, 29.7$_{maj}$, 28.6$_{maj}$, 28.4$_{min}$, 27.6$_{maj}$, 27.2$_{min}$, 26.6$_{min}$, 26.2$_{min}$, 24.1$_{maj}$, 23.7$_{min}$, 23.6$_{maj}$, 23.1$_{min}$, 14.4$_{maj}$, 14.4$_{min}$; **HRMS** (ESI) mass calculated for [M+Na]+ ($C_{32}H_{43}O_5NNa$) requires *m/z* 544.3033, found *m/z* 544.3022.

Synthesis of **4j**

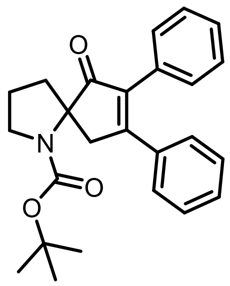

General Procedure C was followed using ketophosphonate **3j** (45 mg, 0.110 mmol). FCC purification (2:1 pentane:EtOAc) afforded the title compound as a yellow amorphous solid (15 mg, 35%). **IR** (thin film) ν_{max}/cm^{-1} 2974 (C-H), 1688 (C=O), 1695 (C=O), 1619 (C=C), 1390; **^{1}H NMR** (500 MHz, $CDCl_3$) δ 7.38 – 7.27 (m, 8H$_{maj}$+8H$_{min}$), 7.27 – 7.22 (m, 2H$_{maj}$+2H$_{min}$), 3.71 – 3.56 (m, 2H$_{maj}$+3H$_{min}$), 3.43 (d, *J* = 17.5 Hz, 1H$_{maj}$), 2.93 (d, *J* = 17.5 Hz, 1H$_{maj}$), 2.85 (d, *J* = 16.8 Hz, 1H$_{min}$), 2.28 – 2.15 (m, 1H$_{maj}$+1H$_{min}$), 2.07 (app. ddt, *J* = 11.7, 6.6, 3.2 Hz, 1H$_{maj}$+1H$_{min}$), 1.99 – 1.89 (m, 2H$_{maj}$+2H$_{min}$), 1.44 (s, 9H$_{min}$), 1.33 (s, 9H$_{maj}$); **^{13}C NMR** (126 MHz, $CDCl_3$) δ 205.8$_{maj}$, 205.9$_{min}$, 163.3$_{maj}$, 162.8$_{min}$, 153.4$_{min}$, 153.1$_{maj}$, 137.5$_{min}$, 137.1$_{maj}$, 135.7$_{min}$, 135.6$_{maj}$, 132.4$_{min}$, 132.0$_{maj}$, 129.9$_{maj}$, 129.8$_{min}$, 129.7$_{min}$, 129.5$_{maj}$, 128.5$_{maj}$, 128.5$_{min}$, 128.4$_{maj}$, 128.3$_{min}$, 128.3$_{min}$, 128.1$_{maj}$, 128.0$_{maj}$, 127.8$_{min}$, 80.2$_{maj}$, 79.6$_{min}$, 68.9$_{min}$, 68.2$_{maj}$, 48.4$_{min}$, 48.1$_{maj}$, 44.9$_{maj}$, 43.5$_{min}$, 40.6$_{maj}$, 39.6$_{min}$, 28.5$_{min}$, 28.5$_{maj}$, 23.9$_{min}$, 23.4$_{maj}$; **HRMS** (ESI) mass calculated for [M+Na]+ ($C_{25}H_{27}O_3NNa$) requires *m/z* 412.1883, found *m/z* 412.1895

Synthesis of **4k**

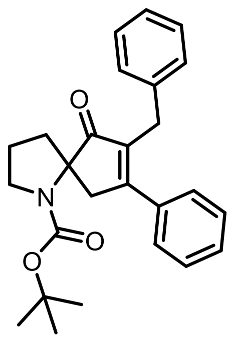

General Procedure C was followed using ketophosphonate **3k** (40 mg, 0.09 mmol). FCC purification (2:1 pentane:EtOAc) afforded the title compound as an off-white amorphous solid (12 mg, 32%). **IR** (thin film) ν_{max}/cm^{-1} 2973 (C-H), 1693 (C=O), 1620 (C=C), 1391; **^{1}H NMR** (400 MHz, $CDCl_3$) δ 7.48 – 7.38 (m, $5H_{maj}$), 7.38 – 7.34 (m, $5H_{min}$), 7.28 – 7.23 (m, $2H_{maj}$+$2H_{min}$), 7.22 – 7.13 (m, $3H_{maj}$+$3H_{min}$), 3.97 – 3.85 (m, $1H_{maj}$), 3.74 (d, *J* = 15.5 Hz, $1H_{min}$), 3.69 – 3.55 (m, $3H_{maj}$+$3H_{min}$), 3.46 (m, $1H_{maj}$+$1H_{min}$), 2.81 (m, $1H_{maj}$+$1H_{min}$), 2.21 – 2.00 (m, $2H_{maj}$+$2H_{min}$), 1.97 – 1.80 (m, $2H_{maj}$+$2H_{min}$), 1.45 (s, $9H_{min}$), 1.26 (s, $9H_{maj}$); **^{13}C NMR** (101 MHz, $CDCl_3$) δ 208.0_{min}, 207.4_{maj}, 164.1_{maj}, 153.2_{maj}, 139.1_{maj}, 136.8_{maj}, 135.8_{maj}, 129.9_{maj}, 129.7_{min}, 128.8_{maj}, 128.6_{maj}, 128.5_{min}, 128.2_{maj}, 128.1_{min}, 127.8_{min}, 127.4_{maj}, 126.2_{maj}, 125.9_{min}, 80.11_{maj}, 79.7_{min}, 68.3_{min}, 67.7_{maj}, 48.3_{min}, 48.1_{maj}, 45.1_{maj}, 43.5_{min}, 40.3_{maj}, 39.3_{min}, 30.1_{min}, 30.0_{maj}, 28.5_{min}, 28.3_{maj}, 23.9_{min}, 23.3_{maj}; **HRMS** (ESI) mass calculated for [M+Na]+ ($C_{26}H_{29}O_3NNa$) requires *m/z* 426.2040, found *m/z* 426.2038.

Synthesis of **4l**

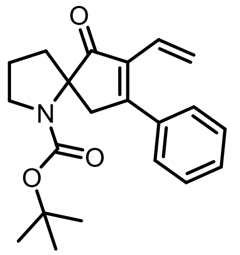

General Procedure C was followed using ketophosphonate **3l** (22 mg, 0.057 mmol). FCC purification (2:1 pentane:EtOAc) afforded the title compound as a yellow oil (13 mg, 67%). **IR** (thin film) ν_{max}/cm^{-1} 2974 (C-H), 1710 (C=O), 1696 (C=O), 1391, 1171; **^{1}H NMR** (400 MHz, $CDCl_3$) δ 7.56 – 7.36 (m, $5H_{maj}$+$5H_{min}$), 6.58 – 6.27 (m, $2H_{maj}$+$2H_{min}$), 5.49 – 5.39 (m, $1H_{maj}$+$1H_{min}$), 3.67 – 3.54 (m, $2H_{maj}$+$3H_{min}$), 3.29 (d, *J* = 18.1 Hz, $1H_{maj}$), 2.87 – 2.79 (m, $1H_{maj}$), 2.72 (d, *J* = 17.3 Hz, $1H_{min}$), 2.24 – 2.10 (m, $1H_{maj}$+$1H_{min}$), 2.05 (tdd, *J* = 11.0, 5.5, 2.4 Hz, $1H_{maj}$+$1H_{min}$), 1.94 – 1.76 (m, $2H_{maj}$+$2H_{min}$), 1.44 (s, $9H_{min}$), 1.29 (s, $9H_{maj}$); **^{13}C NMR** (101 MHz, $CDCl_3$) δ 206.2_{maj}, 163.7_{maj}, 153.1_{maj}, 135.9_{maj}, 132.7_{maj}, 129.9_{maj}, $129.7_{min,}$

128.7$_{maj}$, 128.4$_{min}$, 128.4$_{min}$, 127.9$_{maj}$, 126.8$_{min}$, 126.7$_{maj}$, 121.0$_{maj}$, 120.9$_{min}$, 80.2$_{maj}$, 79.7$_{min}$, 68.0$_{maj}$, 48.3$_{min}$, 48.0$_{maj}$, 44.8$_{maj}$, 43.1$_{min}$, 40.3$_{maj}$, 39.5$_{min}$, 28.5$_{min}$, 28.3$_{maj}$, 23.9$_{min}$, 23.5$_{maj}$; **HRMS** (ESI) mass calculated for [M+Na]+ ($C_{21}H_{25}O_3NNa$) requires *m/z* 362.1727, found *m/z* 362.1730.

Synthesis of **4m**

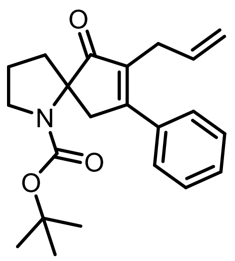

General Procedure C was followed using ketophosphonate **3m** (27 mg, 0.07 mmol). FCC purification (2:1 pentane:EtOAc) afforded the title compound as a yellow oil (18 mg, 70%). **IR** (thin film) ν_{max}/cm^{-1} 2968 (C-H), 1705 (C=O), 1693 (C=O), 1621 (C=C), 1390; **^{1}H NMR** (400 MHz, $CDCl_3$) δ 7.55 – 7.38 (m, 5H$_{maj}$+5H$_{min}$), 5.97 (ddt, *J* = 17.1, 10.3, 5.8 Hz), 5.13 – 5.00 (m, 2H$_{maj}$+2H$_{min}$), 3.69 – 3.53 (m, 2H$_{maj}$+3H$_{min}$), 3.45 – 3.36 (m, 1H$_{maj}$), 3.34 – 3.19 (m, 1H$_{maj}$+1H$_{min}$), 3.16 – 3.05 (m, 1H$_{maj}$+1H$_{min}$), 2.77 (dd, *J* = 18.4, 17.2 Hz, 1H$_{maj}$+1H$_{min}$), 2.19 – 1.98 (m, 2H$_{maj}$+2H$_{min}$), 1.95 – 1.78 (m, 2H$_{maj}$+2H$_{min}$), 1.44 (s, 9H$_{min}$), 1.29 (s, 9H$_{maj}$); **^{13}C NMR** (126 MHz, $CDCl_3$) δ 207.0$_{maj}$, 206.9$_{min}$, 163.4$_{maj}$, 163.3$_{min}$, 153.3$_{min}$, 153.2$_{maj}$, 135.9$_{min}$, 135.8$_{min}$, 135.7$_{maj}$, 134.9$_{min}$, 134.7$_{maj}$, 129.9$_{maj}$, 129.7$_{min}$, 128.8$_{maj}$, 128.5$_{min}$, 127.8$_{min}$, 127.5$_{maj}$, 115.8$_{maj}$, 115.5$_{min}$, 80.1$_{maj}$, 79.6$_{min}$, 68.2$_{min}$, 67.6$_{maj}$, 48.3$_{min}$, 48.0$_{maj}$, 44.8$_{maj}$, 43.4$_{min}$, 40.2$_{maj}$, 39.4$_{min}$, 28.7$_{maj}$, 28.6$_{min}$, 28.5$_{min}$, 28.3$_{maj}$, 23.9$_{min}$, 23.4$_{maj}$; **HRMS** (ESI) mass calculated for [M+Na]+ ($C_{22}H_{27}O_3NNa$) requires *m/z* 376.1883, found *m/z* 376.1877.

Synthesis of **4n**

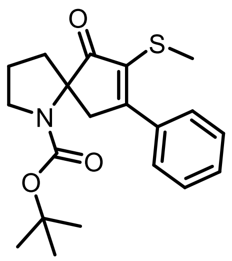

General Procedure C was followed using ketophosphonate **3n** (22 mg, 0.054 mmol). FCC purification (2:1 pentane:EtOAc) afforded the title compound as a yellow oil (14 mg, 74%). **IR** (thin film) ν_{max}/cm^{-1} 2980 (C-H), 1713 (C=O), 1693 (C=O), 1390, 1167, 767; **^{1}H NMR** (400 MHz, $CDCl_3$) δ 7.79 – 7.74 (m, 2H$_{min}$), 7.74 – 7.67 (m, 2H$_{maj}$), 7.52 – 7.39 (m, 3H$_{maj}$+3H$_{min}$), 3.70 –

3.51 (m, 2H$_{maj}$+3H$_{min}$), 3.34 (d, *J* = 17.4 Hz, 1H$_{maj}$), 2.94 (d, *J* = 17.4 Hz, 1H$_{maj}$), 2.85 (d, *J* = 16.8 Hz, 1H$_{min}$), 2.46 (s, 3H$_{maj}$), 2.44 (s, 3H$_{min}$), 2.21 – 1.97 (m, 2H$_{maj}$+2H$_{min}$), 1.97 – 1.78 (m, 2H$_{maj}$+2H$_{min}$), 1.43 (s, 9H$_{min}$), 1.32 (s, 9H$_{maj}$); **^{13}C NMR** (101 MHz, $CDCl_3$) δ 204.7$_{maj}$, 163.6$_{min}$, 163.2$_{maj}$, 153.1 – 152.9 (m)$_{maj+min}$, 135.2$_{maj}$, 132.4$_{maj}$, 130.3 $_{maj}$, 130.2$_{min}$, 128.5$_{maj}$, 128.2$_{min}$, 128.0$_{maj+min}$, 81.2$_{min}$, 80.4$_{maj}$, 68.0$_{min}$, 67.5$_{maj}$, 48.0$_{maj}$, 45.2$_{maj}$, 43.8$_{min}$, 40.3$_{maj}$, 28.5$_{min}$, 28.3$_{maj}$, 23.3$_{maj}$, 15.3$_{min}$, 14.9$_{maj}$; **HRMS** (ESI) mass calculated for [M+Na]+ ($C_{20}H_{25}O_3NNaS$) requires *m/z* 382.1447, found *m/z* 382.1449.

Synthesis of **4o**

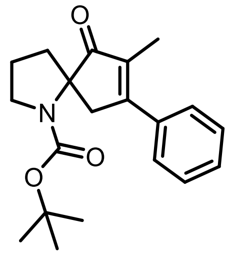

General Procedure C was followed using ketophosphonate **3o** (30 mg, 0.079 mmol). FCC purification (2:1 pentane:EtOAc) afforded the title compound as a colourless oil (17 mg, 66%). **IR** (thin film) ν_{max}/cm^{-1} 2974 (C-H), 1698 (C=O), 1392, 1172; **^{1}H NMR** (400 MHz, $CDCl_3$) δ 7.58 – 7.38 (m, 5H$_{maj}$+5H$_{min}$), 3.69 – 3.54 (m, 2H$_{maj}$+2H$_{min}$), 3.49 (dd, *J* = 16.6, 2.6 Hz, 1H$_{min}$), 3.32 – 3.23 (m, 1H$_{maj}$), 2.81 (dd, *J* = 17.1, 1.8 Hz, 1H$_{maj}$), 2.73 (dd, *J* = 16.6, 1.8 Hz, 1H$_{min}$), 2.18 – 1.99 (m, 2H$_{maj}$+2H$_{min}$), 2.04 (m, 3H$_{min}$), 2.02 – 1.99 (m, 3H$_{maj}$), 1.96 – 1.75 (m, 2H$_{maj}$+2H$_{min}$), 1.43 (s, 9H$_{min}$), 1.28 (s, 9H$_{maj}$); **^{13}C NMR** (101 MHz, $CDCl_3$) δ 207.9$_{maj}$, 161.8$_{maj}$, 153.3$_{maj}$, 136.0$_{maj}$, 134.1$_{maj}$, 129.6$_{maj}$, 129.4$_{min}$, 128.7$_{maj}$, 128.4$_{min}$, 128.0$_{min}$, 127.6$_{maj}$, 80.0$_{maj}$, 67.4$_{maj}$, 48.2$_{min}$, 47.9$_{maj}$, 44.8$_{maj}$, 43.2$_{min}$, 40.2$_{maj}$, 39.5$_{min}$, 28.5$_{min}$, 28.3$_{maj}$, 24.0$_{min}$, 23.5$_{maj}$, 10.5$_{min}$, 10.3$_{maj}$; **HRMS** (ESI) mass calculated for [M+Na]+ ($C_{20}H_{25}O_3NNa$) requires *m/z* 350.1727, found *m/z* 350.1725

Synthesis of **4p**

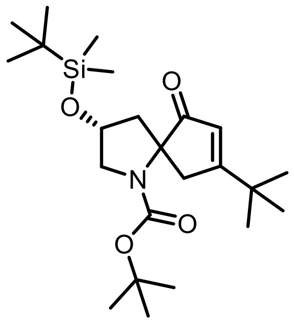

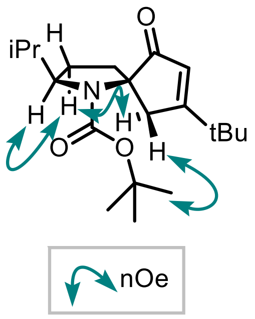

General Procedure C was followed using ketophosphonate **3p** (46 mg, 0.103 mmol).

FCC purification (10:1 pentane:EtOAc) afforded the title compound as a white solid (28 mg, 64%). **MP** 143-144 °C; **IR** (thin film) ν_{max}/cm^{-1} 2959 (C-H), 1716 (C=O), 1699 (C=O), 1603, 1392, 1125, 1110, 838; **^{1}H NMR** (400 MHz, $CDCl_3$) δ 5.99 (dd, *J* = 2.1, 1.2 Hz, $1H_{min}$), 5.94 (dd, *J* = 2.0, 1.3 Hz, $1H_{maj}$), 4.42 – 4.29 (m, $1H_{maj}$+$1H_{min}$), 3.77 (m, $1H_{maj}$), 3.67 (ddd, *J* = 10.0, 7.2, 1.1 Hz, $1H_{min}$), 3.24 (m, $1H_{maj}$+$1H_{min}$), 3.04 – 2.96 (m, $1H_{min}$), 2.93 – 2.84 (m, $1H_{maj}$), 2.56 (dd, *J* = 17.5, 1.4 Hz, $1H_{maj}$), 2.49 (dd, *J* = 17.0, 1.2 Hz, $1H_{min}$), 2.08 (ddd, *J* = 11.6, 10.5, 0.9 Hz, $1H_{maj}$), 2.00 (ddd, *J* = 11.6, 10.5, 0.9 Hz, $1H_{min}$), 1.85 – 1.70 (m, $1H_{maj}$+$1H_{min}$), 1.41 (s, $9H_{min}$), 1.30 (s, $9H_{maj}$), 1.17 (s, $9H_{maj}$), 1.17 (s, $9H_{min}$), 0.86 (s, $9H_{min}$), 0.85 (s, $9H_{maj}$), 0.06 (s, $3H_{min}$), 0.05 (s, $3H_{maj}$+$3H_{min}$), 0.04 (s, $3H_{maj}$); **^{13}C NMR** (101 MHz, $CDCl_3$) δ 207.3_{maj}, 207.1_{min}, 184.9_{min}, 184.8_{maj}, 153.1_{min}, 152.8_{maj}, $125.0_{maj+min}$, 80.6_{maj}, 79.9_{min}, 68.9_{min}, 68.3_{maj}, 67.8_{min}, 67.3_{maj}, 54.0_{min}, 53.6_{maj}, 47.8_{maj}, 47.1_{min}, 42.9_{maj}, 41.8_{min}, 35.0_{min}, 35.0_{maj}, 28.4_{min}, 28.4_{maj}, 28.3_{maj}, 28.2_{min}, $25.7_{maj+min}$, $17.9_{maj+min}$, -4.8_{maj}, -4.8_{min}, -4.9_{maj}; **HRMS** (ESI) mass calculated for [M+H]+ ($C_{23}H_{42}O_4NSi$) requires *m/z* 424.2878, found *m/z* 424.2873

Synthesis of **4q**

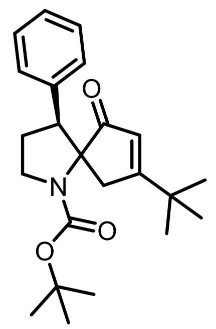

General Procedure C was followed using ketophosphonate **3q** (26 mg, 0.066 mmol). FCC purification (3:1 pentane:EtOAc) afforded the title compound as a yellow oil (17 mg, 69%). **IR** (thin film) ν_{max}/cm^{-1} 2969 (C-H), 1705 (C=O), 1694 (C=O), 1604, 1389, 1176, 1129; **^{1}H NMR** (400 MHz, $CDCl_3$) δ 7.20 (app. tt, *J* = 3.8, 1.6 Hz, $3H_{maj}$+$3H_{min}$), 7.14 (app. dt, *J* = 6.1, 3.4 Hz, $2H_{maj}$+$2H_{min}$), 5.45 (t, *J* = 1.6 Hz, $1H_{min}$), 5.41 – 5.35 (m, $1H_{maj}$), 4.09 (dd, *J* = 10.6, 8.3 Hz, $1H_{maj}$), 3.94 (dd, *J* = 10.5, 8.5 Hz, $1H_{min}$), 3.57 – 3.38 (m, $1H_{maj}$+$2H_{min}$), 3.36 – 3.21 (m, $2H_{maj}$+$1H_{min}$), 2.79 – 2.58 (m, $2H_{maj}$, 3_{maj}+$2H_{min}$), 1.91 (dtd, *J* = 12.0, 5.7, 2.7 Hz, $1H_{maj}$+$1H_{min}$), 1.45 (s, $9H_{min}$), 1.32 (s, $9H_{maj}$), 0.96 (s, $9H_{maj}$+$9H_{min}$); **^{13}C NMR** (101 MHz,

CDCl$_3$) δ 208.0$_{min}$, 207.7$_{maj}$, 185.9$_{min}$, 185.2$_{maj}$, 153.9$_{maj}$, 153.8$_{min}$, 136.3$_{min}$, 136.3$_{maj}$, 128.4$_{min}$, 128.4$_{maj}$, 128.2$_{maj+min}$, 127.7$_{maj}$, 127.6$_{min}$, 125.3$_{min}$, 125.2$_{maj}$, 80.4$_{maj}$, 79.7$_{min}$, 72.8$_{min}$, 72.5$_{maj}$, 57.4$_{maj}$, 56.3$_{min}$, 47.2$_{min}$, 47.0$_{maj}$, 41.2$_{maj}$, 39.4$_{min}$, 34.9$_{min}$, 34.8$_{maj}$, 28.5$_{min}$, 28.4$_{maj}$, 28.4$_{maj}$, 28.4$_{min}$, 28.0$_{min}$, 27.6$_{maj}$; **HRMS** (ESI) mass calculated for [M+Na]+ ($C_{23}H_{31}O_3NNa$) requires *m/z* 392.2196, found *m/z* 392.2198.

Synthesis of **4r**

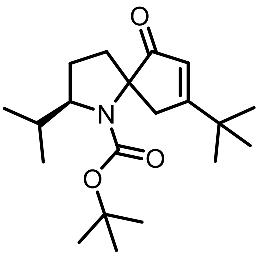

General Procedure C was followed using ketophosphonate **3r** (36 mg, 0.100 mmol). FCC purification (2:1 pentane:EtOAc) afforded the title compound as a yellow oil (16 mg, 47%). **IR** (thin film) ν_{max}/cm^{-1} 2964 (C-H), 1717 (C=O), 1693 (C=O), 1603 (C=C), 1378, 1366, 1114; **^{1}H NMR** (400 MHz, CDCl$_3$) δ 5.97 (t, *J* = 1.6 Hz, 1H$_{min}$), 5.93 (t, *J* = 1.6 Hz, 1H$_{maj}$), 3.80 (ddd, *J* = 7.5, 5.1, 1.7 Hz, 1H$_{maj}$), 3.65 (dd, *J* = 7.8, 5.7 Hz, 1H$_{min}$), 3.02 – 2.96 (m, 1H$_{min}$), 2.81 (dt, *J* = 17.4, 1.4 Hz, 1H$_{maj}$), 2.54 (dd, *J* = 17.4, 1.4 Hz, 1H$_{maj}$), 2.44 (dd, *J* = 16.8, 1.2 Hz, 1H$_{min}$), 2.30 (m, 1H$_{maj}$+1H$_{min}$), 2.17 – 1.97 (m, 1H$_{maj}$+1H$_{min}$), 1.95 – 1.75 (m, 2H$_{maj}$+2H$_{min}$), 1.53 – 1.44 (m, 1H$_{maj}$+1H$_{min}$), 1.41 (s, 9H$_{min}$), 1.31 (s, 9H$_{maj}$), 1.16 (s, 9H$_{maj}$), 1.16 (s, 9H$_{min}$), 0.98 (d, *J* = 6.9 Hz, 3H$_{maj}$+3H$_{min}$), 0.93 (d, *J* = 7.0 Hz, 3H$_{maj}$+3H$_{min}$). **^{13}C NMR** (101 MHz, CDCl$_3$) δ 210.6$_{min}$, 207.8$_{maj}$, 184.8$_{min}$, 184.1$_{maj}$, 153.4$_{min}$, 153.3$_{maj}$, 125.2$_{maj}$, 125.0$_{min}$, 80.2$_{maj}$, 79.6$_{min}$, 70.4$_{min}$, 69.8$_{maj}$, 64.2$_{maj}$, 64.0$_{min}$, 42.6$_{maj}$, 40.8$_{min}$, 39.1$_{maj}$, 38.1$_{min}$, 35.0$_{min}$, 34.9$_{maj}$, 31.5$_{min}$, 30.5$_{maj}$, 28.4$_{maj+min}$, 28.3$_{maj}$, 28.2$_{min}$, 25.8$_{min}$, 24.6$_{maj}$, 20.5$_{min}$, 20.2$_{maj}$, 17.7$_{min}$, 17.3$_{maj}$. **HRMS** (ESI) mass calculated for [M+Na]+ ($C_{20}H_{33}O_3NNa$) requires *m/z* 358.2353, found *m/z* 358.2346.

Synthesis of **4a’**

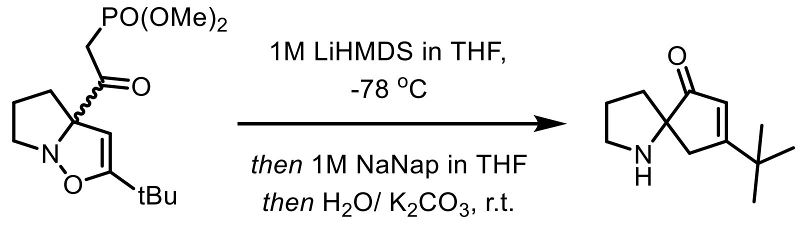

To a solution of ketophosphonate **3a** (36 mg, 0.11 mmol) in dry THF (2.3 mL) at -78 °C was added 1M LiHMDS in THF (114 µL, 0.114 mmol). The reaction was stirred for 10 min before adding 1M sodium naphthalenide in THF (568 µL, 0.568 mmol) dropwise until complete consumption of starting material was observed by TLC. H_2O (0.5 mL) was then added, followed by addition of sat. aq. K_2CO_3 (1 mL). The reaction mixture was then stirred for 30 min. The aqueous phase was then extracted with EtOAc (3 x 20 mL) and the organic layers were combined, dried with $MgSO_4$ and filtered before removal of the solvent under reduced pressure. The resulting crystalline crude material was purified by FCC (9:1 EtOAc:MeOH) to yield the title compound as a yellow oil (14 mg, 63%). **IR** (thin film) ν_{max}/cm^{-1} 2965 (C-H), 1704 (C=O), 1599 (C=C), 1249, 867; **^{1}H NMR** (400 MHz, $CDCl_3$) δ 5.93 (t, *J* = 1.7 Hz, 1H), 3.30 – 3.20 (m, 1H), 3.02 – 2.93 (m, 1H), 2.64 (d, *J* = 1.7 Hz, 2H), 2.28 – 2.13 (m, 1H), 2.01 – 1.77 (m, 3H,), 1.72 – 1.60 (m, 1H), 1.17 (s, 9H); **^{13}C NMR** (101 MHz, $CDCl_3$) δ 211.6, 188.8, 124.4, 70.6, 47.9, 45.2, 38.6, 35.2, 28.4, 26.5. **HRMS** (ESI) mass calculated for [M+H]+ ($C_{12}H_{20}ON$) requires *m/z* 194.15394, found *m/z* 194.15402.

9. Formal Synthesis of (±)-Cephalotaxine

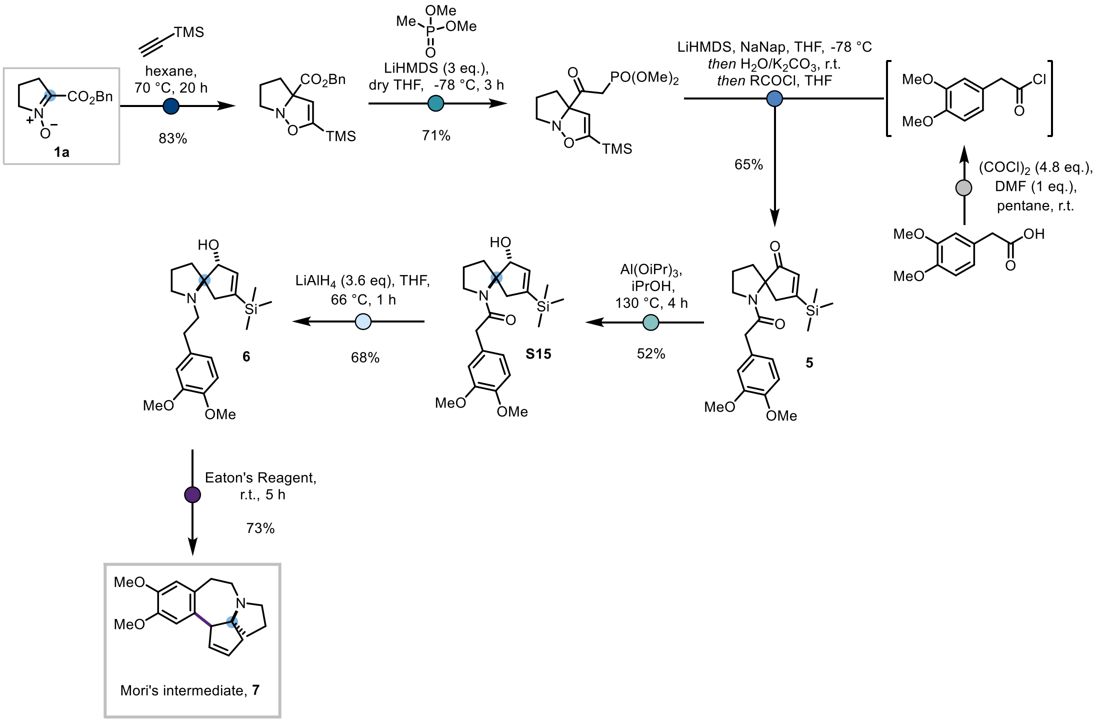

Scheme S5. Formal synthesis of (±)-cephalotaxine.

Synthesis of **2s**

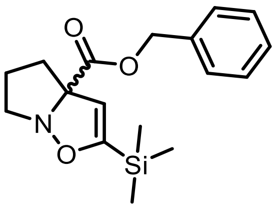

General Procedure A was followed using nitrone **1a** (100 mg, 0.456 mmol), trimethylsilane acetylene (190 µL) and stirred for 22 h. The resulting oil was purified by FCC (4:1 pentane:EtOAc) to give the title compound as a colourless oil (97 mg, 67%). **IR** (thin film) ν_{max}/cm^{-1} 2960 (C-H), 1732 (C=O), 1249, 1109, 836 (C-Si); **^{1}H NMR** (500 MHz, $CDCl_3$) δ 7.39 – 7.28 (m, 5H), 5.22 (d, *J* = 12.5 Hz, 1H), 5.17 (d, *J* = 12.5 Hz, 1H), 4.87 (s, 1H), 3.39 – 3.24 (m, 2H), 2.16 (ddd, *J* = 12.7, 9.2, 6.9 Hz, 1H), 2.00 (dddd, *J* = 12.4, 6.9, 4.1, 1.1 Hz, 1H), 1.84 – 1.68 (m, 2H), 0.17 (s, 9H); **^{13}C NMR** (126 MHz, $CDCl_3$) δ 172.9, 160.0, 136.0, 128.5, 128.1, 127.8, 108.7, 82.0, 66.7, 60.4, 36.9, 23.1, -2.2; **HRMS** (ESI) mass calculated for [M+H]+ ($C_{17}H_{24}O_3NSi$) requires *m/z* 318.1520, found *m/z* 318.1520. As a large-scale modification to General Procedure A, nitrone **1a** (3.59 g, 16.4 mmol) was stirred in hexane at 70 °C in a round-

bottomed flask with a reflux condenser attached. To this was added TMS-acetylene (6.8 mL, 49.2 mmol) and the heterogeneous mixture stirred vigorously under an N_2 atmosphere for 20 h. The solvent was then removed under reduced pressure to yield an orange oil as the crude product. FCC purification (4:1 pentane: EtOAc) yielded the title compound as a colourless oil (4.30 g, 83%).

Synthesis of **3s**

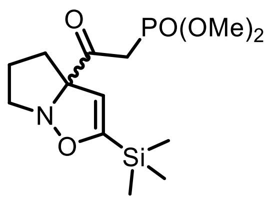

General Procedure B was followed using cycloadduct **2s** (50 mg, 0.158 mmol). FCC afforded the title compound as a yellow oil (34 mg, 65%). **IR** (thin film) ν_{max}/cm^{-1} 3032 (C-H), 1713 (C=O), 1250 (P=O), 1031 (P-O antisymmetric); **^{1}H NMR** (400 MHz, $CDCl_3$) δ 4.69 (s, 1H), 3.79 (d, *J* = 6.9 Hz, 3H), 3.76 (d, *J* = 6.9 Hz, 3H), 3.62 (dd, *J* = 21.3, 14.9 Hz, 1H), 3.30 – 3.22 (m, 2H), 3.21 (dd, *J* = 21.0, 14.9 Hz, 1H), 2.26 (m, *J* = 12.8, 8.0 Hz, 1H), 1.92 – 1.85 (m, 1H), 1.76 – 1.68 (m, 2H), 0.17 (s, 9H); **^{13}C NMR** (101 MHz, $CDCl_3$) δ 204.3 (d, *J* = 6.4 Hz), 163.1, 111.1, 90.3 (d, *J* = 3.6 Hz), 62.4, 55.2 (d, *J* = 6.3 Hz), 55.1 (d, *J* = 6.3 Hz), 37.8 (d, *J* = 134.9 Hz), 36.1, 25.5, -0.0; **^{31}P NMR** (162 MHz, $CDCl_3$) δ 24.17; **HRMS** (ESI) mass calculated for [M+H]+ ($C_{13}H_{25}O_5NPSi$) requires *m/z* 334.1234, found *m/z* 334.1232. On a larger scale, General Procedure B was followed using cycloadduct **2s** (4.3 g, 13.6 mmol) to yield the title compound as a yellow oil (3.20 g, 71%).

Synthesis of **4s**

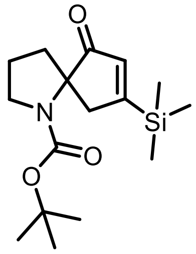

General Procedure C was followed using ketophosphonate **3s** (33 mg, 0.099 mmol). FCC purification (4:1 pentane:EtOAc) afforded the title compound as a colourless oil (15 mg, 49%). **IR** (thin film) ν_{max}/cm^{-1} 2971 (C-H), 1717 (C=O), 1697 (C=O), 1391, 1210, 842; **^{1}H NMR** (400 MHz, $CDCl_3$) δ 6.41 (dd, *J* = 2.5, 1.5 Hz, $1H_{min}$), 6.36 (dd, *J* = 2.4, 1.8 Hz, $1H_{maj}$), 3.63 – 3.45 (m, $2H_{maj}$+$2H_{min}$),

3.13 (dd, J = 17.5, 2.5 Hz, $1H_{min}$), 2.98 (dd, J = 18.1, 2.4 Hz, $1H_{maj}$), 2.62 (ddd, J = 24.5, 17.8, 1.7 Hz, $1H_{maj}$+$1H_{min}$), 2.07 – 1.93 (m, $2H_{maj}$+$2H_{min}$), 1.92 – 1.77 (m, $1H_{maj}$+$1H_{min}$), 1.77 – 1.59 (m, $1H_{maj}$+$1H_{min}$), 1.41 (s, $9H_{min}$), 1.30 (s, $9H_{maj}$), 0.20 (s, $9H_{maj}$), 0.19 (s, $9H_{min}$); **^{13}C NMR** (101 MHz, $CDCl_3$) δ 208.6_{maj}, 179.3_{maj}, 153.0_{maj}, 139.1_{min}, 139.1_{maj}, 80.2_{maj}, 79.6_{min}, 68.6_{min}, 67.9_{maj}, 48.1_{min}, 47.9_{maj}, 46.5_{maj}, 45.2_{min}, 40.3_{maj}, 39.5_{min}, 28.5_{min}, 28.3_{maj}, 24.0_{min}, 23.4_{maj}, $-2.6_{maj+min}$; **HRMS** (ESI) mass calculated for [M+Na]+ ($C_{16}H_{27}O_3NNaSi$) requires *m/z* 332.1652, found *m/z* 332.1651.

2-(3,4-Dimethoxyphenyl)acetyl chloride (S14)

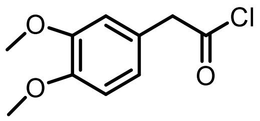

Oxalyl chloride (8.04 mL, 95 mmol) was added to a solution of homoveratric acid (3.92 g, 20.0 mmol) and DMF (1.54 mL) in pentane (800 mL) at r.t.. After 1 h the solid was observed to have been completely consumed and the mixture was filtered and the residue concentrated to afford a yellow oil. The yield was assumed to be quantitative and this crude product was used directly in preparing spiroenone **5** without purification or characterisation.

Synthesis of **5**

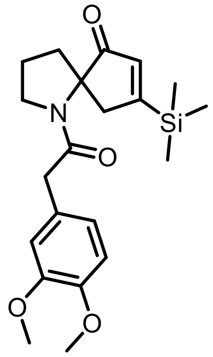

General Procedure C was followed using ketophosphonate **3s** (100 mg, 0.30 mmol) however, rather than quenching with a THF solution of Boc_2O, a THF solution of acid chloride **S14** (2 eq.) was used. The resulting biphasic mixture was stirred for 6 hours before being worked up in the usual manner. The crude crystalline material was purified by FCC (EtOAc) to yield the title compound as a colourless oil (76 mg, 65%) **IR** (thin film) ν_{max}/cm^{-1} 2954 (C-H), 1711 (C=O), 1638 (C=O), 1590 (C=C), 1515, 1409, 1249, 1020, 841; **^{1}H NMR** (400 MHz, $CDCl_3$) δ 6.84 – 6.73 (m, 3H), 6.42 (dd, J = 2.4, 1.6 Hz, 1H), 3.89 (s, 3H), 3.84 (s, 3H), 3.63 – 3.48 (m, 4H), 3.15 (dd, J = 17.5, 2.4 Hz, 1H), 2.54 (dd, J = 17.6, 1.6 Hz, 1H), 2.09 –

1.98 (m, 1H), 1.99 – 1.86 (m, 2H), 1.68 – 1.59 (m, 1H), 0.17 (s, 9H); **^{13}C NMR** (101 MHz, $CDCl_3$) δ 207.2, 178.2, 168.7, 149.2, 147.8, 139.2, 127.0, 121.0, 111.8, 111.3, 69.4, 55.9 (app. d, *J*= 1.8 Hz), 48.6, 44.9, 41.7, 38.5, 24.7, -2.6; **HRMS** (ESI) mass calculated for [M+H]+ ($C_{21}H_{30}O_4NSi$) requires *m/z* 388.1932, found *m/z* 388.1928. This procedure was repeated on a larger scale (2.30 g, 6.9 mmol) to yield the title compound as a yellow oil (1.49 g, 56%).

Synthesis of **S15**

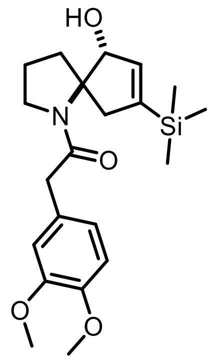

Following a modified literature procedure,[18] to a stirred solution of spiroenone **5** (800 mg, 2.1 mmol) in propan-2-ol (21 mL, 0.1 M) was added Al(O*i*-Pr)$_3$ (16 g, 79 mmol). The mixture was heated at 80 °C and the temperature was increased to 130 °C over the course of 30 min with concurrent distillation of the solvent. The reaction was then stirred for 2 h at 130 °C. The molten mixture was poured into 1 M HCl (400 mL) at -10 °C. The solid residue left in the flask was dissolved in cold 1M HCl using sonication and this was added to the rest of the reaction mixture. The resulting aqueous suspension was extracted with $CHCl_3$ (3 x 250 mL). The organics were combined, dried with Na_2SO_4 and filtered before removal of the solvent under reduced pressure. The resulting crude yellow oil was purified by FCC (1:1 pentane:EtOAc to 100% EtOAc, eluent cooled to 0 °C) to yield the title compound as a colourless oil (423 mg, 52%). **IR** (thin film) ν_{max}/cm^{-1} 3386 (O-H), 2953, 1619, 1514, 1248; **^{1}H NMR** (400 MHz, $CDCl_3$) δ 6.79 – 6.68 (m, 3H), 5.82 (p, *J* = 1.3 Hz, 1H), 5.71 (app. q, *J*

= 1.9 Hz, 1H), 3.81 (app. d, *J* = 2.1 Hz, 6H), 3.52 (d, *J* = 2.9 Hz, 2H), 3.49 – 3.36 (m, 2H), 3.25 (dt, *J* = 15.2, 2.4 Hz, 1H), 2.38 (ddd, *J* = 12.6, 9.0, 6.5 Hz, 1H), 2.07 (dt, *J* = 15.3, 1.5 Hz, 1H), 1.83 (tdd, *J* = 11.8, 6.6, 5.2 Hz, 1H), 1.70 (ddtd, *J* = 12.2, 8.9, 7.6, 6.3 Hz, 1H), 1.41 (dt, *J* = 12.1, 5.9 Hz, 1H), -0.00 (s, 9H); **^{13}C NMR** (101 MHz, $CDCl_3$) δ 171.9, 151.3, 150.1, 148.5, 144.1, 129.7, 123.3, 114.2, 113.5, 81.5, 78.2, 58.1 (app. d, *J* = 6.8 Hz), 51.4, 47.3, 45.5, 35.8, 25.8, -0.0; **HRMS** (ESI) mass calculated for [M+H]+ ($C_{21}H_{32}O_4NSi$) requires *m/z* 390.2095, found *m/z* 390.2098.

Major side products for this reaction included a base-mediated condensation product and the elimination of the secondary alcohol:

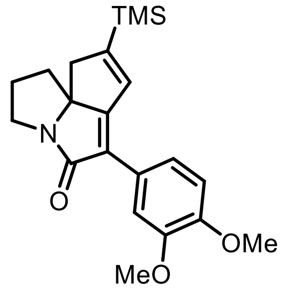

condensation side product

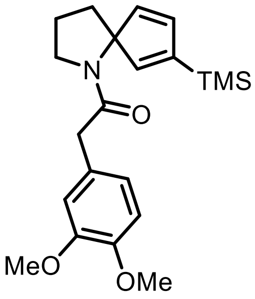

elimination side product

Synthesis of **6**

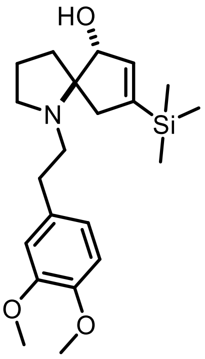

Following a modified literature procedure,[18] to a suspension of $LiAlH_4$ (141 mg, 3.7 mmol) in dry THF (9 mL), was slowly added a THF solution (9 mL) of allylic alcohol **S15** (400 mg,

1.0 mmol). The resulting mixture was heated at 66 °C for 80 minutes. The reaction was cooled to r.t., diluted with Et_2O (20 mL) before slow addition of H_2O in THF (2 mL in 38 mL) followed by addition of 2M NaOH solution (2 mL). This suspension was filtered and the solvents were removed under reduced pressure. The resulting crude yellow oil was purified by FCC (95:5:1 $CHCl_3$:MeOH:NH_3(aq)) to yield the title compound as a colourless oil (257 mg, 68%)

IR (thin film) ν_{max}/cm^{-1} 3362 (O-H), 2951, 1591, 1515, 1261; **^{1}H NMR** (400 MHz, $CDCl_3$) δ 6.78 (d, *J* = 8.7 Hz, 1H), 6.75 – 6.68 (m, 2H), 5.91 (q, *J* = 1.9 Hz, 1H), 4.65 (q, *J* = 1.6 Hz, 1H), 3.85 (app. d, *J* = 4.6 Hz, 6H), 3.12 (td, *J* = 8.7, 5.8 Hz, 1H), 2.78 – 2.63 (m, 4H), 2.52 – 2.36 (m, 2H), 2.30 – 2.19 (m, 1H), 2.15 (dt, *J* = 16.4, 1.6 Hz, 1H), 1.91 – 1.76 (m, 2H), 1.53 (ddd, *J* = 12.4, 8.3, 5.8 Hz, 1H), 0.05 (s, 9H); **^{13}C NMR** (101 MHz, $CDCl_3$) δ 151.0, 149.6, 149.2, 143.6, 135.5, 122.7, 114.3, 113.4, 81.4, 78.7, 58.1 (app. d, *J* = 2.8 Hz) 53.9 (app d, *J* = 12.5 Hz), 41.5, 37.8, 34.3, 23.1, -0.0; **HRMS** (ESI) mass calculated for [M+H]+ ($C_{21}H_{34}O_3NSi$) requires *m/z* 376.2302, found *m/z* 376.2304.

<u>Investigation of Intramolecular Friedel-Crafts</u>

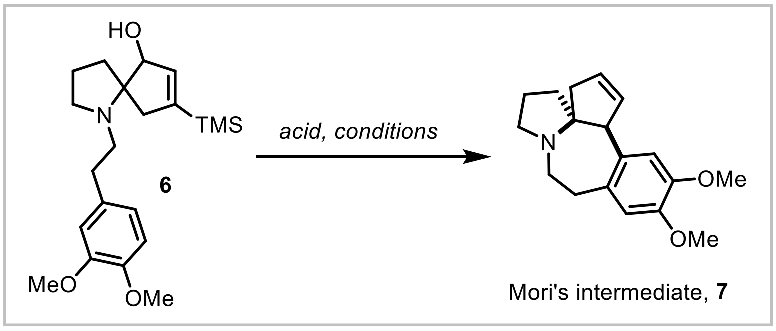

entry	acid	solvent	NMR yield	observations
1[a]	PPA	neat	37%	-
2[b]	Eaton's reagent	neat	64%	-
3[c]	Eaton's reagent	neat	58%	-
4[d]	H_2SO_4	AcOH	60%	-
5[d]	TsOH	HFIP	-	m/z 358 dominant
6[c]	HI	PhMe	-	m/z 288 dominant
7[e]	TsOH then HI	HFIP then PhMe	-	m/z 288 dominant
8[c]	TfOH	neat	-	m/z 272 dominant

Table S4. Screen of different acids to bring about key Friedel-Crafts reaction. [a] 65 °C for 43 h. [b] r.t. for 4 h. [c] 55 °C for 4-6 h. [d] 55 °C for 3 d. [e] 55 °C for 5 h then solvent swap and r.t. for 2 d.

Synthesis of Mori's intermediate **7**

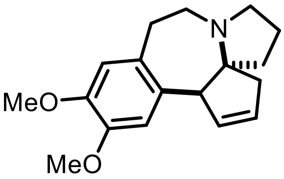

To **6** (120 mg, 0.32 mmol) was added Eaton's Reagent (7.7 wt % P_2O_5 in MsOH, 3.2 mL) and the mixture stirred at r.t. for 5 h. Sat. aq. $NaHCO_3$ was slowly introduced before the mixture was basified to pH 10 with 2M aq. NaOH. The aqueous phase was extracted with CH_2Cl_2 (4 x 50 mL) and the combined organics were dried with Na_2SO_4, filtered and the solvent was removed under reduced pressure. Purification by FCC (95:5:1 EtOAc:MeOH:NH_3 (35% aq)) yielded the title compound as a colourless oil (67 mg, 73%). **IR** (thin film) ν_{max}/cm^{-1} 2926 (C-H), 1684, 1515, 1220, 1126; **^{1}H NMR** (400 MHz, $CDCl_3$) δ 6.68 (s, 1H), 6.62 (s, 1H), 5.79 (dq, *J* = 5.2, 2.5 Hz, 1H), 5.54 (dq, *J* = 6.0, 2.3 Hz, 1H), 3.89 (dq, *J* = 5.2, 2.4 Hz, 1H), 3.87 (s, 3H), 3.84 (s, 3H), 3.20 (ddd, *J* = 14.0, 12.3, 7.4 Hz, 1H), 3.08 (td, *J* = 9.0, 4.6 Hz, 1H), 2.94 (td, *J* = 11.9, 6.8 Hz, 1H), 2.76 (dq, *J* = 17.8, 2.6 Hz, 1H), 2.57 (dd, *J* = 11.4, 7.4 Hz, 1H), 2.47 – 2.31 (m,

2H), 2.05 – 1.93 (m, 3H), 1.74 (tddd, *J* = 12.6, 10.8, 8.6, 4.6 Hz, 2H). **^{13}C NMR** (101 MHz, $CDCl_3$) δ 147.6, 147.2, 132.5, 131.1, 130.9, 128.5, 114.1, 113.1, 68.1, 62.3, 56.1, 56.0, 53.7, 49.1, 43.4, 34.7, 30.4, 19.9. **HRMS** (ESI) mass calculated for [M+H]+ ($C_{18}H_{24}NO_2$) requires *m/z* 286.1802, found *m/z* 286.1801. Data were consistent with the literature.[19]

10. Challenging Substrates and Unsuccessful Intermediates

Despite the numerous successful applications of this method and the significant broadening of synthetic access to this chemical space, a number of additional substrates failed at various points along the sequence. Scheme S5 details the additional substrates investigated and at which stage along the three-step sequence issues were encountered.

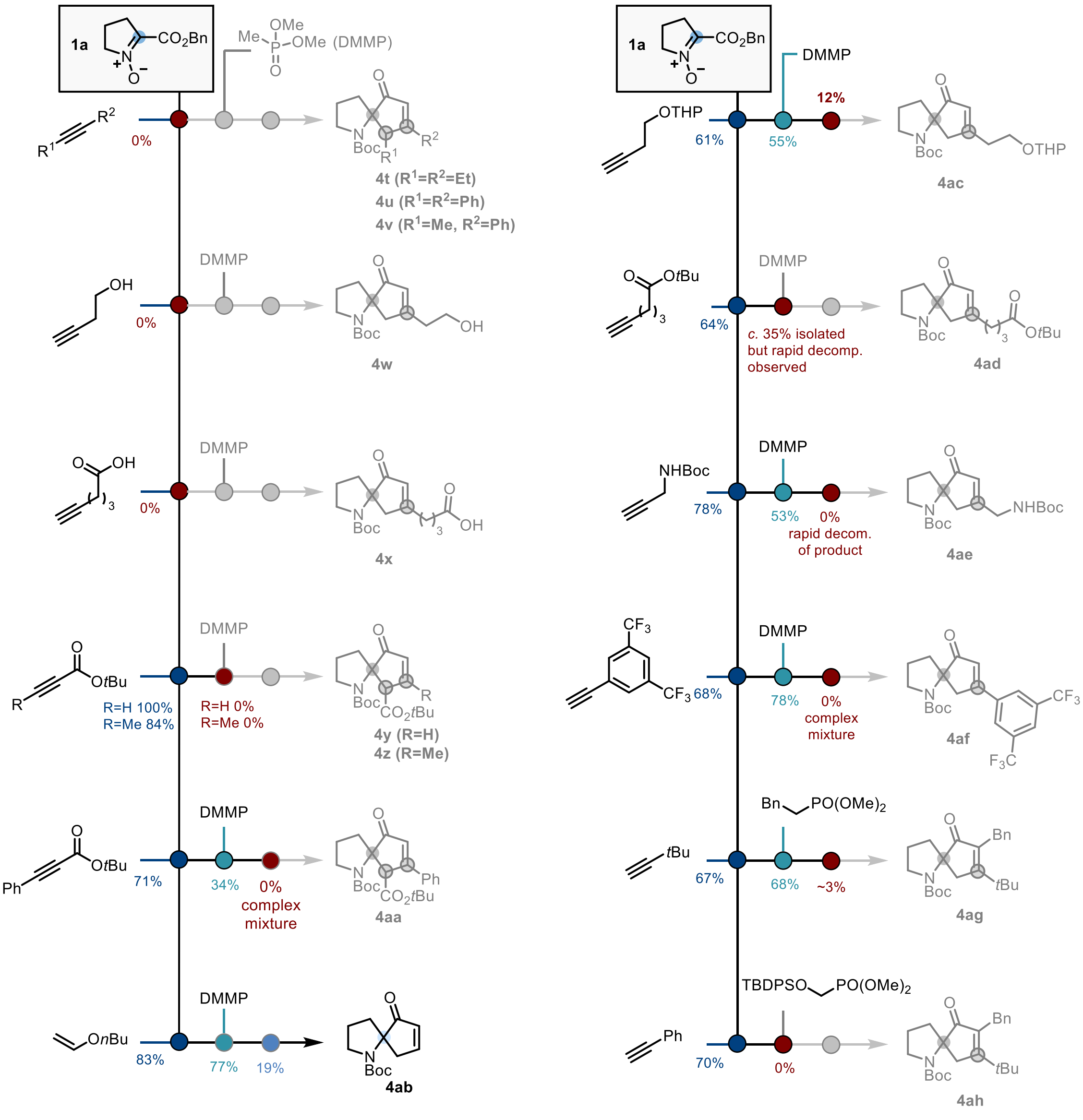

Scheme S6. Challenging substrates

NMR Spectra for Novel Compounds

^{1}H NMR Spectra for Compound 1a

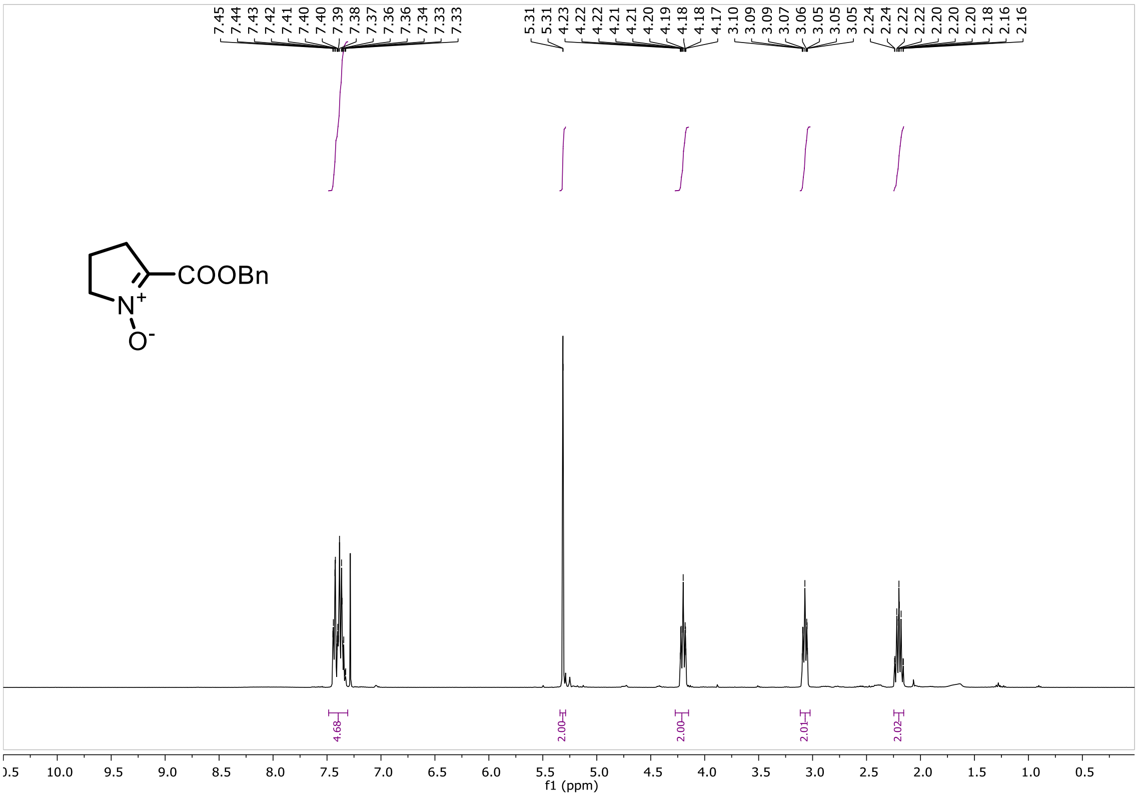

^{13}C NMR Spectra for Compound 1a

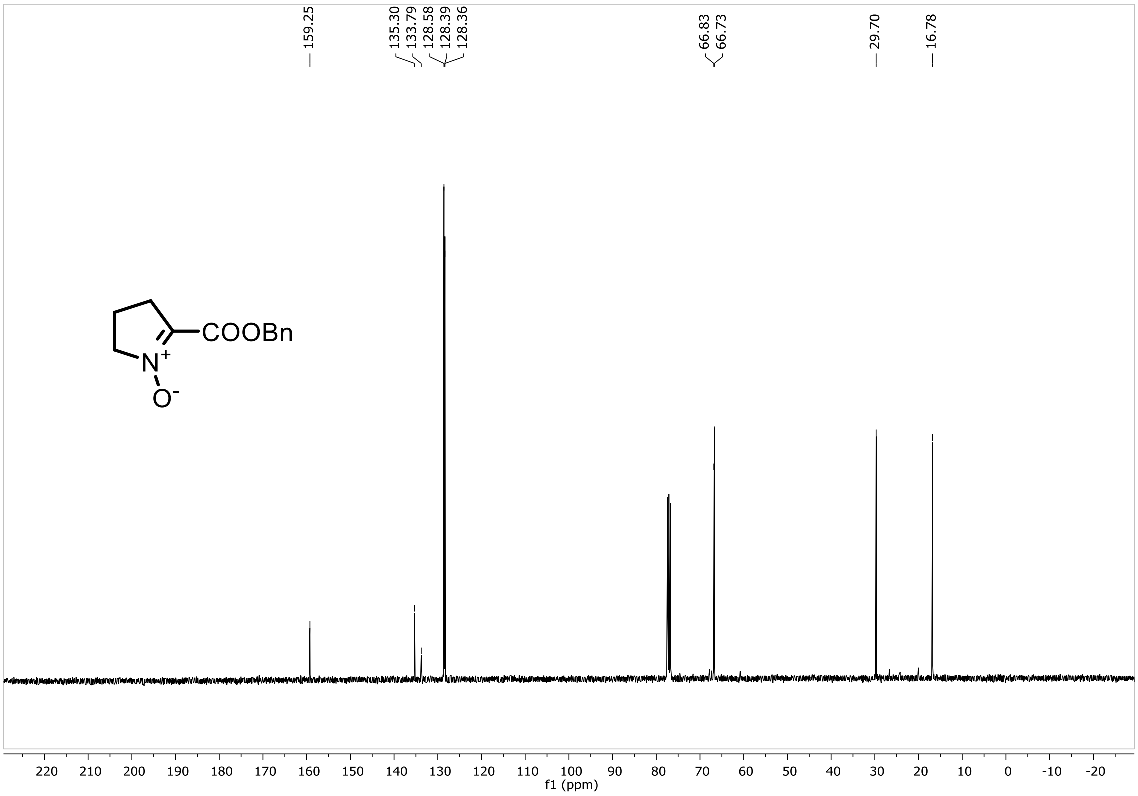

^{1}H NMR Spectra for Compound 1b

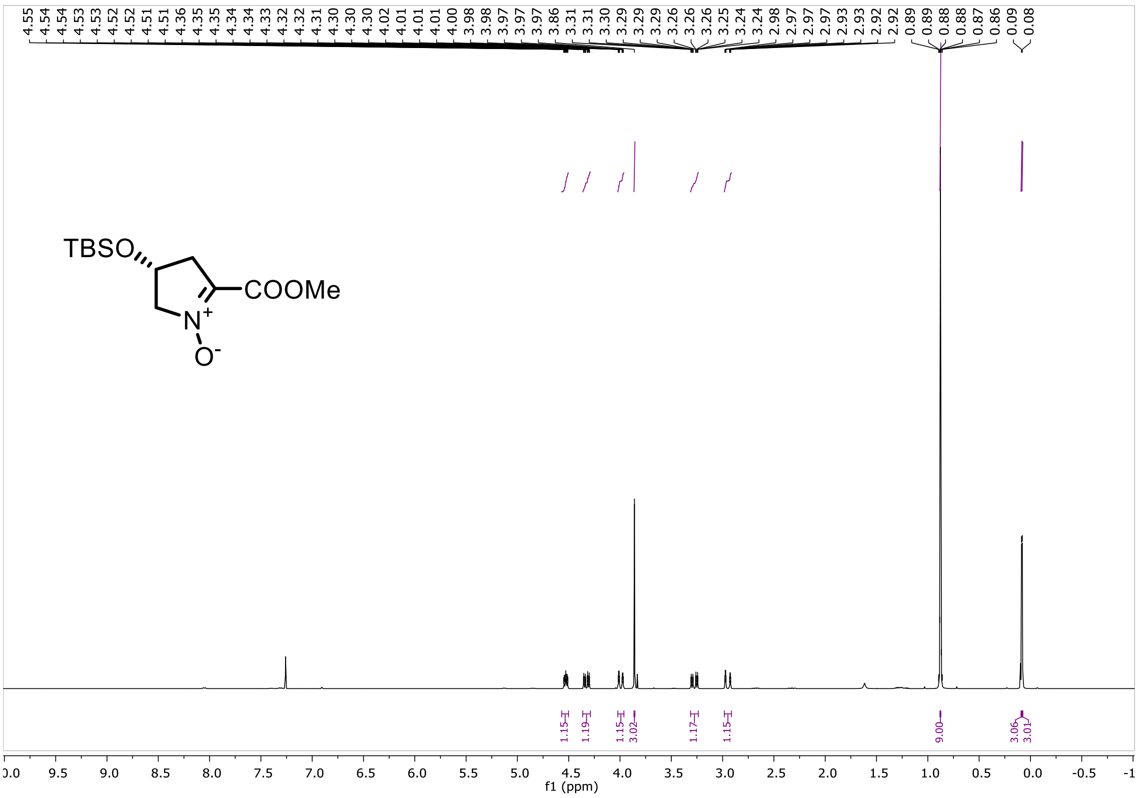

^{13}C NMR Spectra for Compound 1b

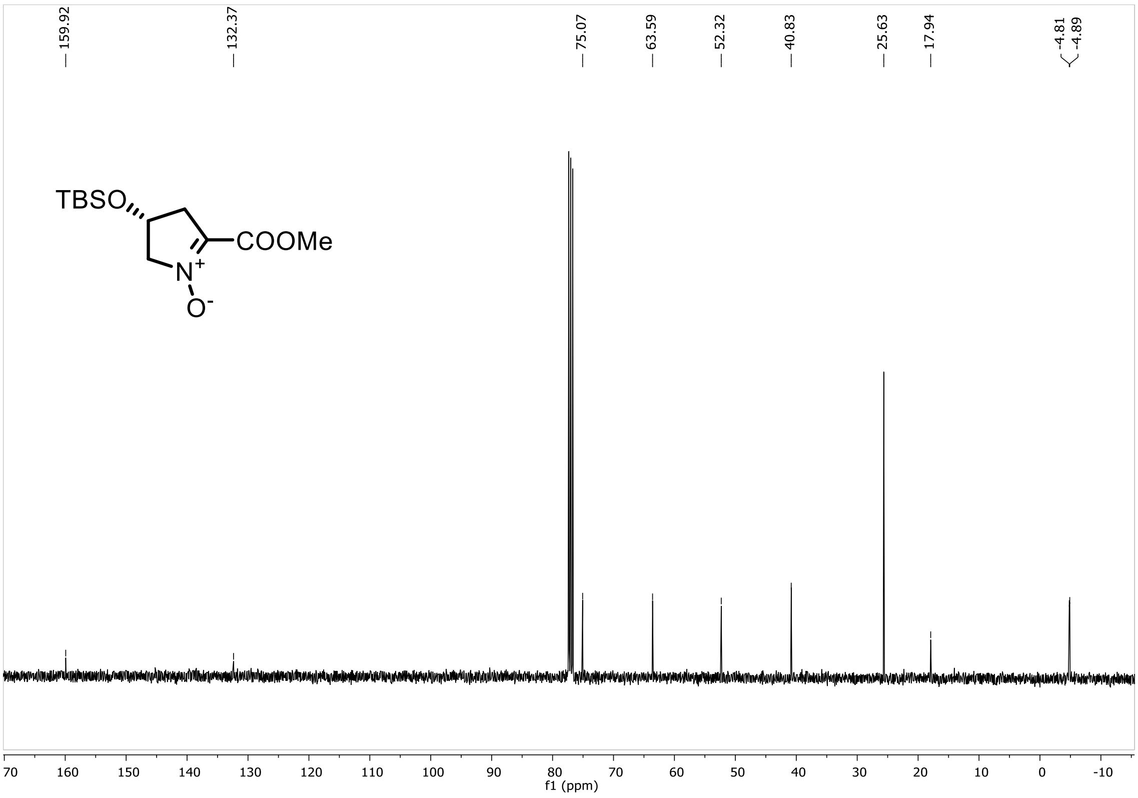

^{1}H NMR Spectra for Compound S6

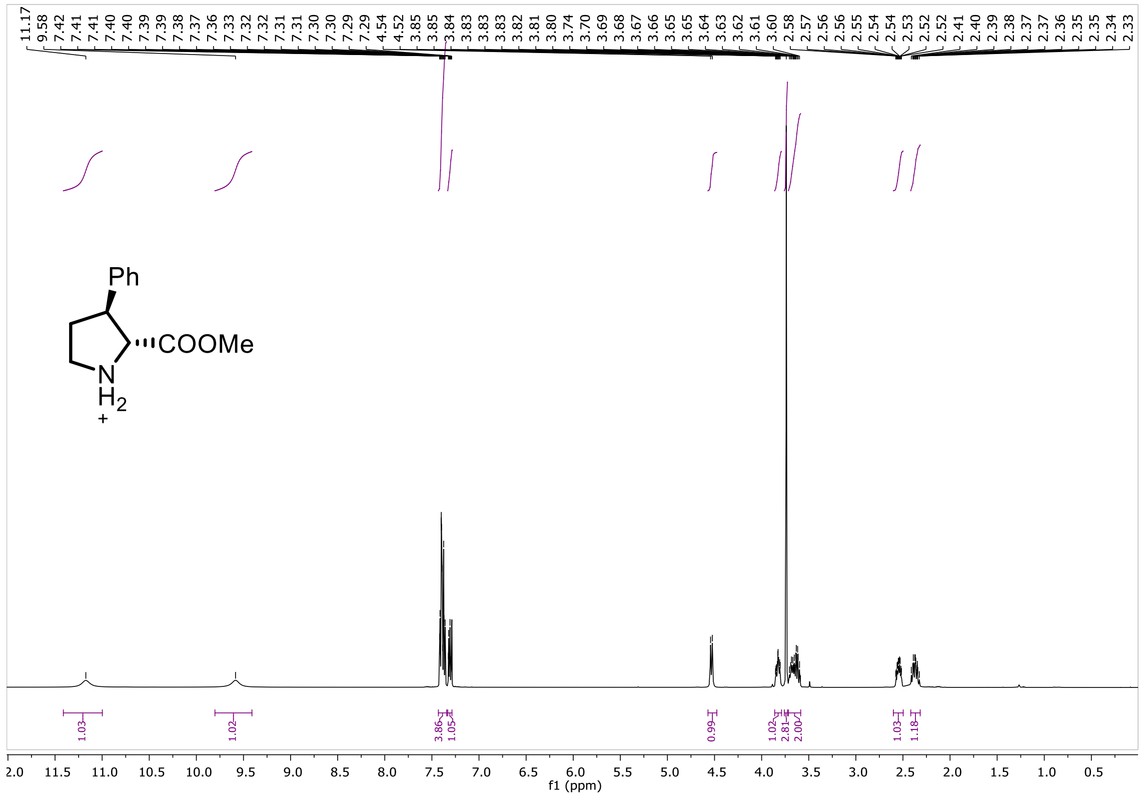

^{13}C NMR Spectra for Compound S6

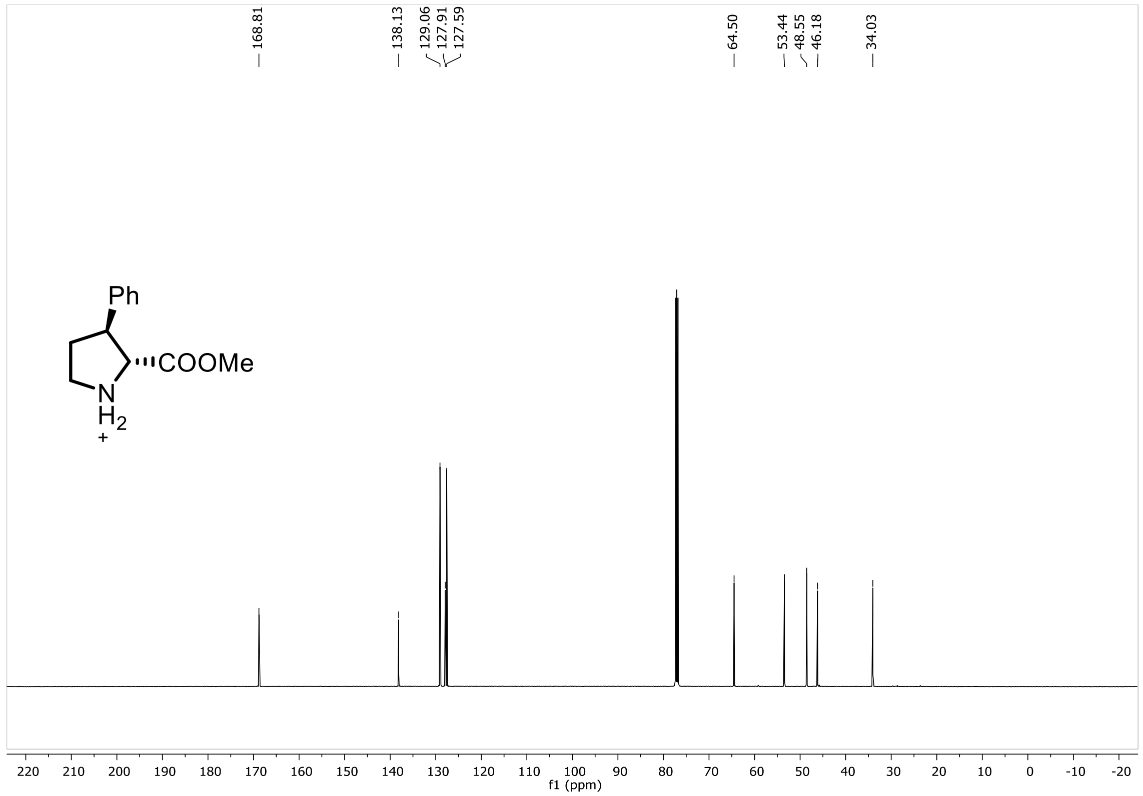

^{1}H NMR Spectra for Compound 1c

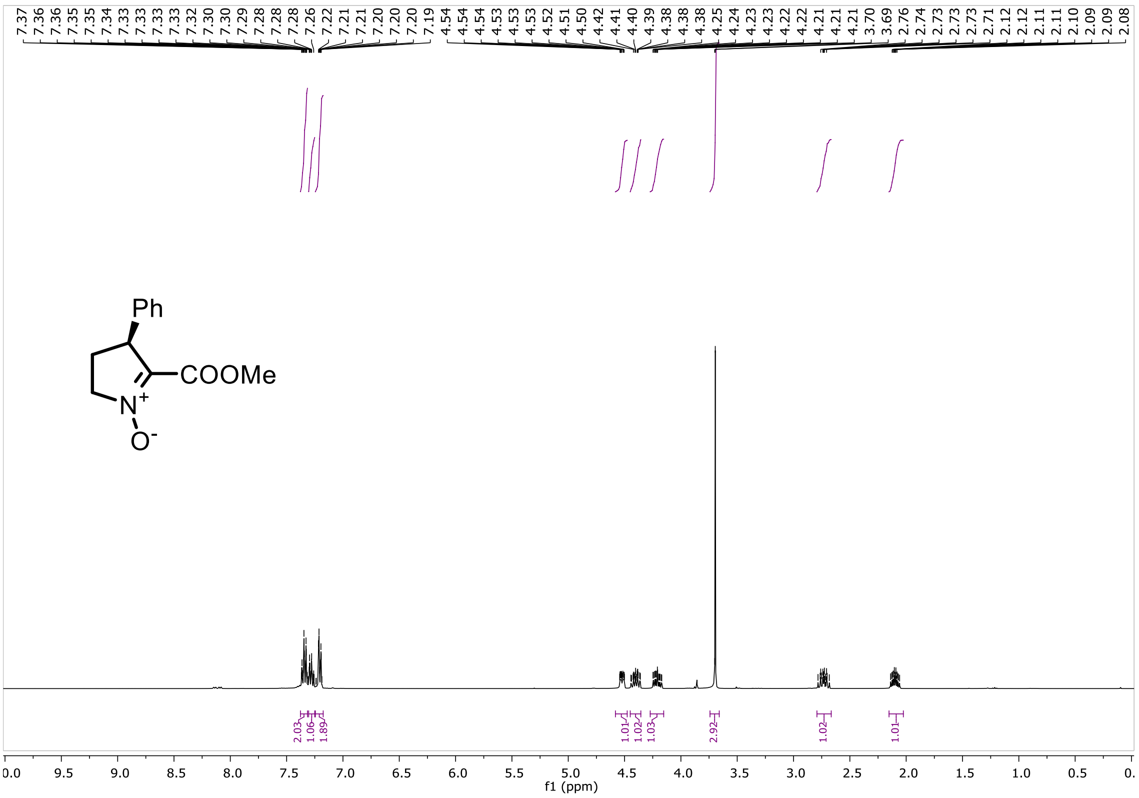

^{13}C NMR Spectra for Compound 1c

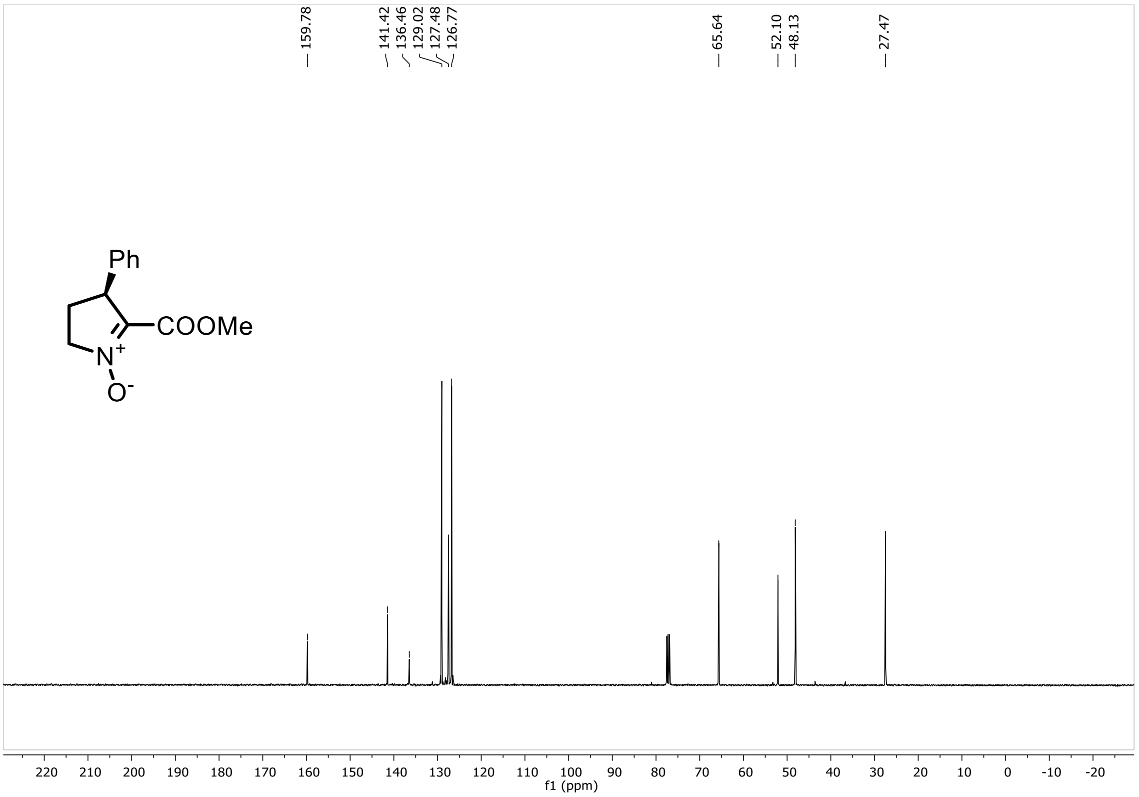

^{1}H NMR Spectra for Compound S10

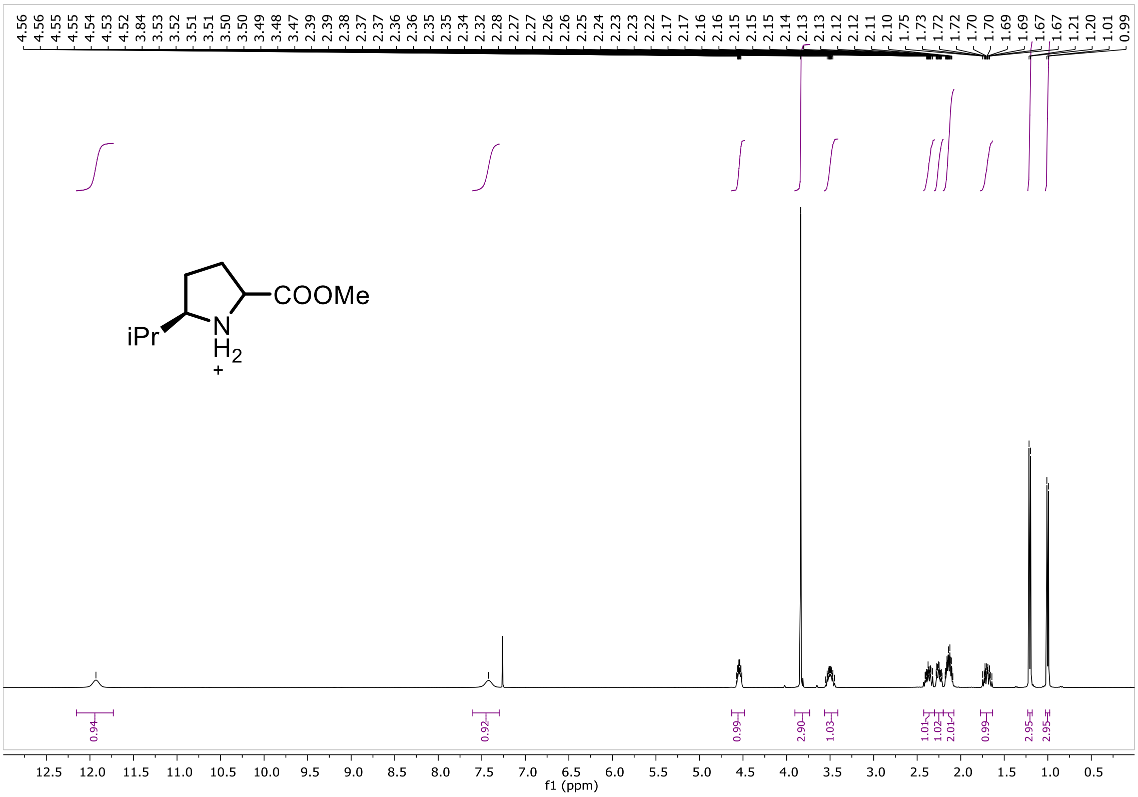

^{13}C NMR Spectra for Compound S10

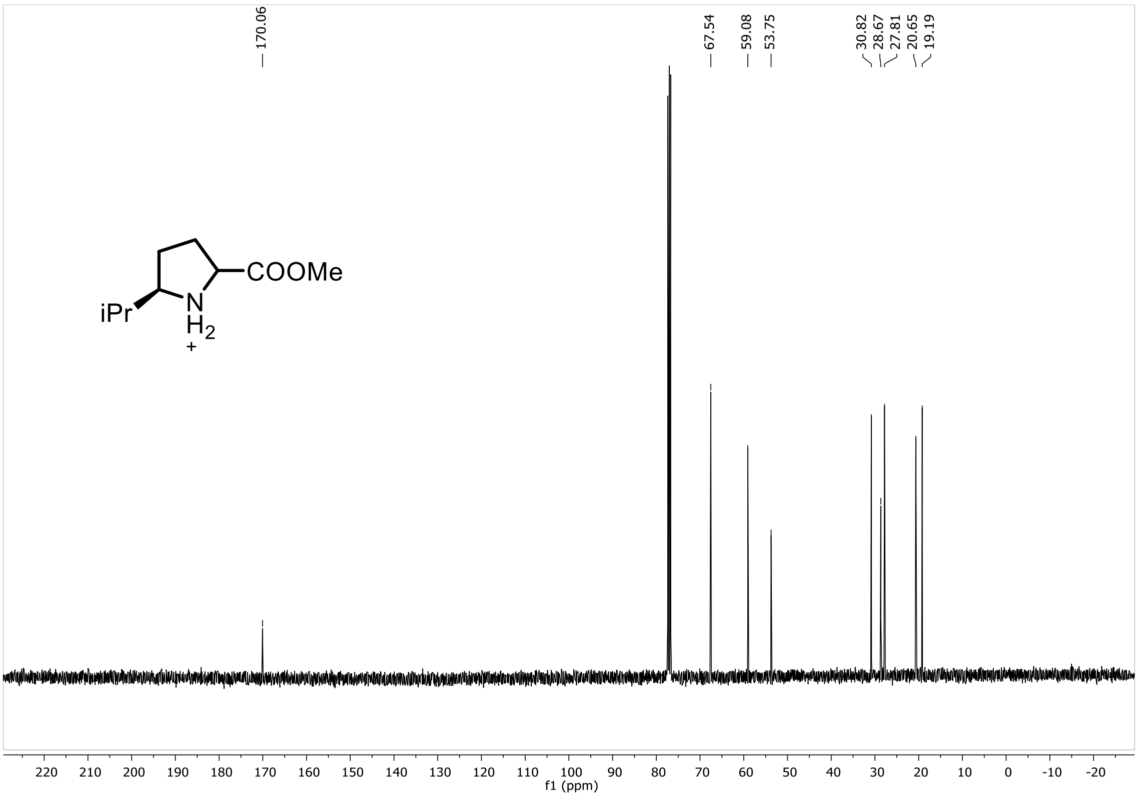

^{1}H NMR Spectra for Compound 1d

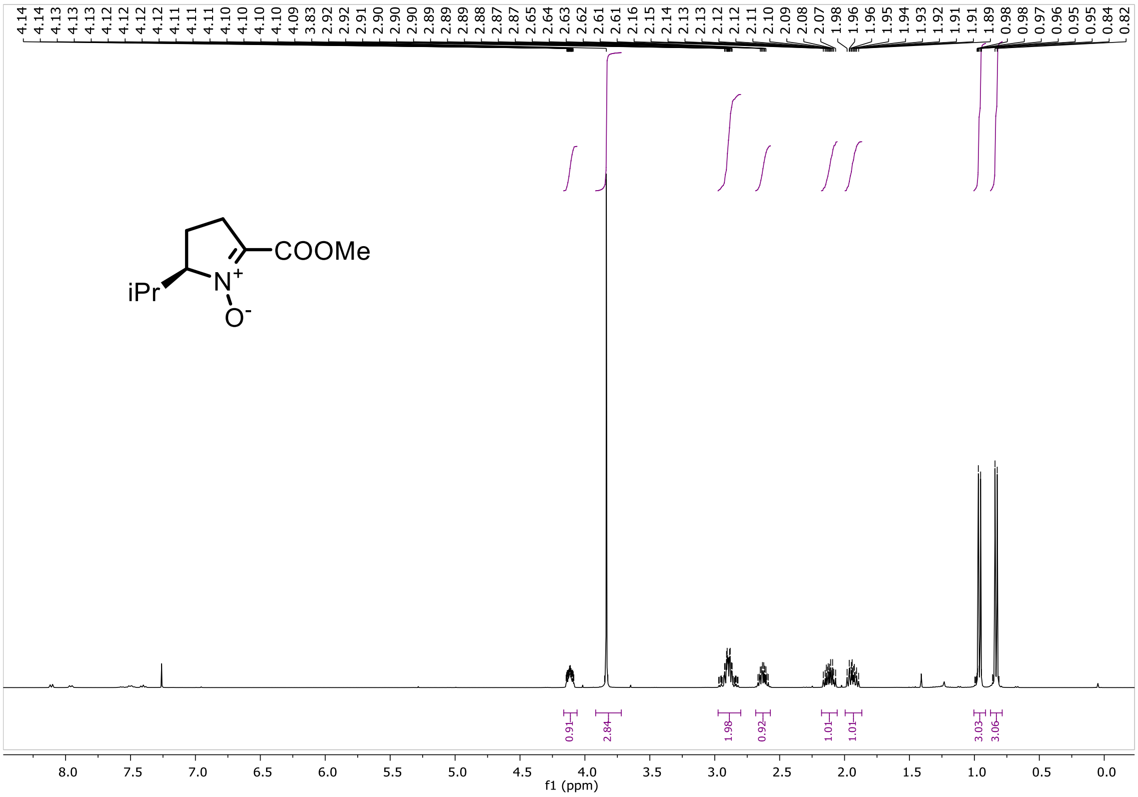

^{13}C NMR Spectra for Compound 1d

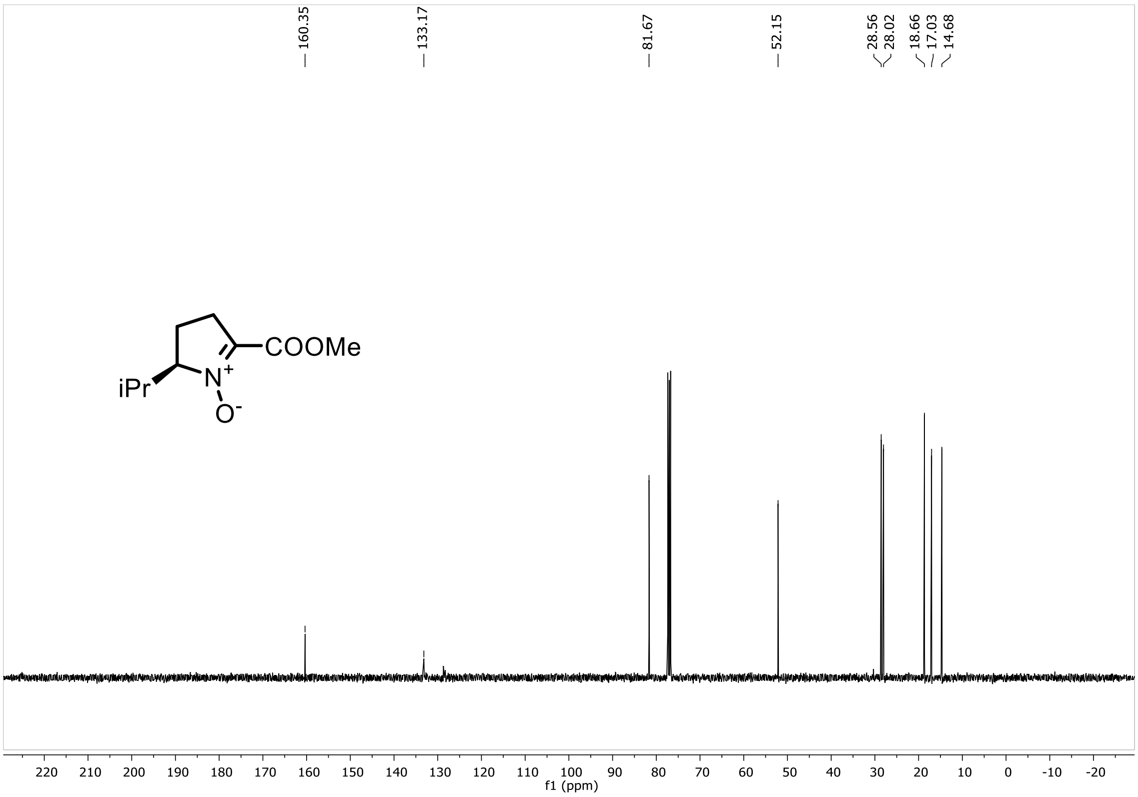

^{1}H NMR Spectra for Compound 2a

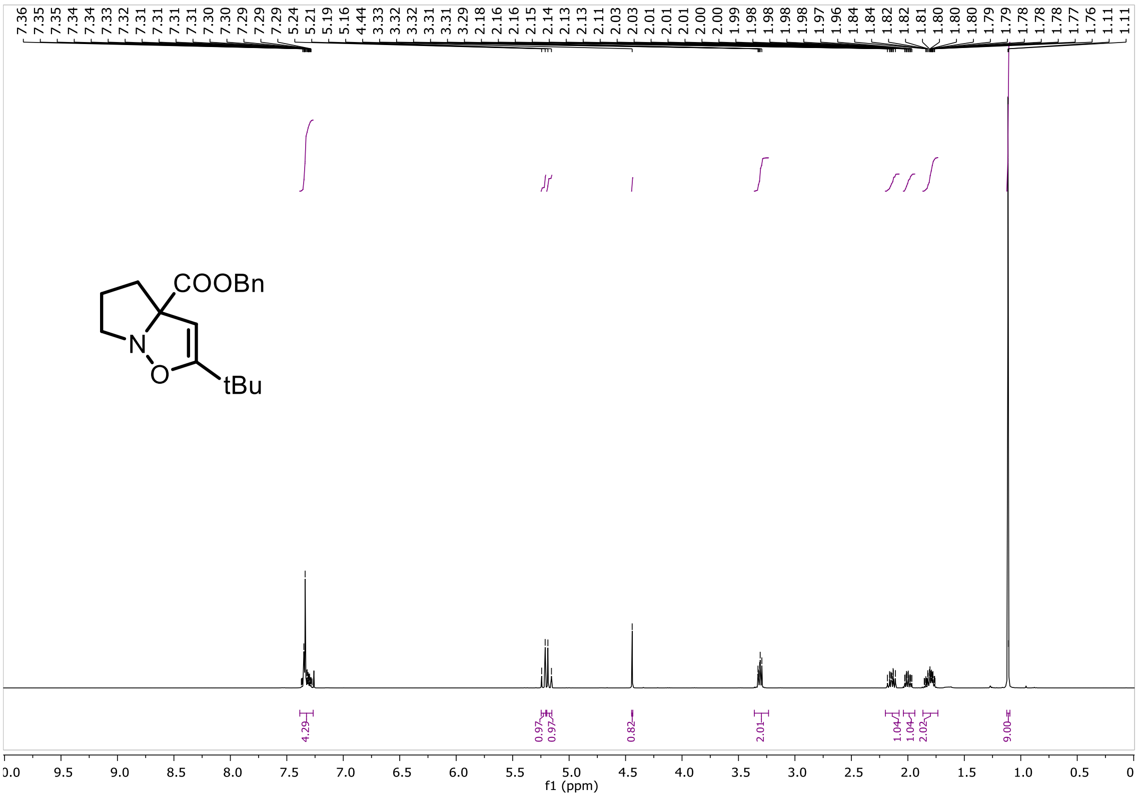

^{13}C NMR Spectra for Compound 2a

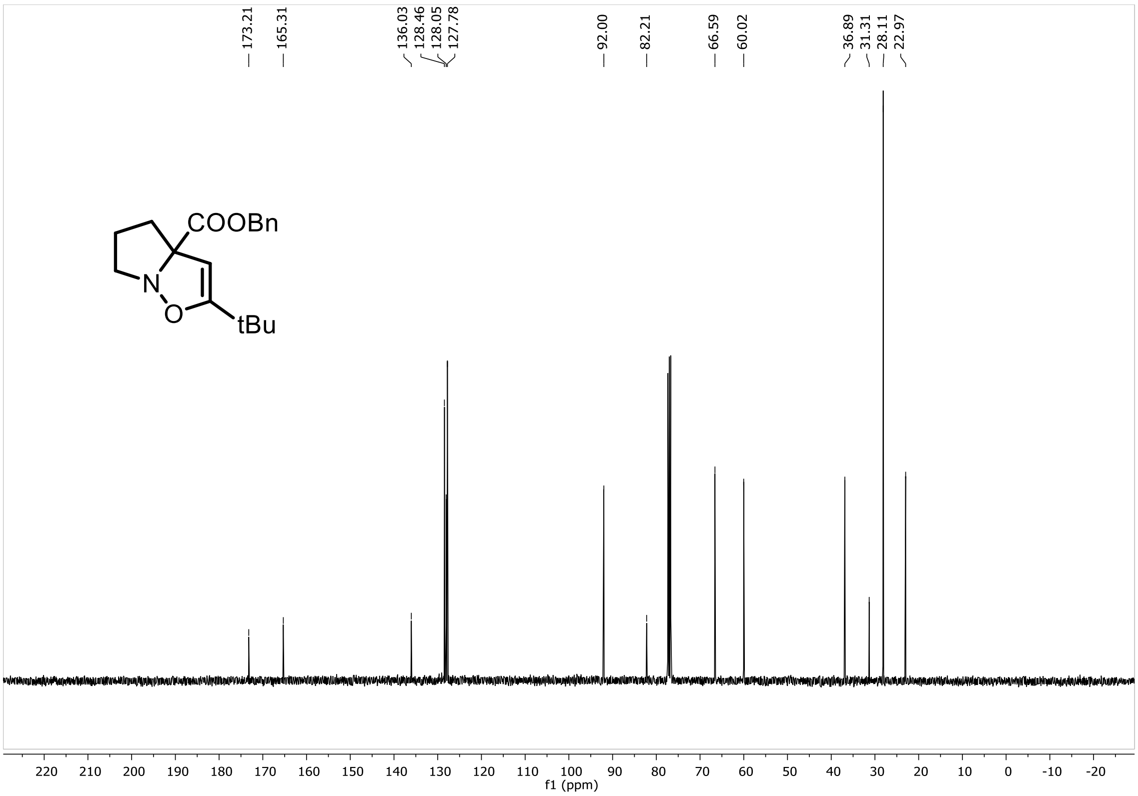

^{1}H NMR Spectra for Compound 2b

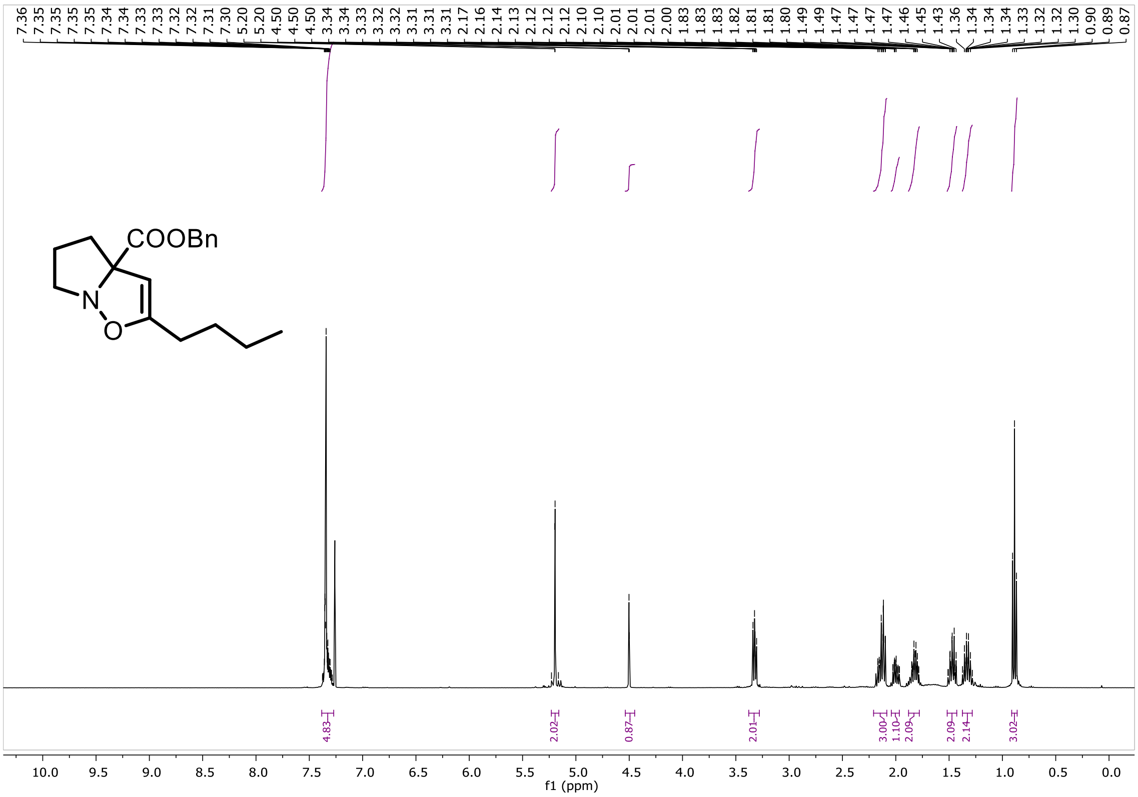

^{13}C NMR Spectra for Compound 2b

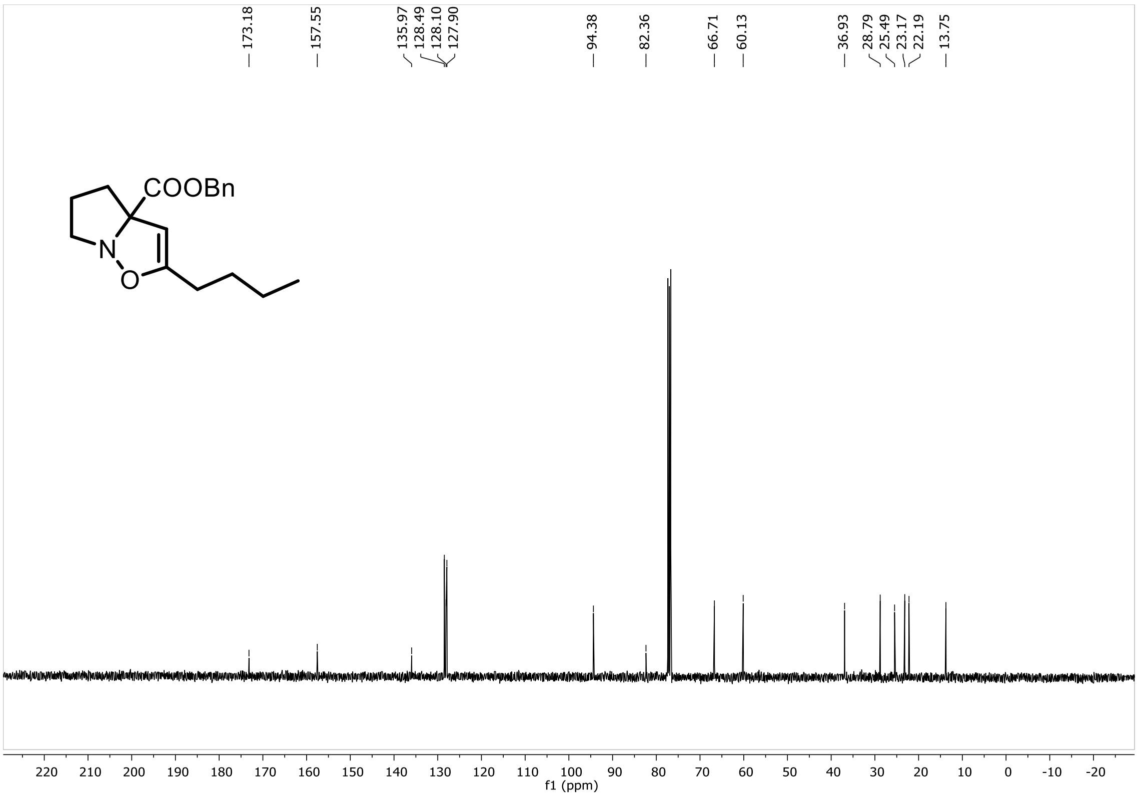

^{1}H NMR Spectra for Compound 2c

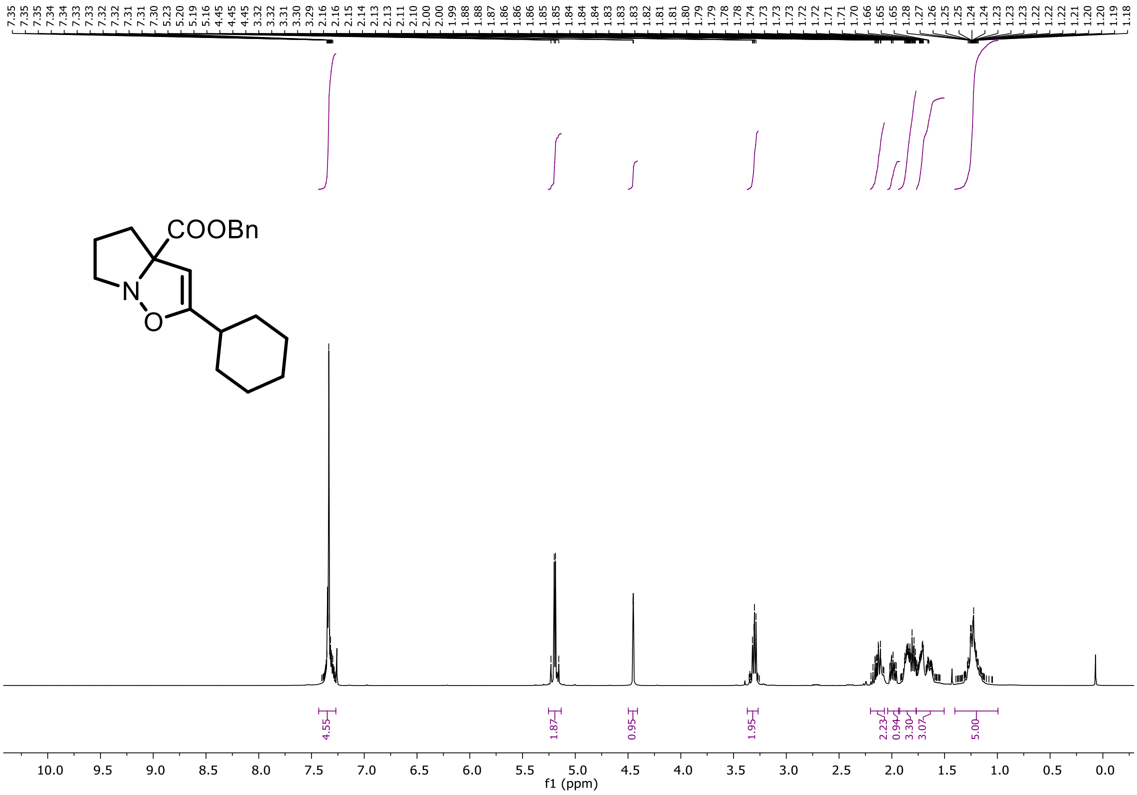

^{13}C NMR Spectra for Compound 2c

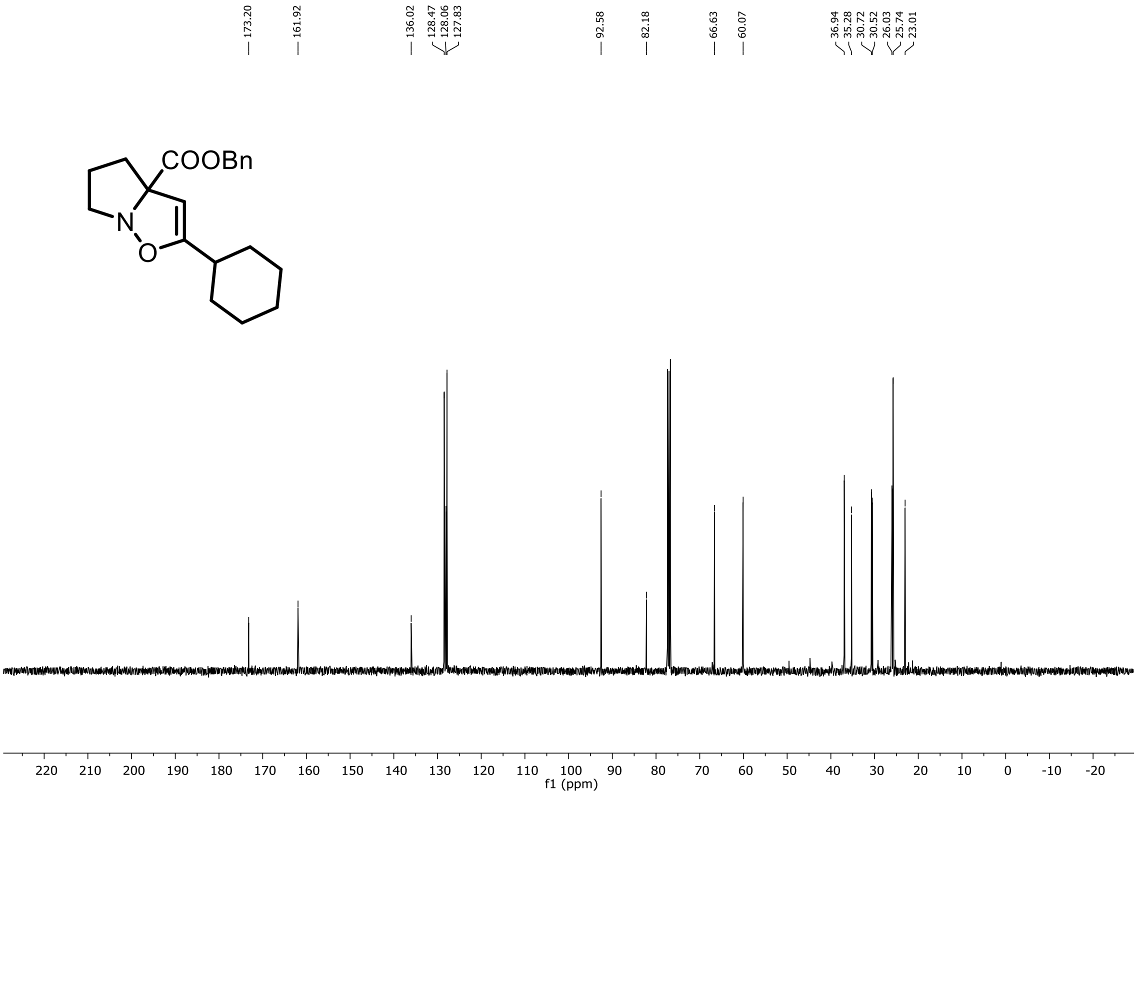

^{1}H NMR Spectra for Compound 2d

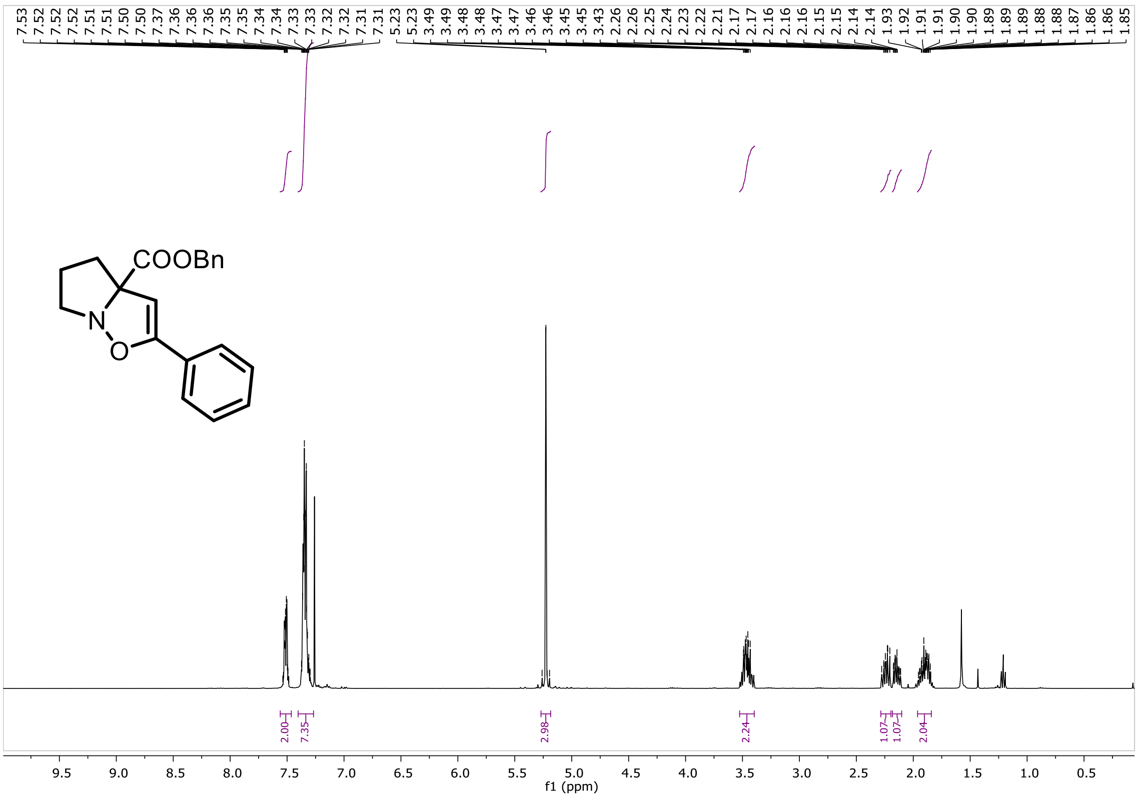

^{13}C NMR Spectra for Compound 2d

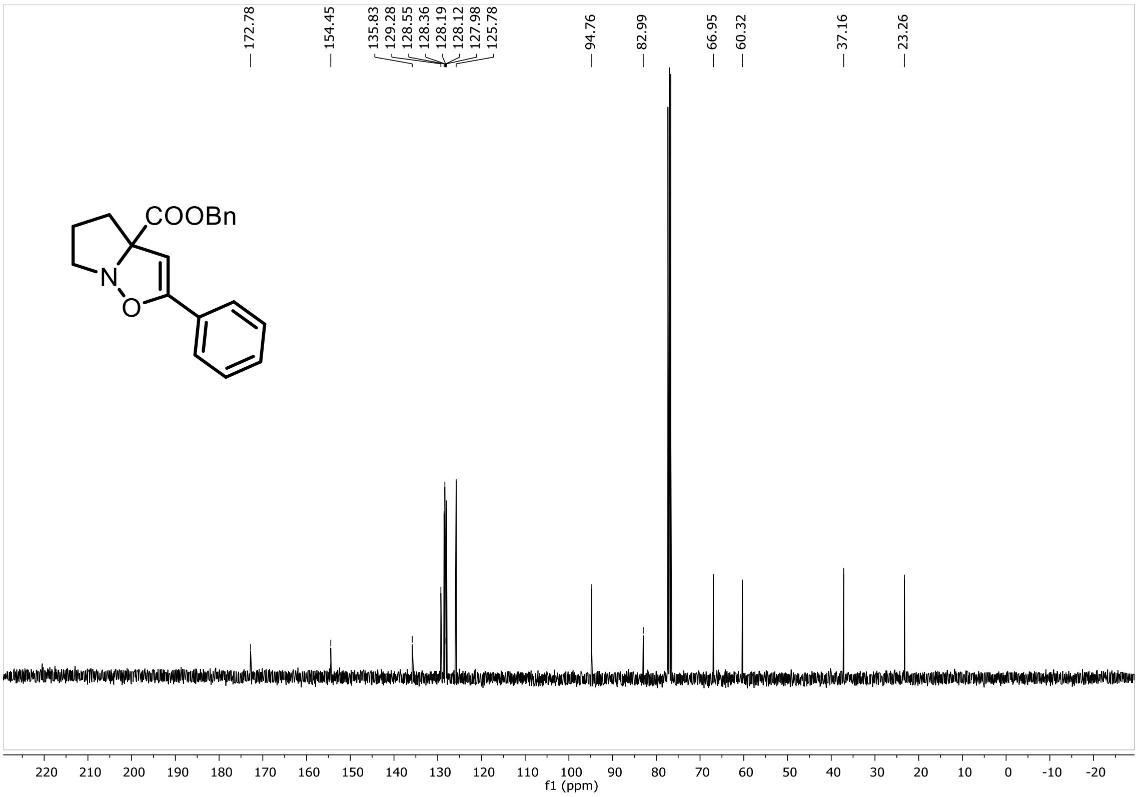

^{1}H NMR Spectra for Compound 2e

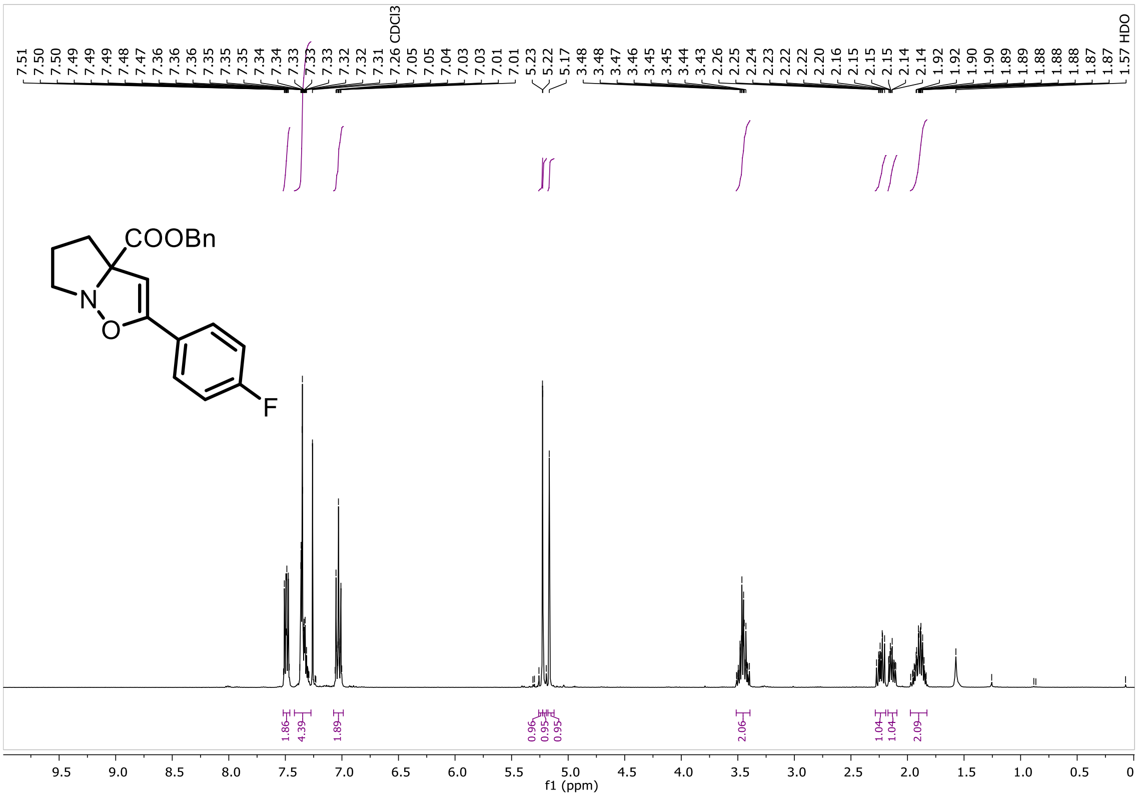

^{13}C NMR Spectra for Compound 2e

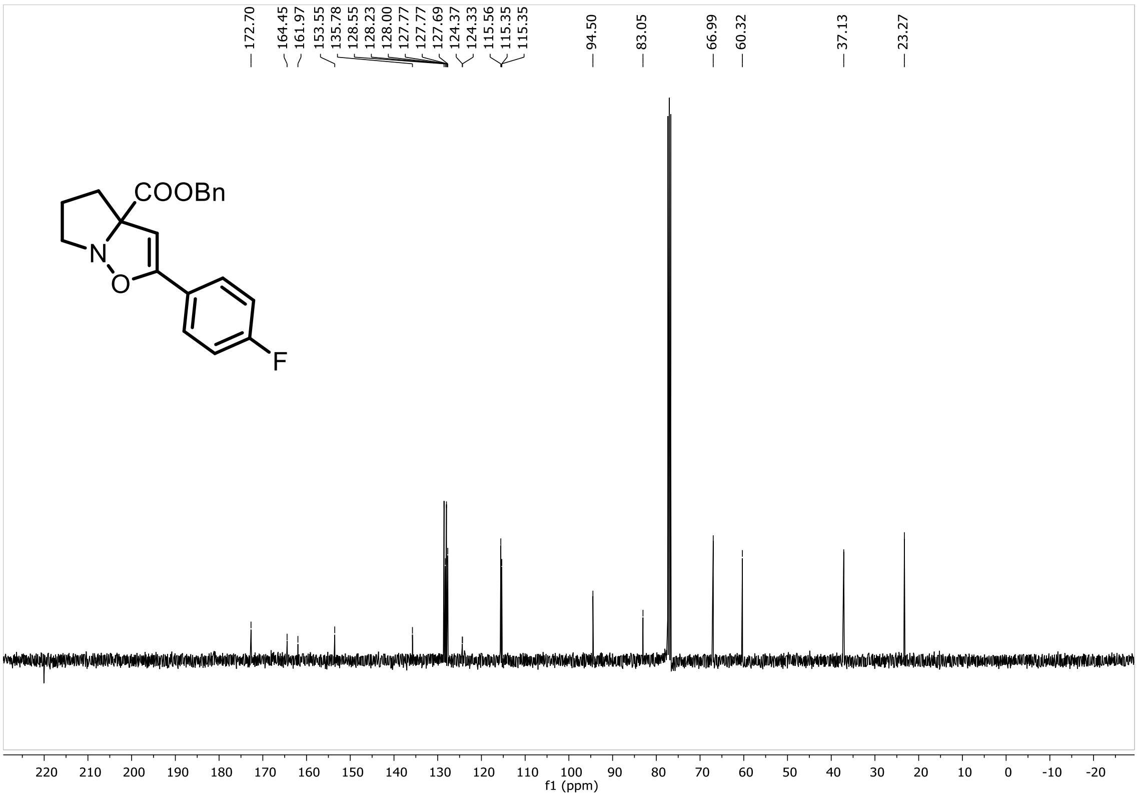

^{19}F NMR Spectra for Compound 2e

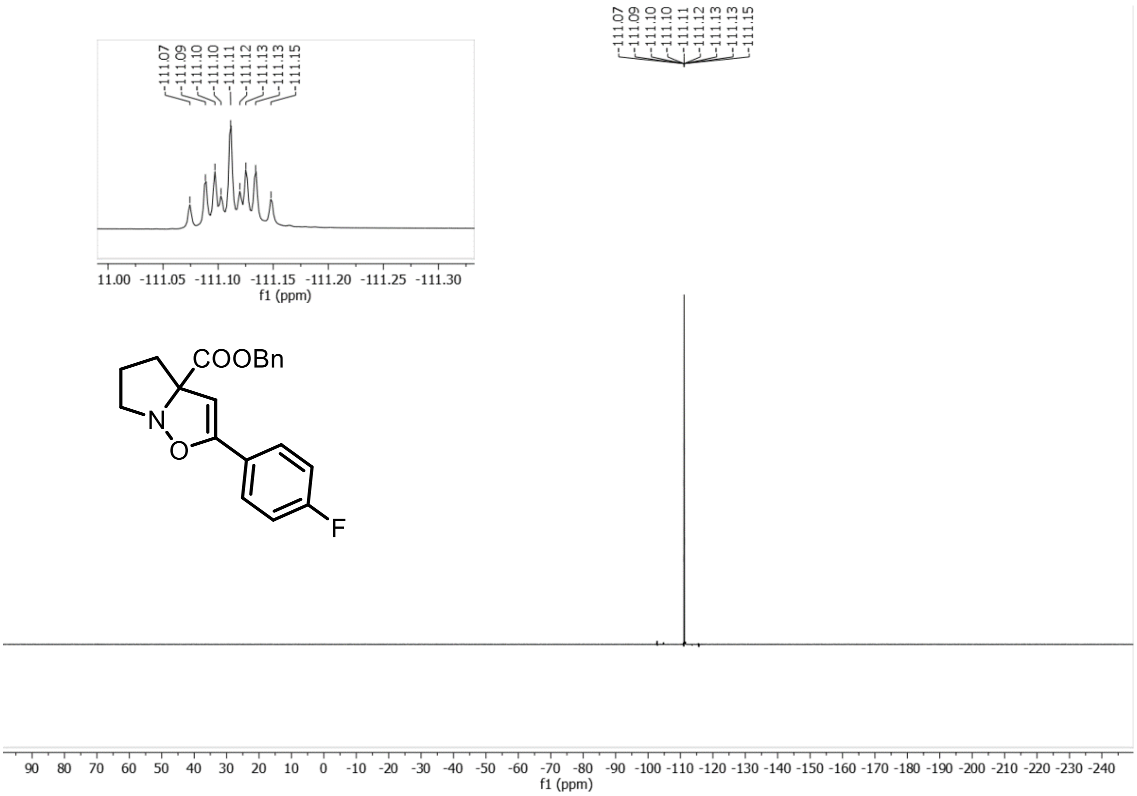

^{1}H NMR Spectra for Compound 2f

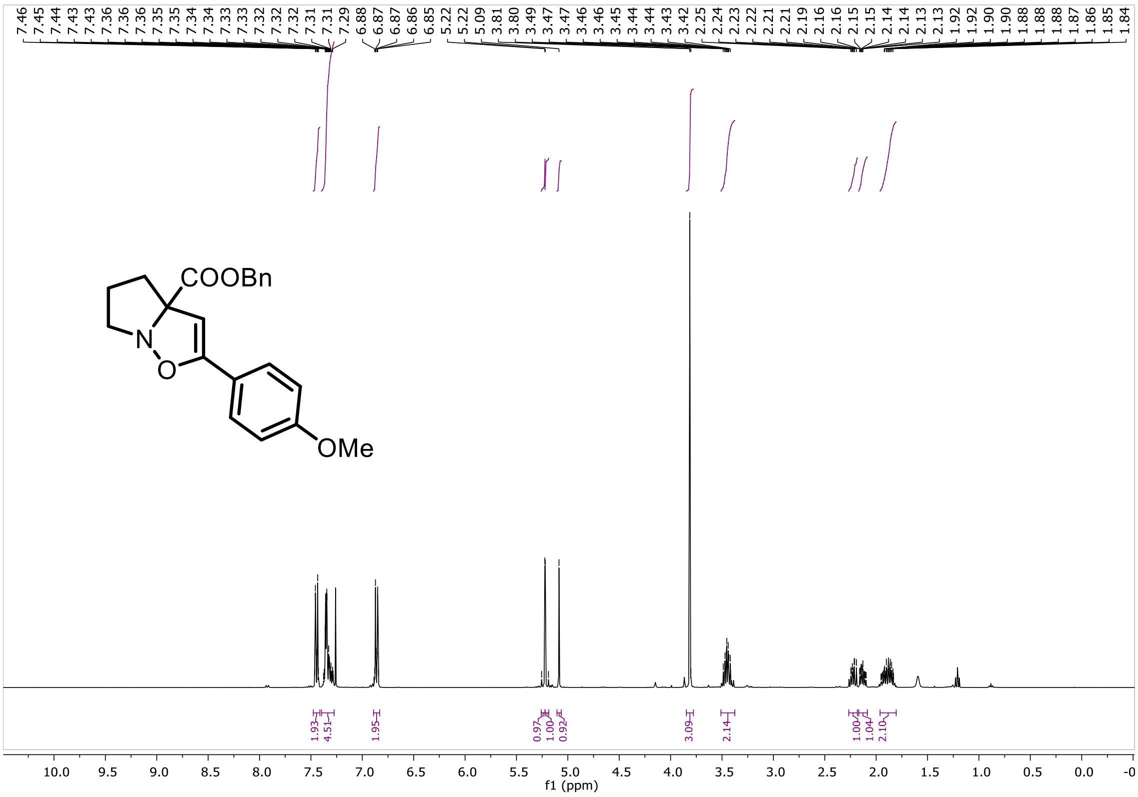

^{13}C NMR Spectra for Compound 2f

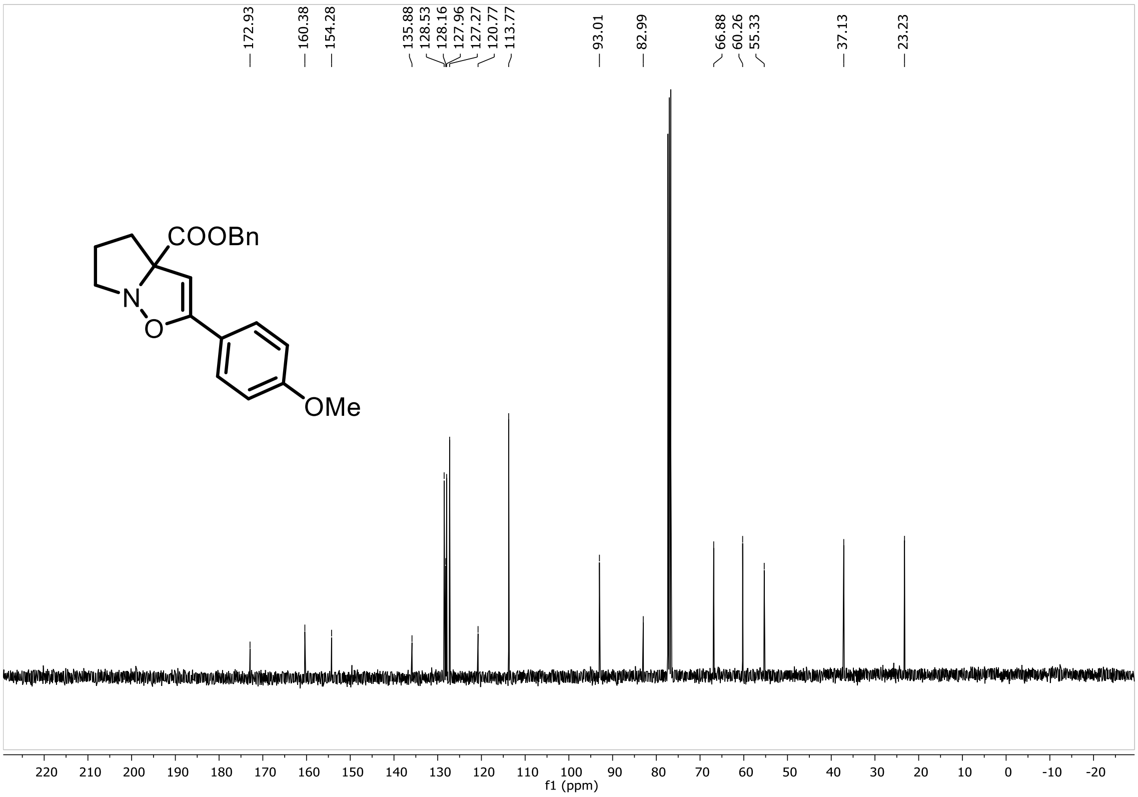

^{1}H NMR Spectra for Compound 2g

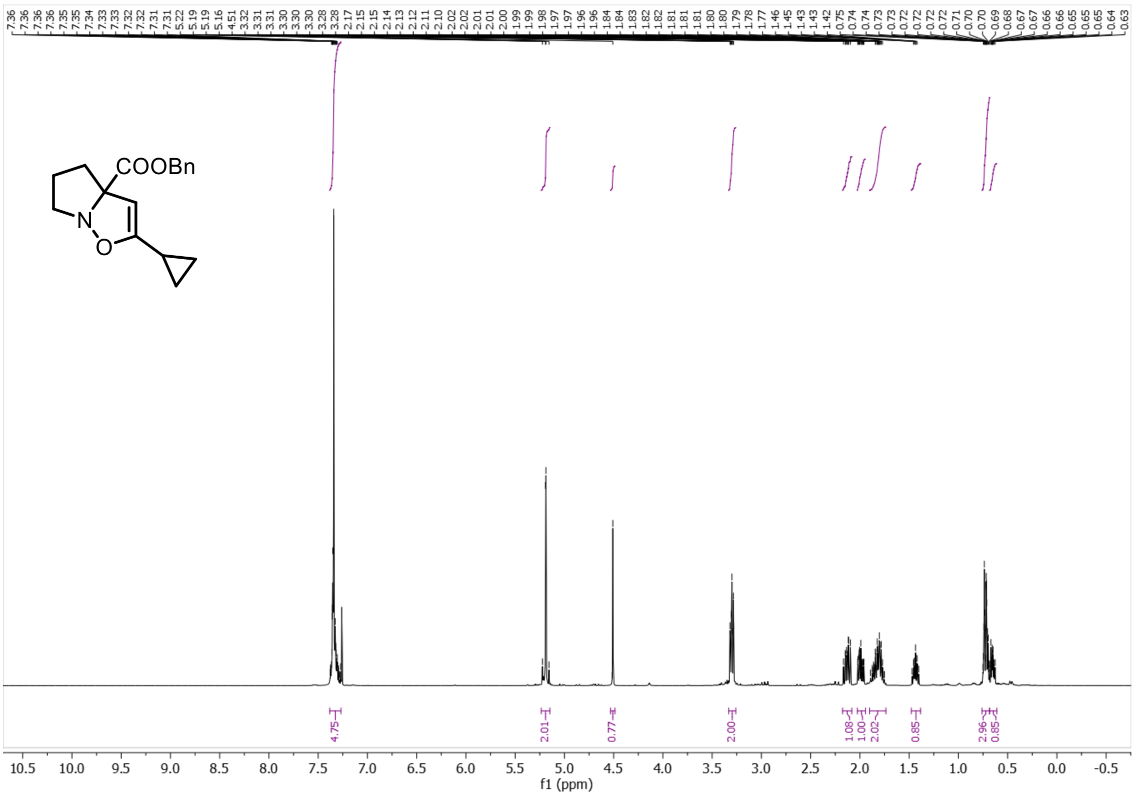

^{13}C NMR Spectra for Compound 2g

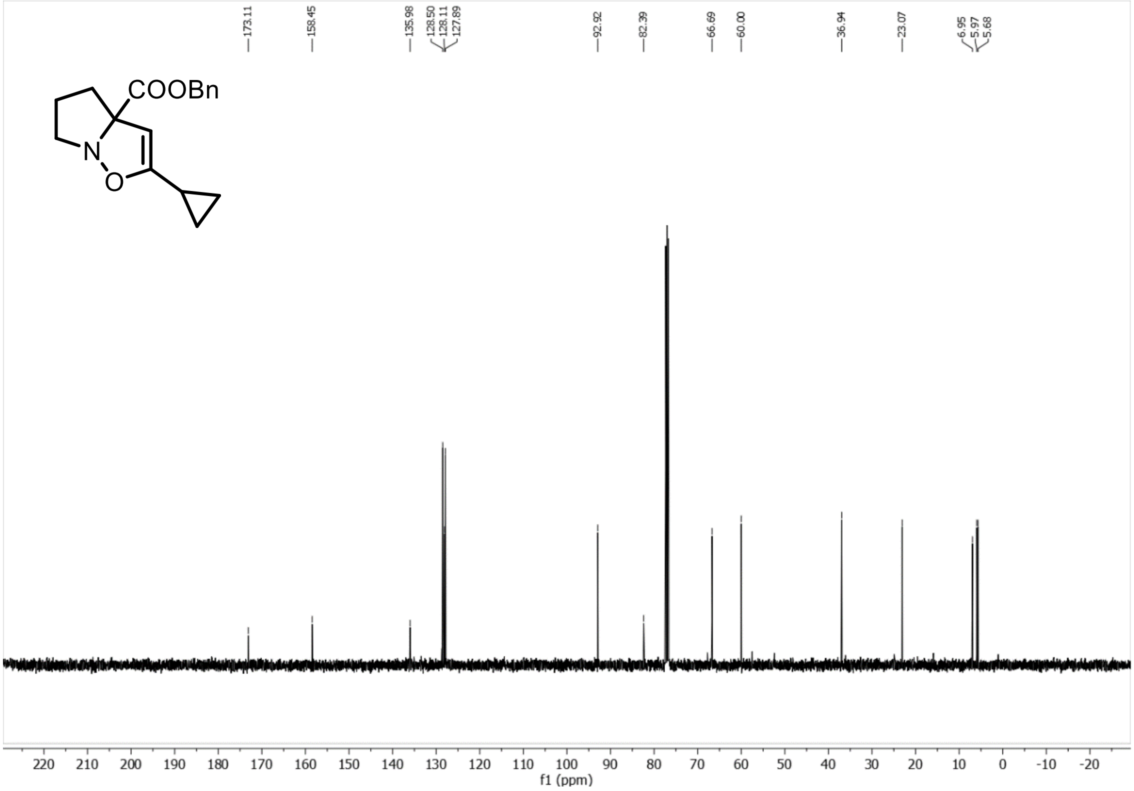

^{1}H NMR Spectra for Compound 2h

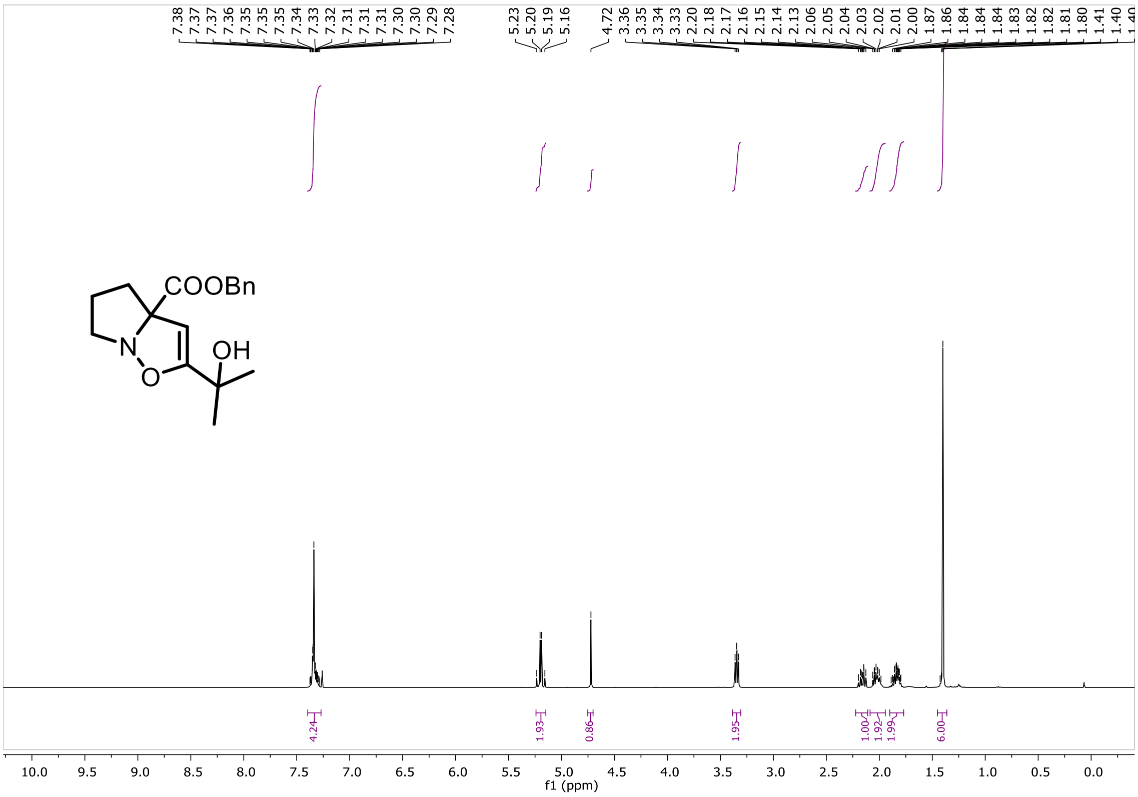

^{13}C NMR Spectra for Compound 2h

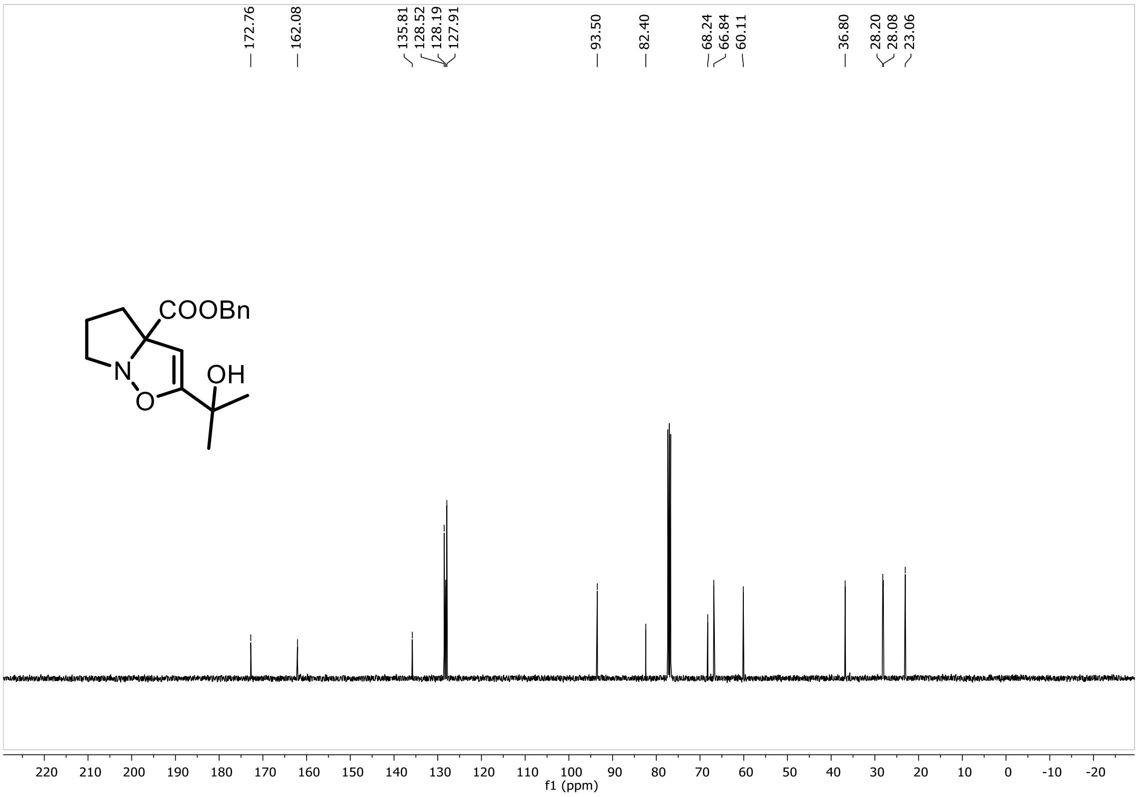

^{1}H NMR Spectra for Compound 2i major

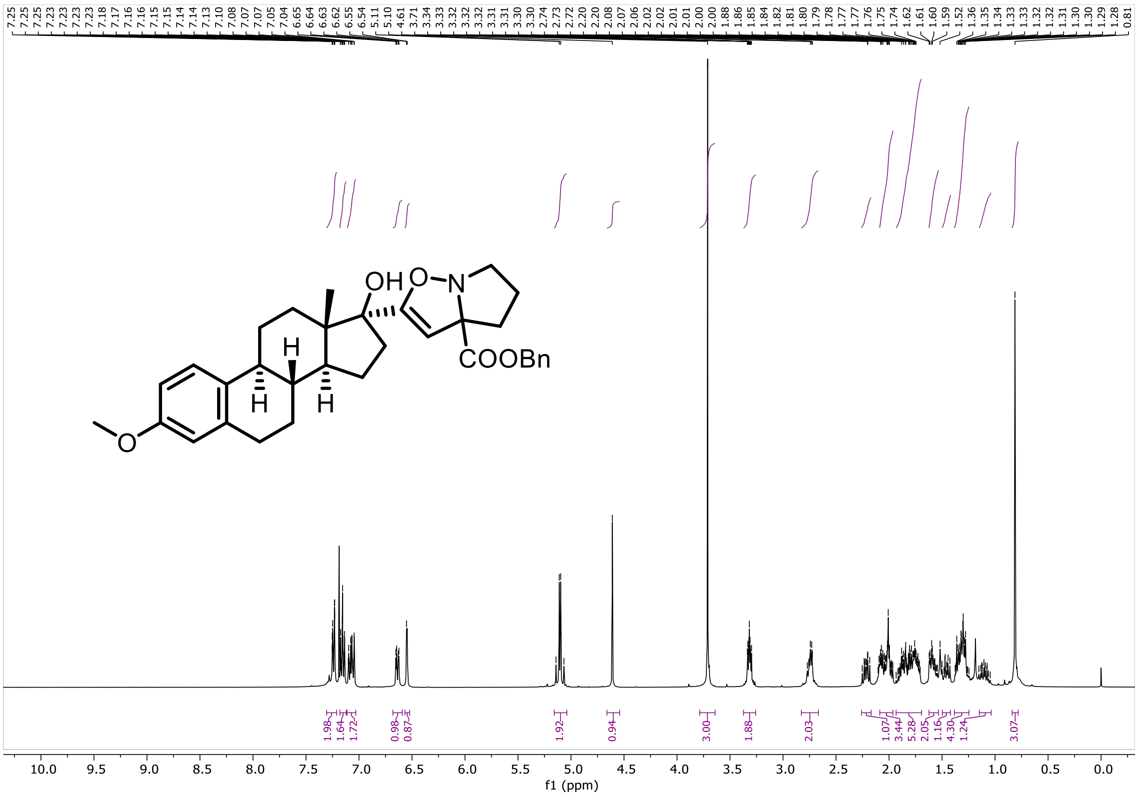

^{13}C NMR Spectra for Compound 2i major

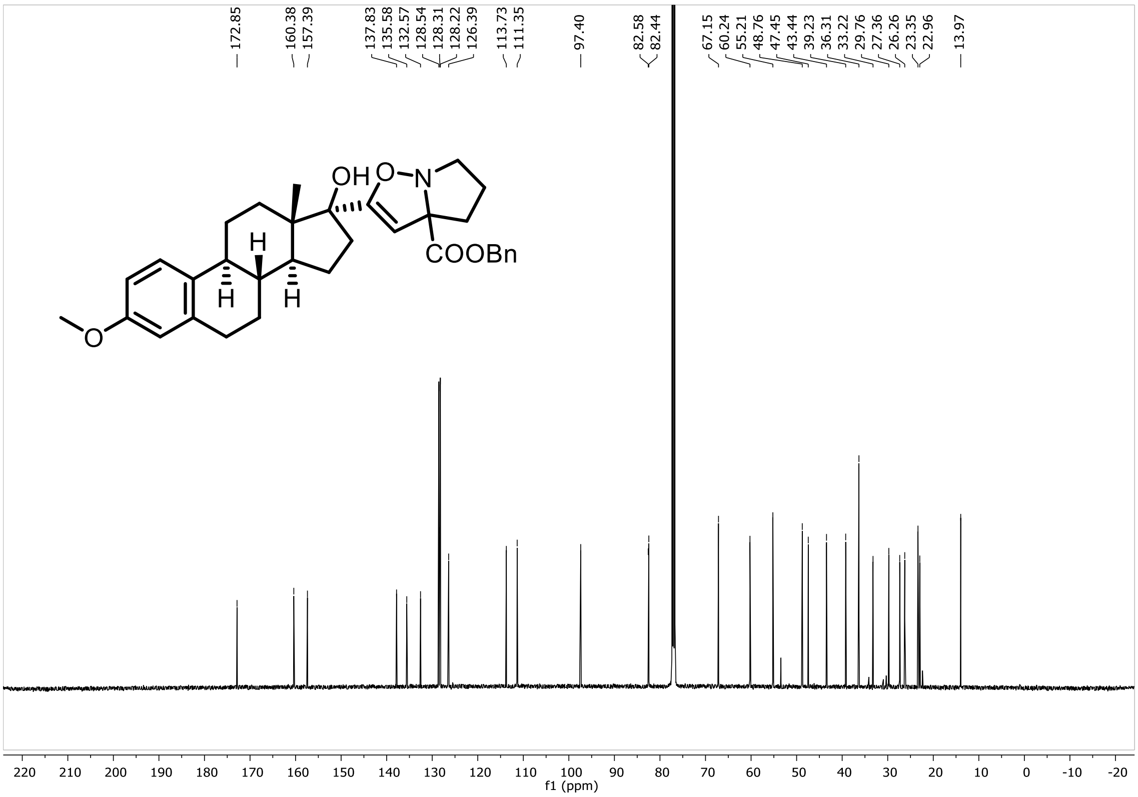

^{1}H NMR Spectra for Compound 2i minor

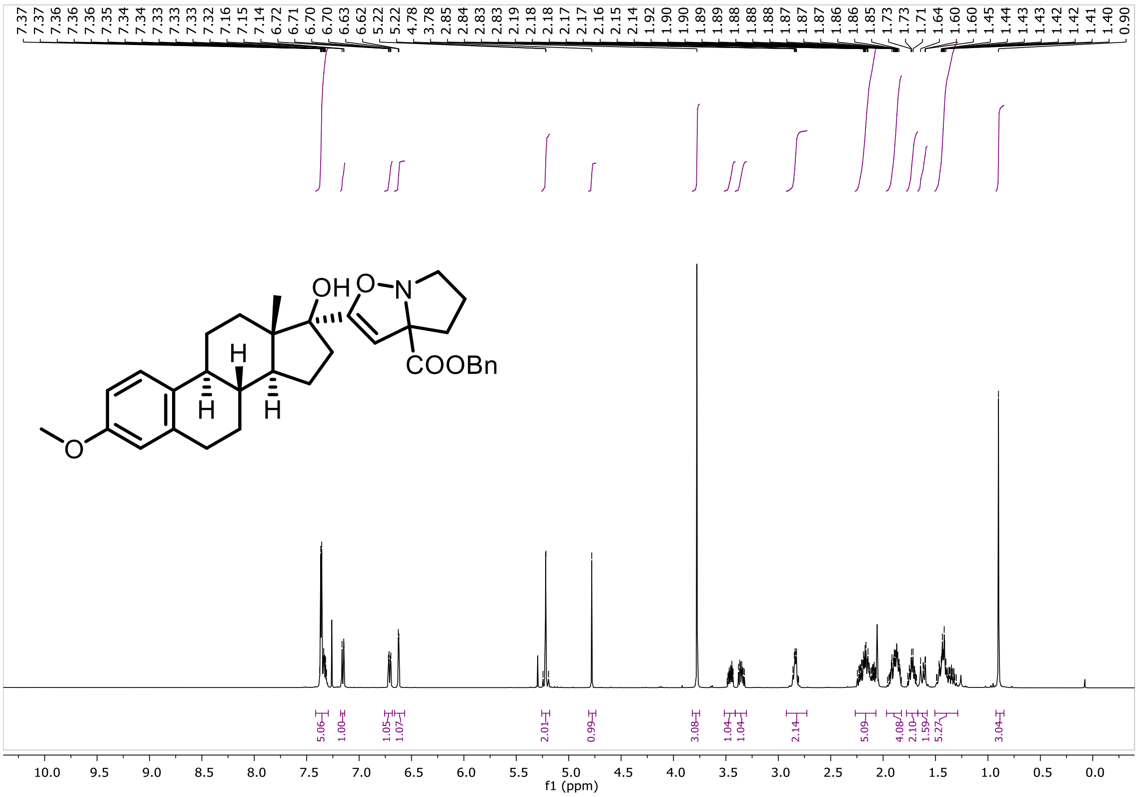

^{13}C NMR Spectra for Compound 2i minor

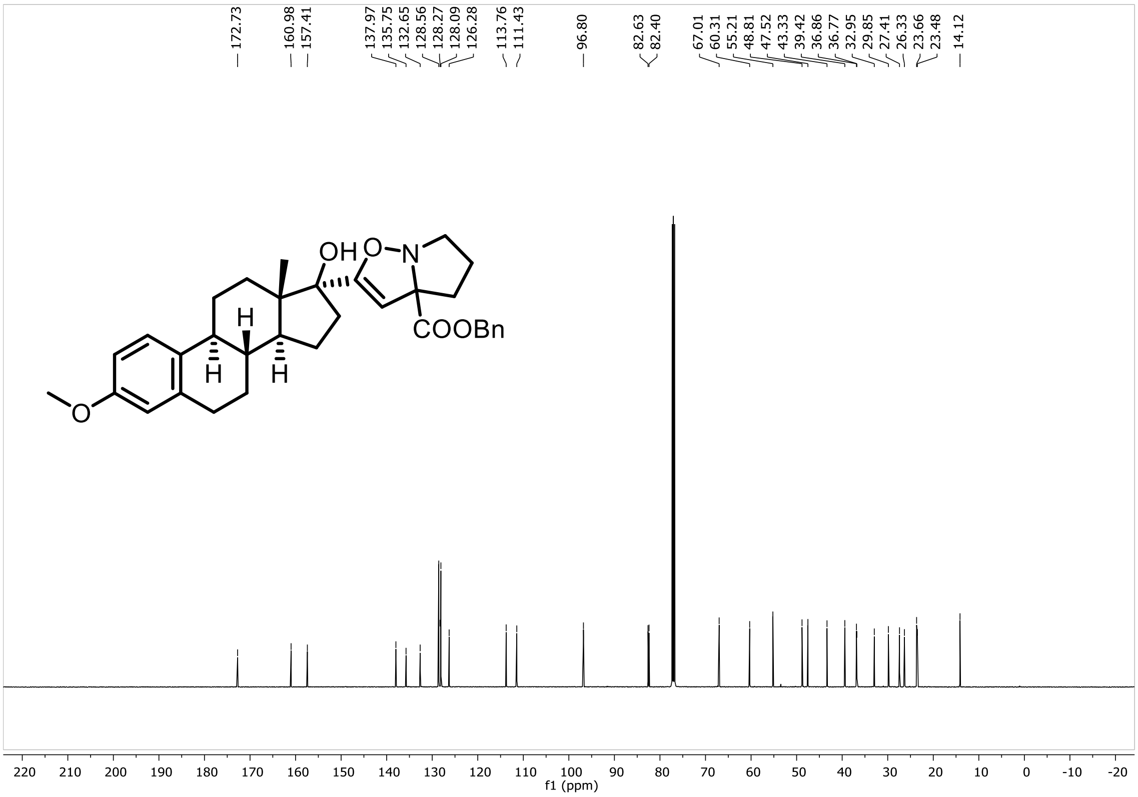

^{1}H NMR Spectra for Compound 2p Major

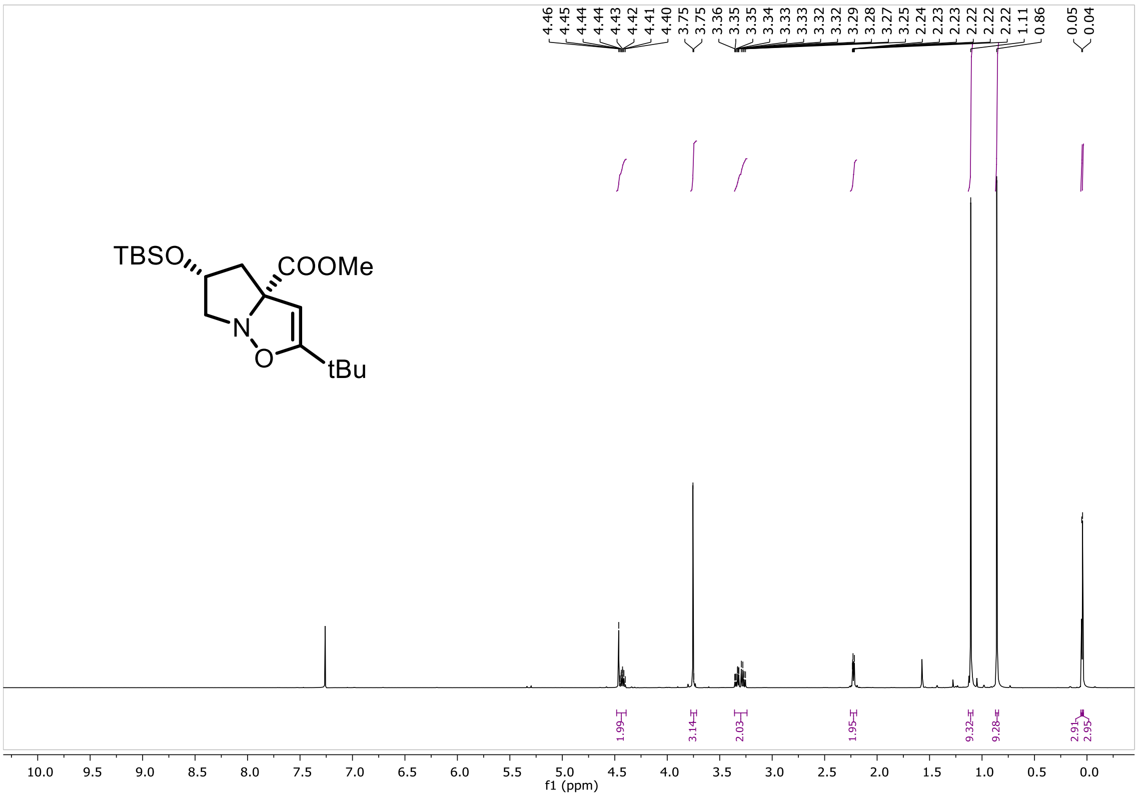

^{13}C NMR Spectra for Compound 2p Major

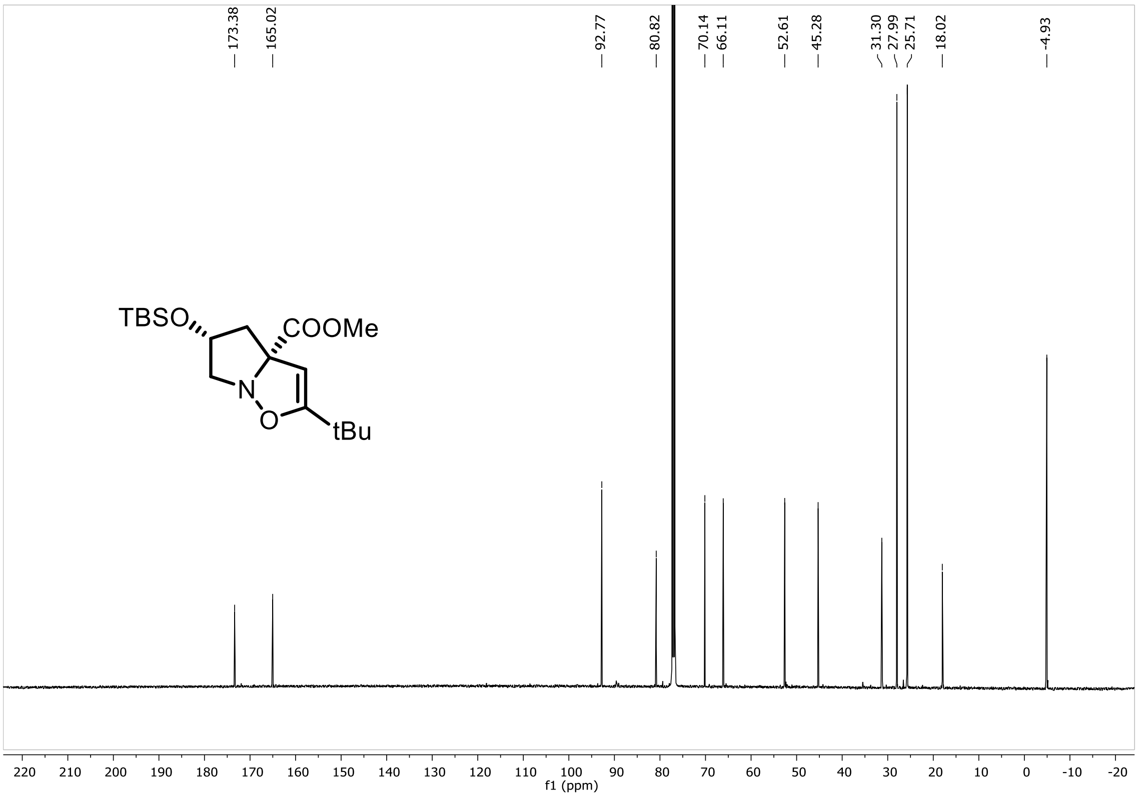

^{1}H NMR Spectra for Compound 2p Minor

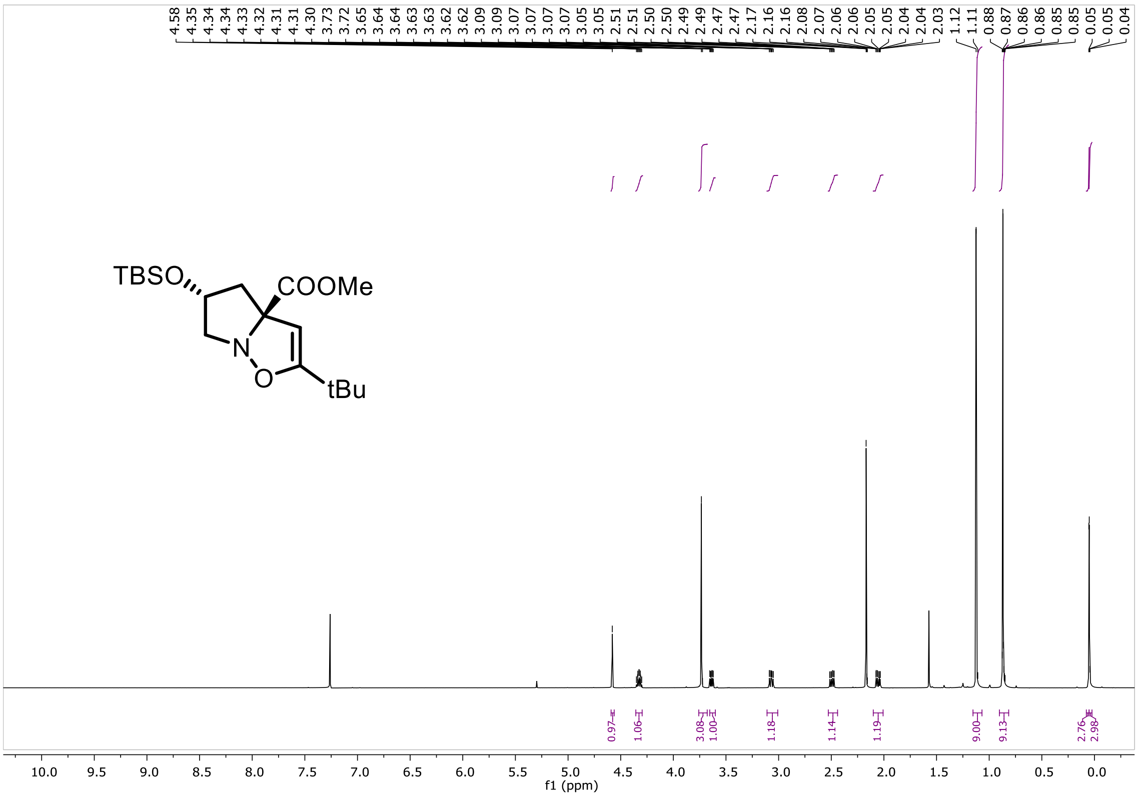

^{13}C NMR Spectra for Compound 2p Minor

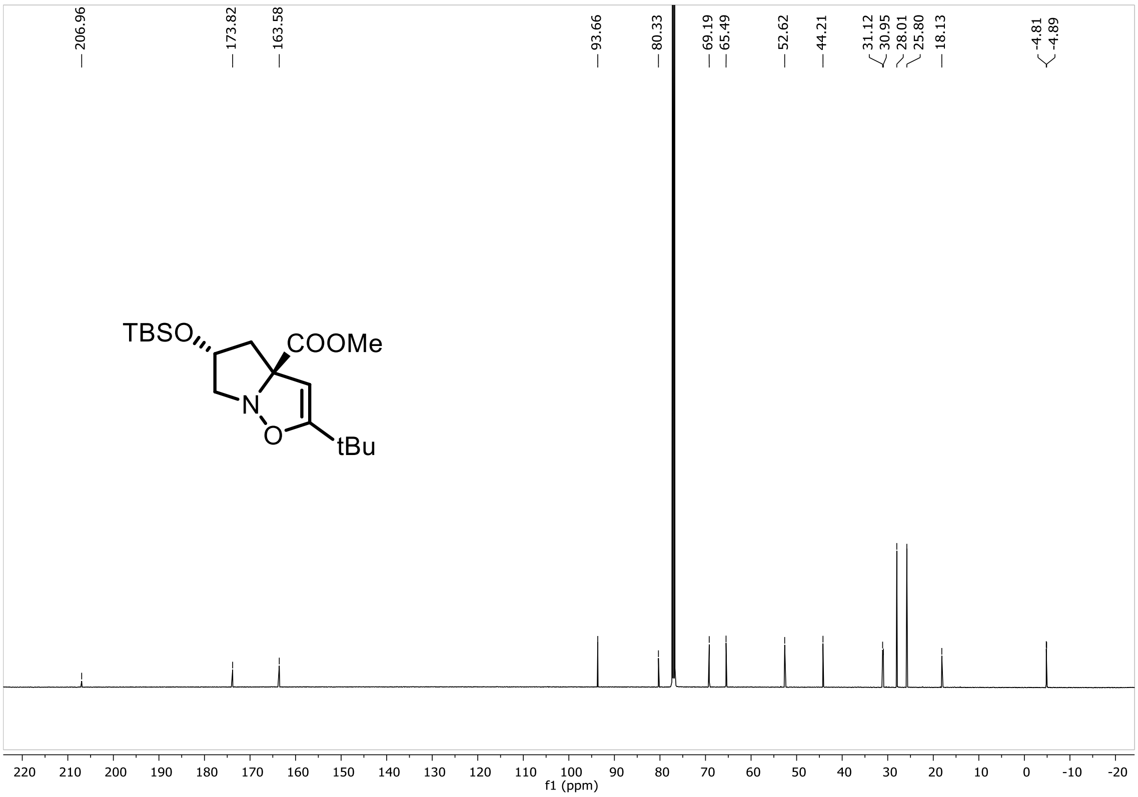

^{1}H NMR Spectra for Compound 2q

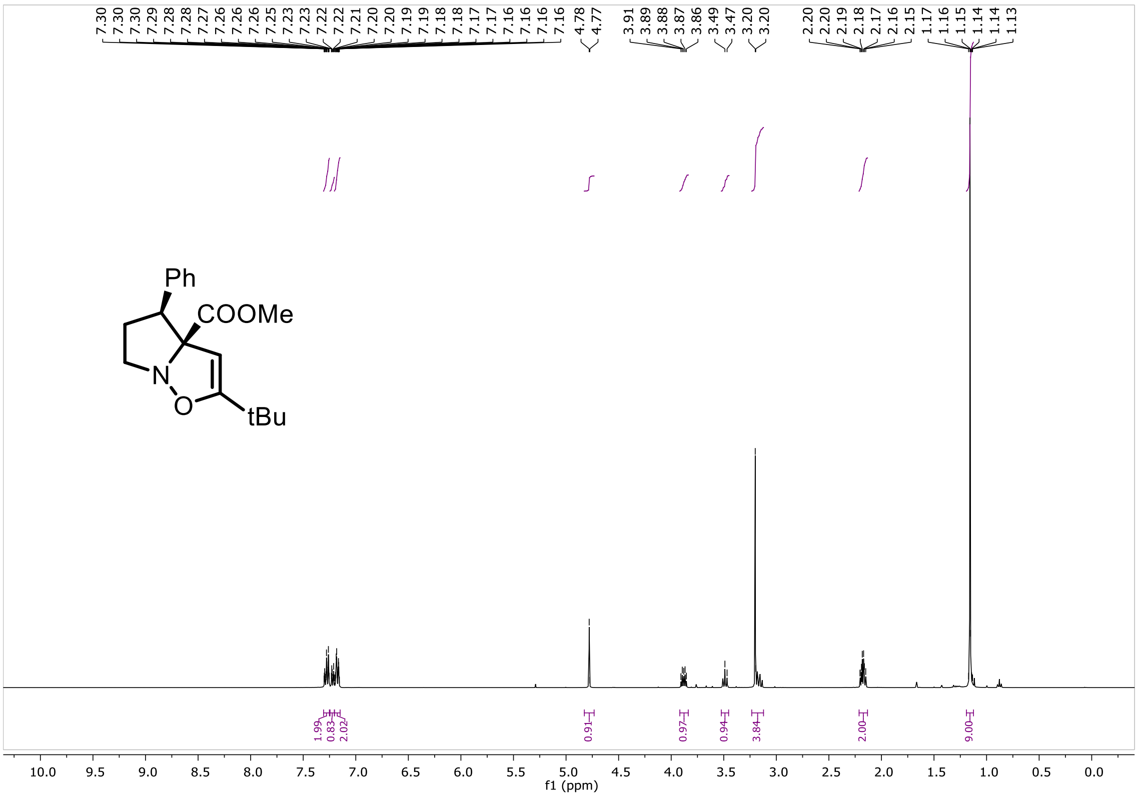

^{13}C NMR Spectra for Compound 2q

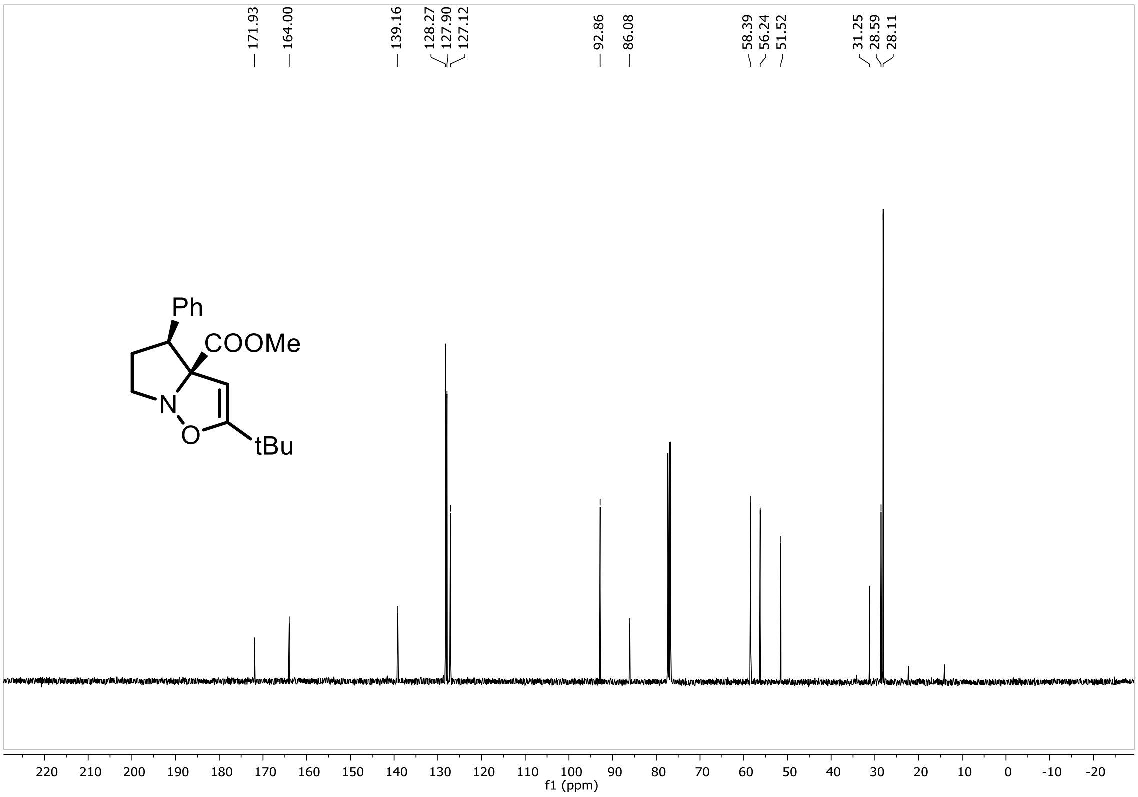

^{1}H NMR Spectra for Compound 2r

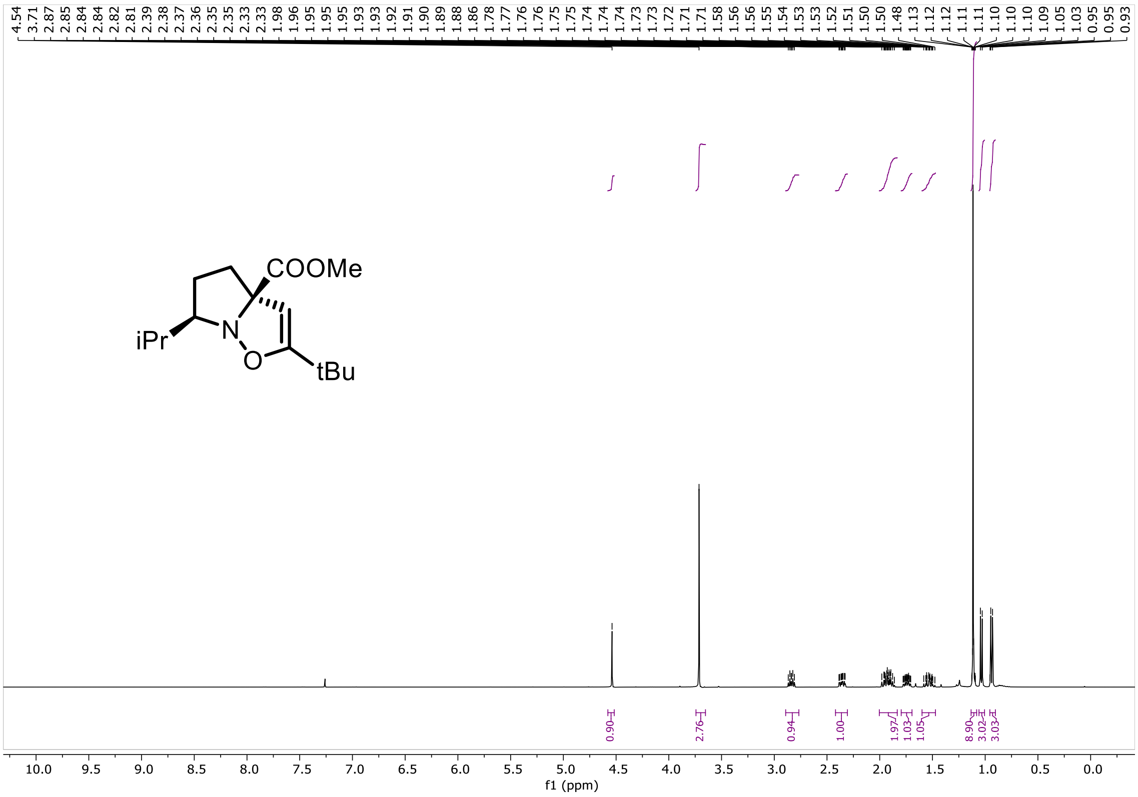

^{13}C NMR Spectra for Compound 2r

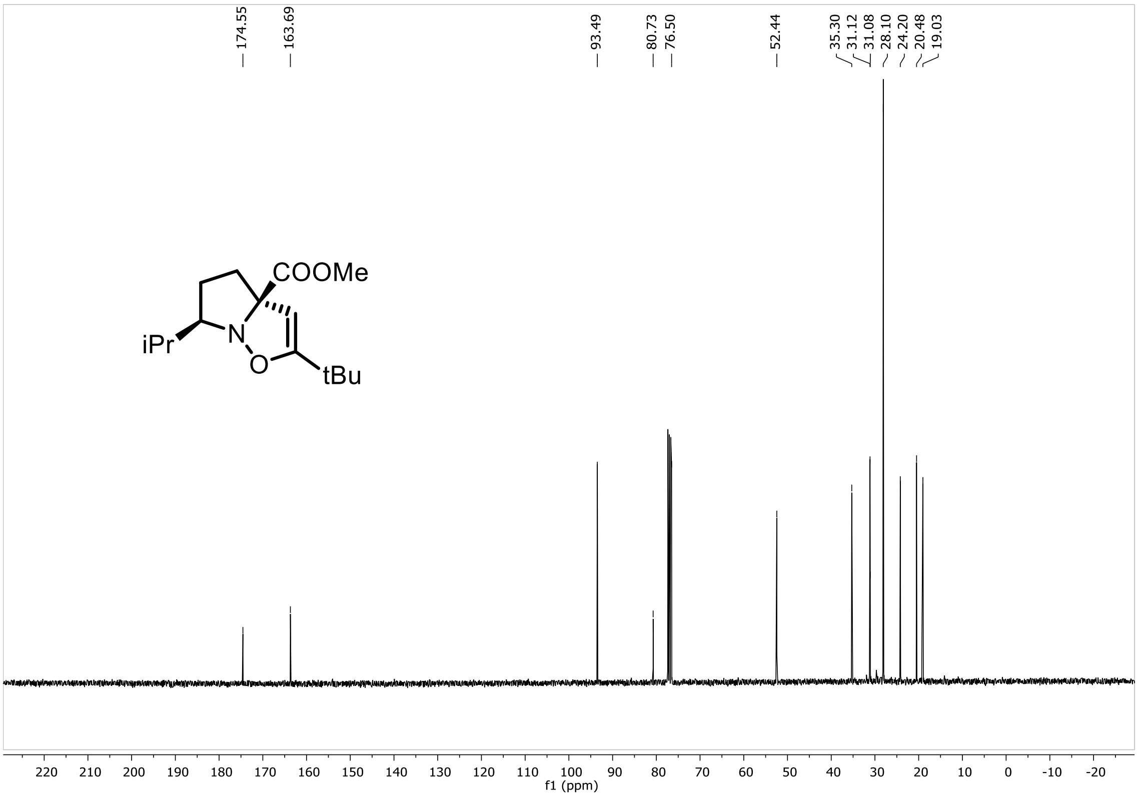

^{1}H NMR Spectra for Compound 3a

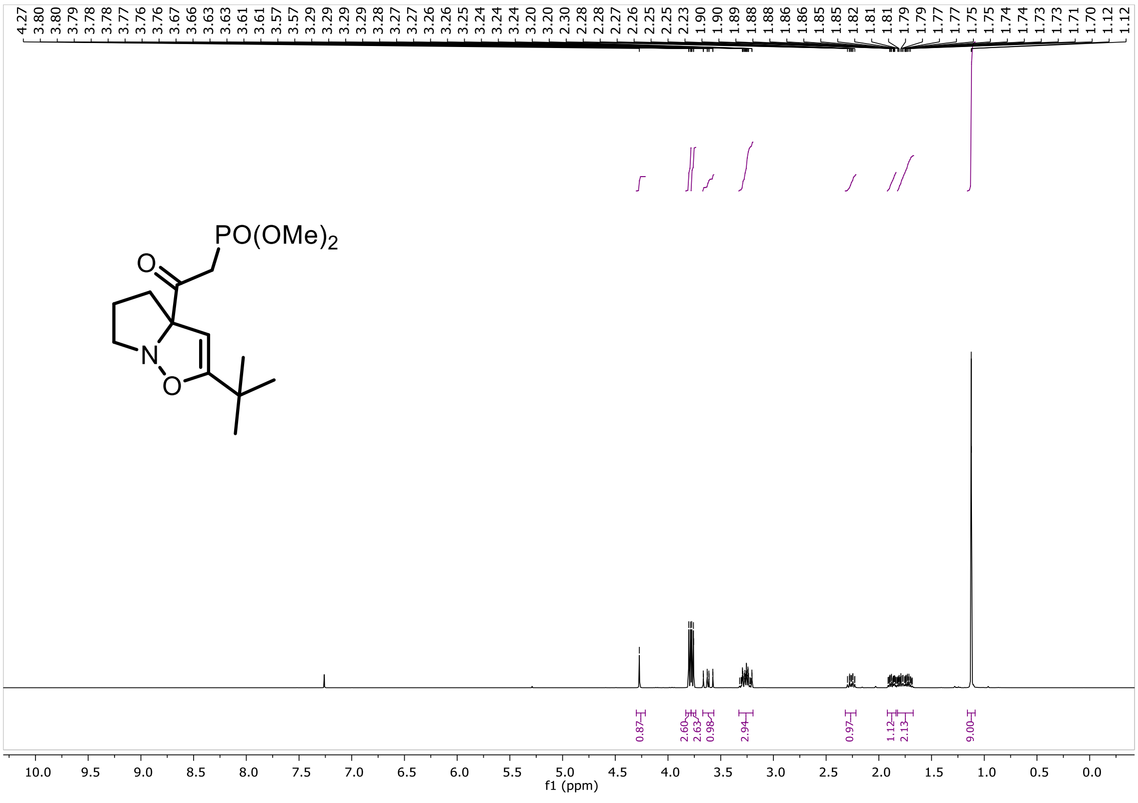

^{13}C NMR Spectra for Compound 3a

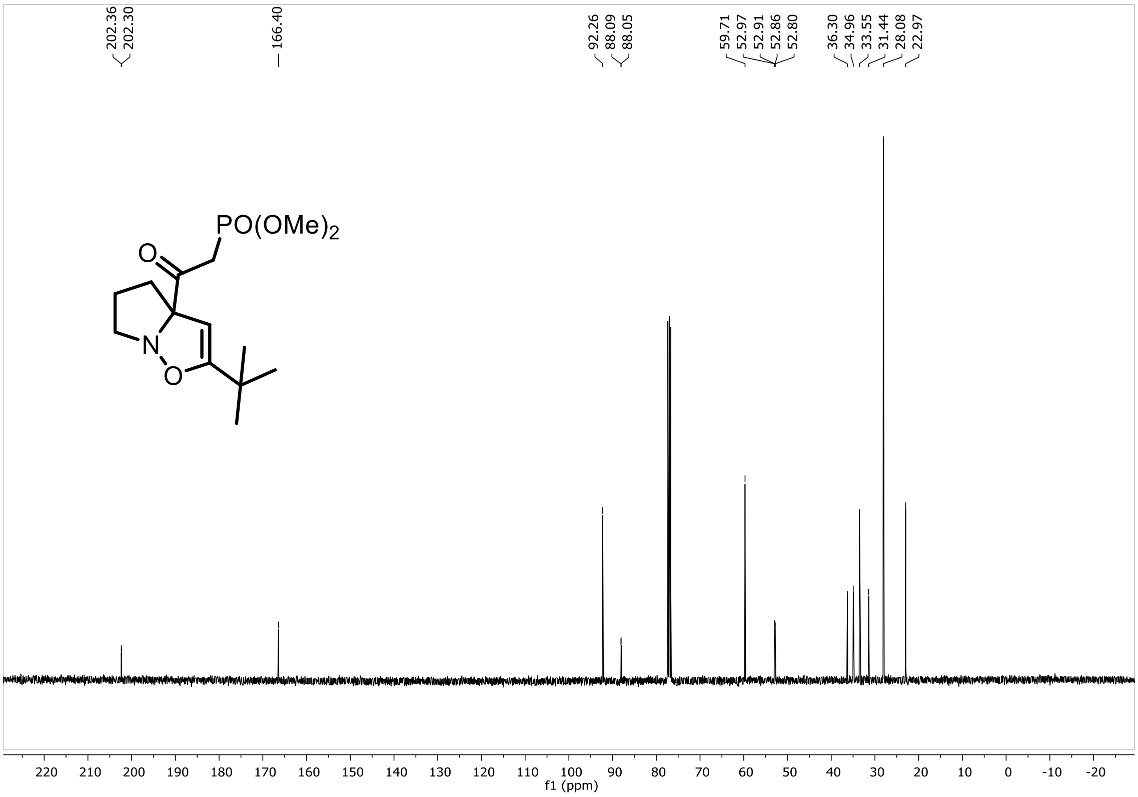

^{31}P NMR Spectra for Compound 3a

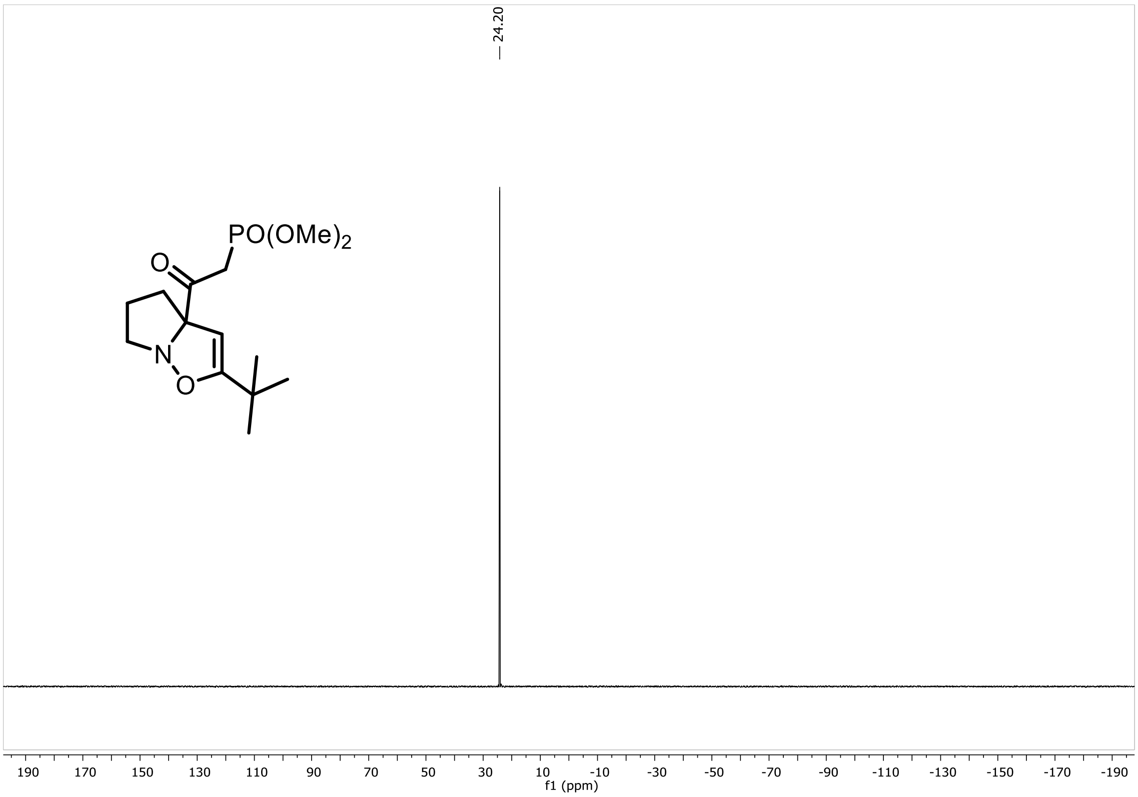

^{1}H NMR Spectra for Compound 3b

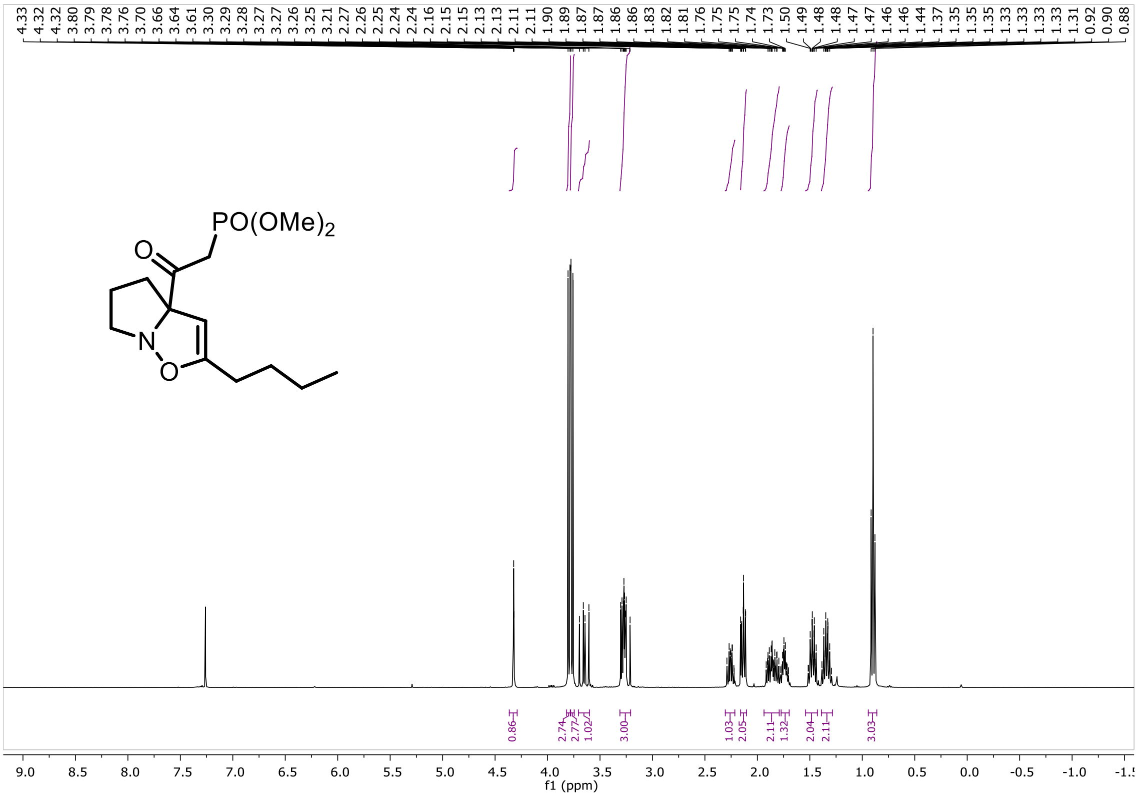

^{13}C NMR Spectra for Compound 3b

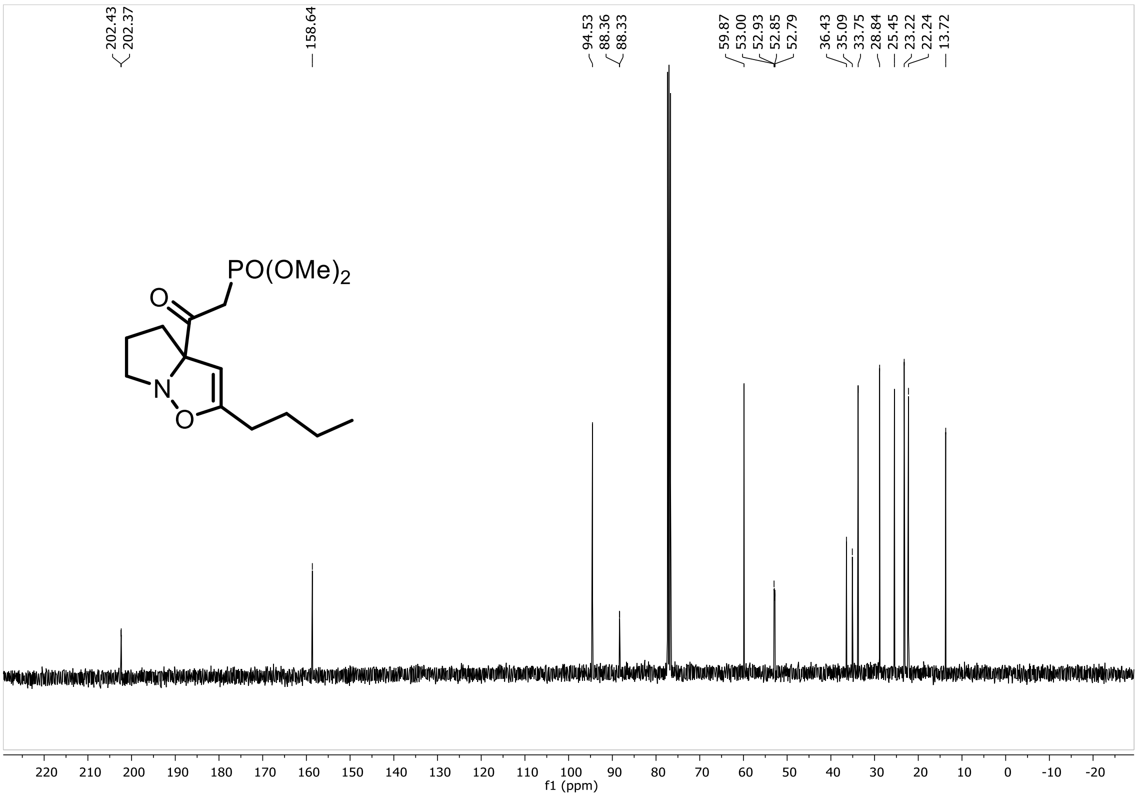

^{31}P NMR Spectra for Compound 3b

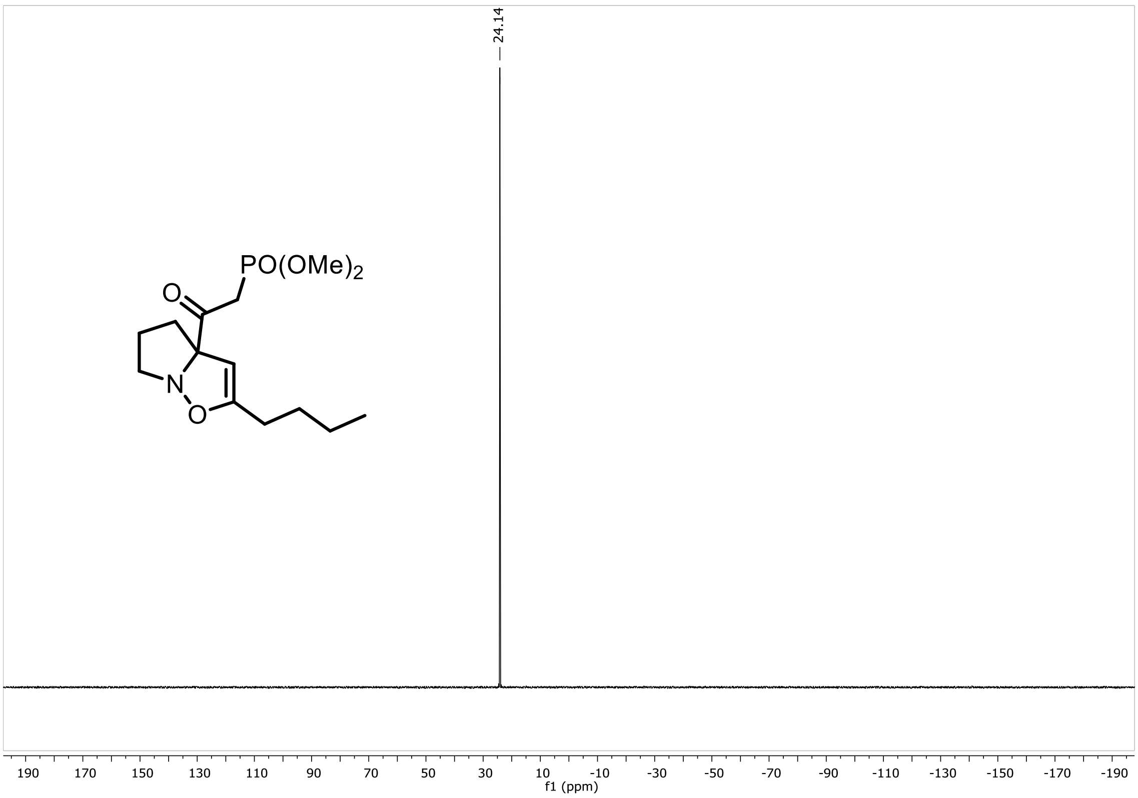

^{1}H NMR Spectra for Compound 3c

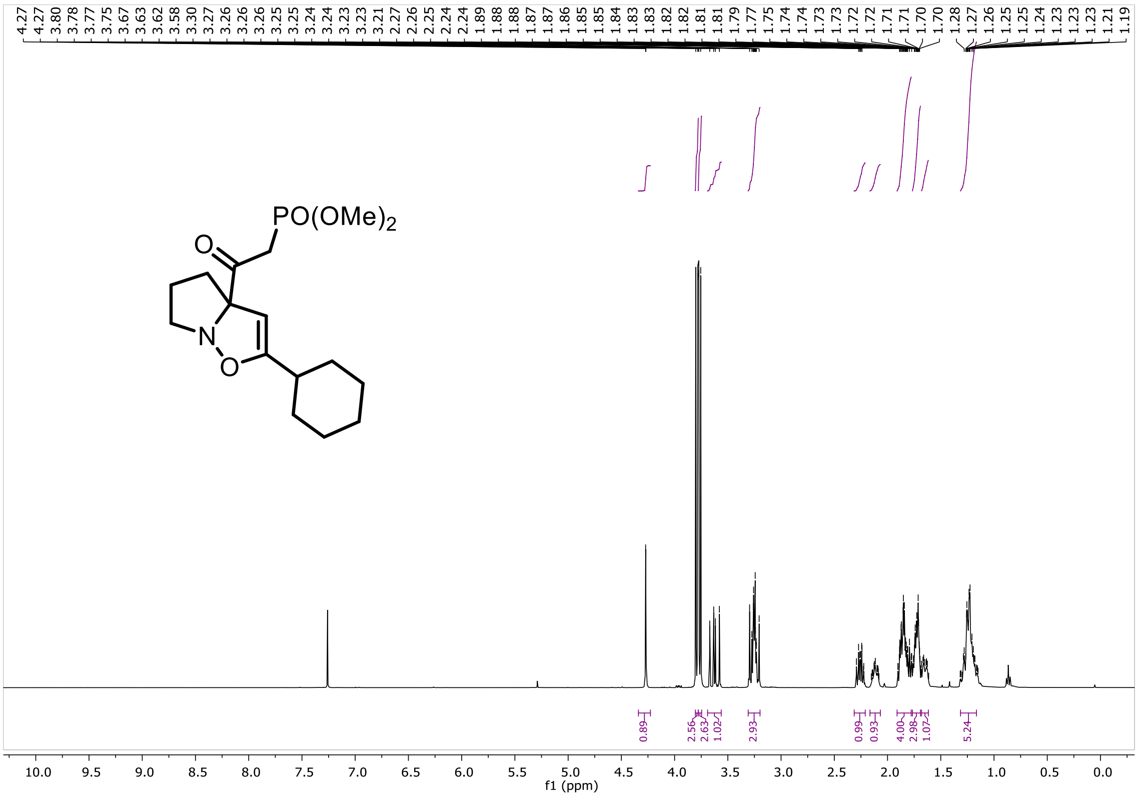

^{13}C NMR Spectra for Compound 3c

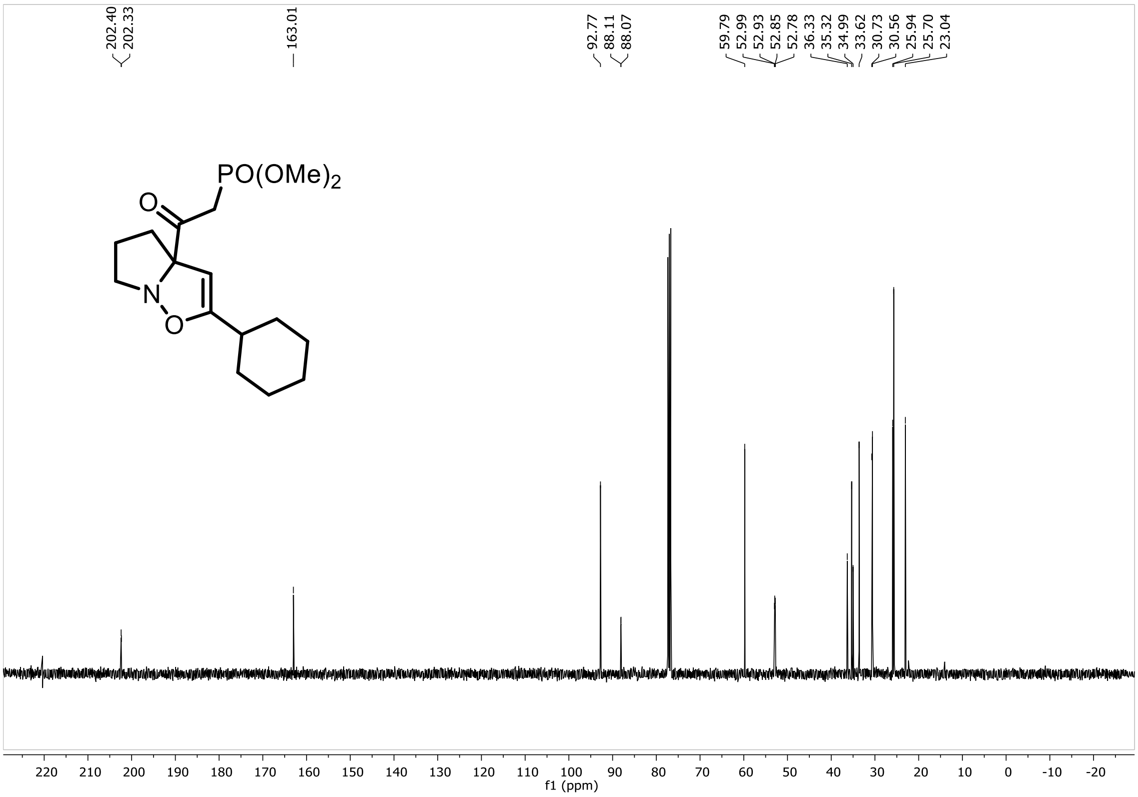

^{31}P NMR Spectra for Compound 3c

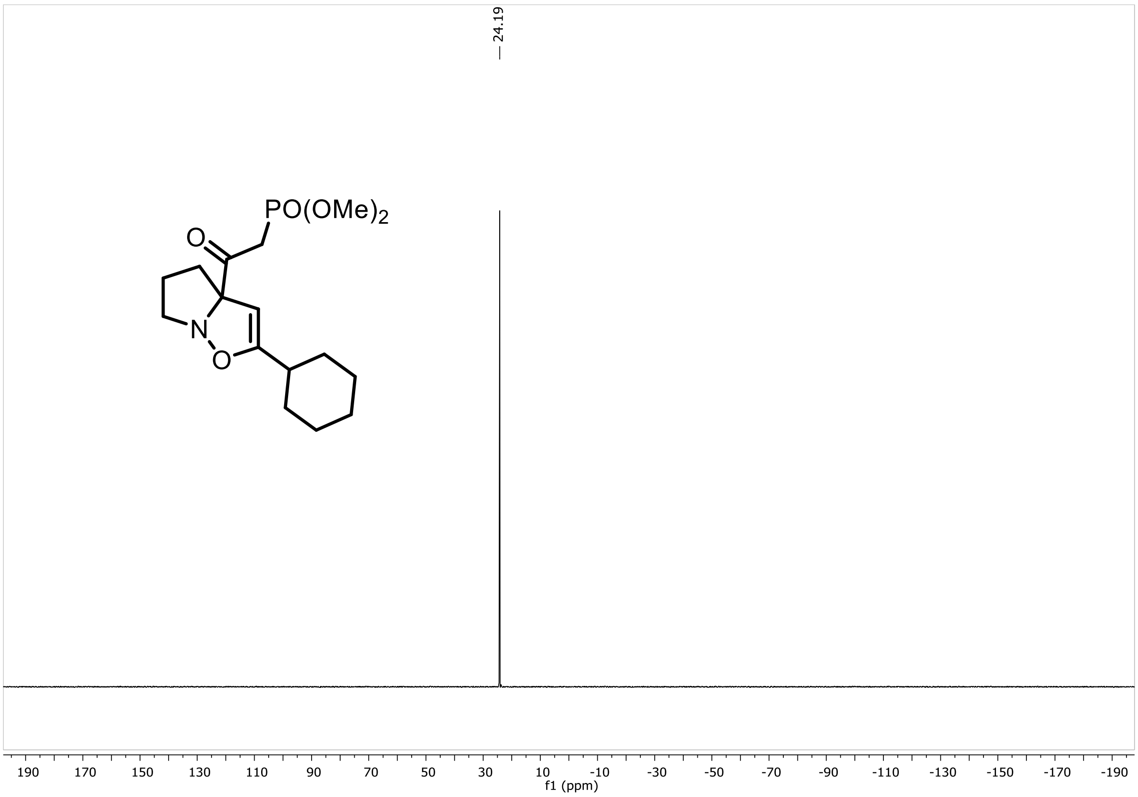

^{1}H NMR Spectra for Compound 3d

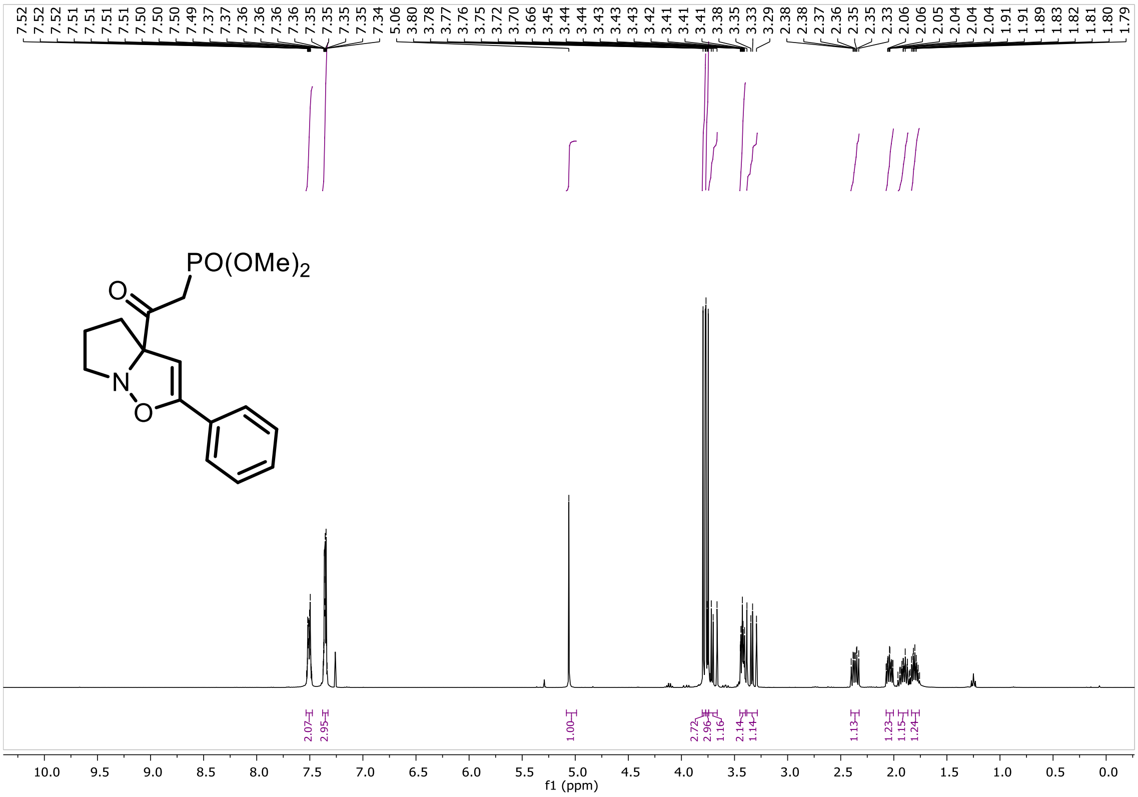

^{13}C NMR Spectra for Compound 3d

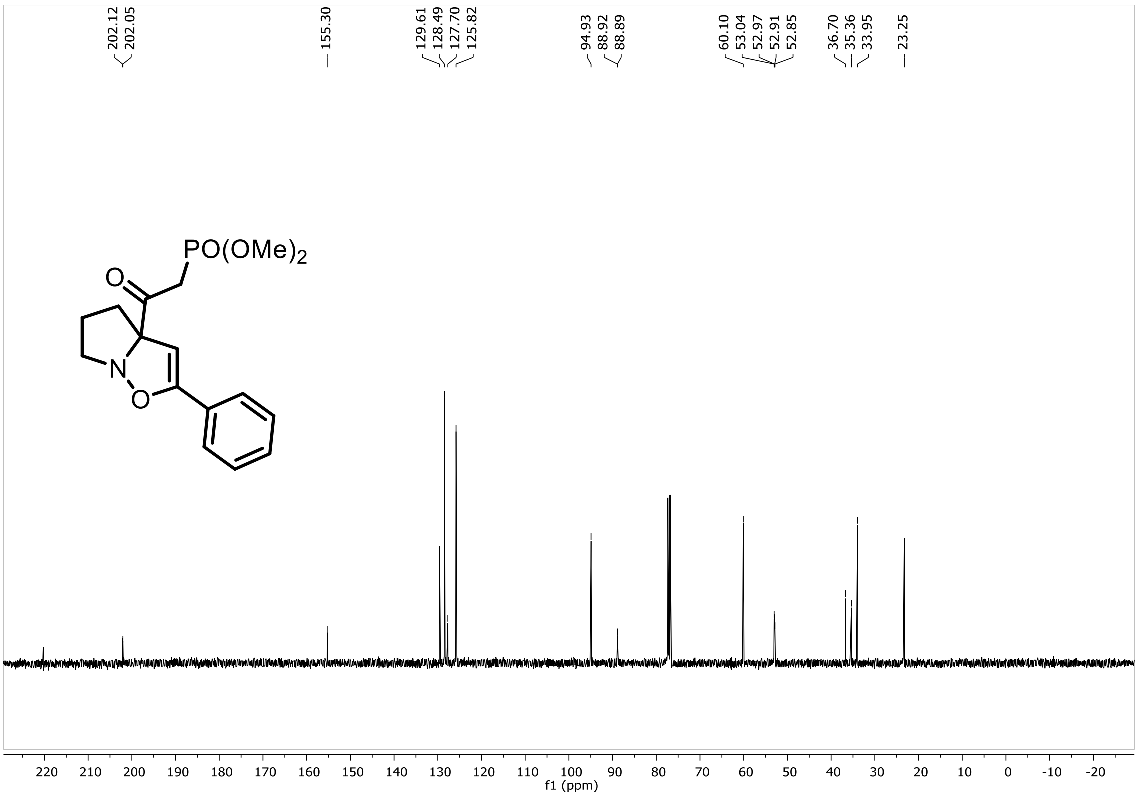

^{31}P NMR Spectra for Compound 3d

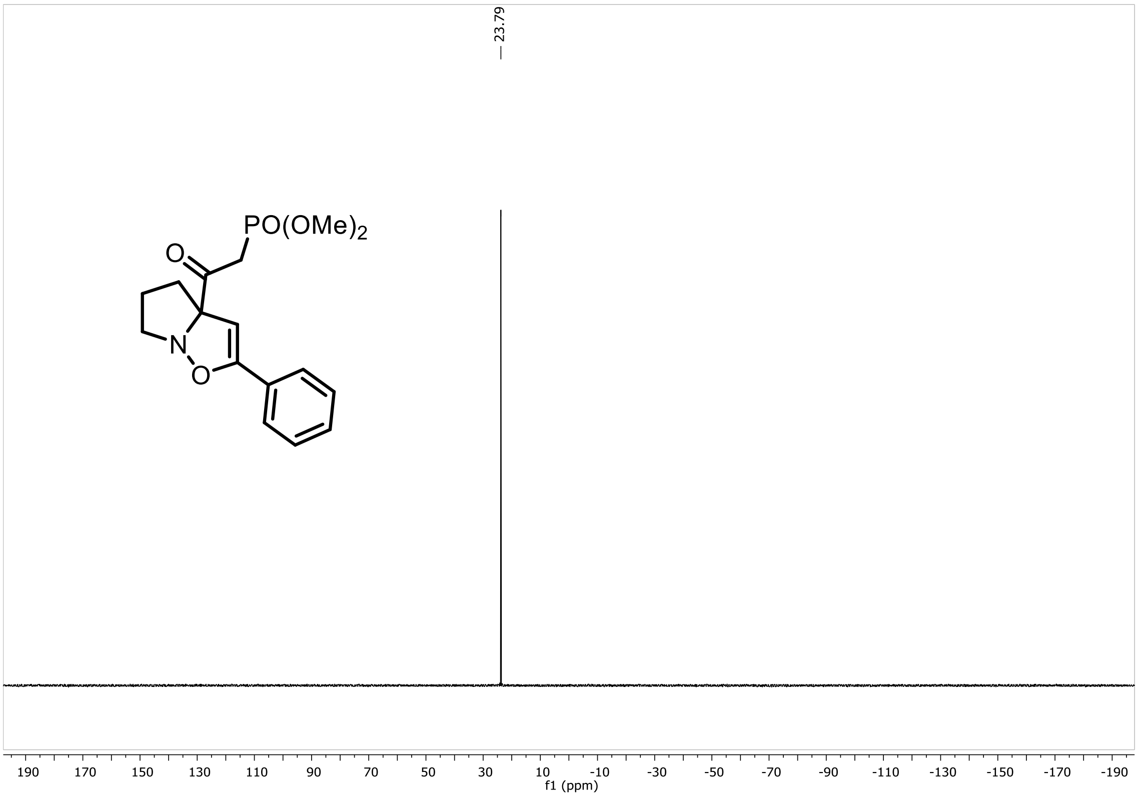

^{1}H NMR Spectra for Compound 3e

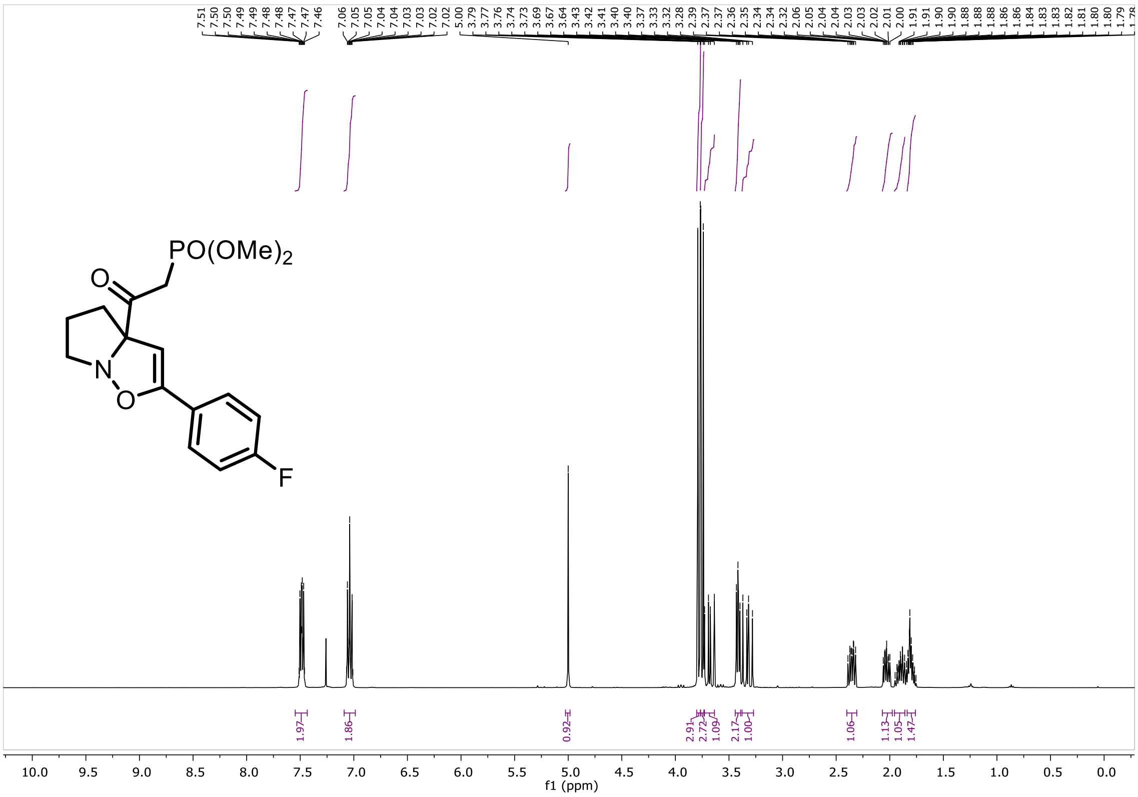

^{13}C NMR Spectra for Compound 3e

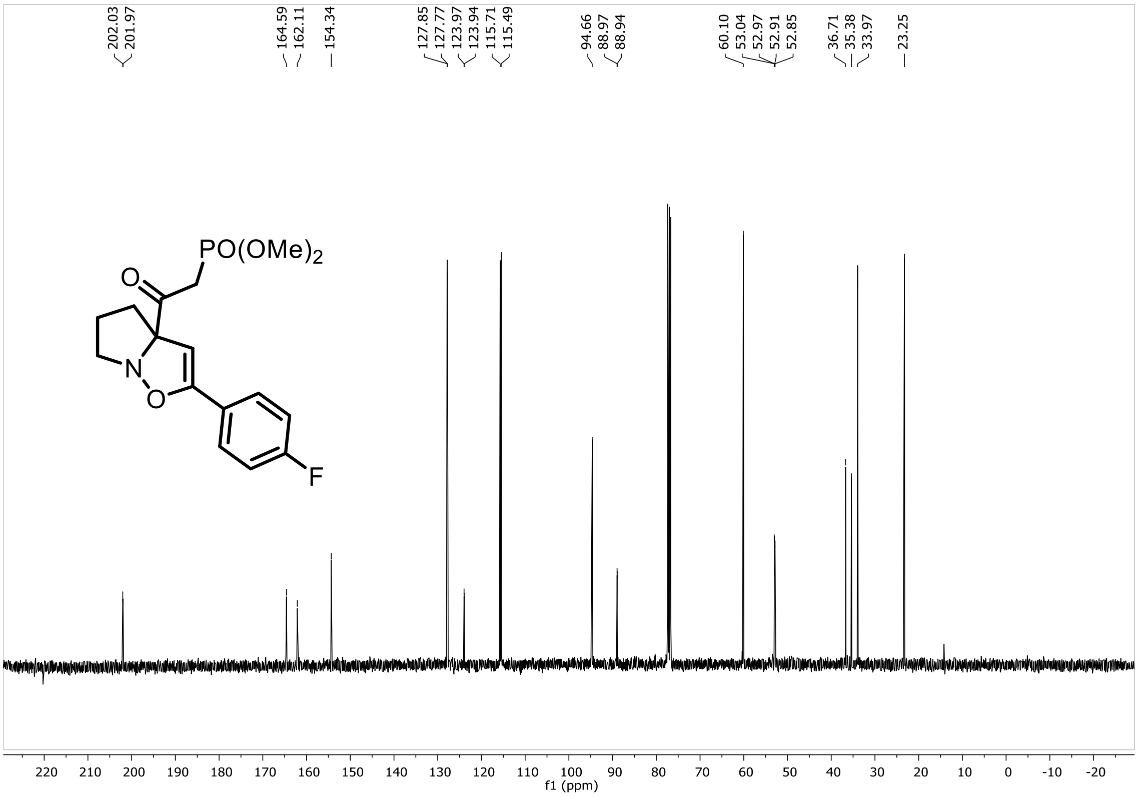

^{19}F NMR Spectra for Compound 3e

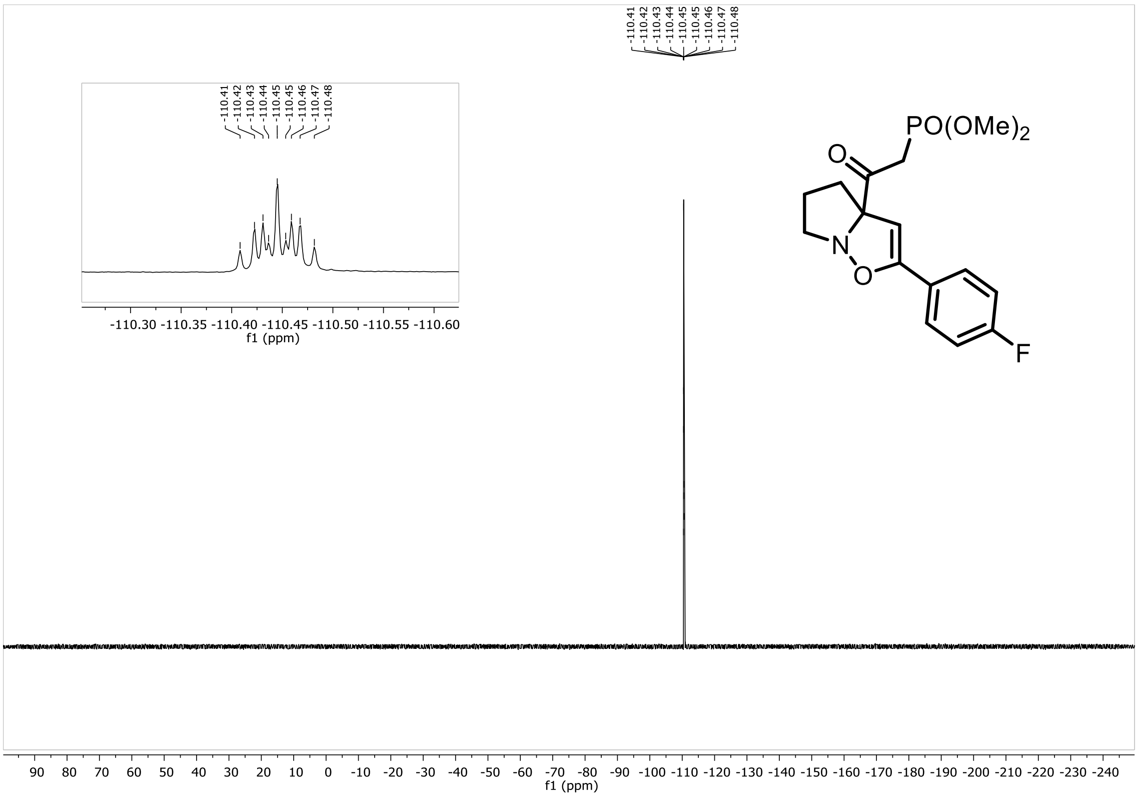

^{31}P NMR Spectra for Compound 3e

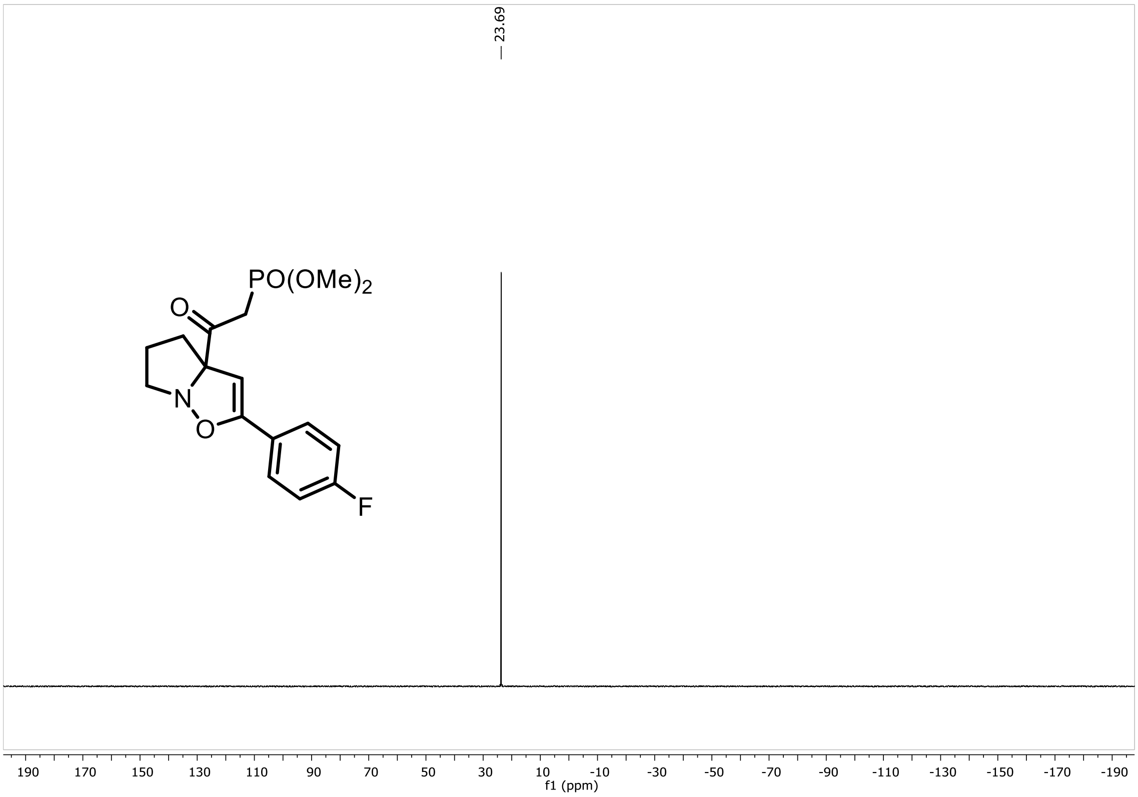

^{1}H NMR Spectra for Compound 3f

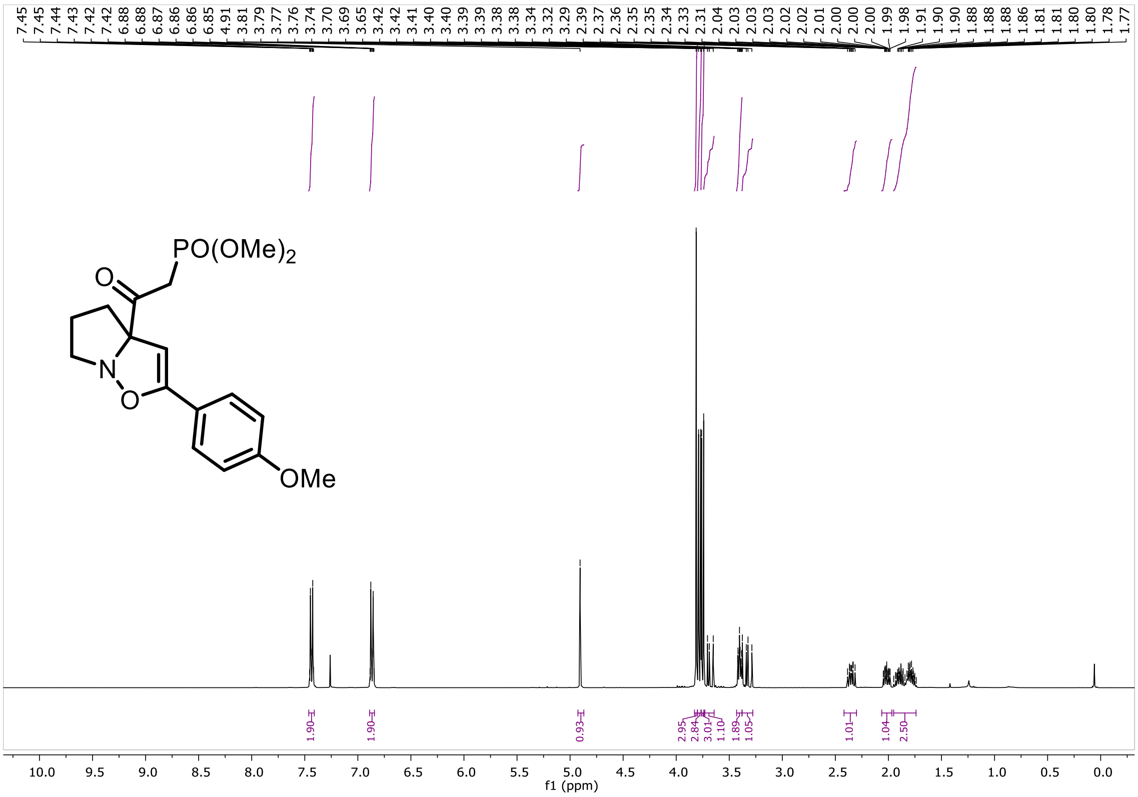

^{13}C NMR Spectra for Compound 3f

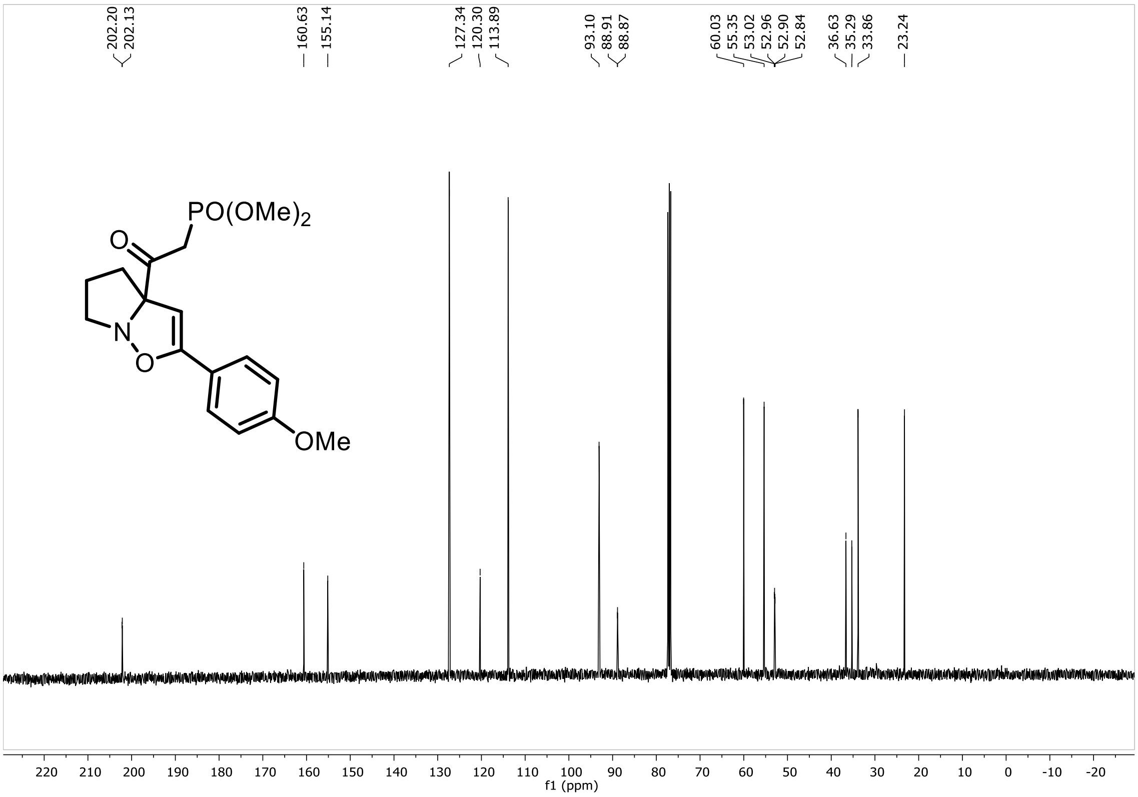

^{31}P NMR Spectra for Compound 3f

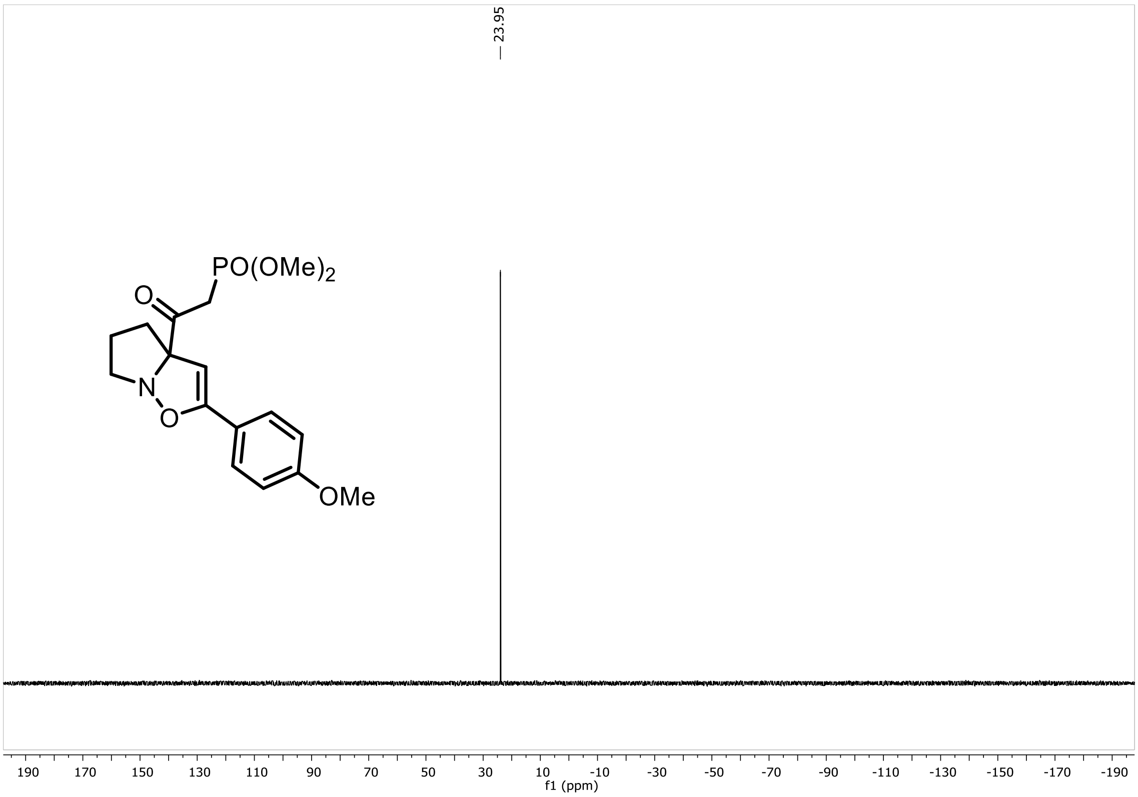

^{1}H NMR Spectra for Compound 3g

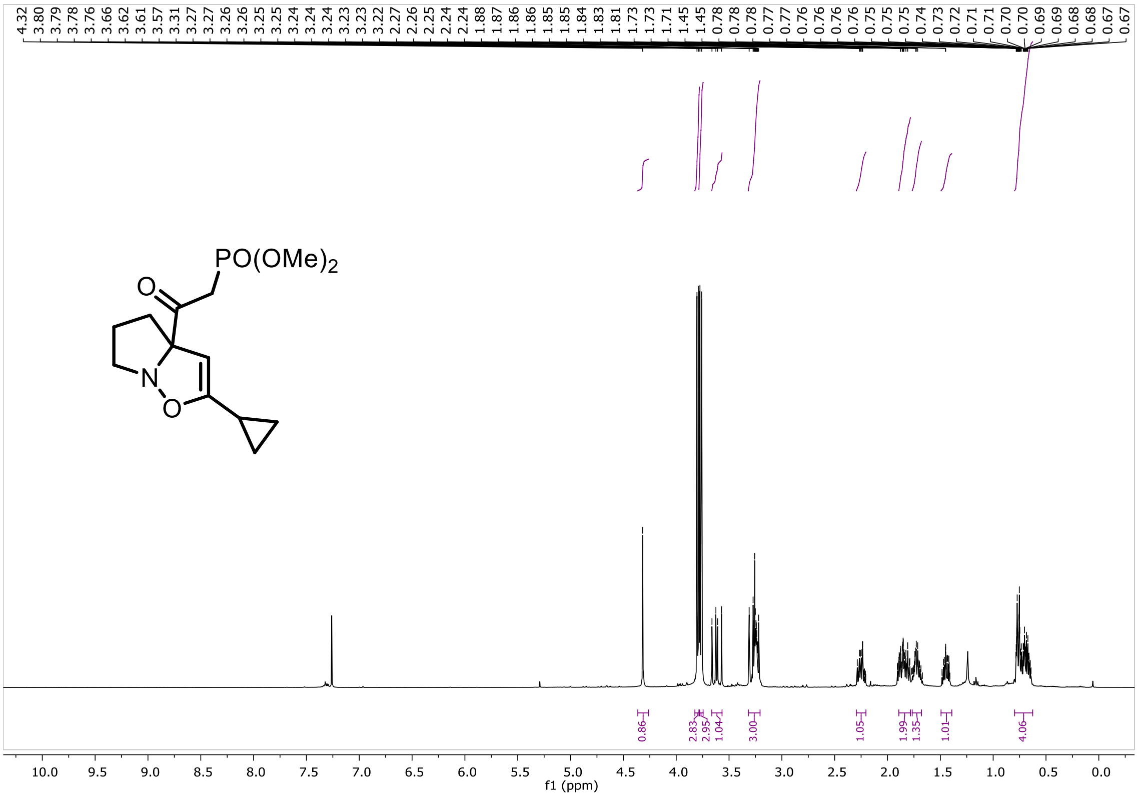

^{13}C NMR Spectra for Compound 3g

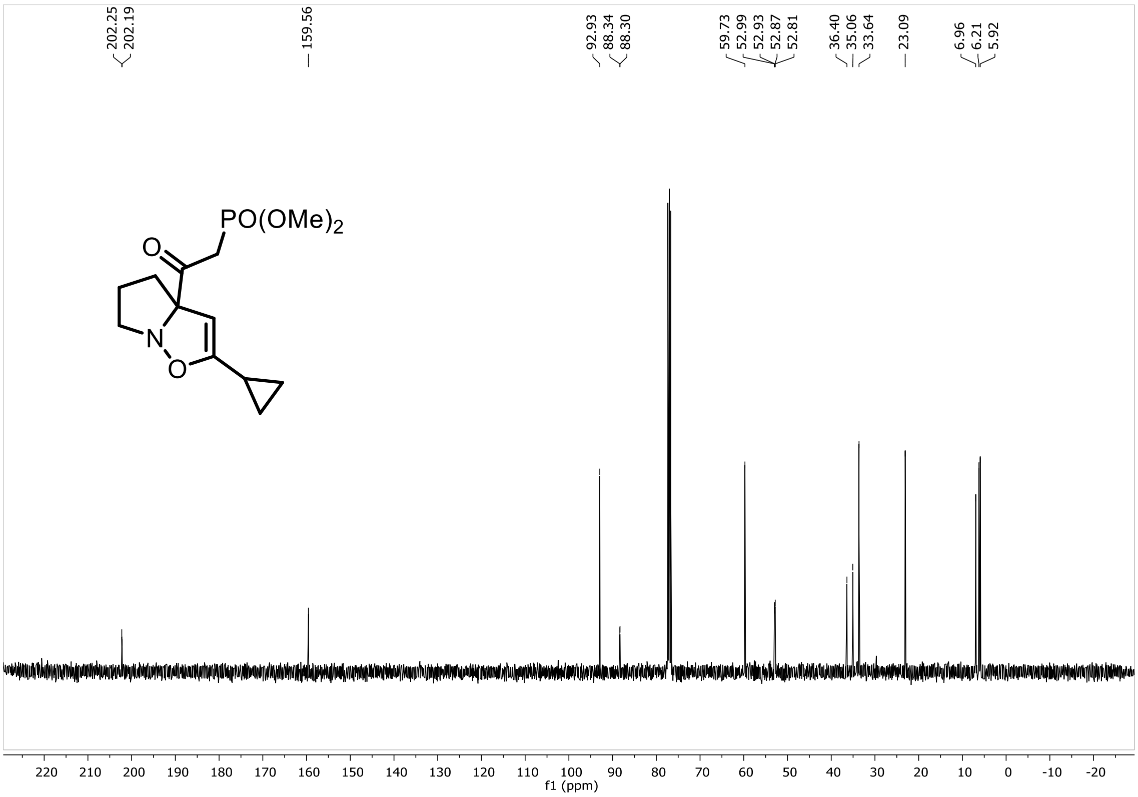

^{31}P NMR Spectra for Compound 3g

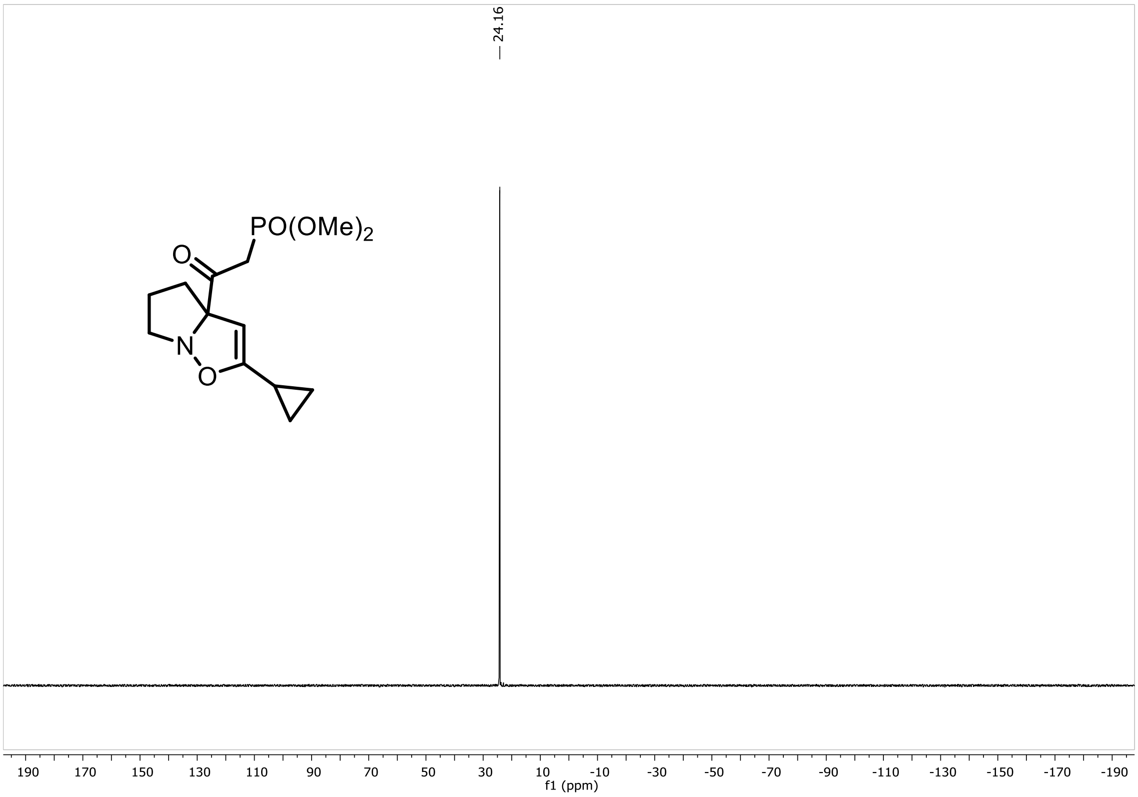

^{1}H NMR Spectra for Compound 3h

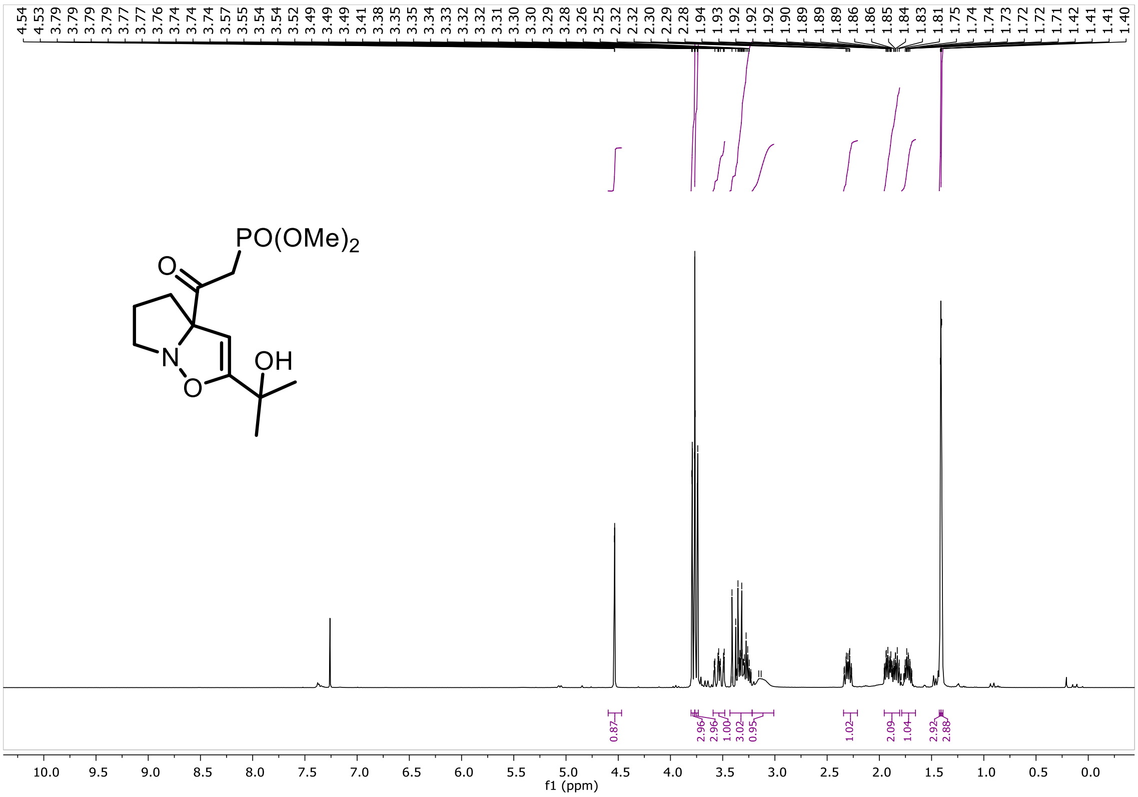

^{13}C NMR Spectra for Compound 3h

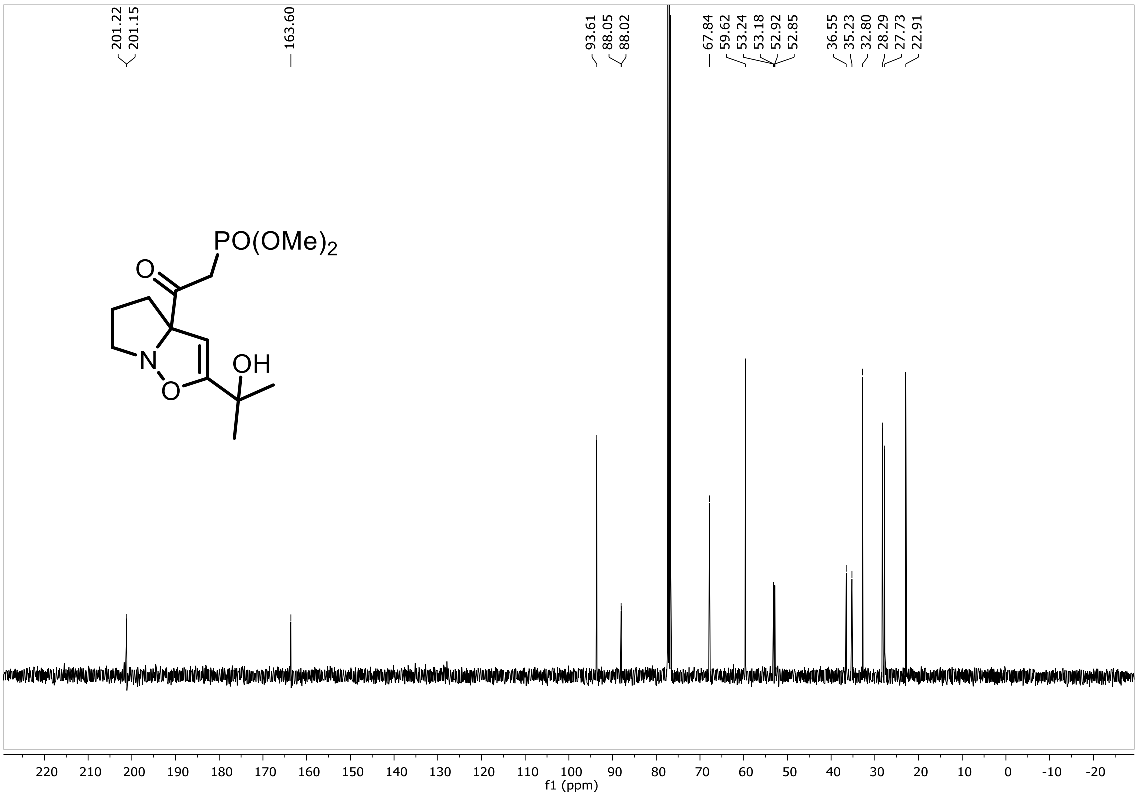

^{31}P NMR Spectra for Compound 3h

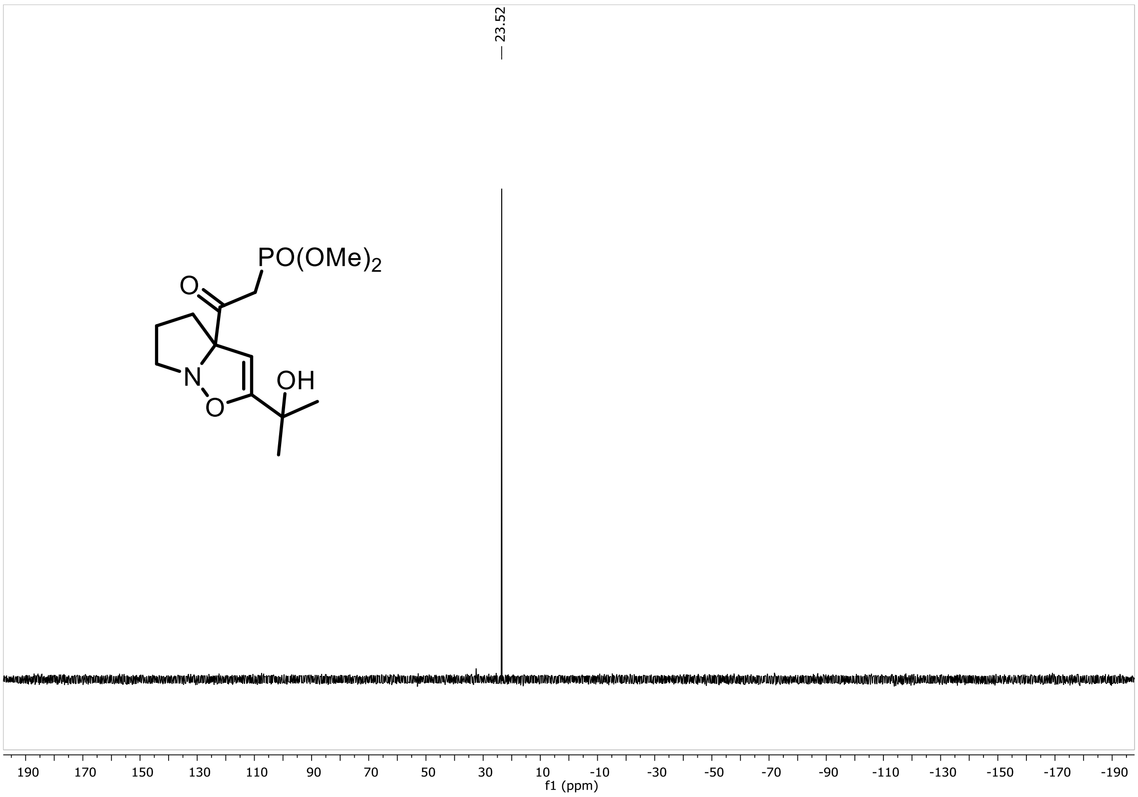

^{1}H NMR Spectra for Compound 3i

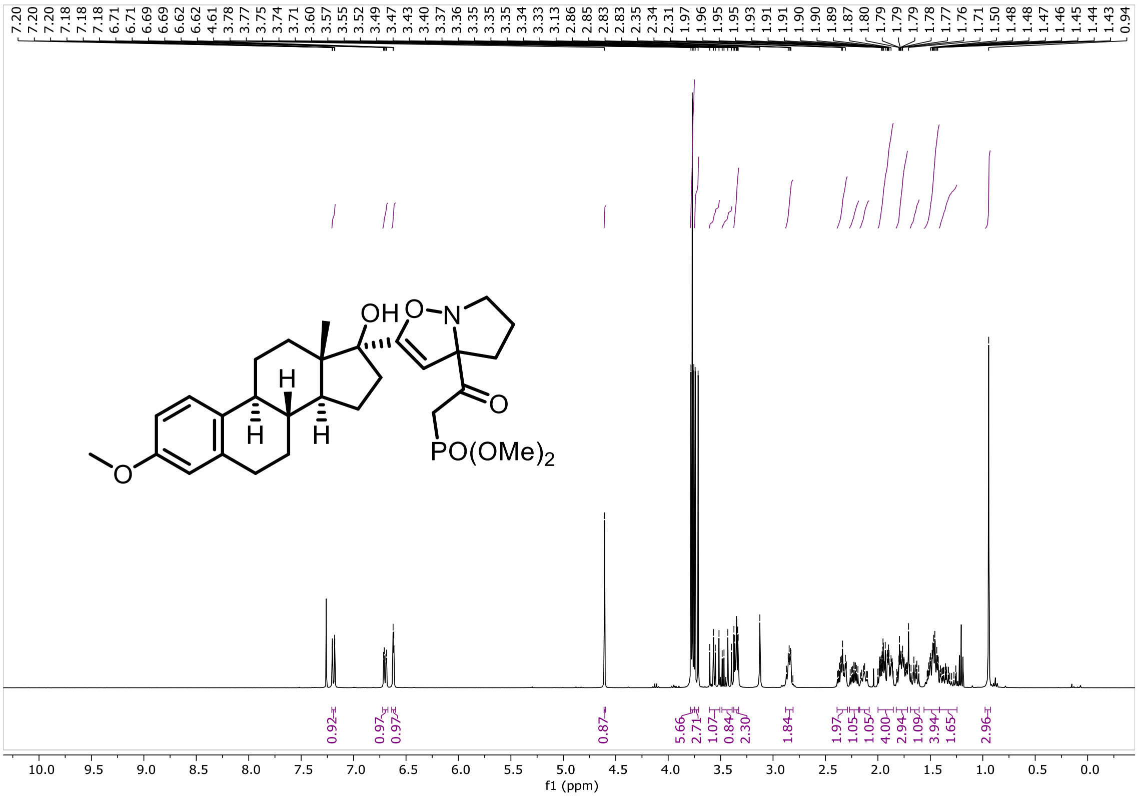

^{13}C NMR Spectra for Compound 3i

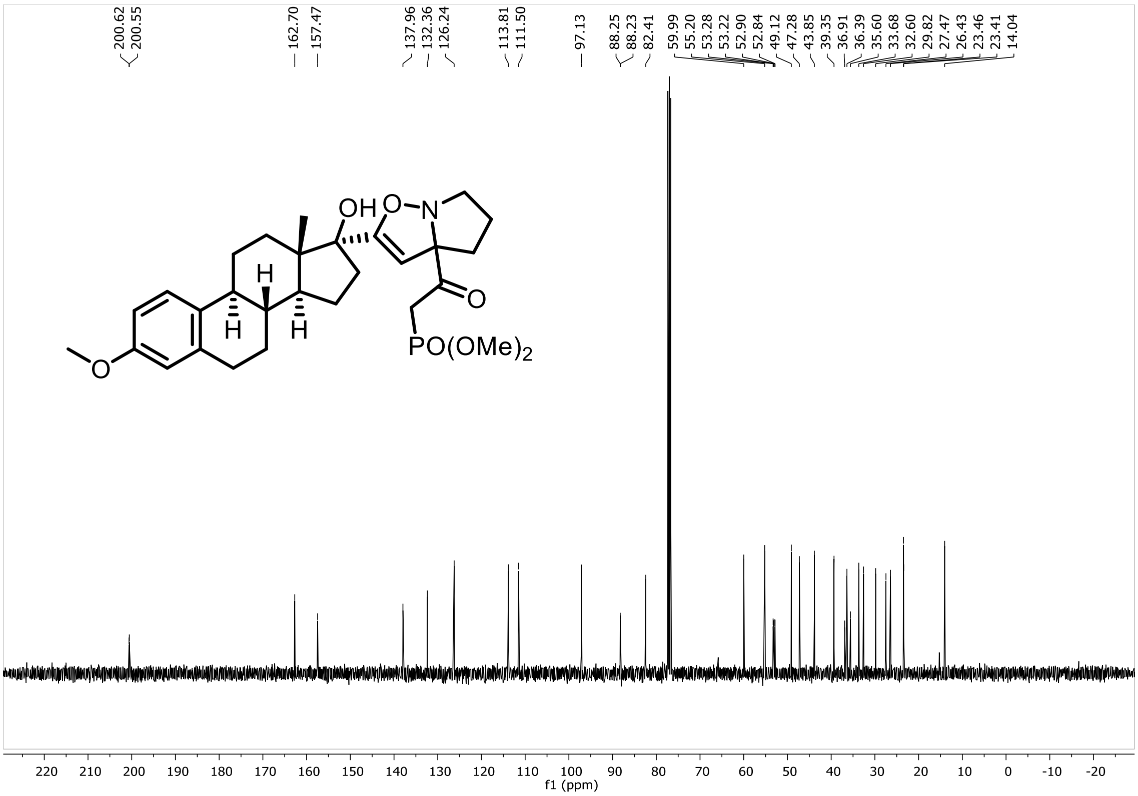

^{31}P NMR Spectra for Compound 3i

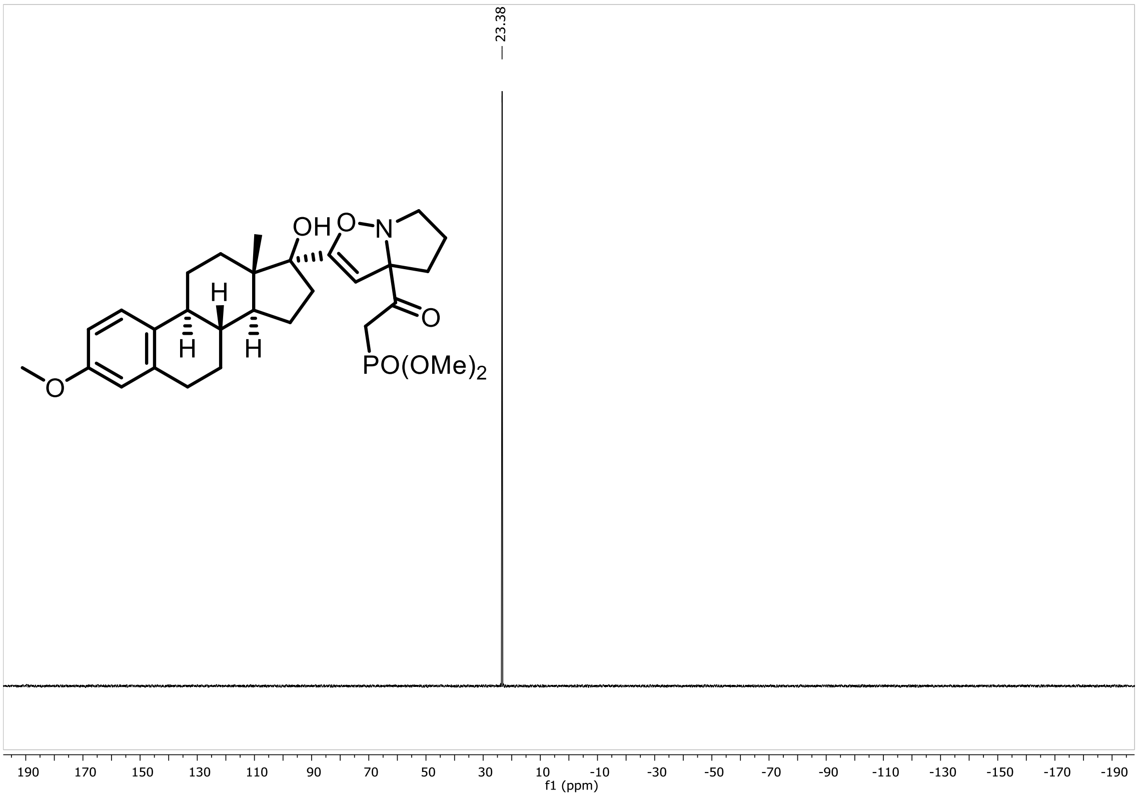

^{1}H NMR Spectra for Compound mixture 3j

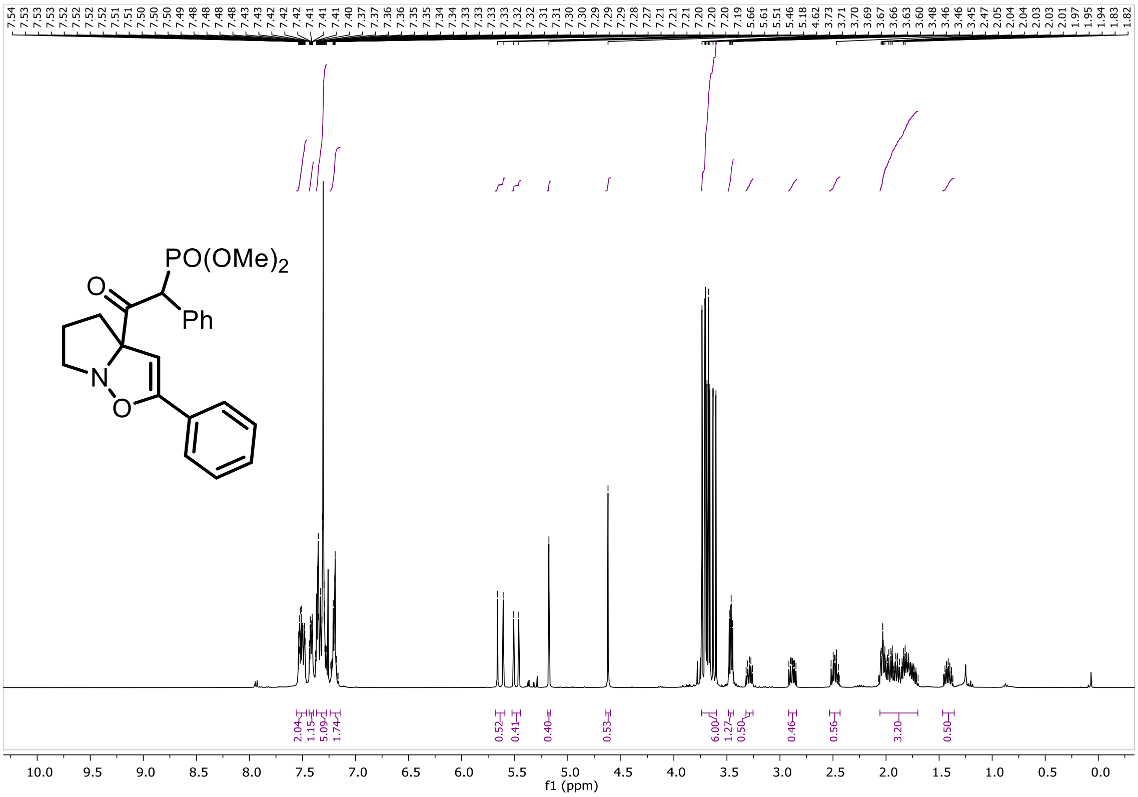

^{13}C NMR Spectra for Compound mixture 3j

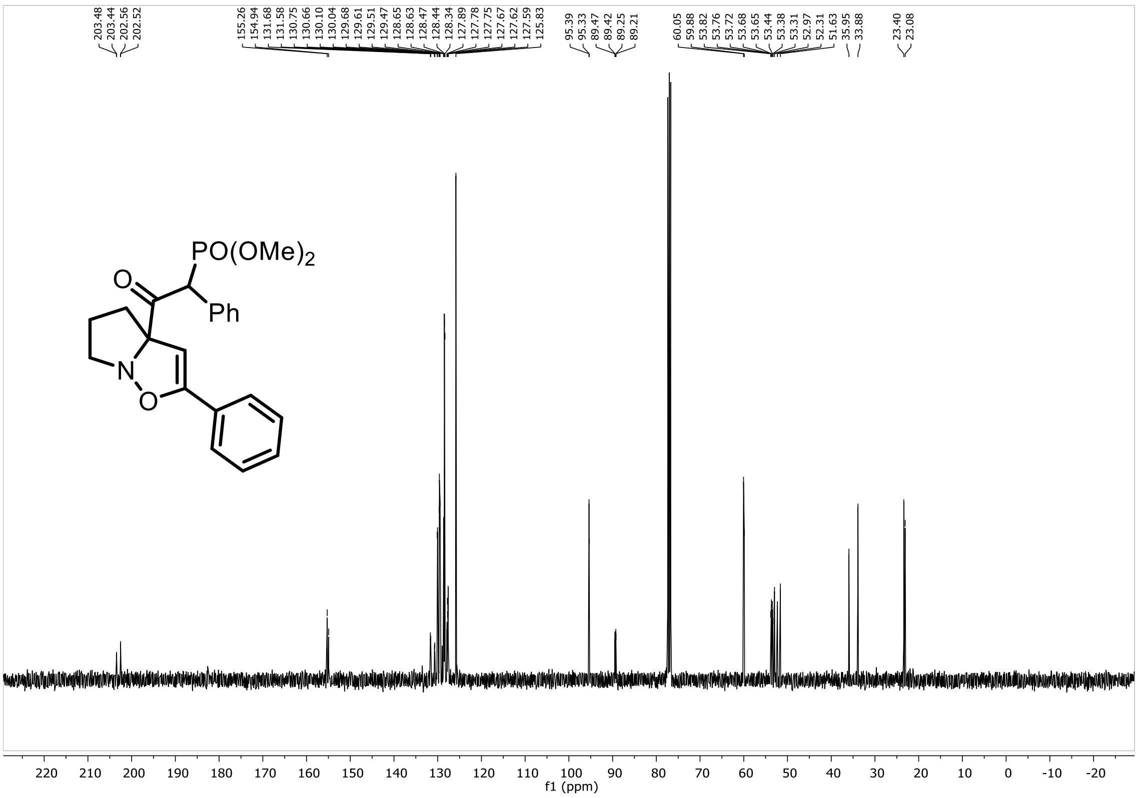

^{31}P NMR Spectra for Compound mixture 3j

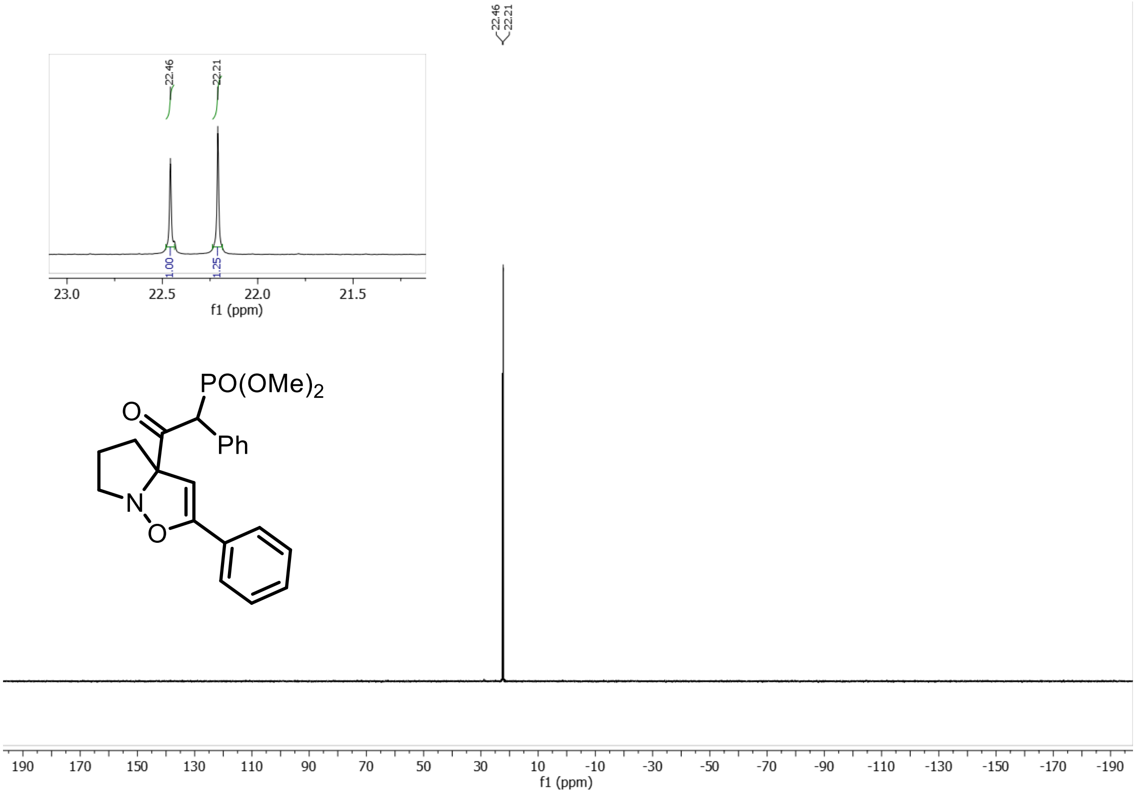

^{1}H NMR Spectra for Compound mixture 3k

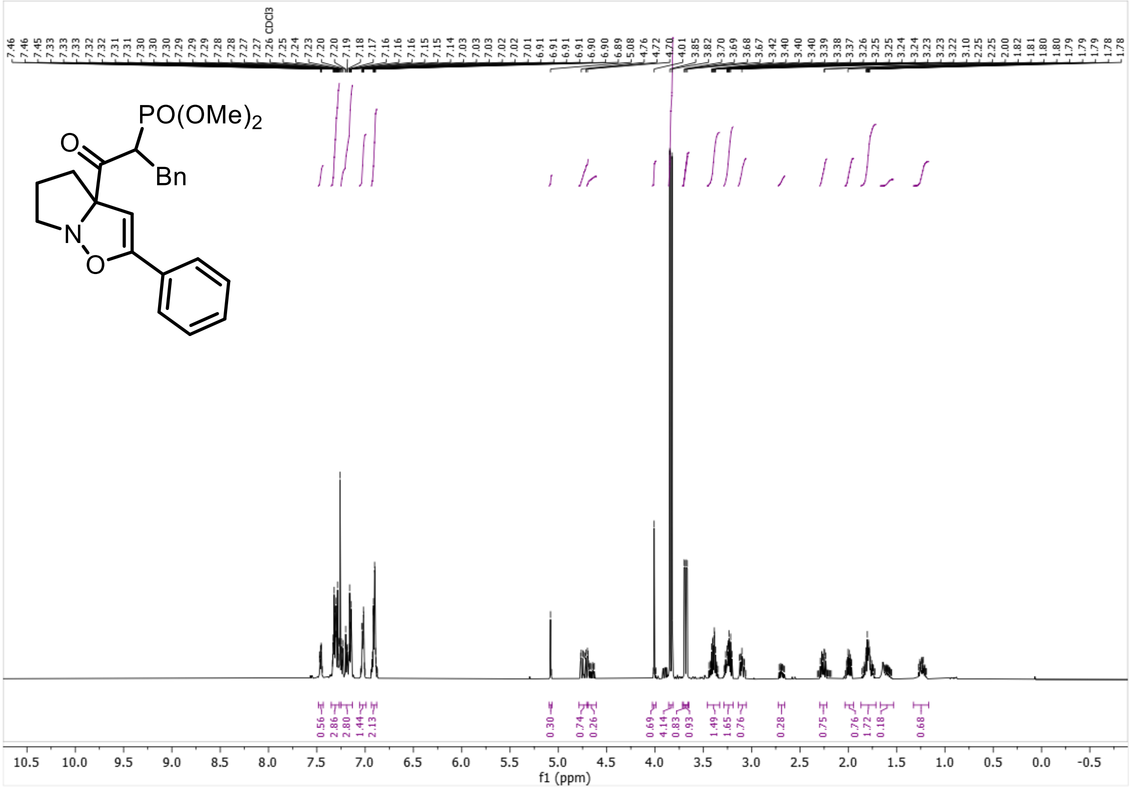

^{13}C NMR Spectra for Compound mixture 3k

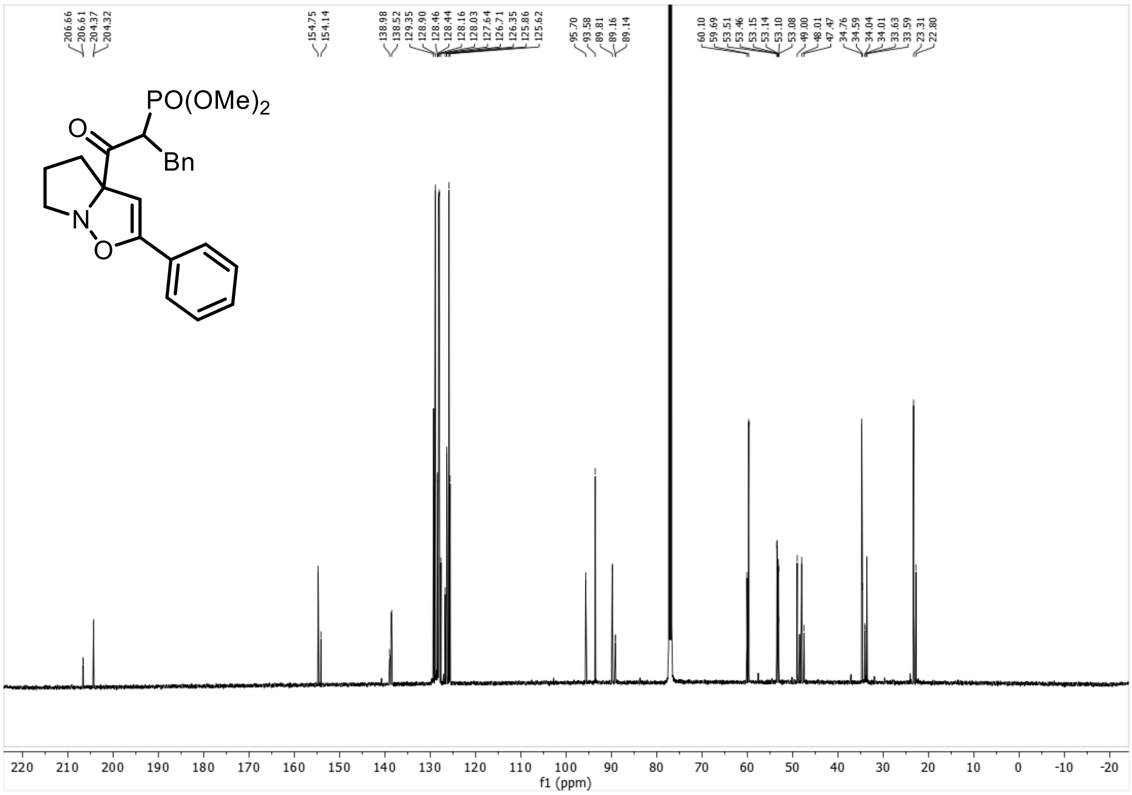

^{31}P NMR Spectra for Compound mixture 3k

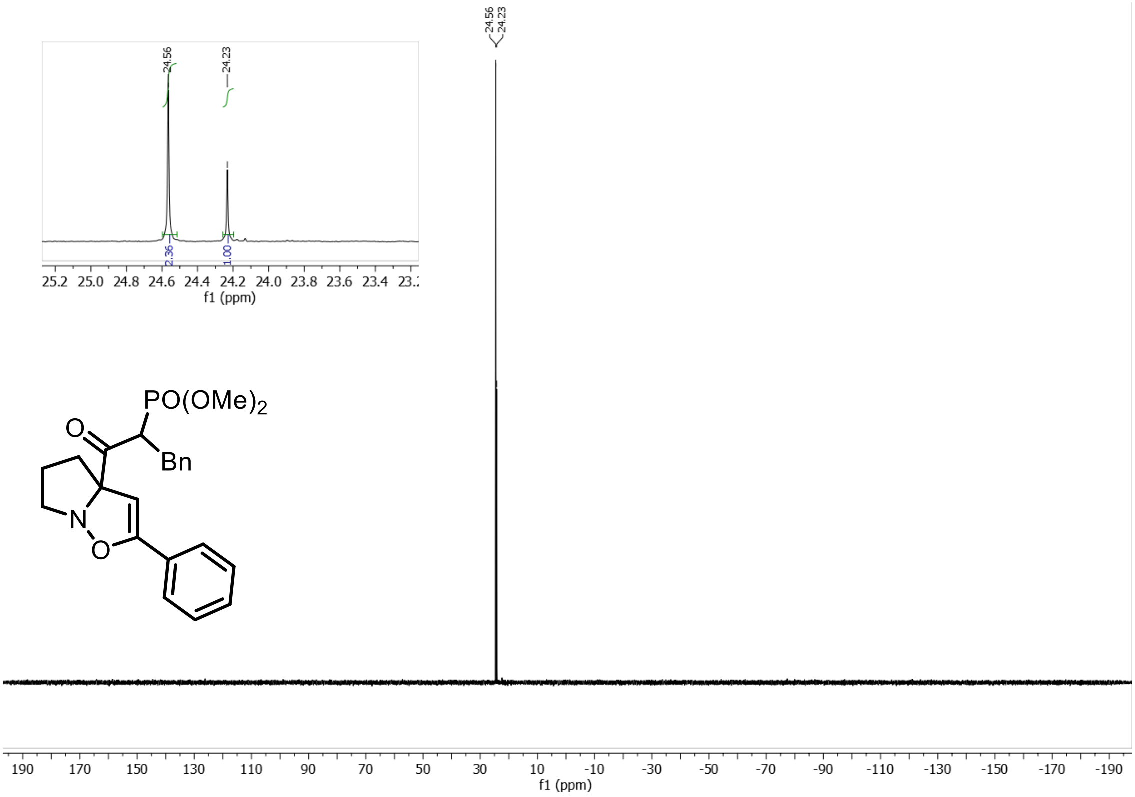

^{1}H NMR Spectra for Compound mixture 3l

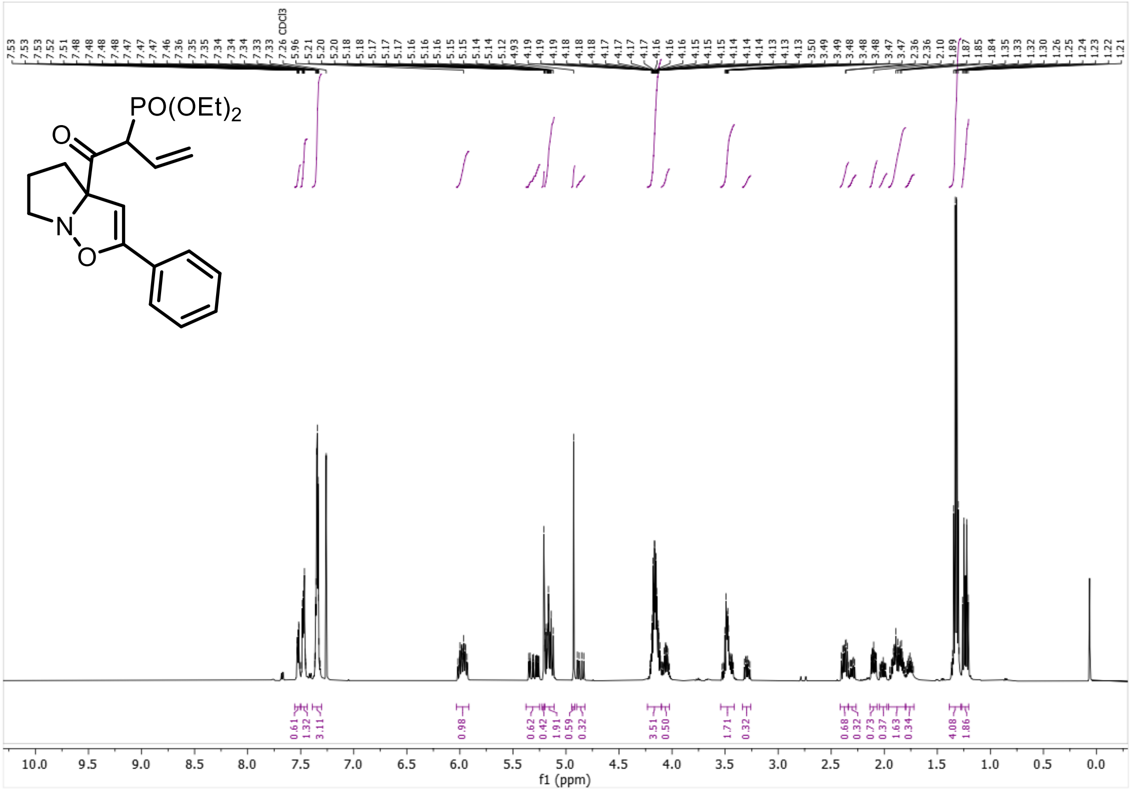

^{13}C NMR Spectra for Compound mixture 3l

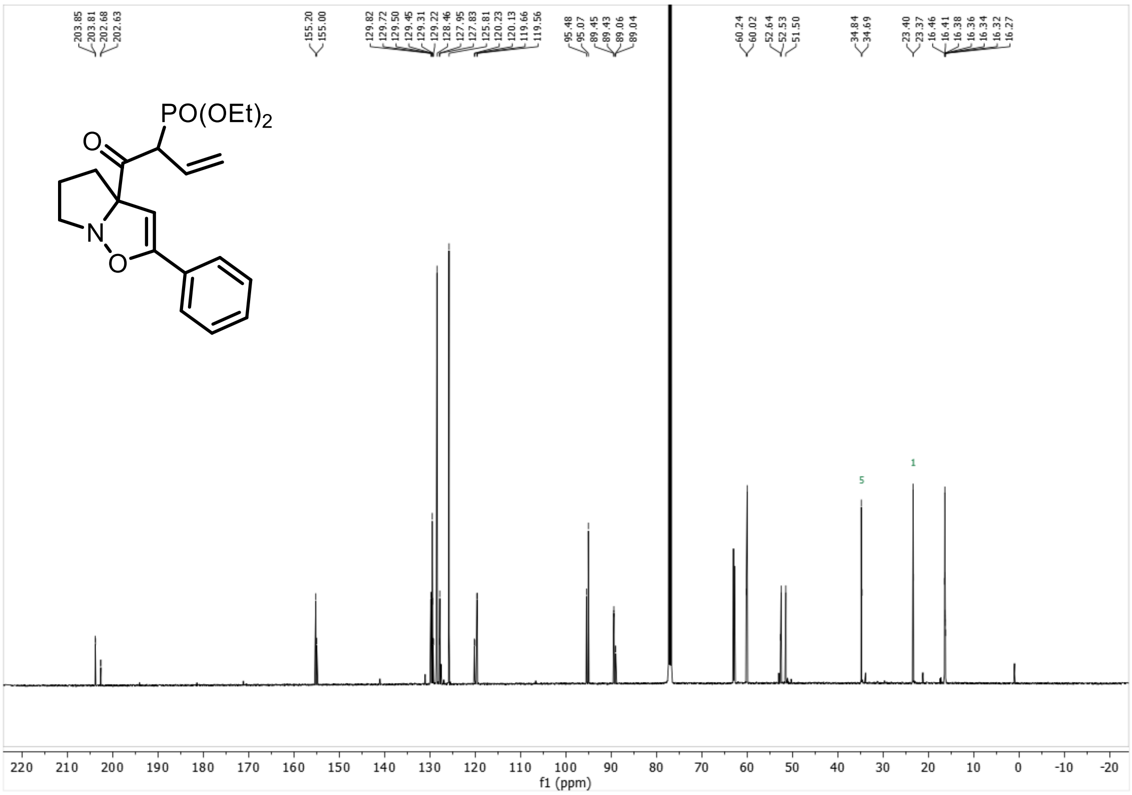

^{31}P NMR Spectra for Compound mixture 3l

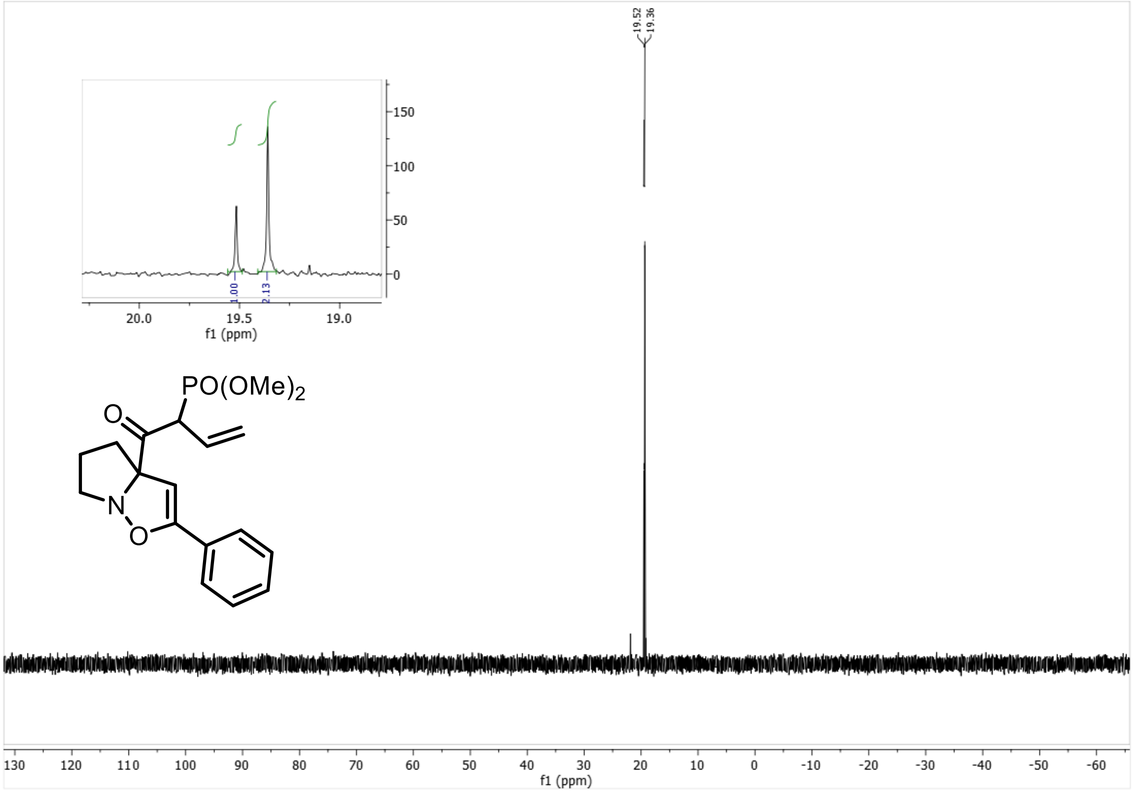

^{1}H NMR Spectra for Compound mixture 3m

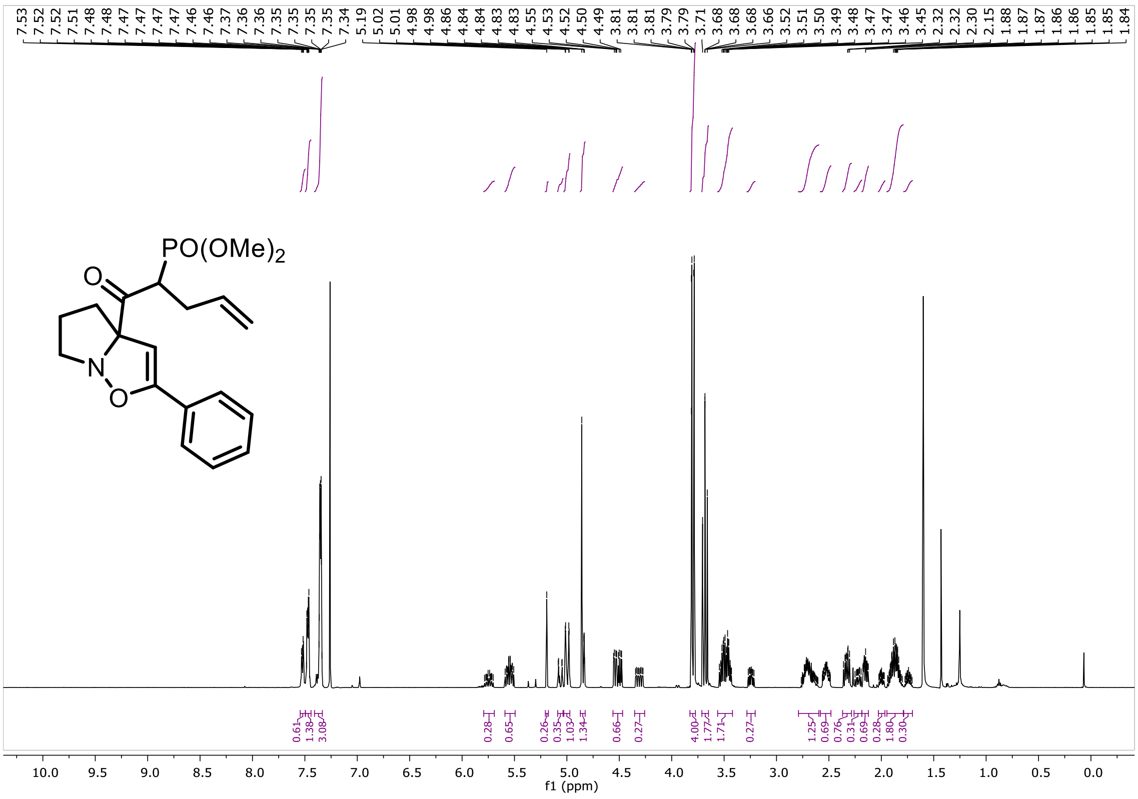

^{13}C NMR Spectra for Compound mixture 3m

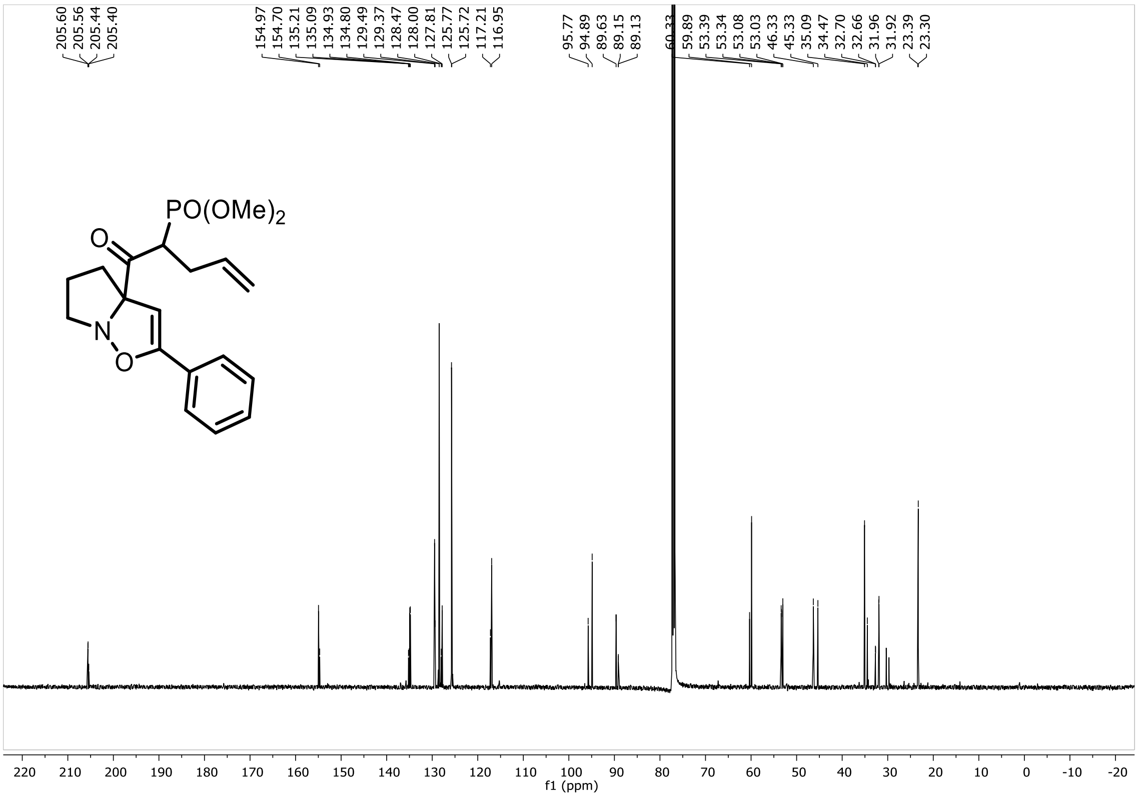

^{31}P NMR Spectra for Compound mixture 3m

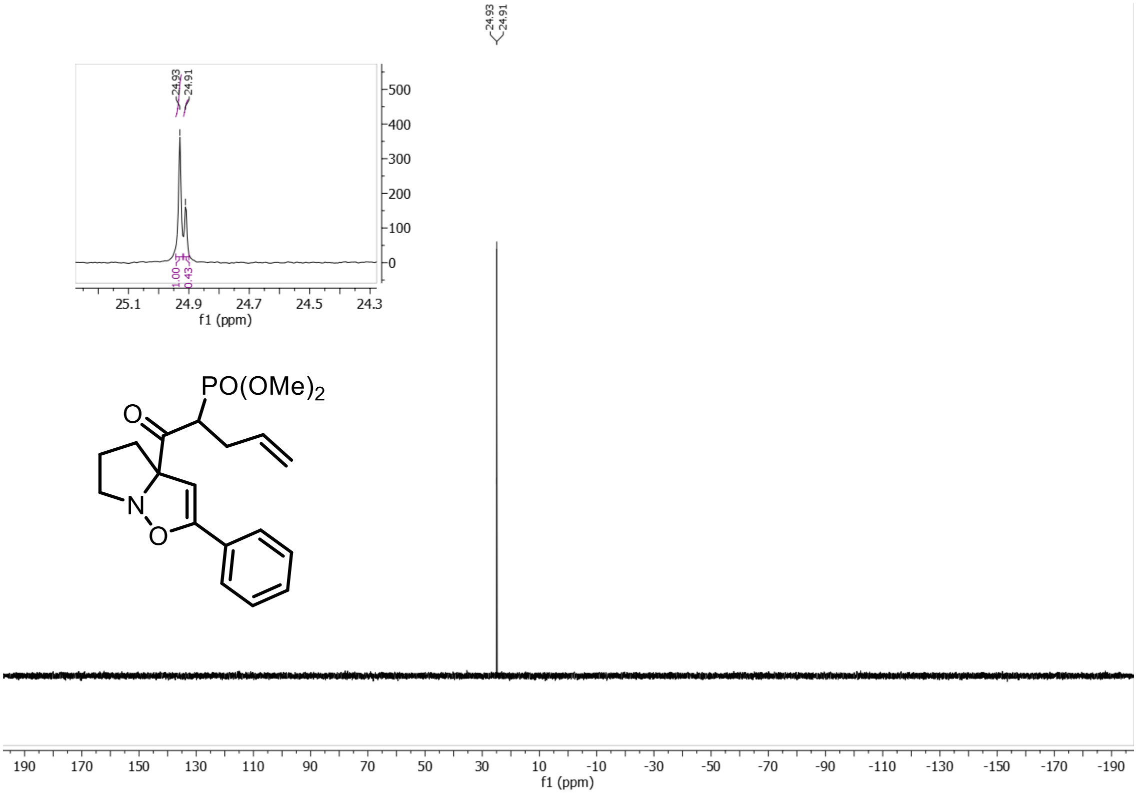

^{1}H NMR Spectra for Compound mixture 3n

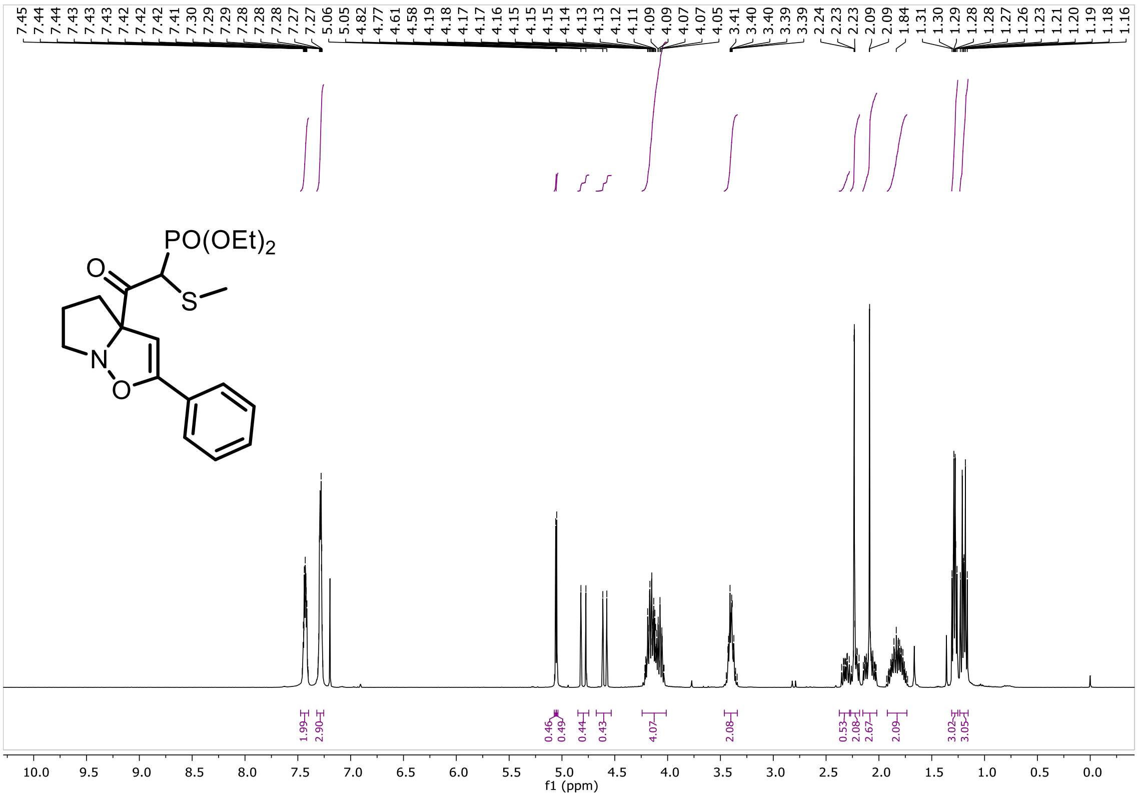

^{13}C NMR Spectra for Compound mixture 3n

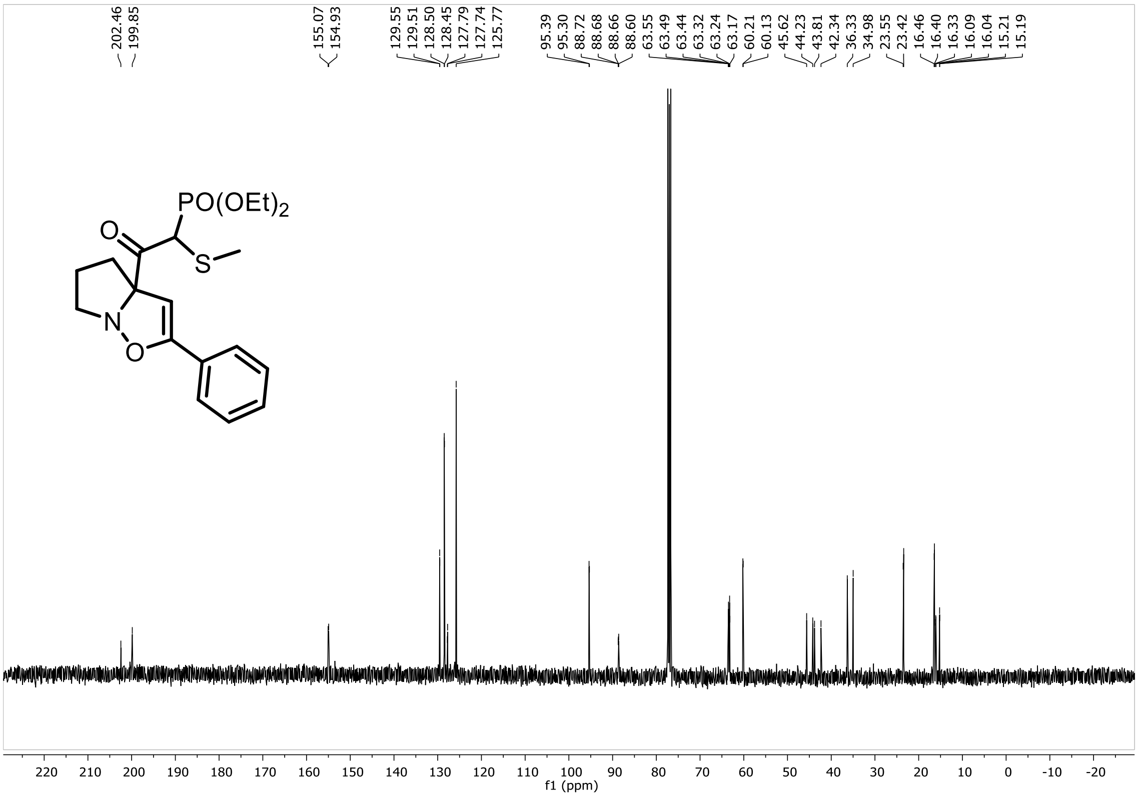

^{31}P NMR Spectra for Compound mixture 3n

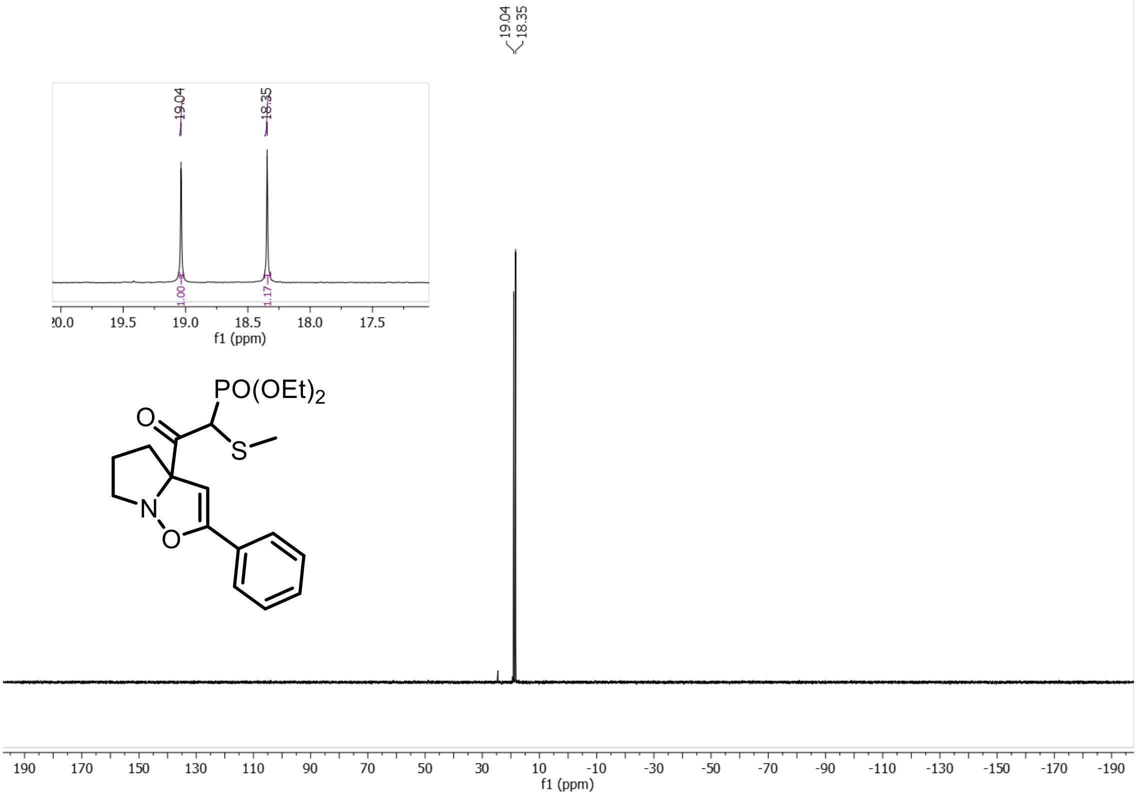

^{1}H NMR Spectra for Compound mixture 3o

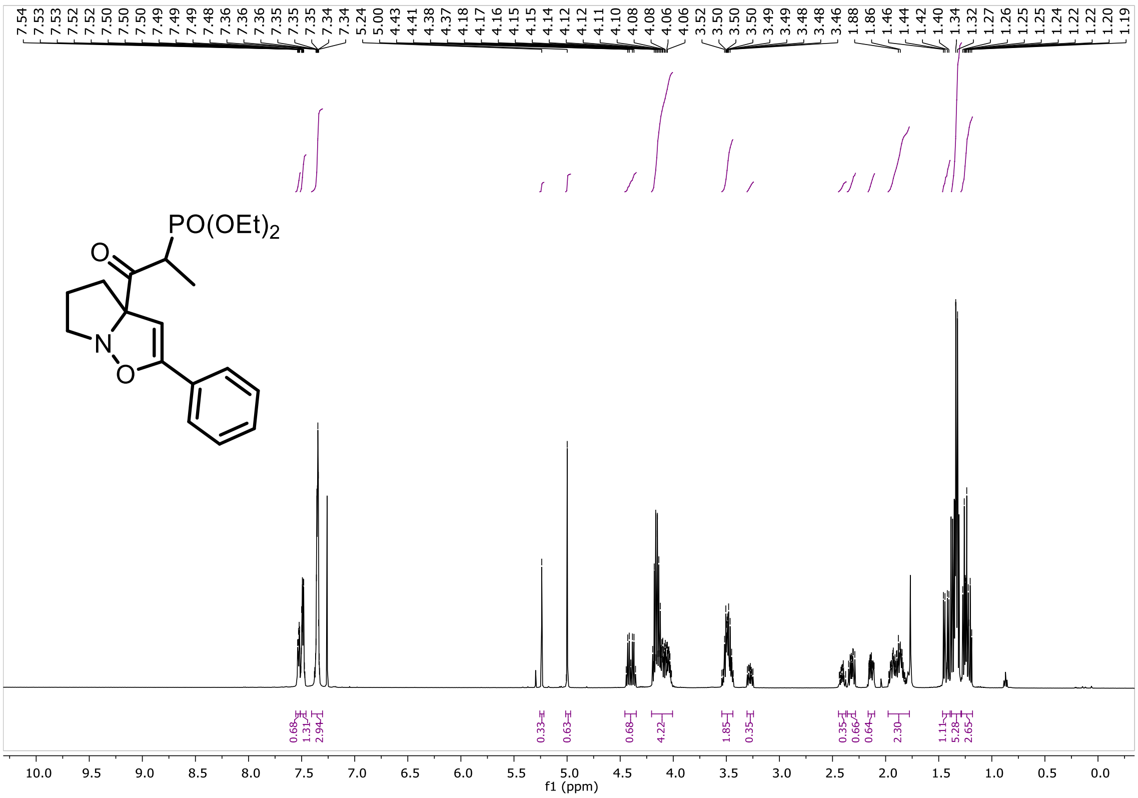

^{13}C NMR Spectra for Compound mixture 3o

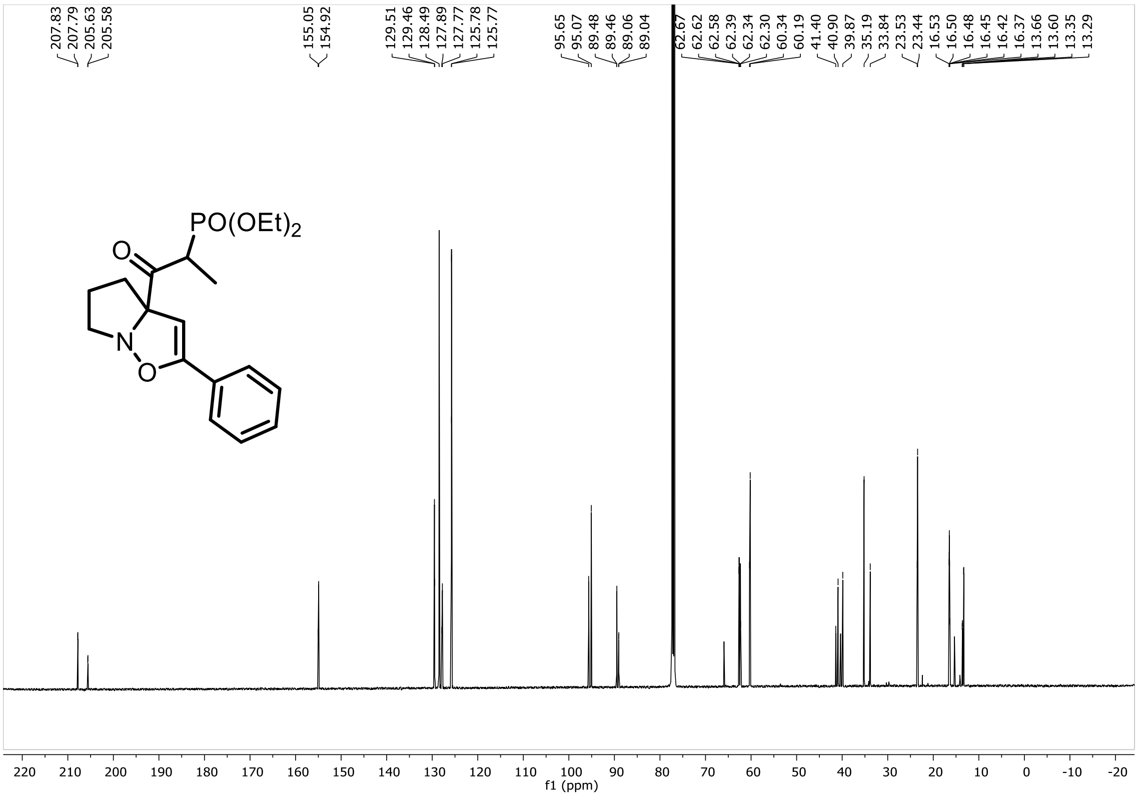

^{31}P NMR Spectra for Compound mixture 3o

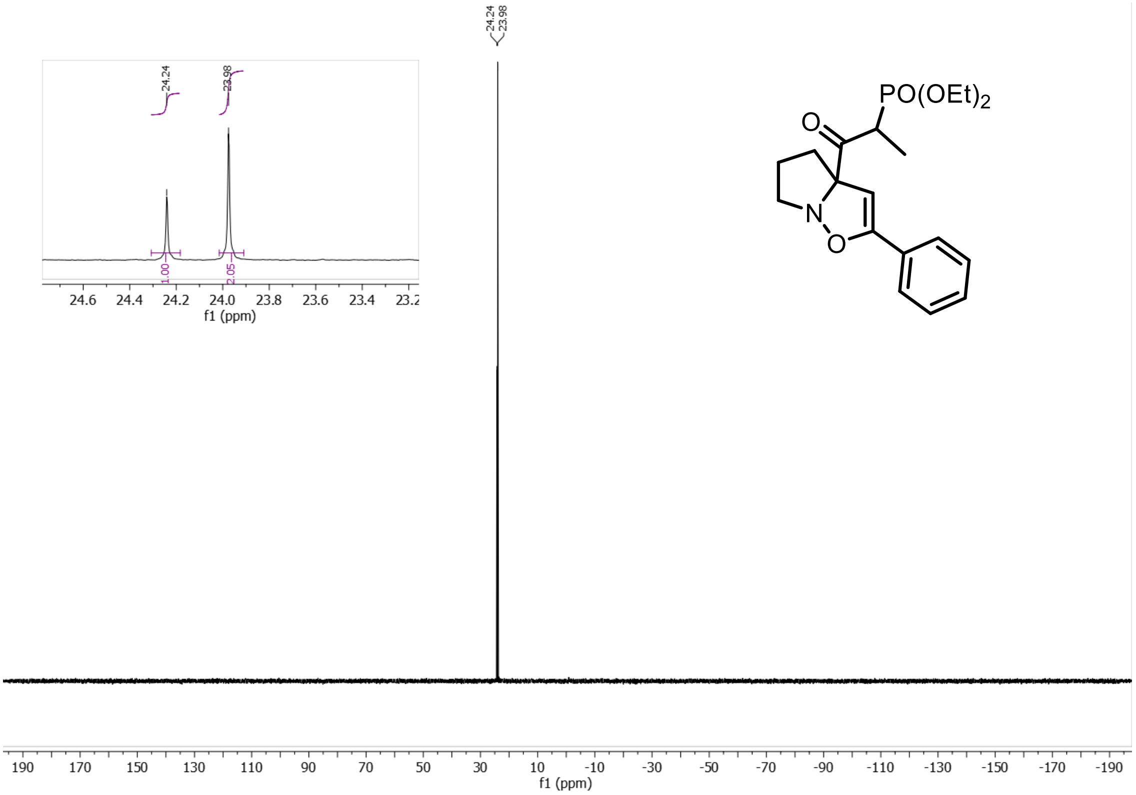

^{1}H NMR Spectra for Compound 3p

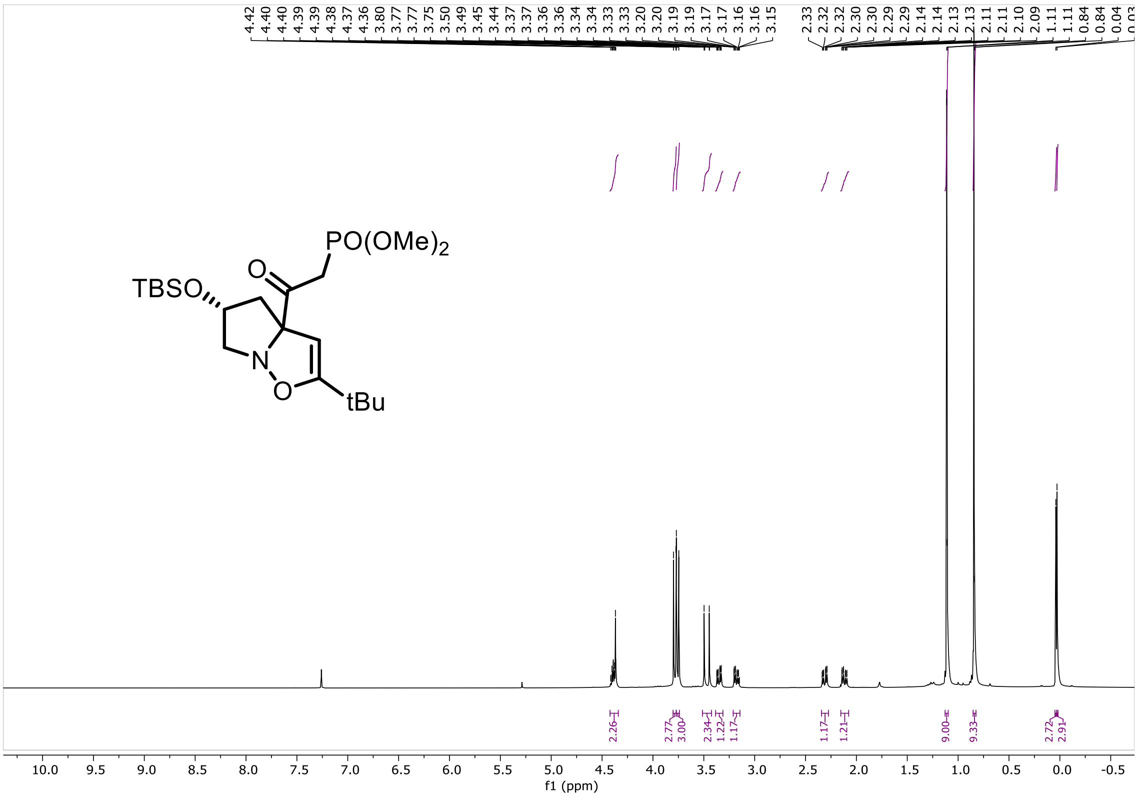

^{13}C NMR Spectra for Compound 3p

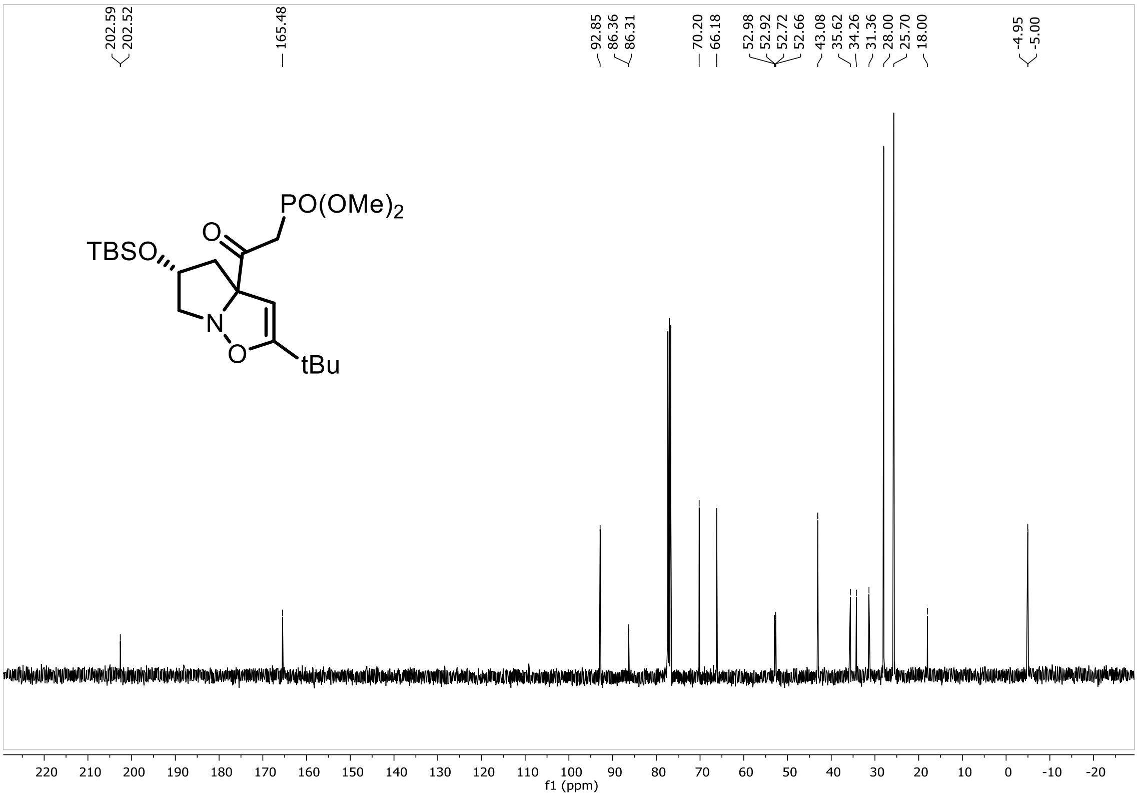

^{31}P NMR Spectra for Compound 3p

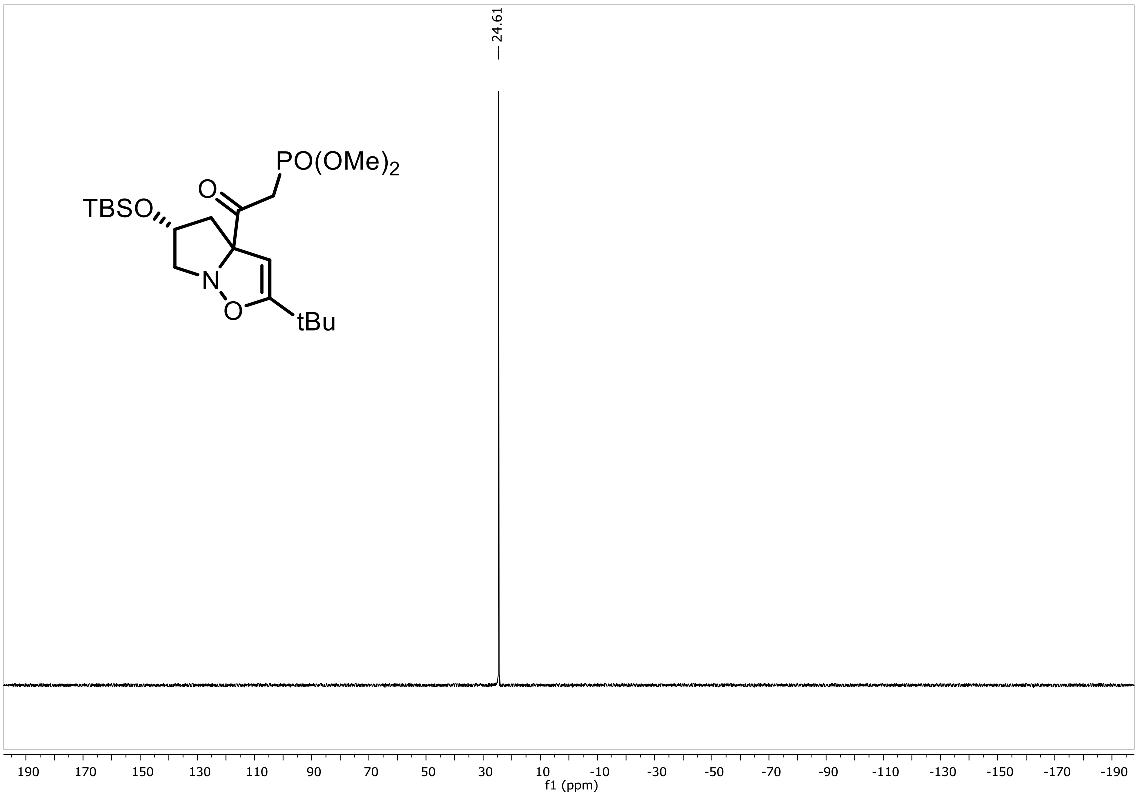

^{1}H NMR Spectra for Compound 3q

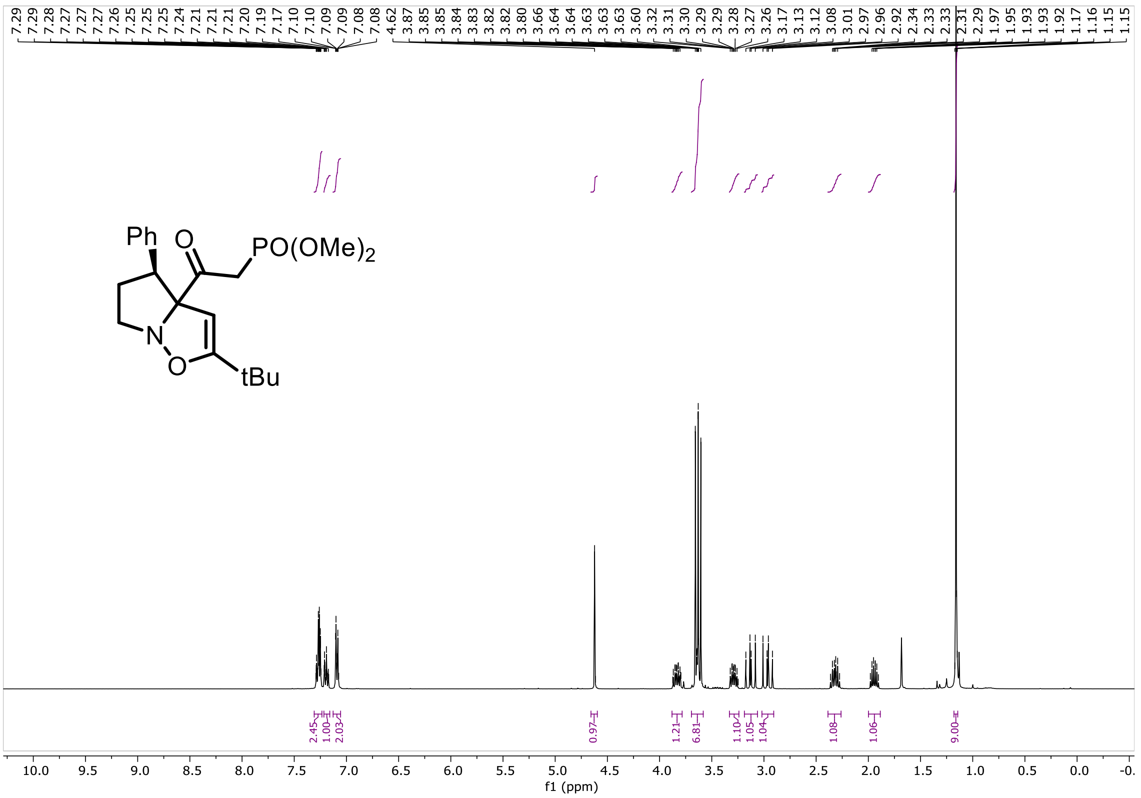

^{13}C NMR Spectra for Compound 3q

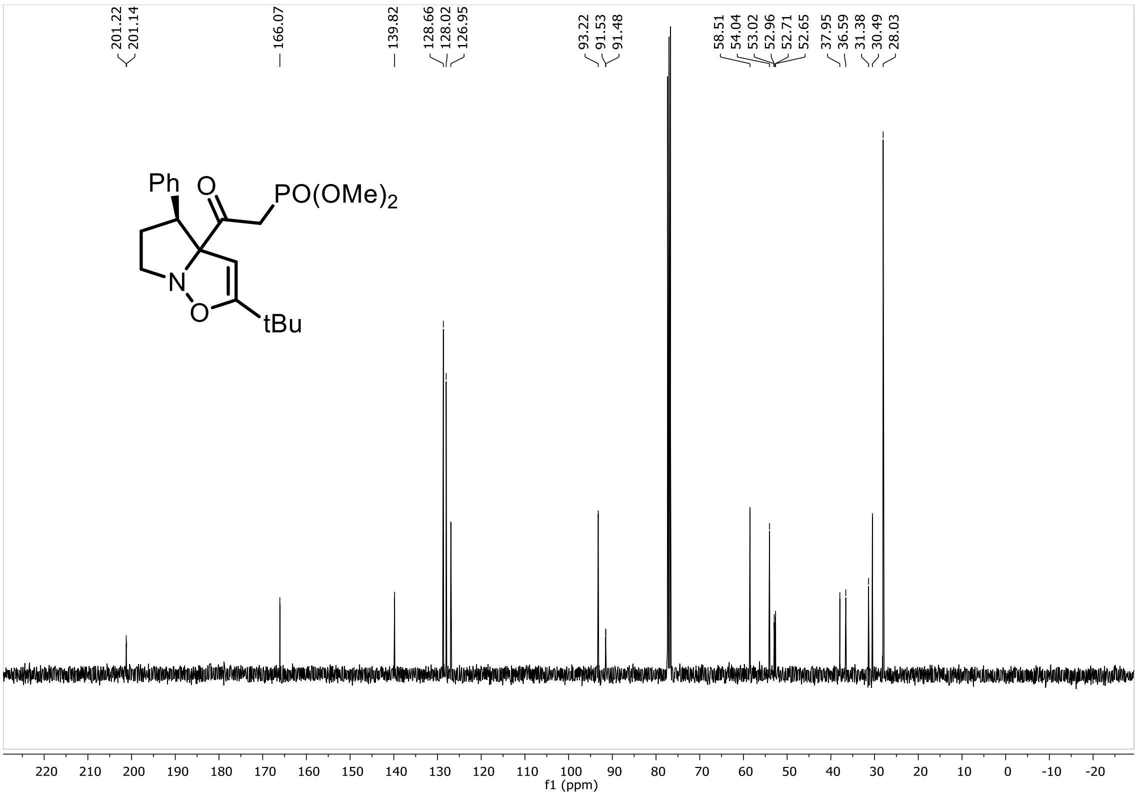

^{31}P NMR Spectra for Compound 3q

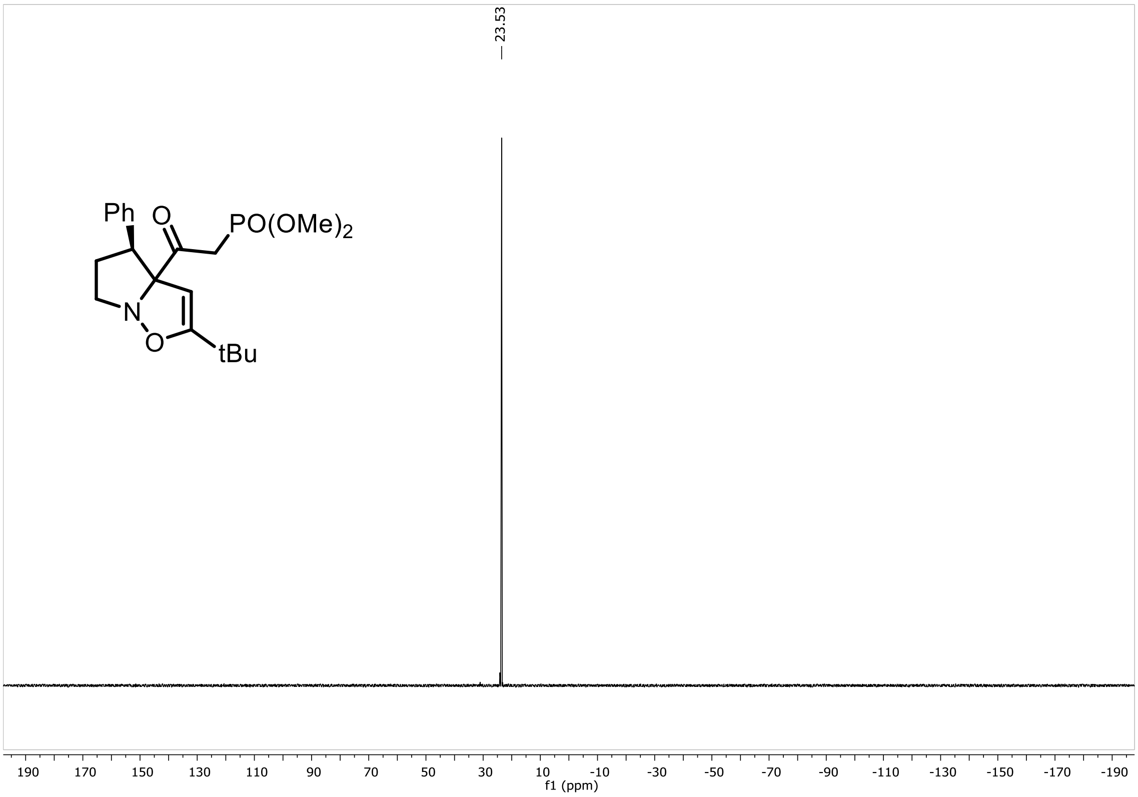

^{1}H NMR Spectra for Compound 3r

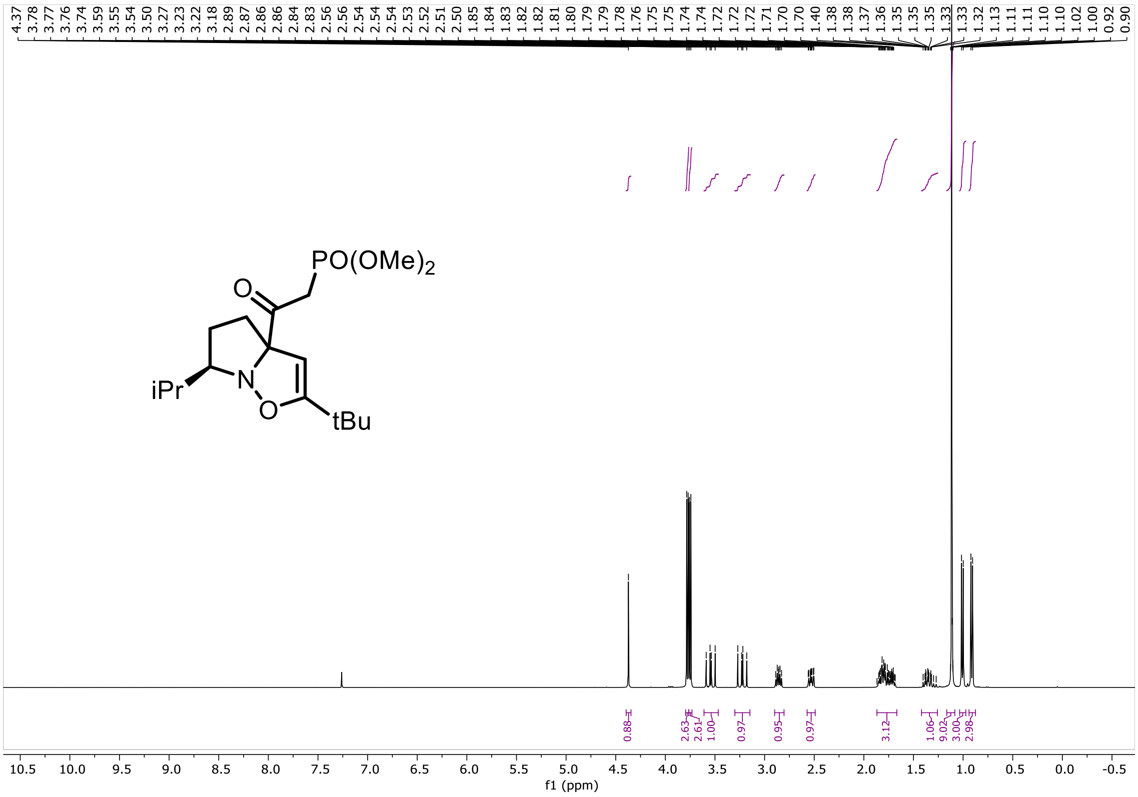

^{13}C NMR Spectra for Compound 3r

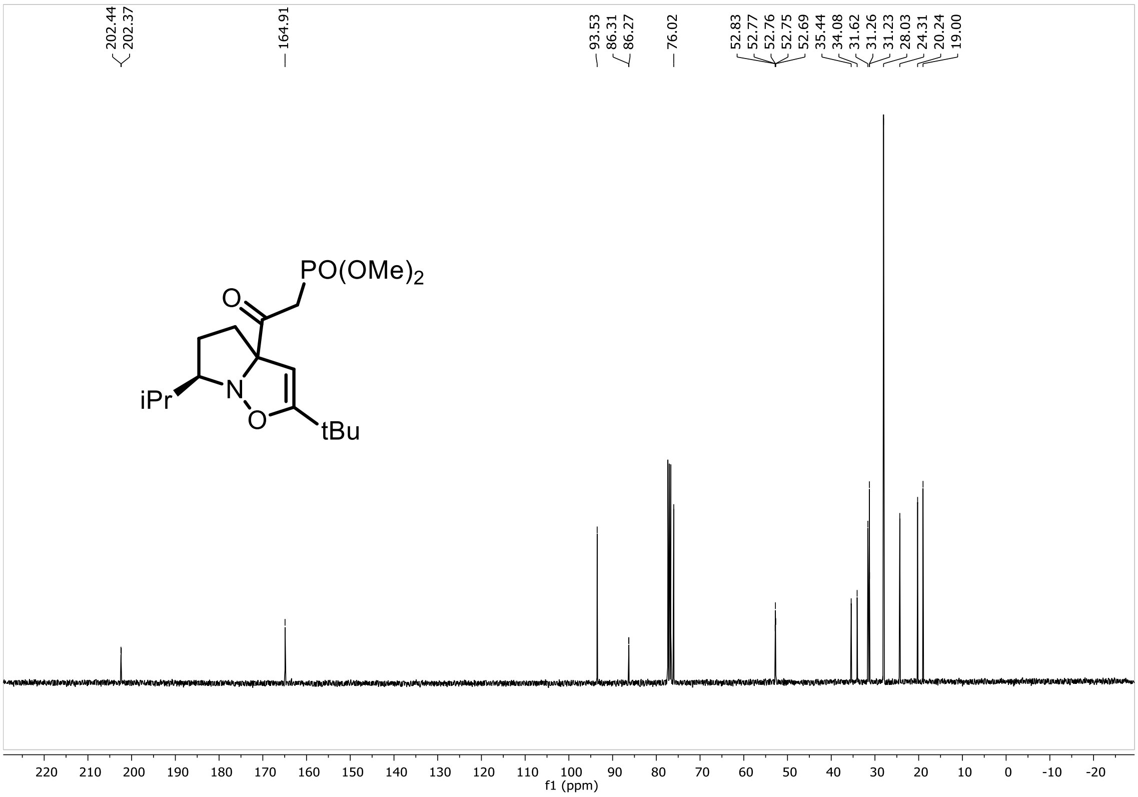

^{31}P NMR Spectra for Compound 3r

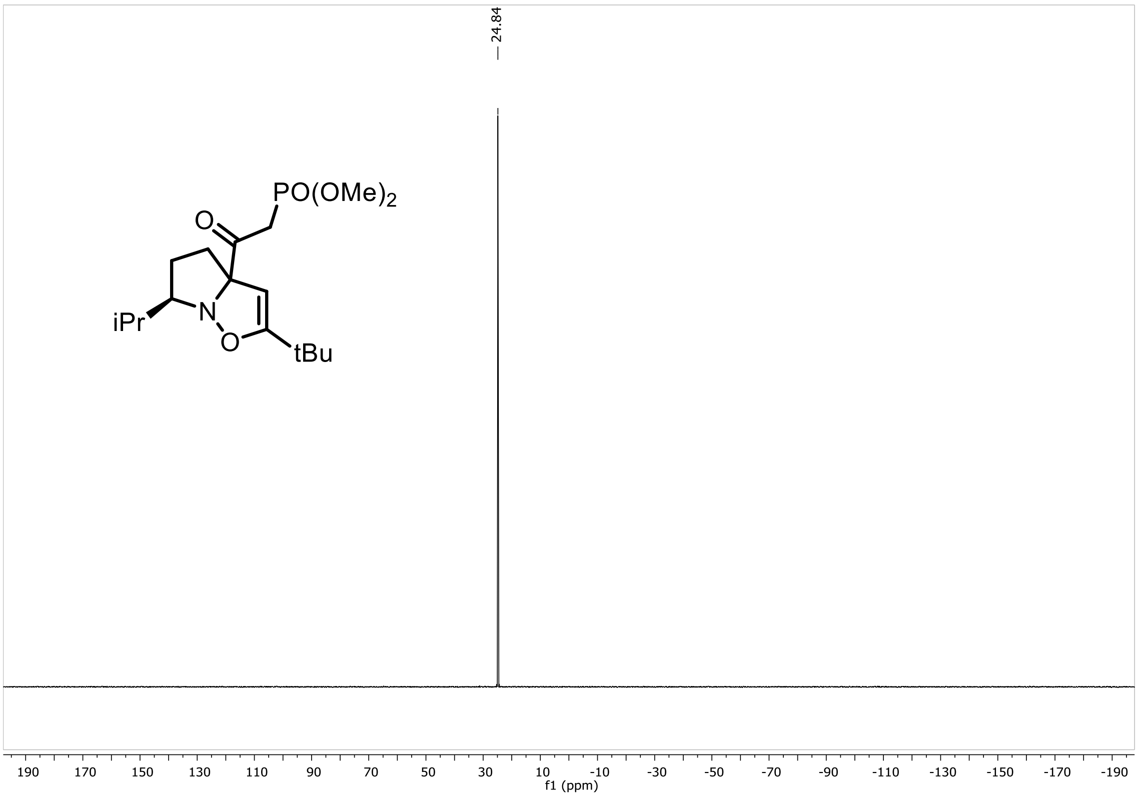

^{1}H NMR Spectra for Compound 4a

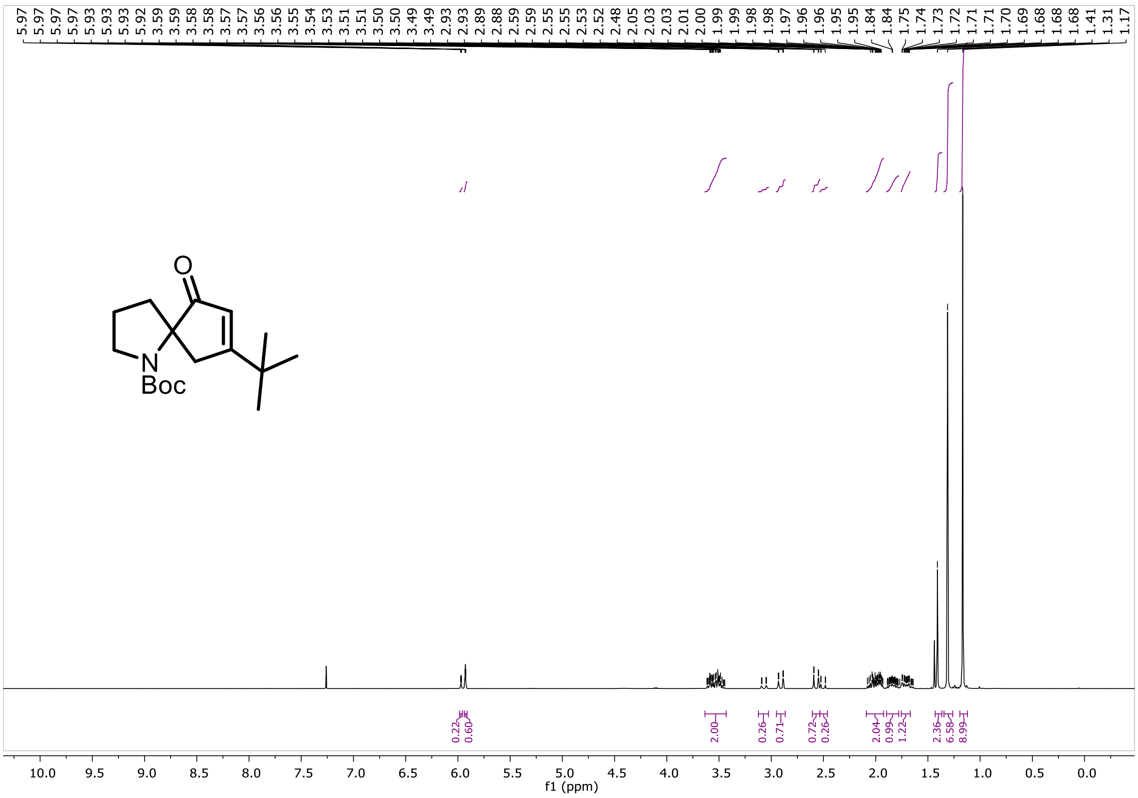

^{13}C NMR Spectra for Compound 4a

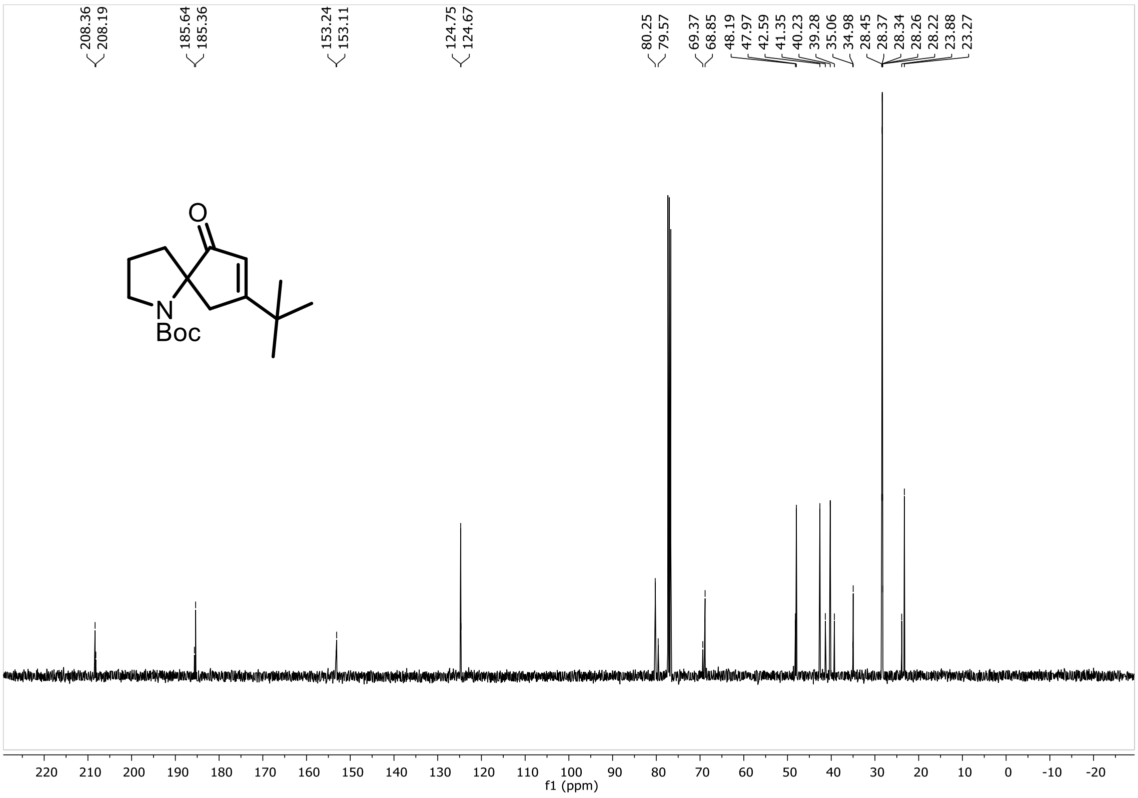

^{1}H NMR Spectra for Compound 4b

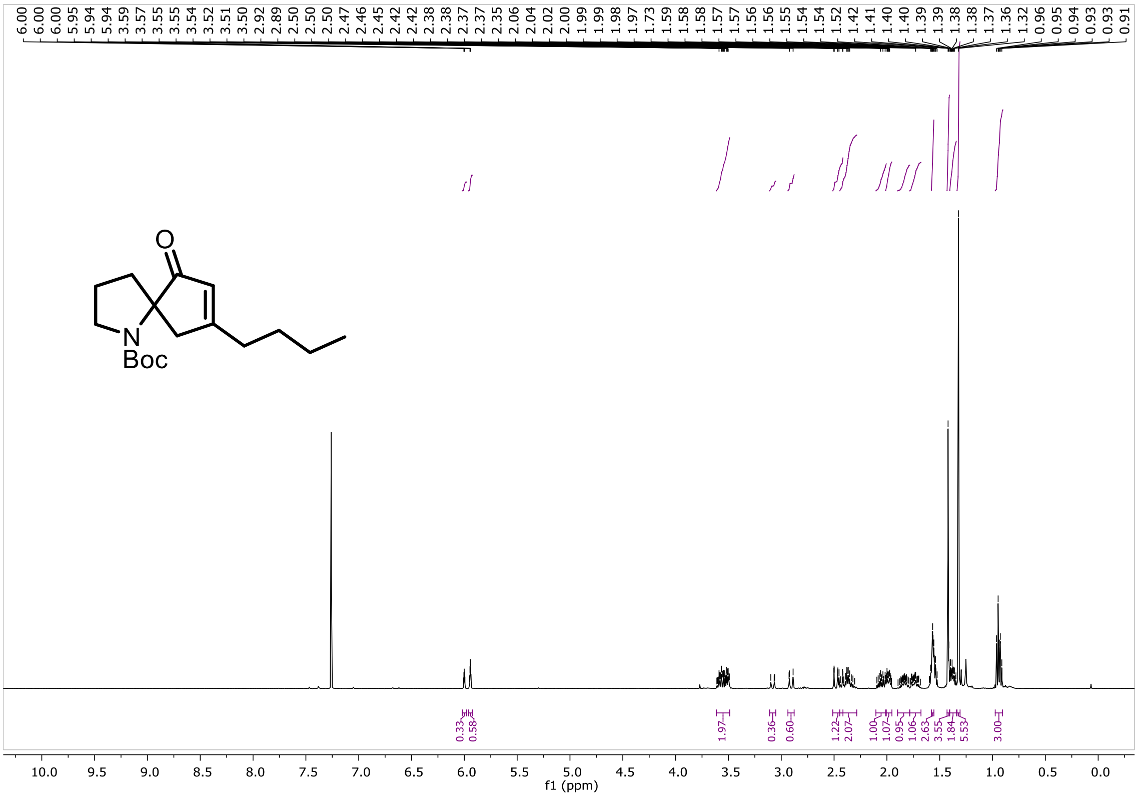

^{13}C NMR Spectra for Compound 4b

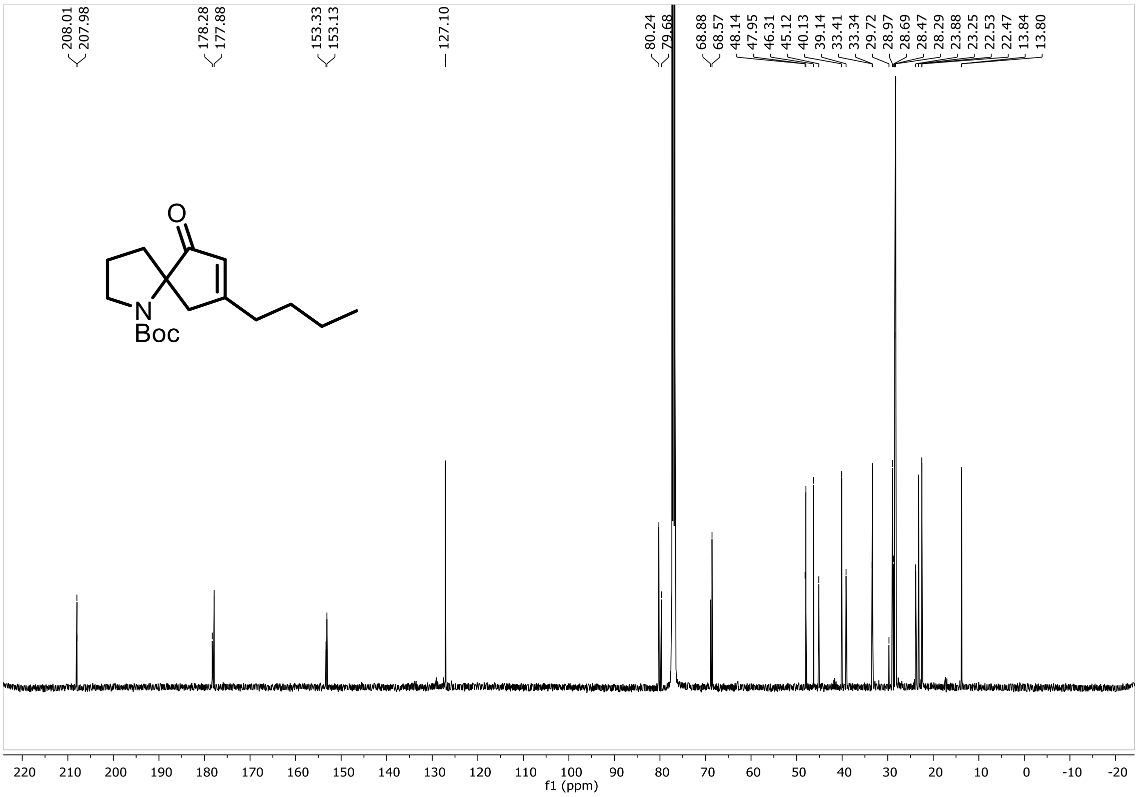

^{1}H NMR Spectra for Compound 4c

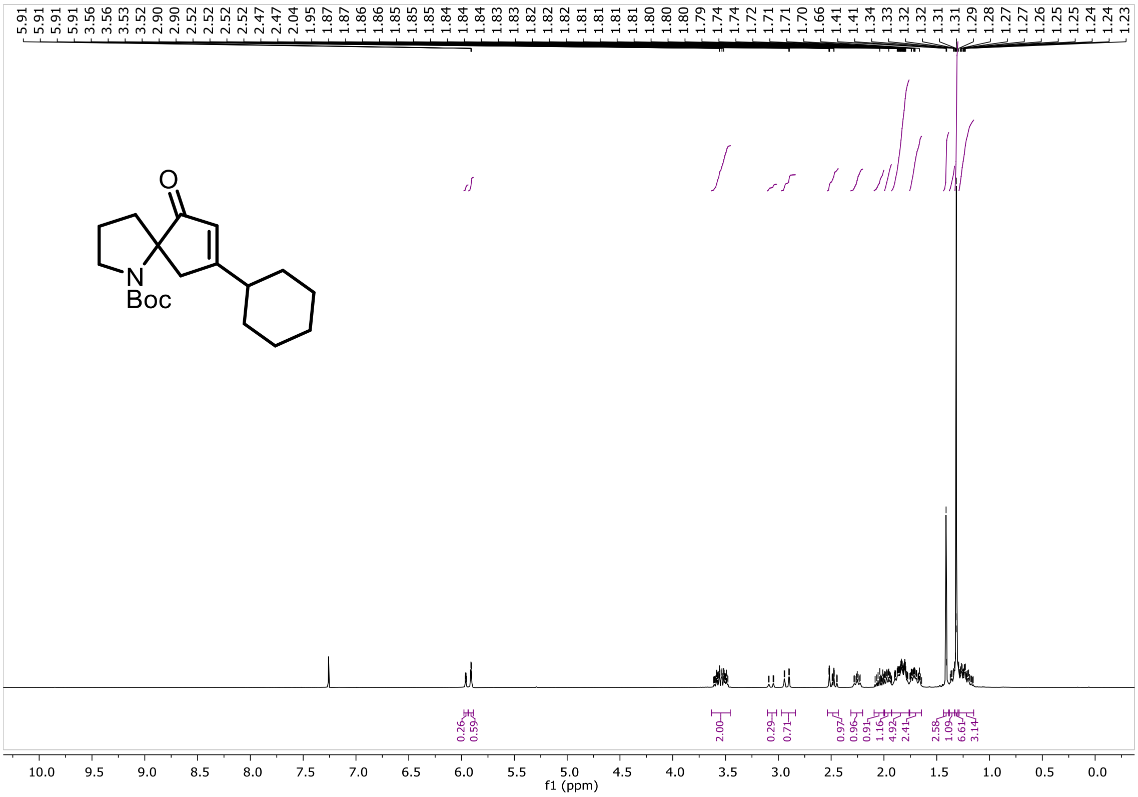

^{13}C NMR Spectra for Compound 4c

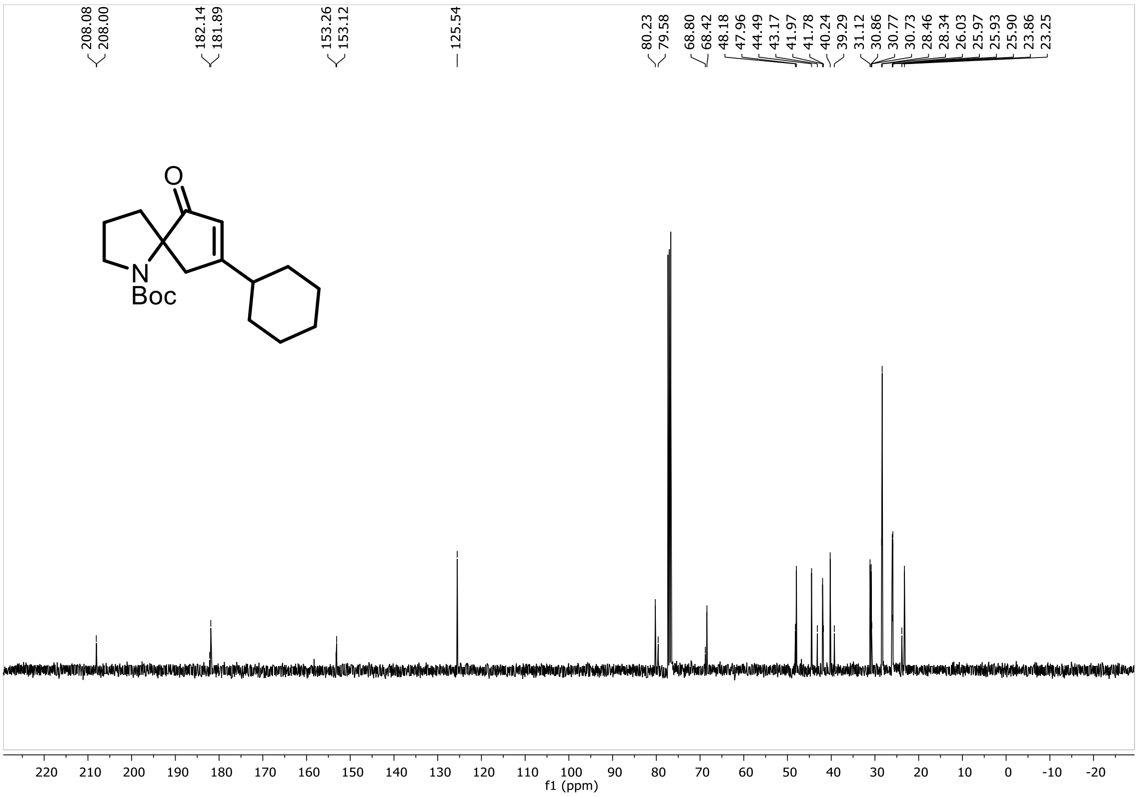

^{1}H NMR Spectra for Compound 4d

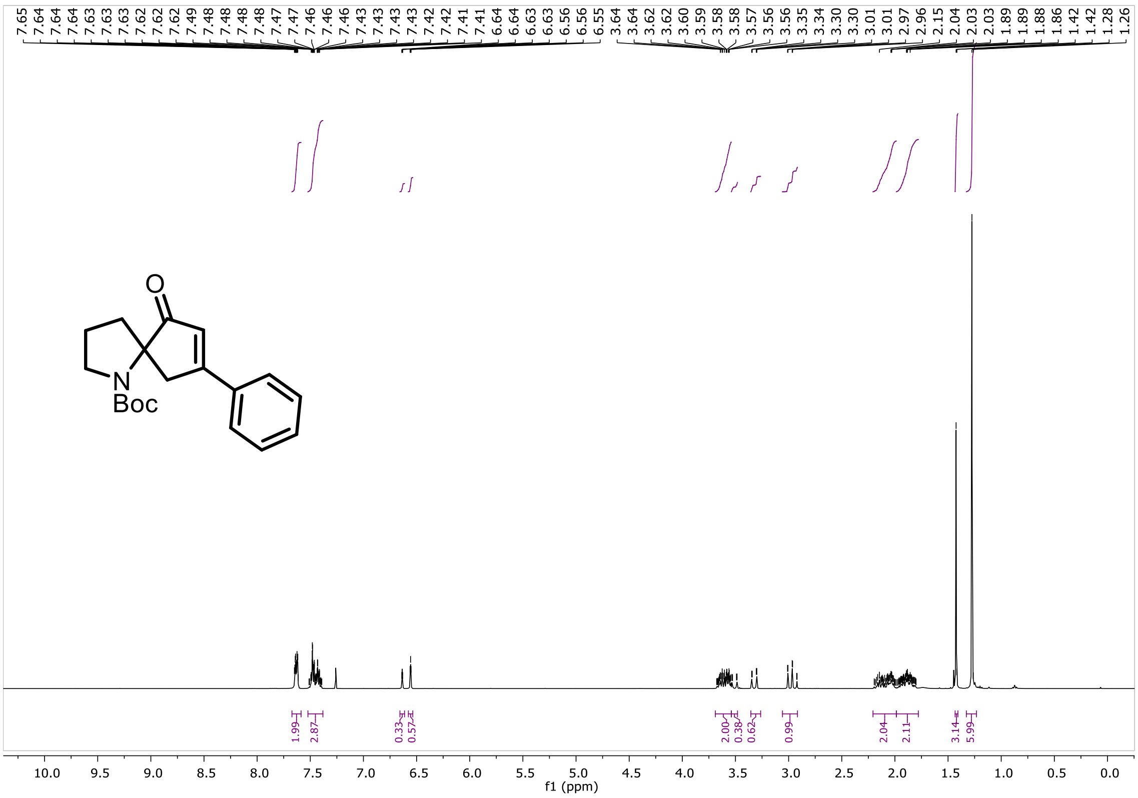

^{13}C NMR Spectra for Compound 4d

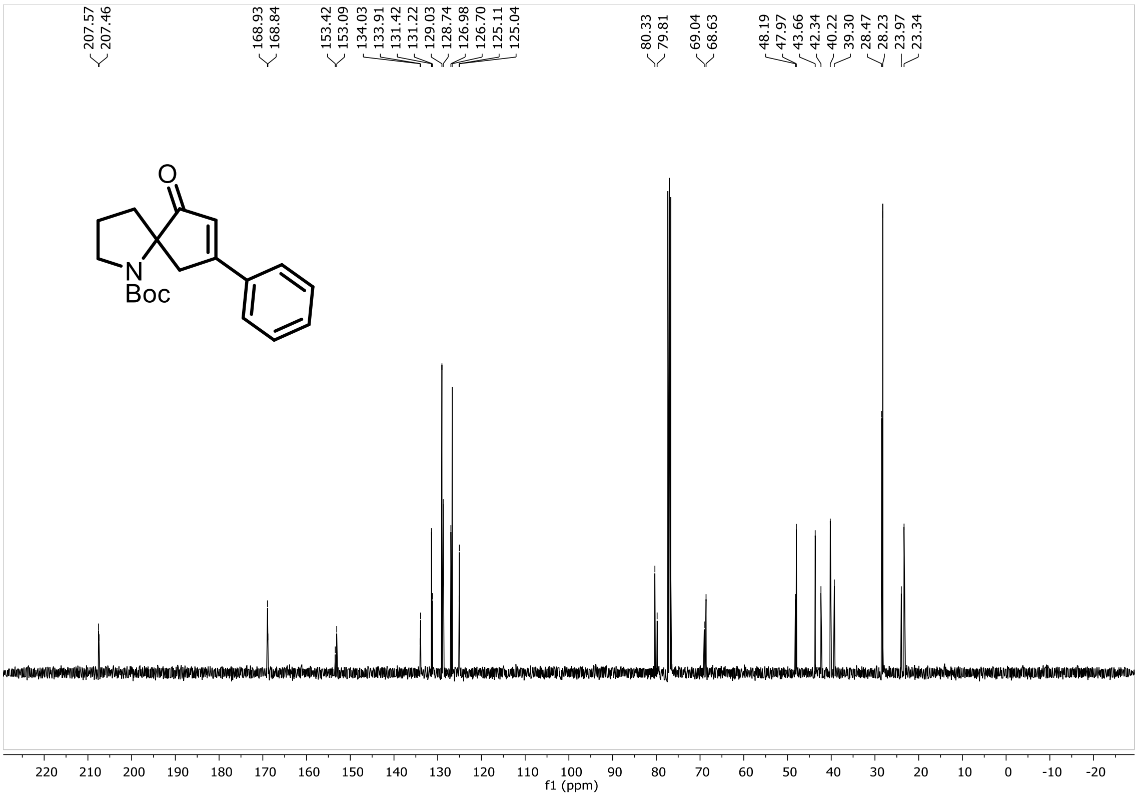

^{1}H NMR Spectra for Compound 4e

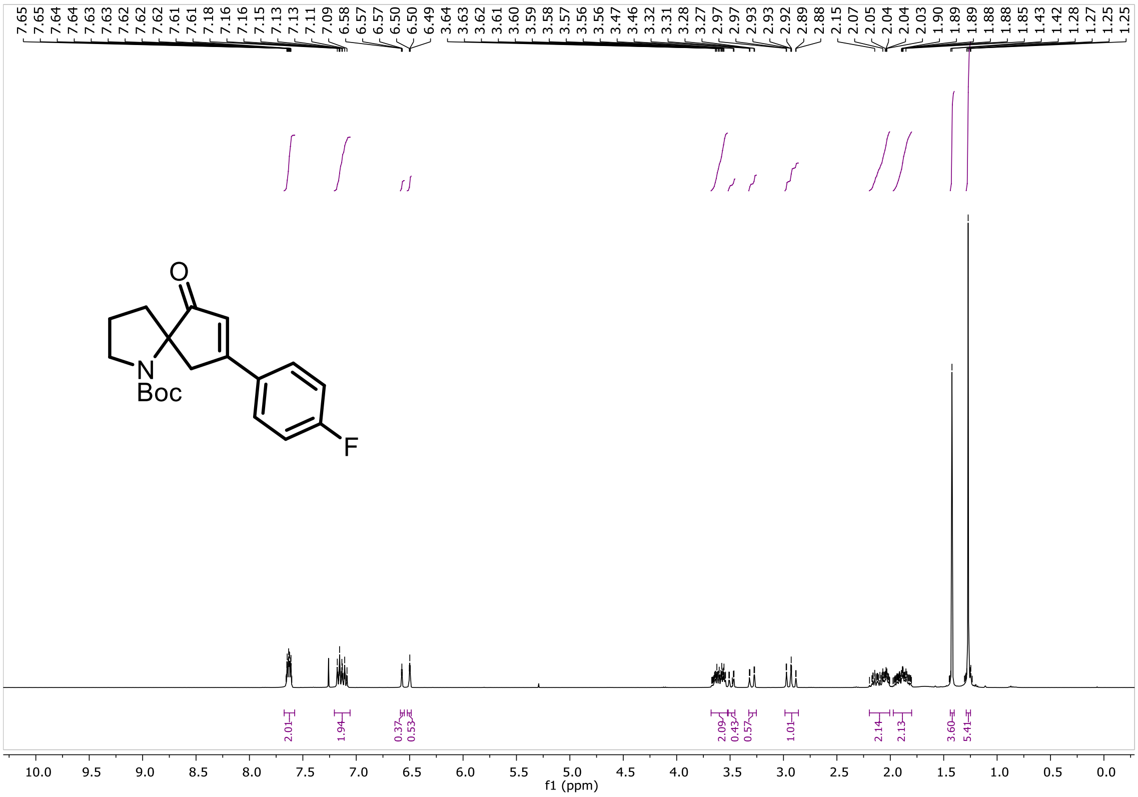

^{13}C NMR Spectra for Compound 4e

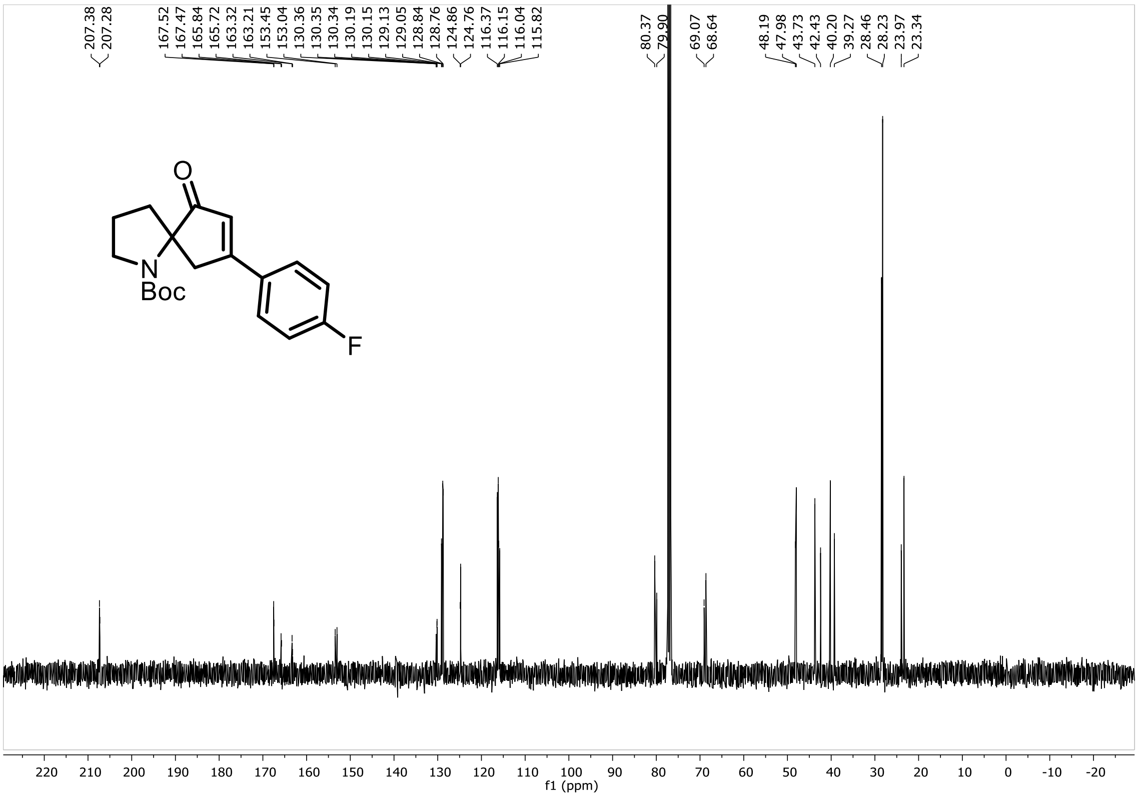

^{19}F NMR Spectra for Compound 4e

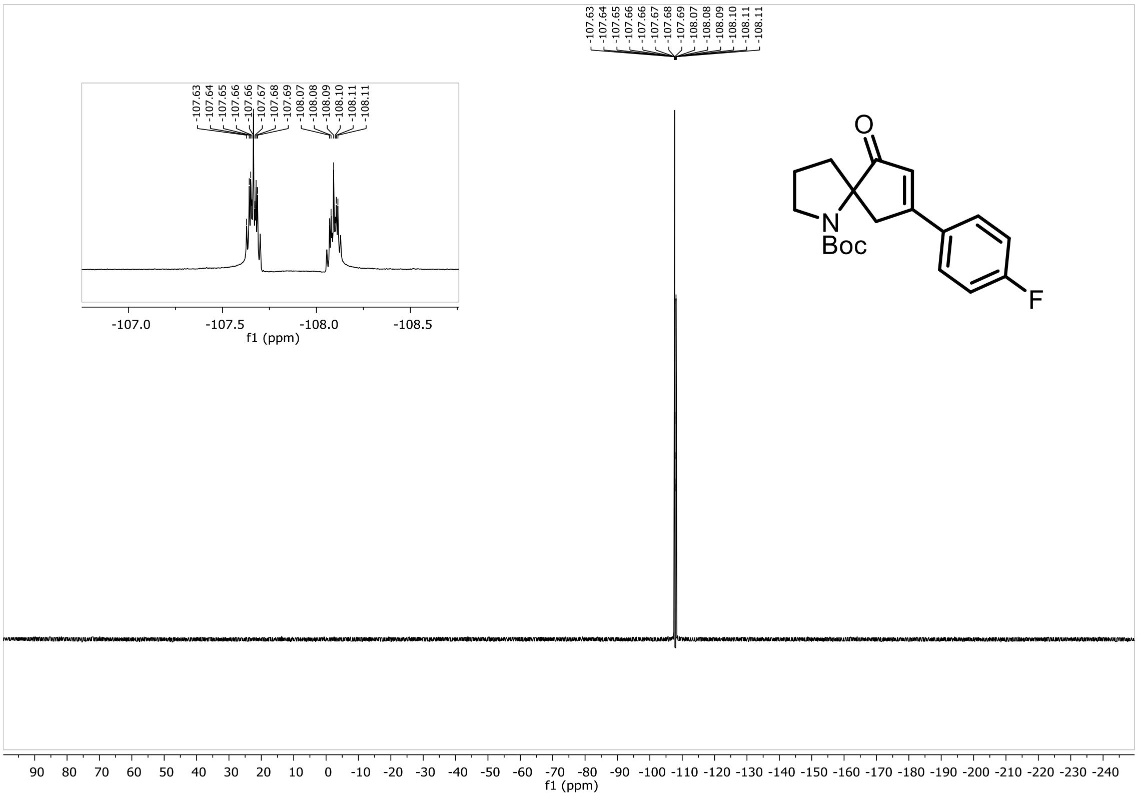

^{1}H NMR Spectra for Compound 4f

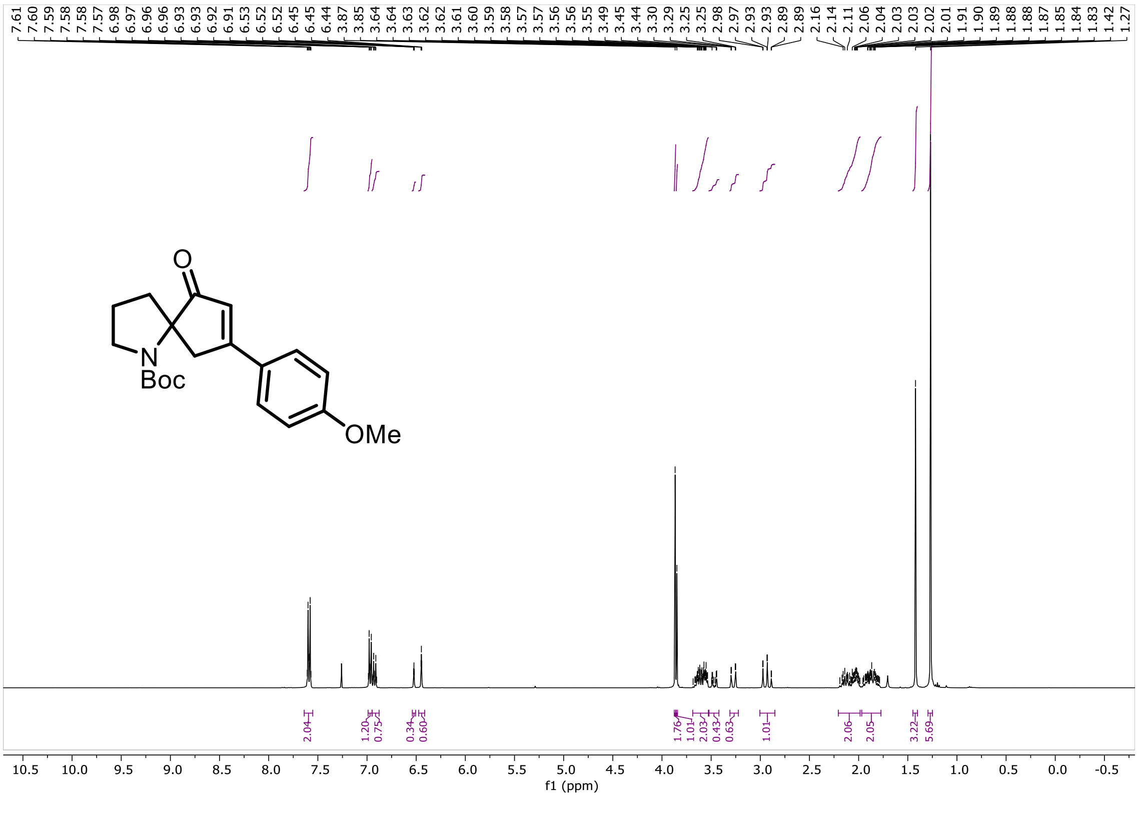

^{13}C NMR Spectra for Compound 4f

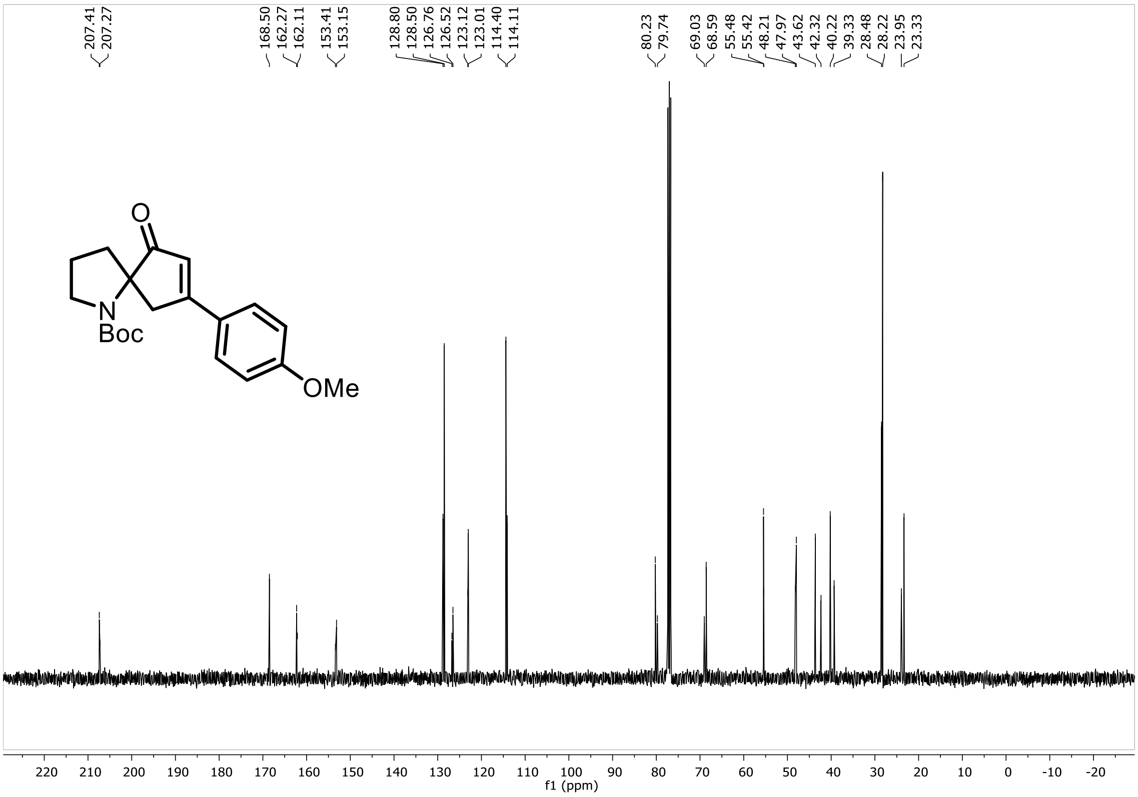

^{1}H NMR Spectra for Compound 4g

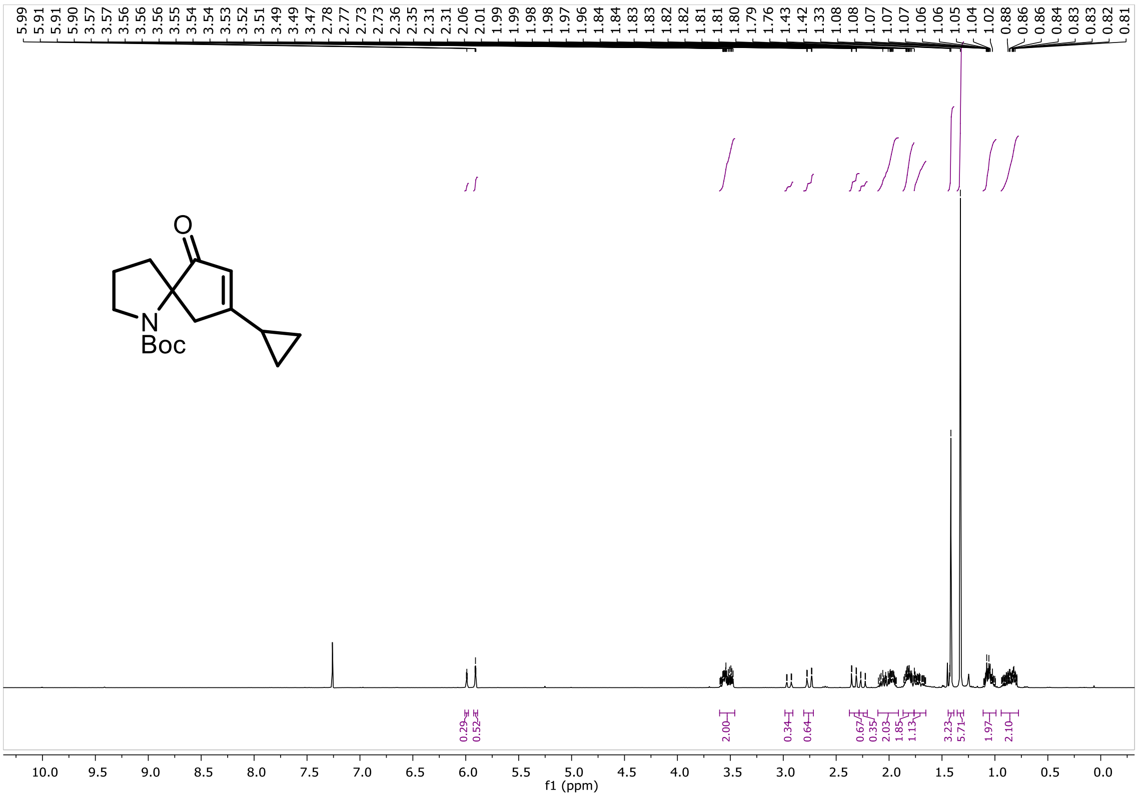

^{13}C NMR Spectra for Compound 4g

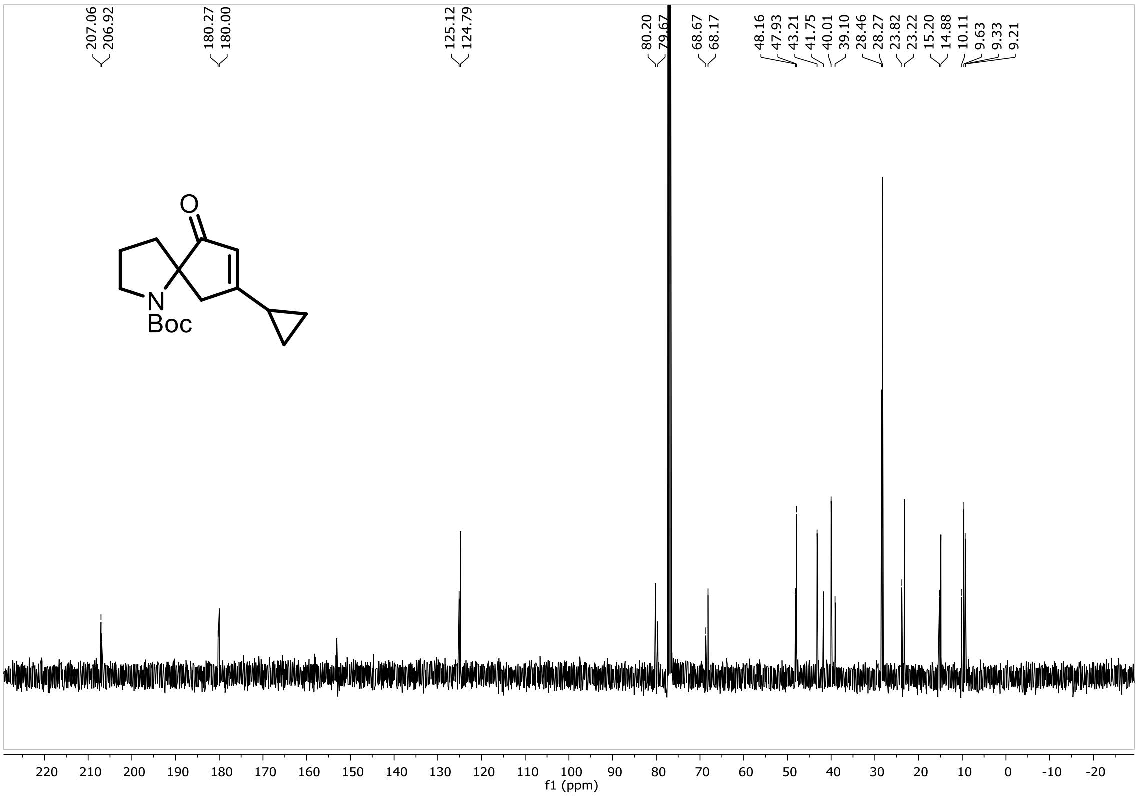

^{1}H NMR Spectra for Compound 4h

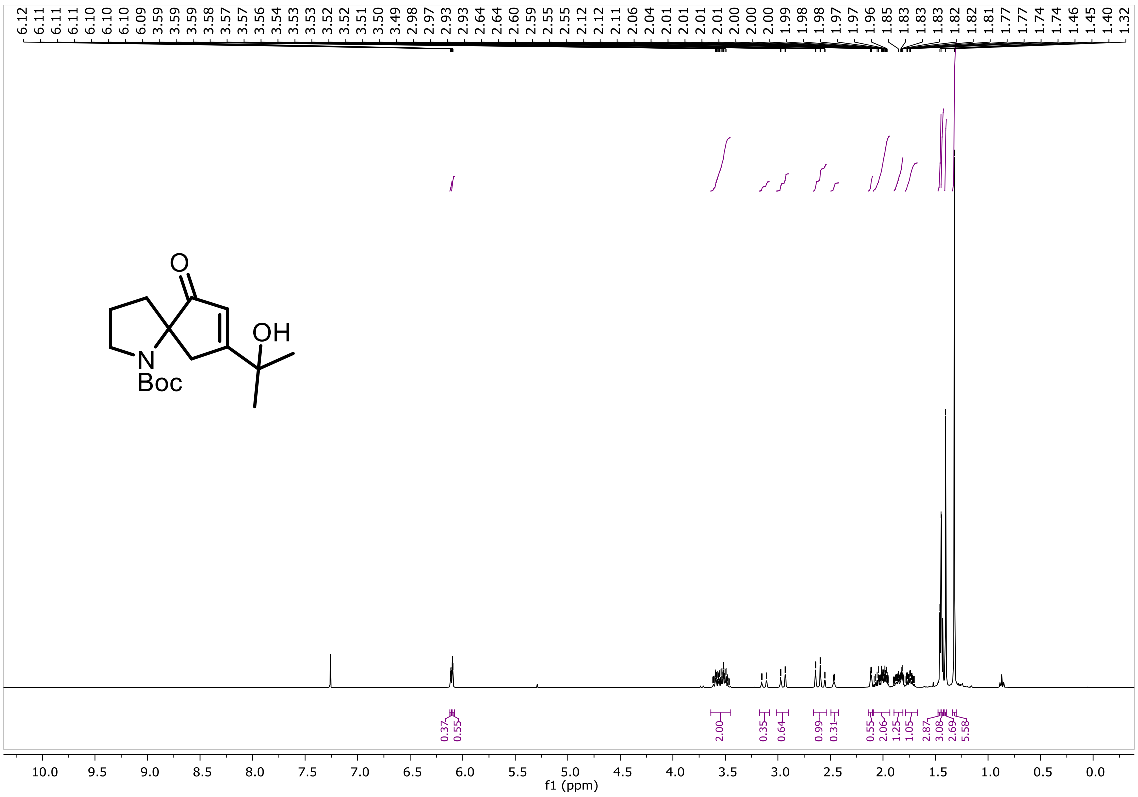

13C NMR Spectra for Compound 4h

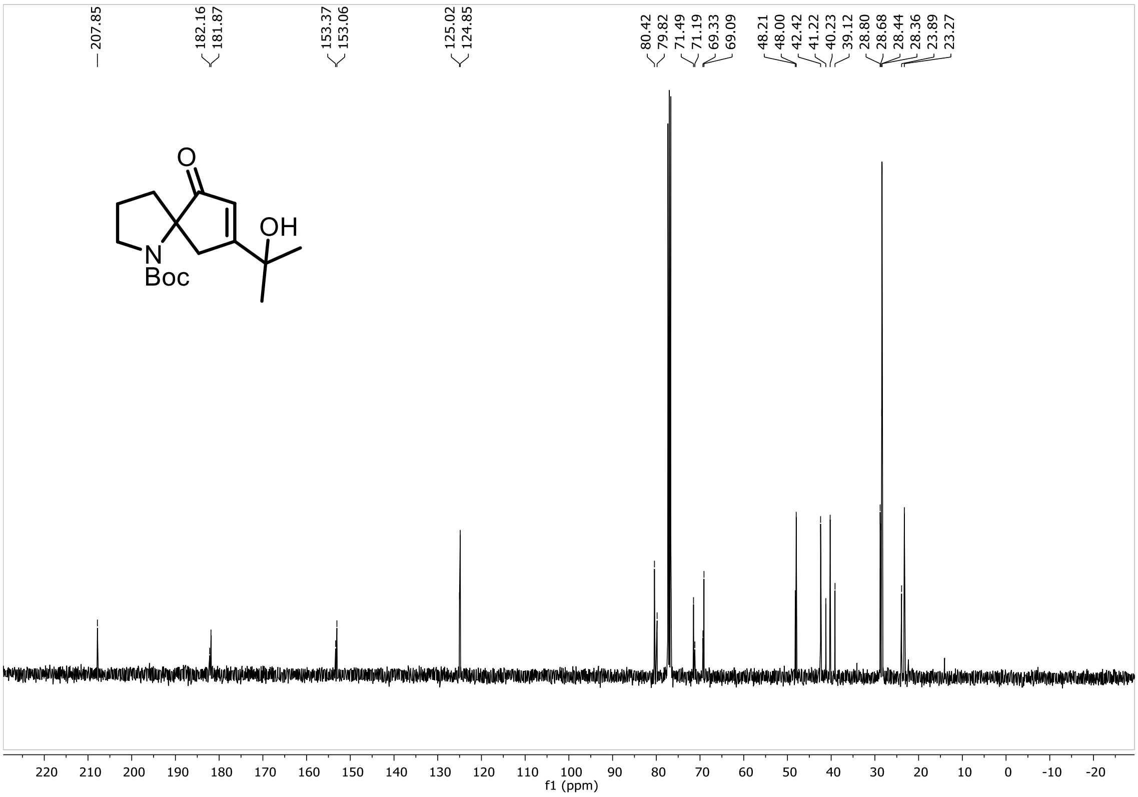

1H NMR Spectra for Compound 4i

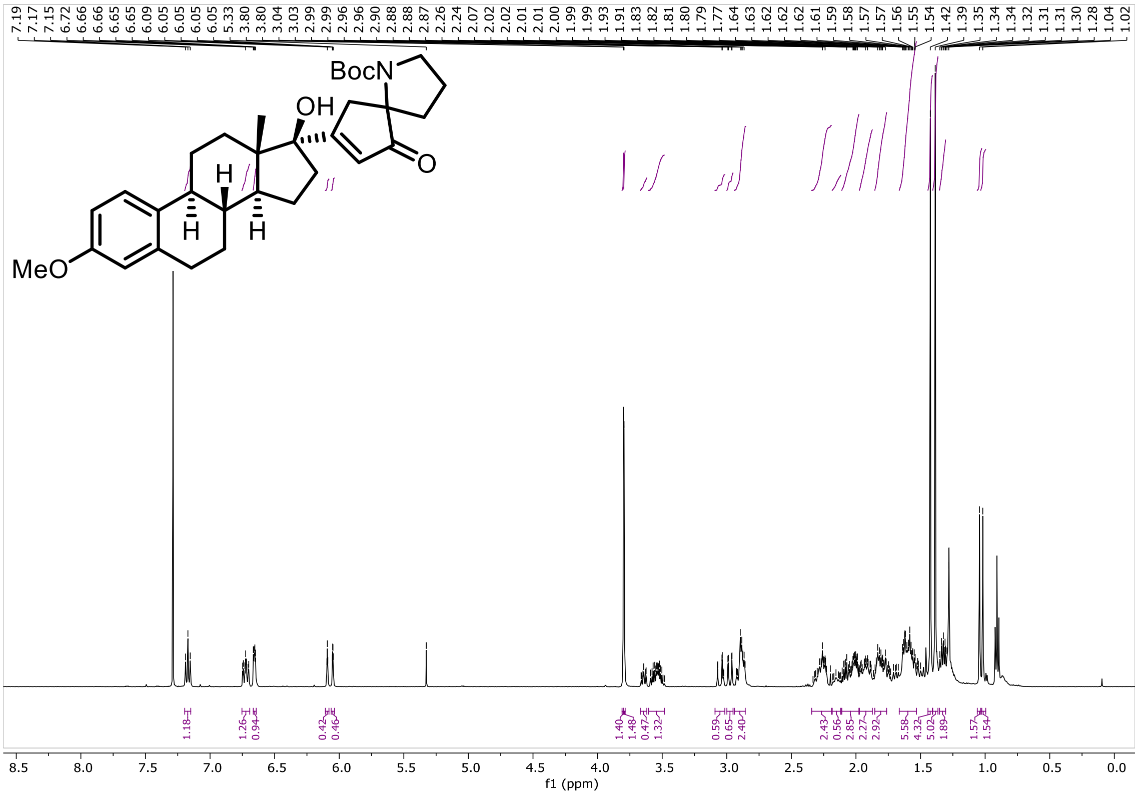

^{13}C NMR Spectra for Compound 4i

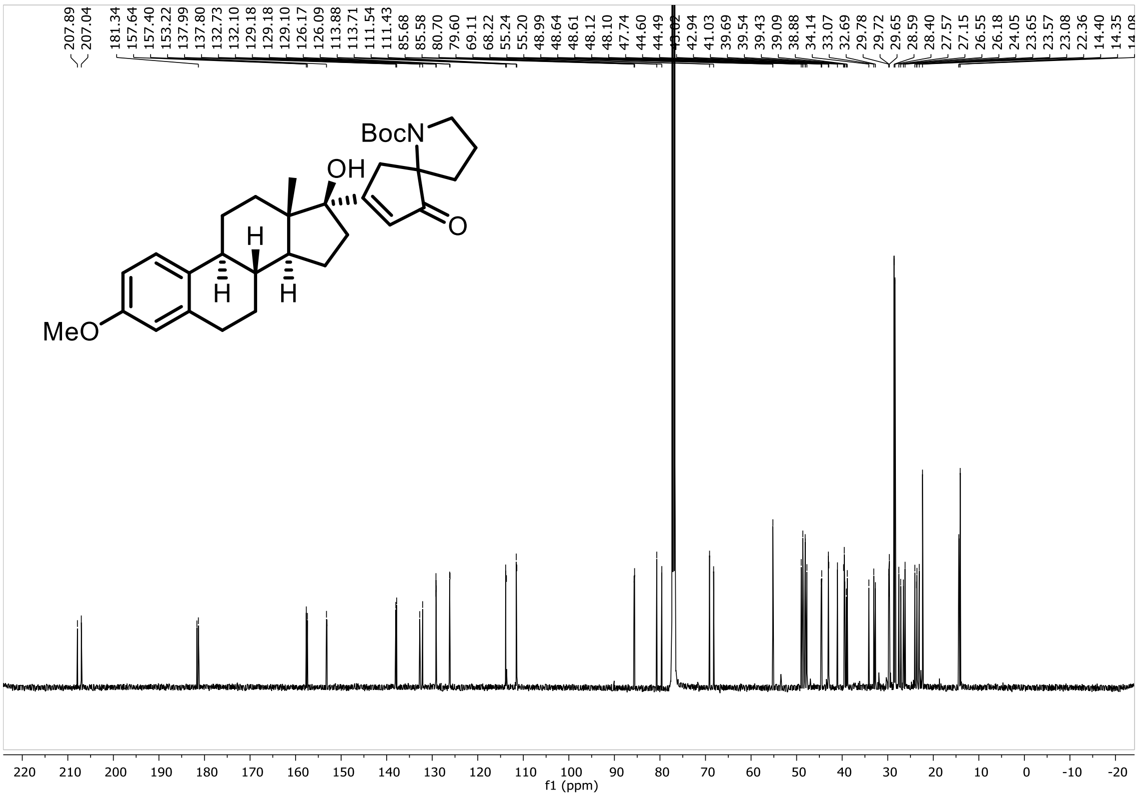

^{1}H NMR Spectra for Compound 4j

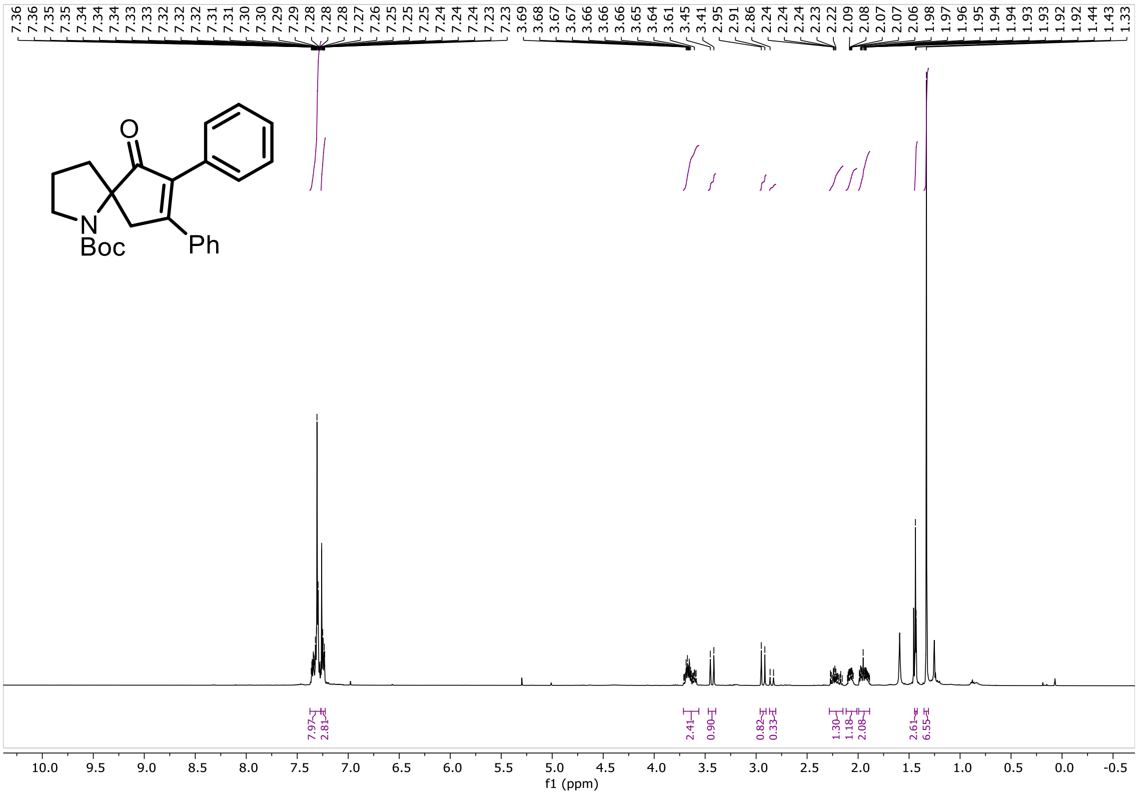

^{13}C NMR Spectra for Compound 4j

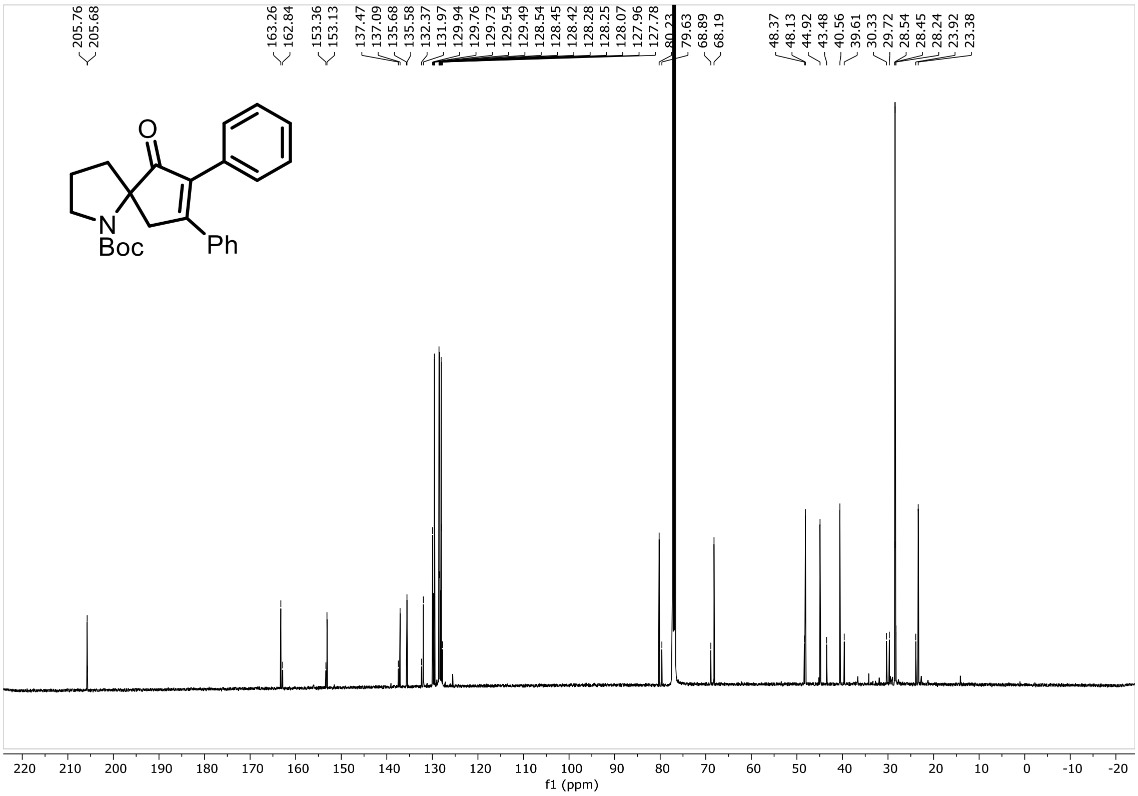

^{1}H NMR Spectra for Compound 4k

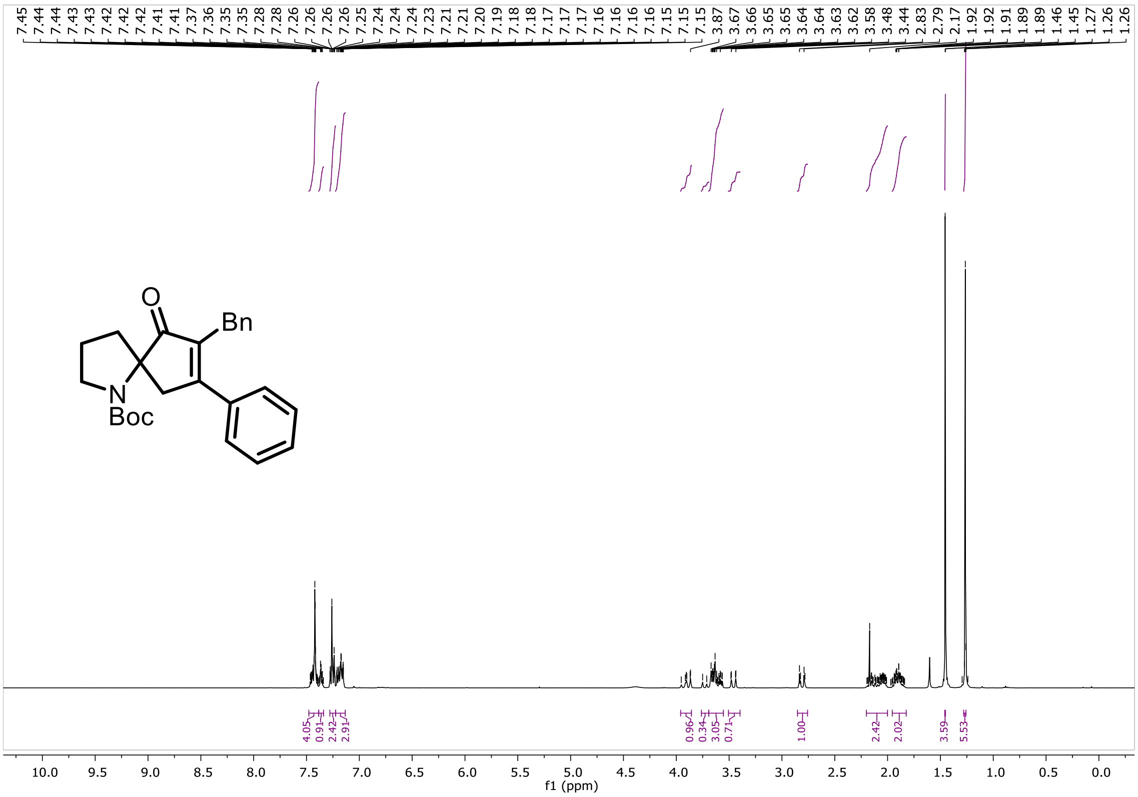

^{13}C NMR Spectra for Compound 4k

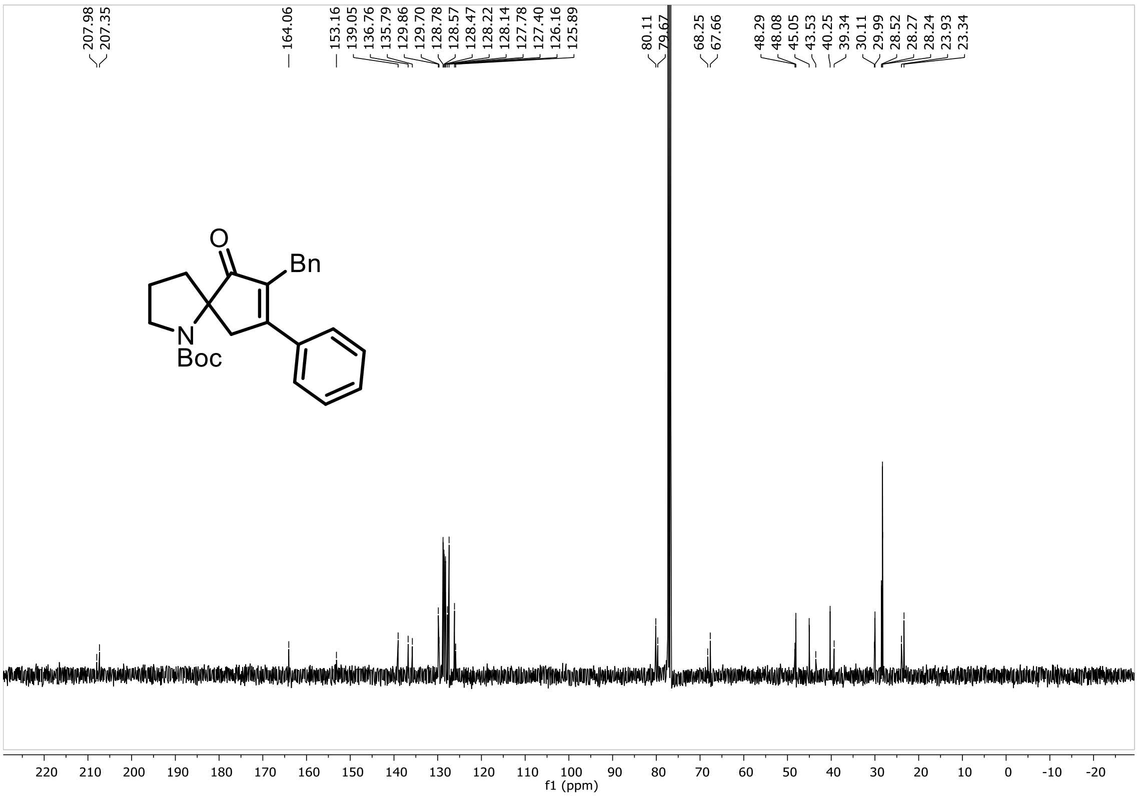

^{1}H NMR Spectra for Compound 4l

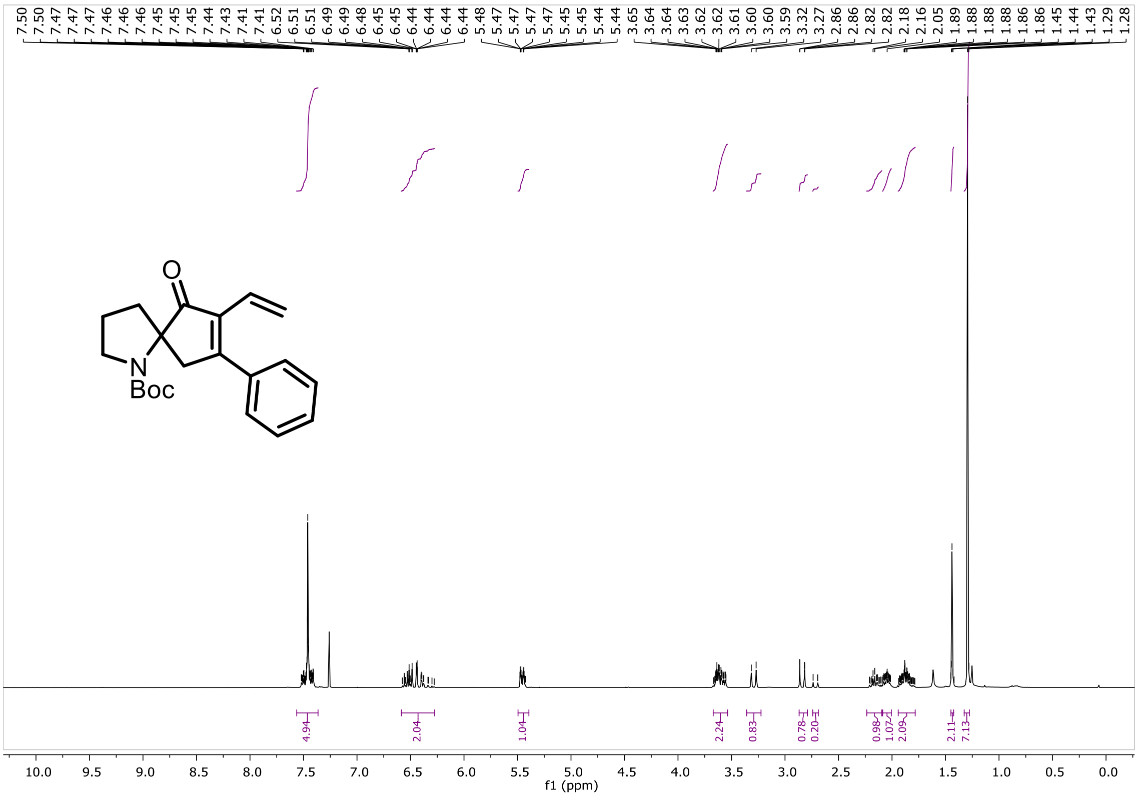

^{13}C NMR Spectra for Compound 4l

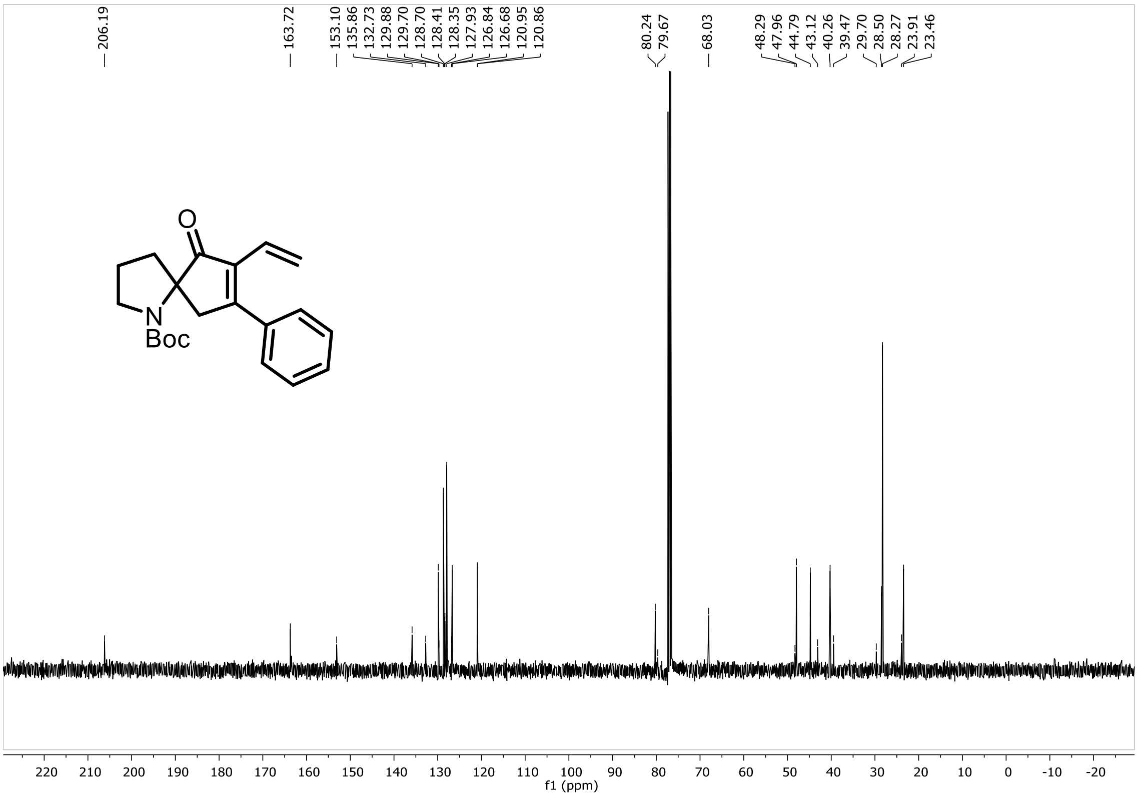

^{1}H NMR Spectra for Compound 4m

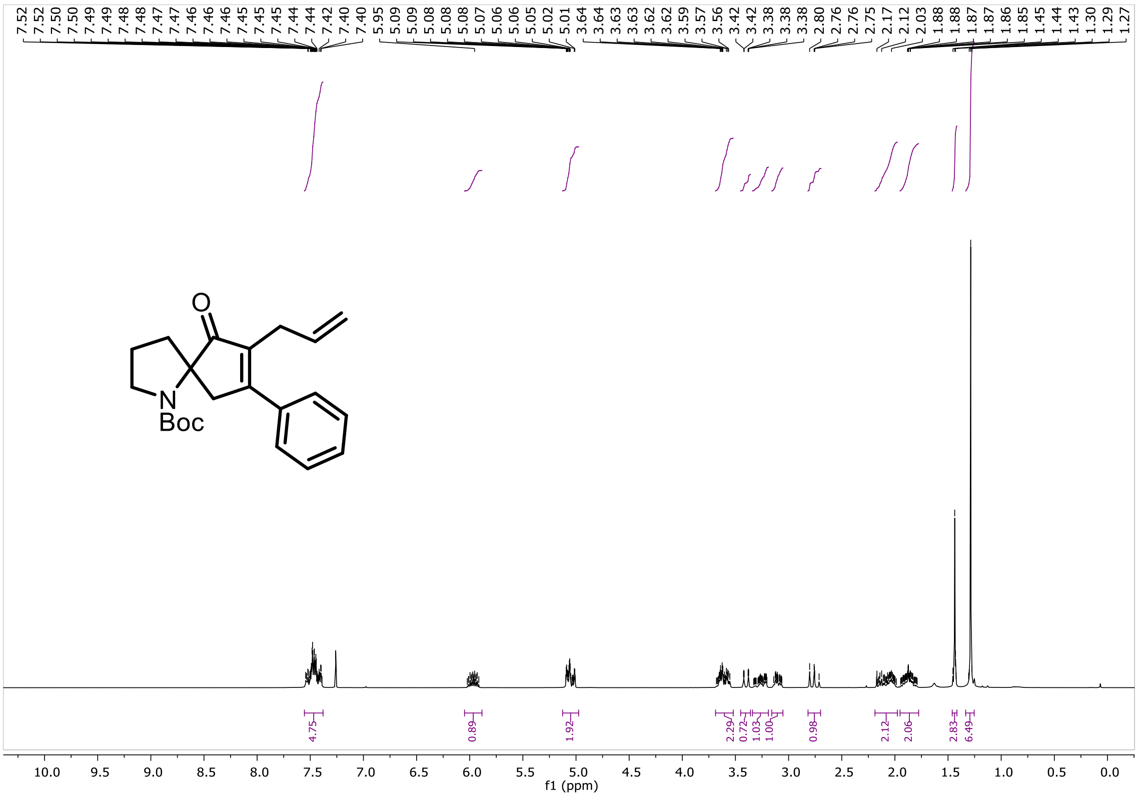

^{13}C NMR Spectra for Compound 4m

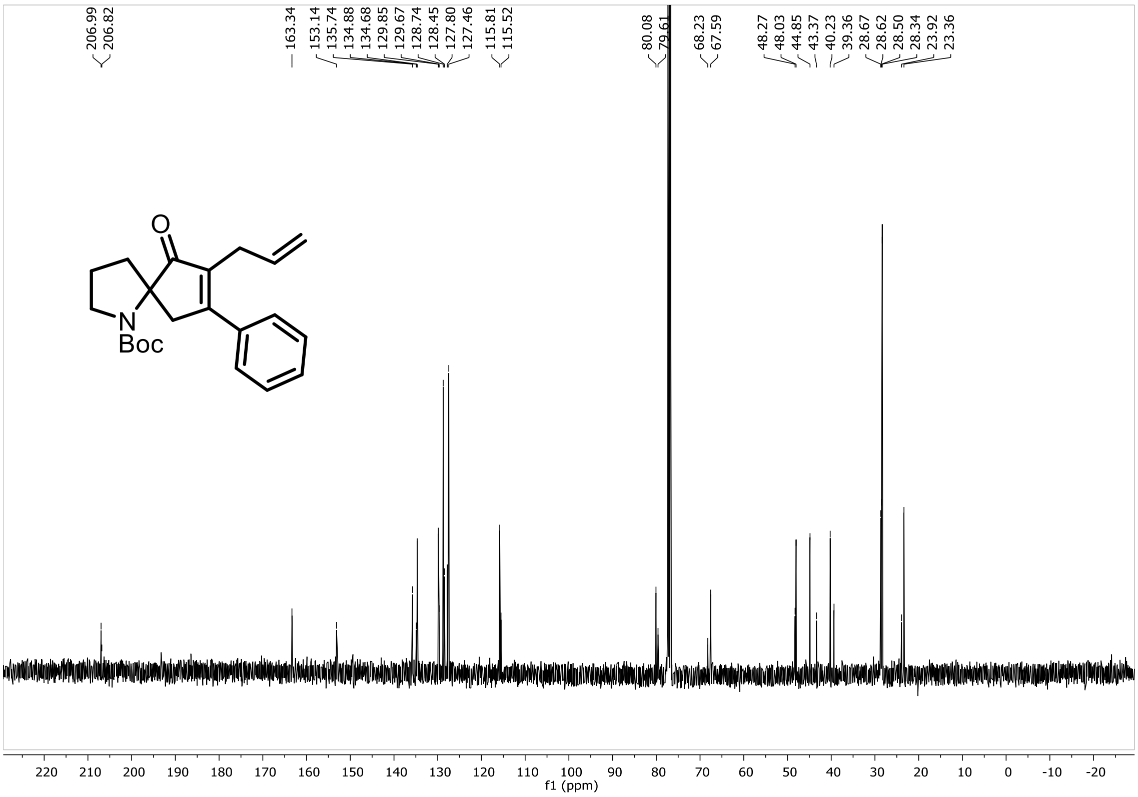

^{1}H NMR Spectra for Compound 4n

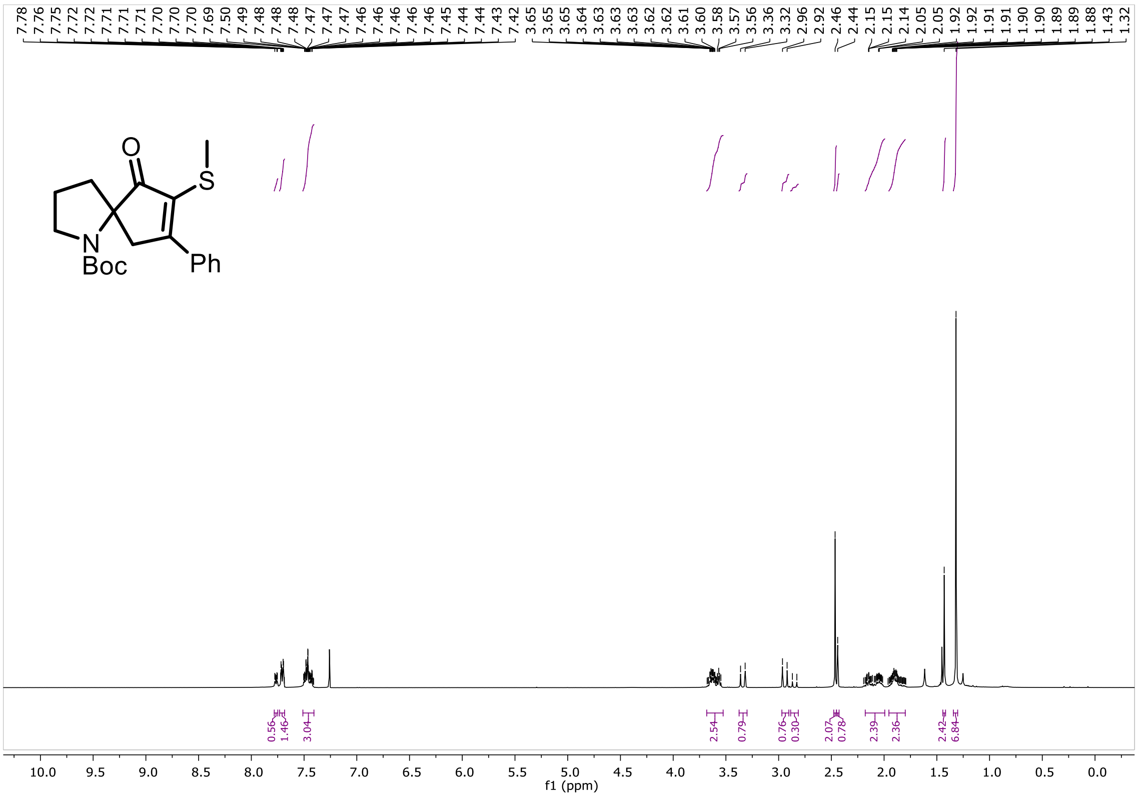

^{13}C NMR Spectra for Compound 4n

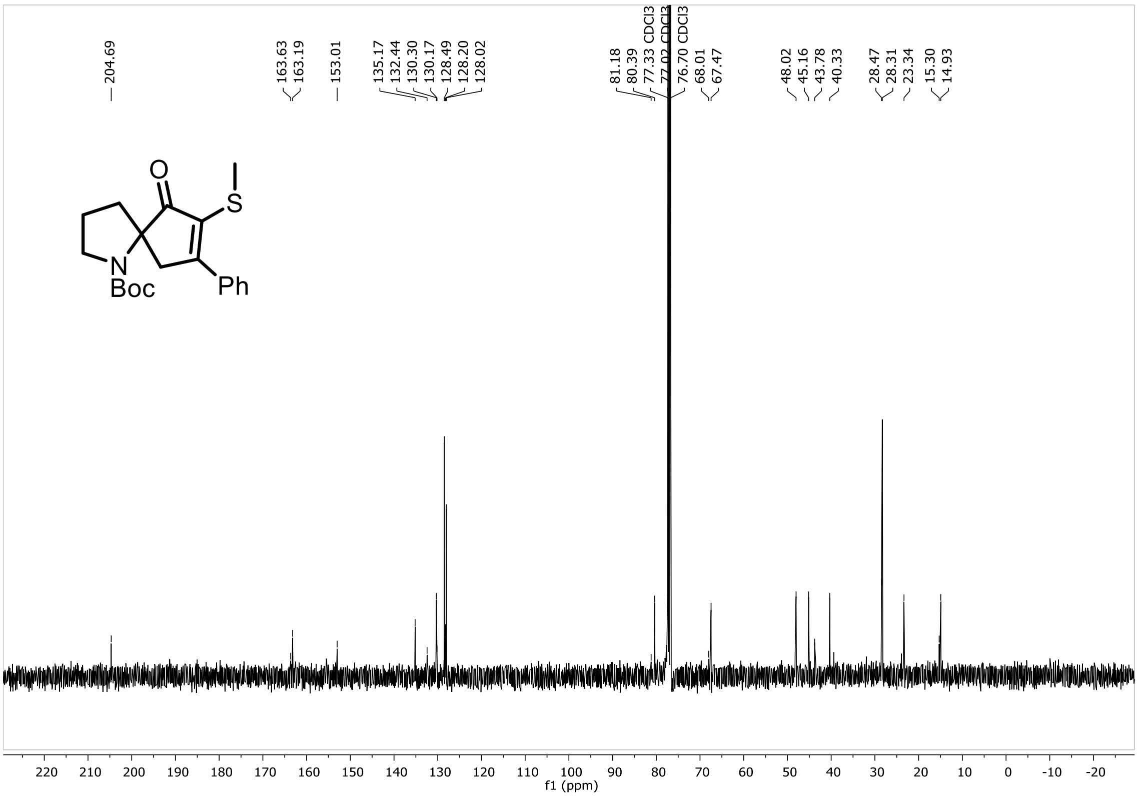

^{1}H NMR Spectra for Compound 4o

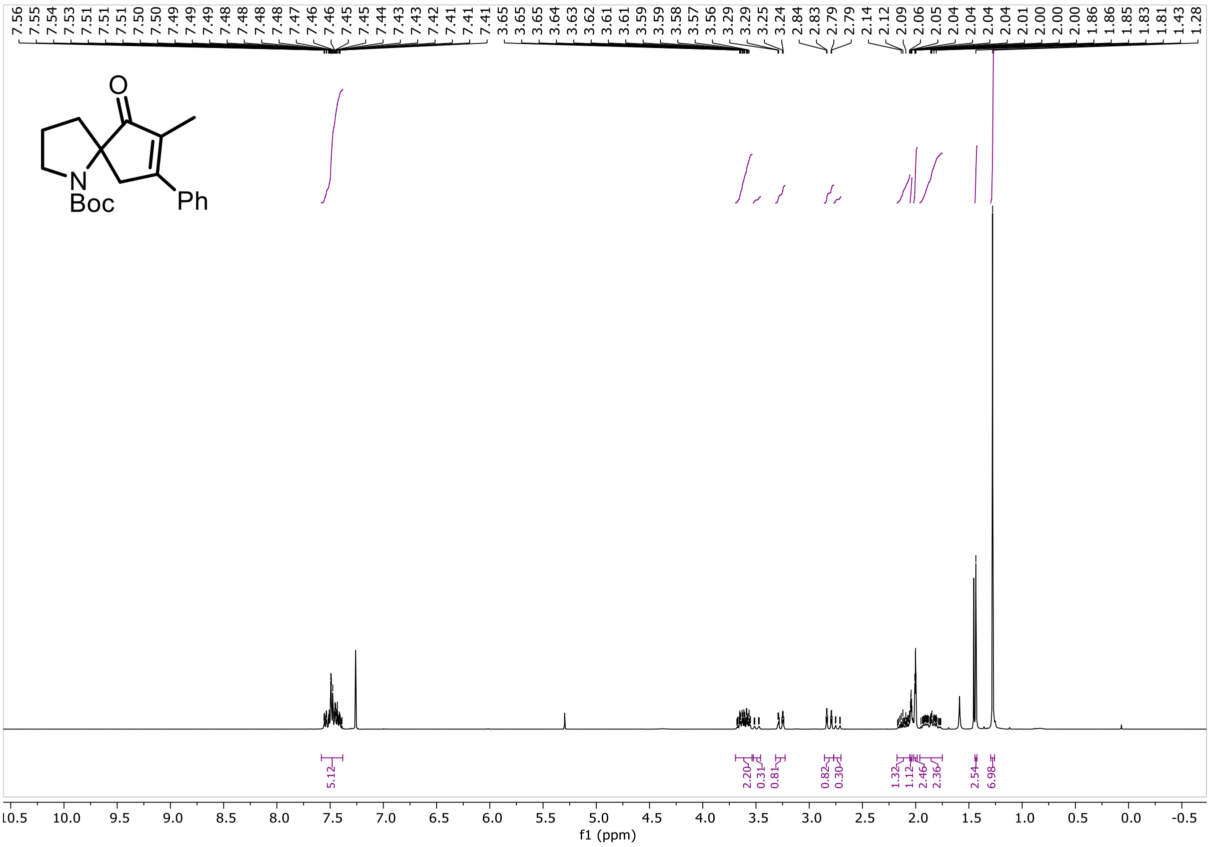

^{13}C NMR Spectra for Compound 4o

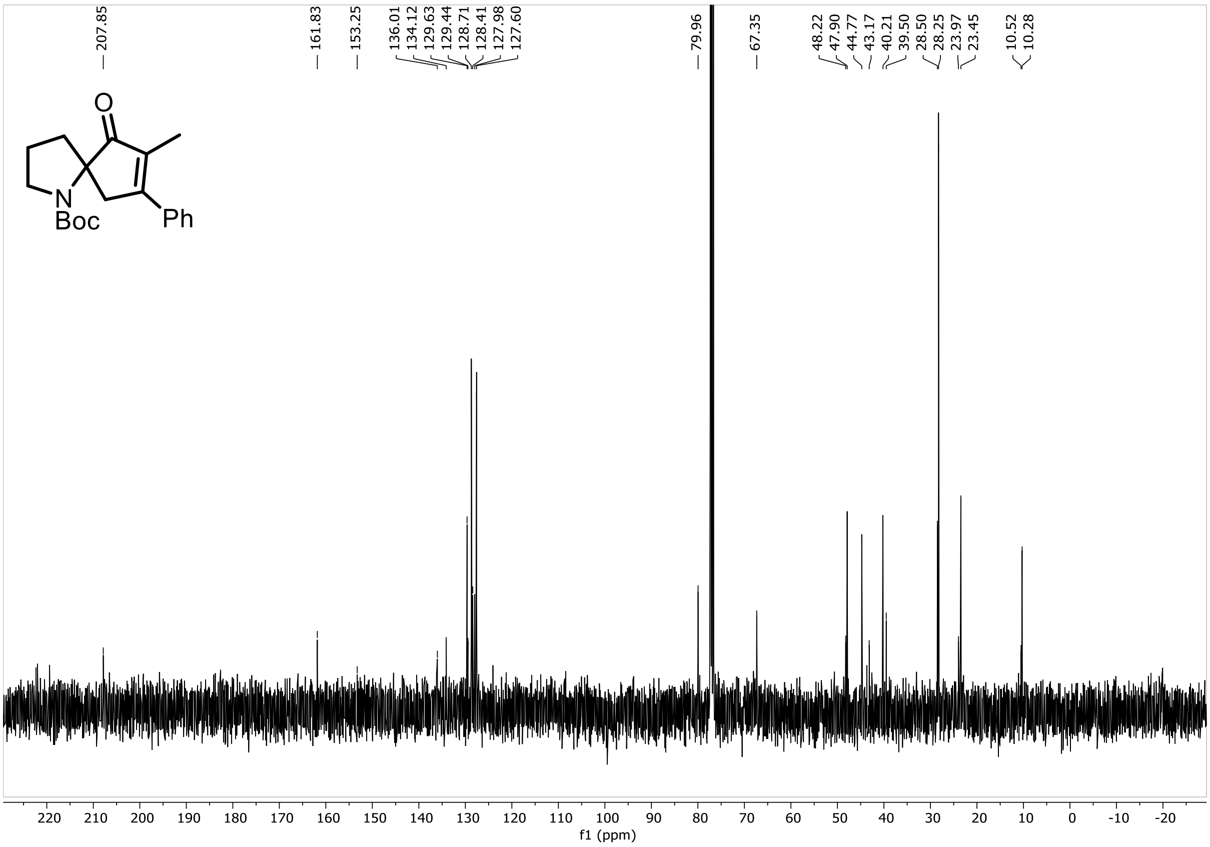

^{1}H NMR Spectra for Compound 4p

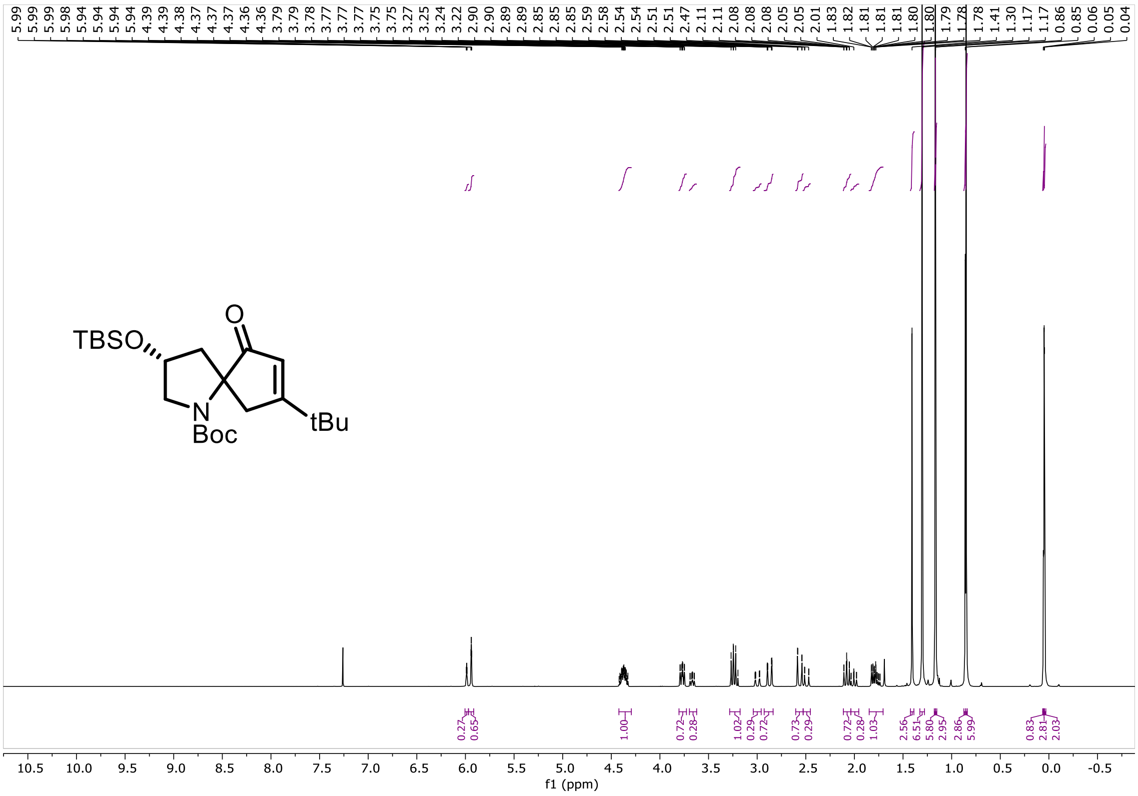

^{13}C NMR Spectra for Compound 4p

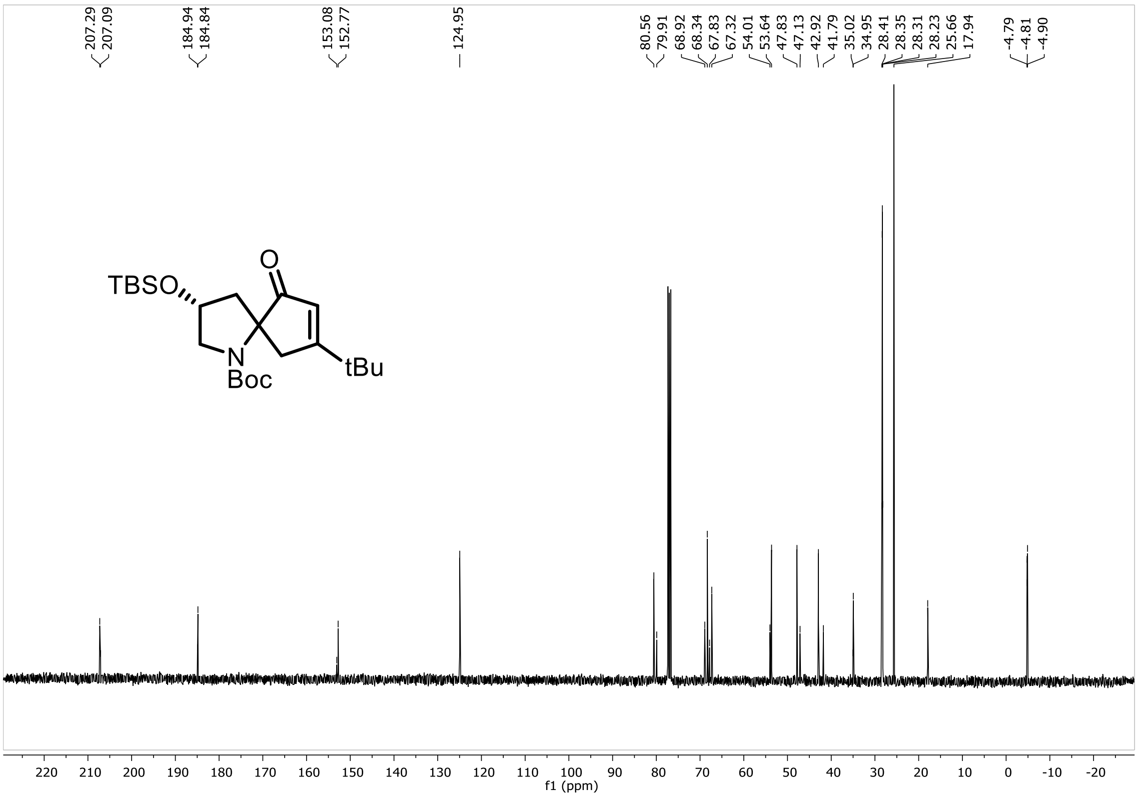

^{1}H NMR Spectra for Compound 4q

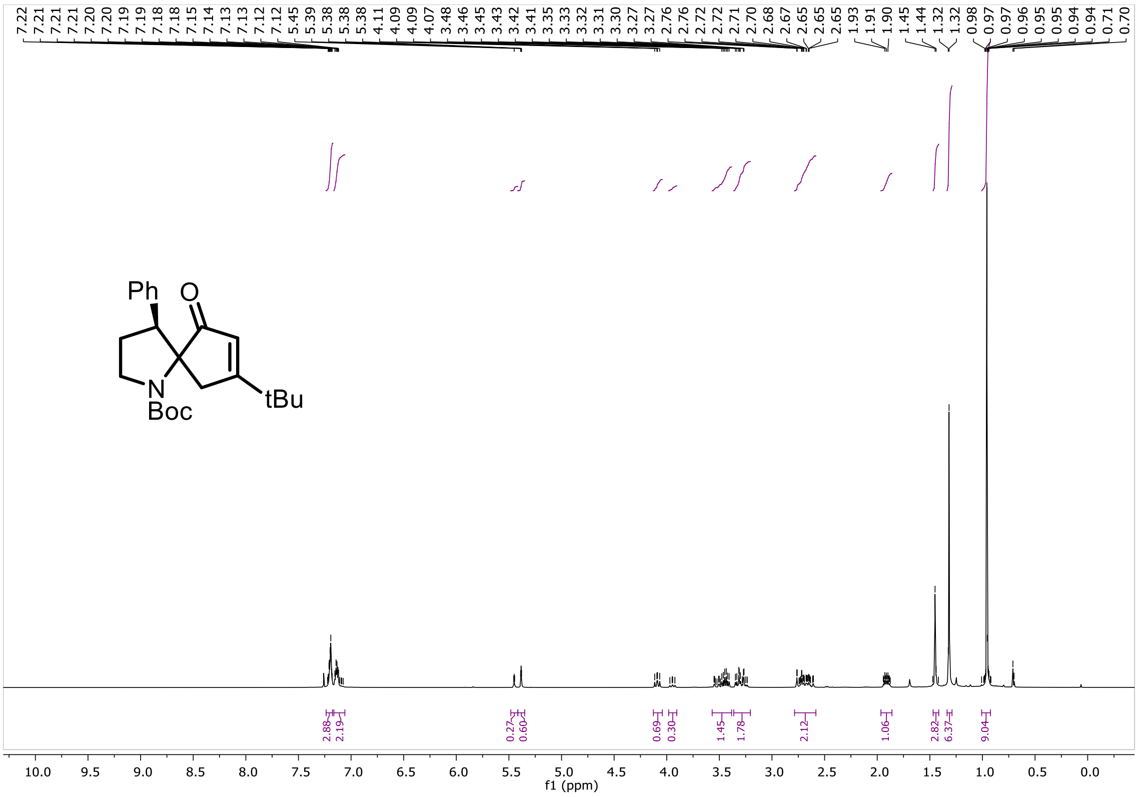

^{13}C NMR Spectra for Compound 4q

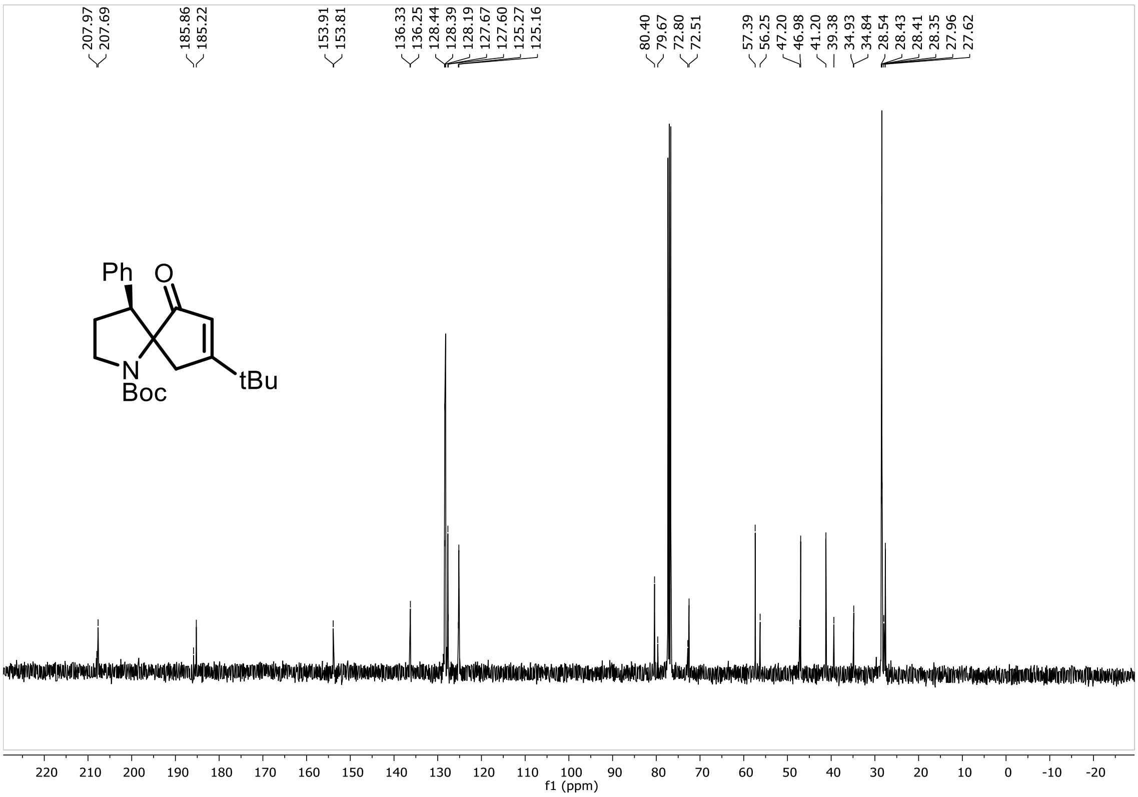

^{1}H NMR Spectra for Compound 4r

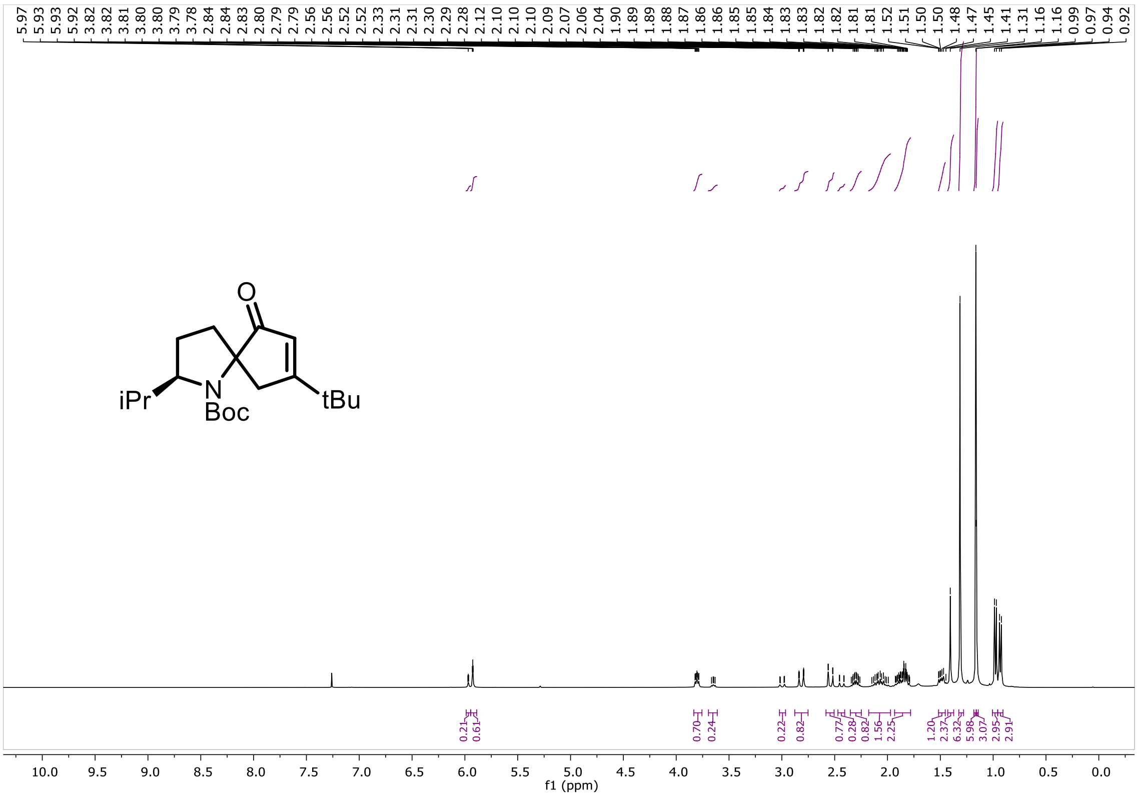

^{13}C NMR Spectra for Compound 4r

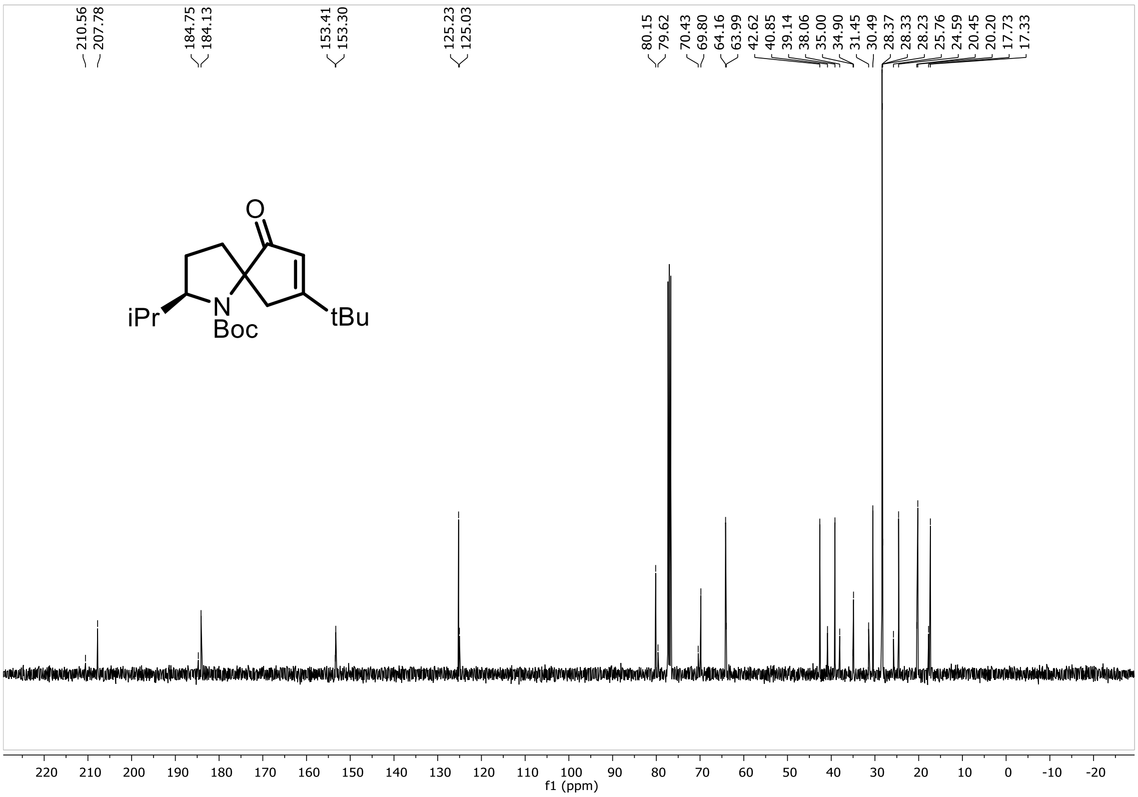

^{1}H NMR Spectra for Compound 4a'

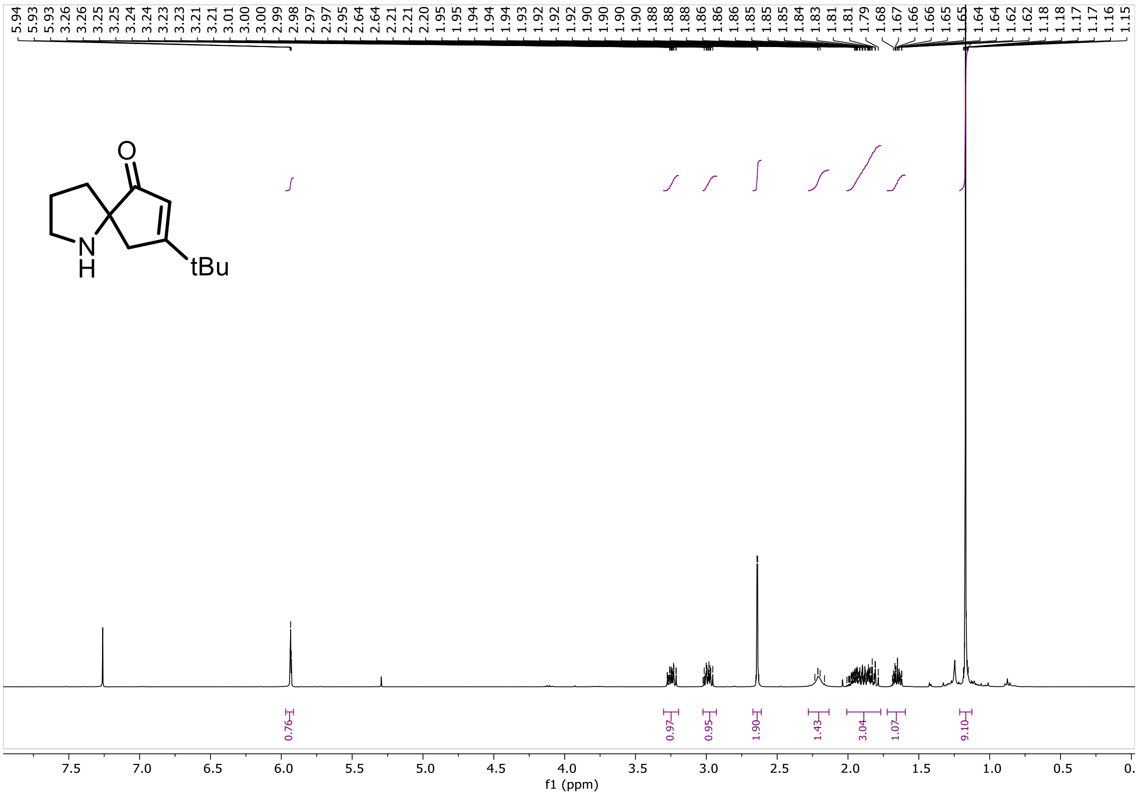

^{13}C NMR Spectra for Compound 4a'

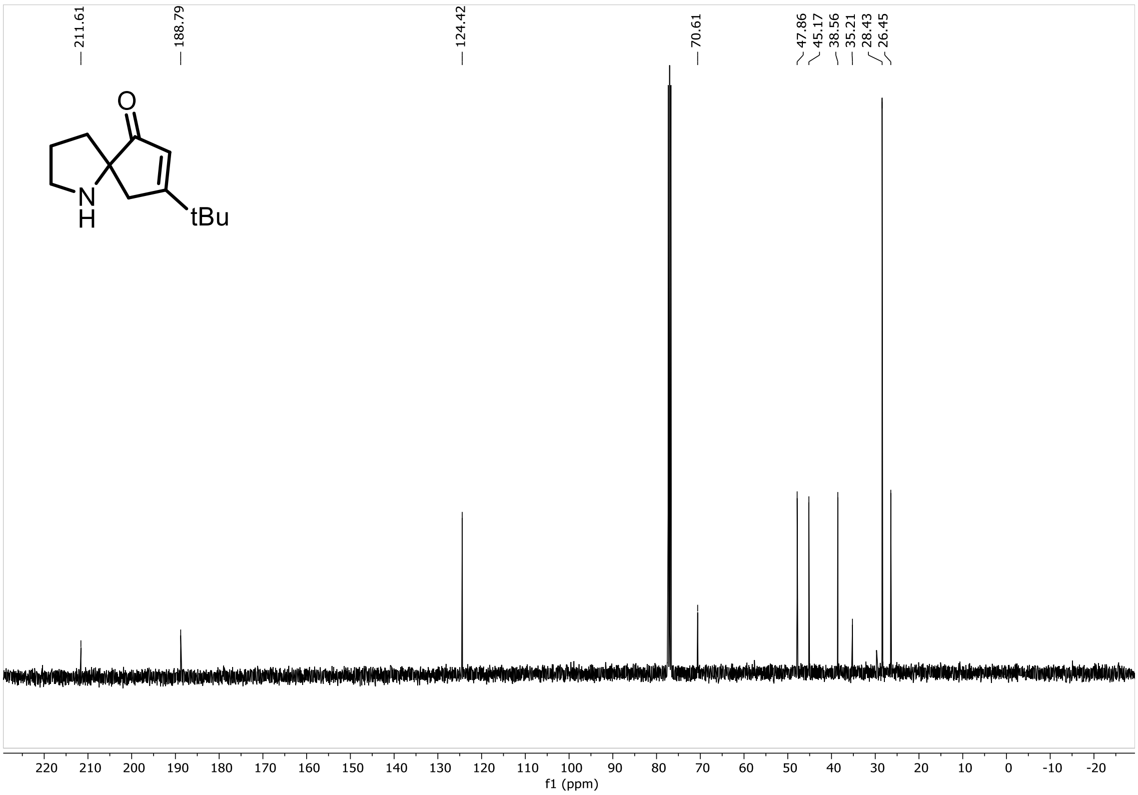

^{1}H NMR Spectra for Compound 2s

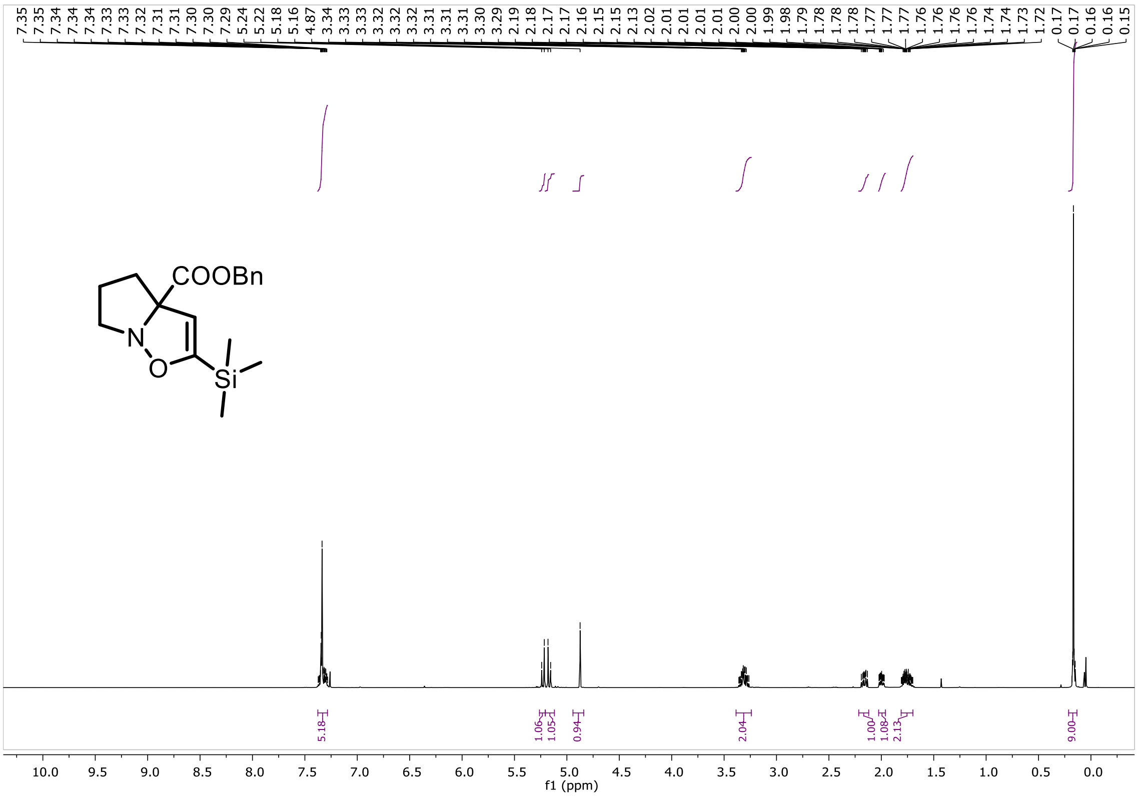

^{13}C NMR Spectra for Compound 2s

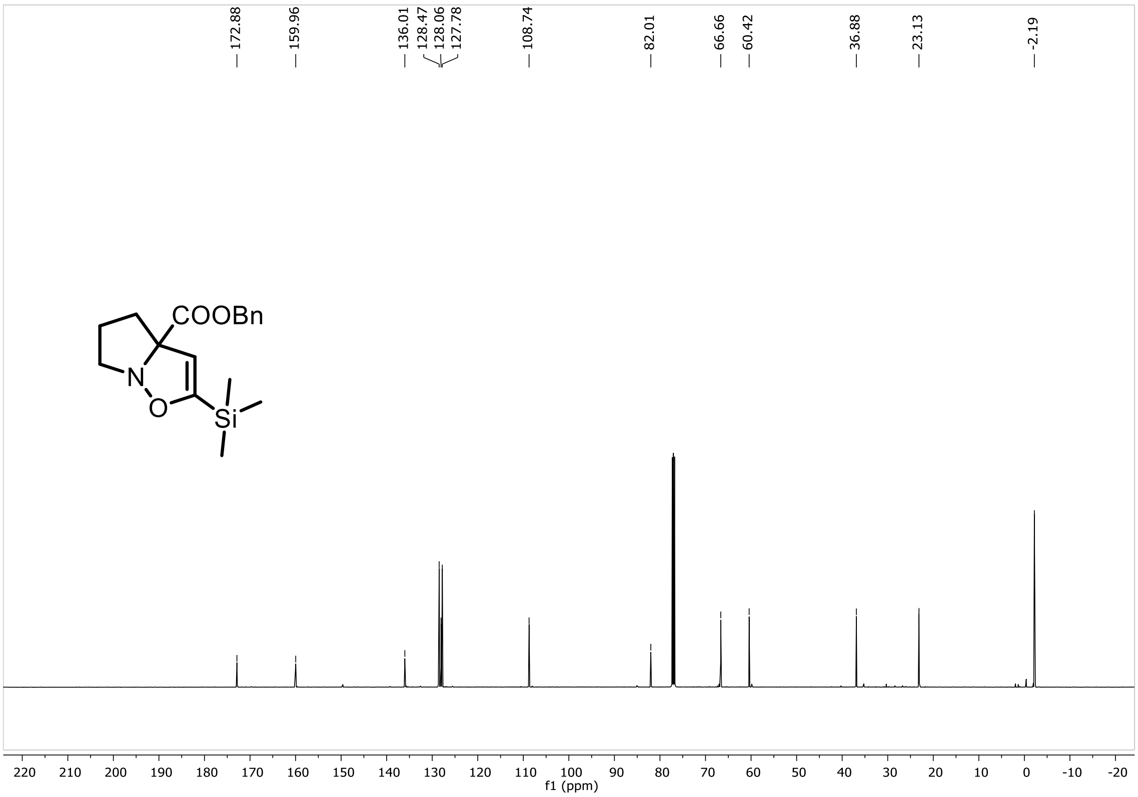

^{1}H NMR Spectra for Compound 3s

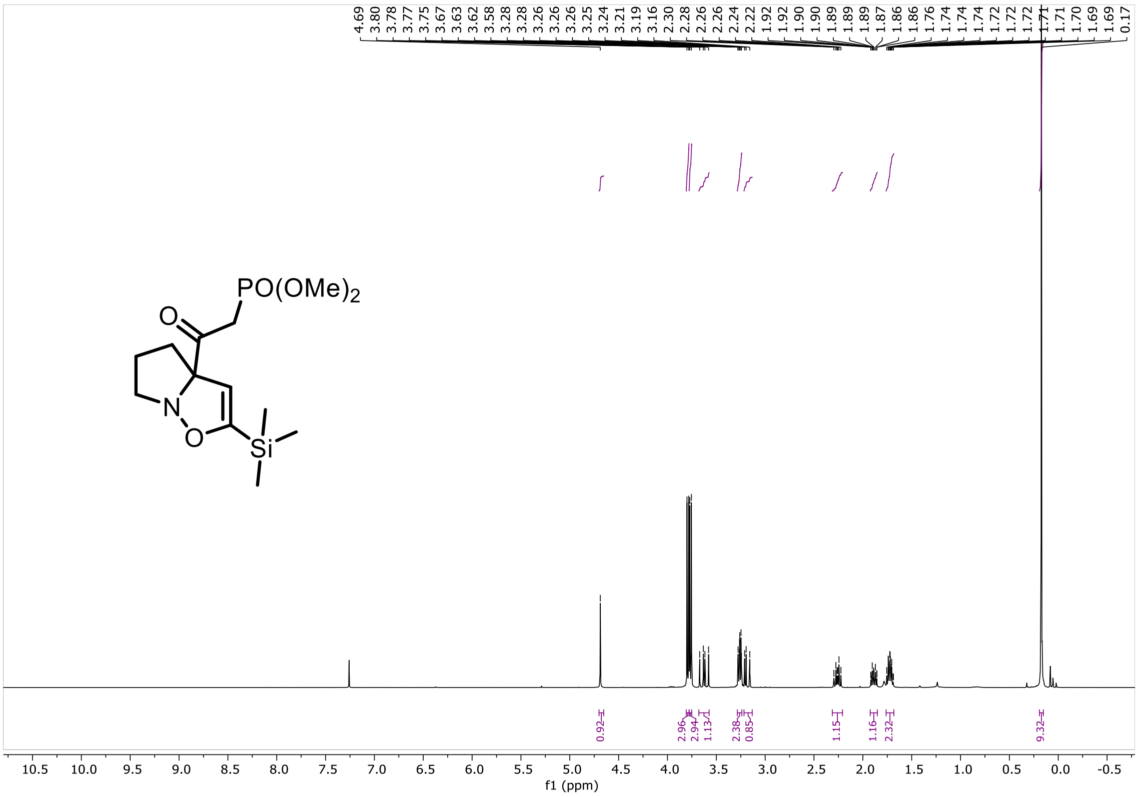

^{13}C NMR Spectra for Compound 3s

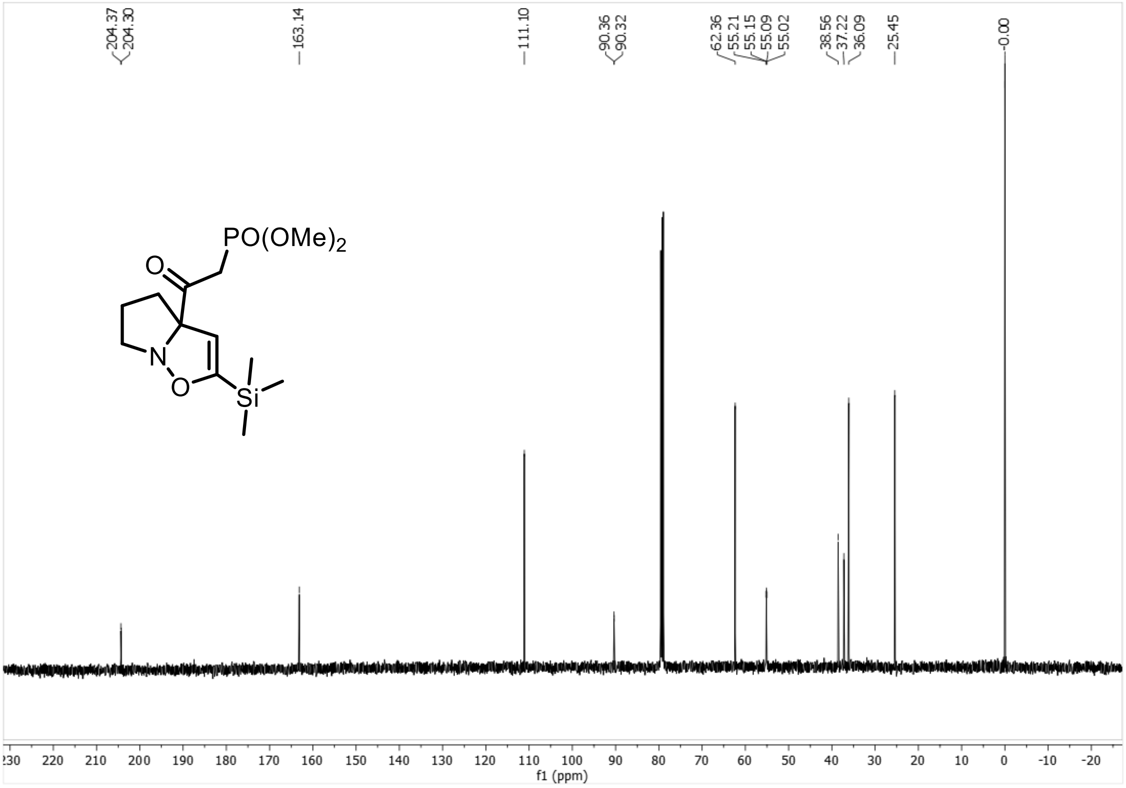

^{31}P NMR Spectra for Compound 3s

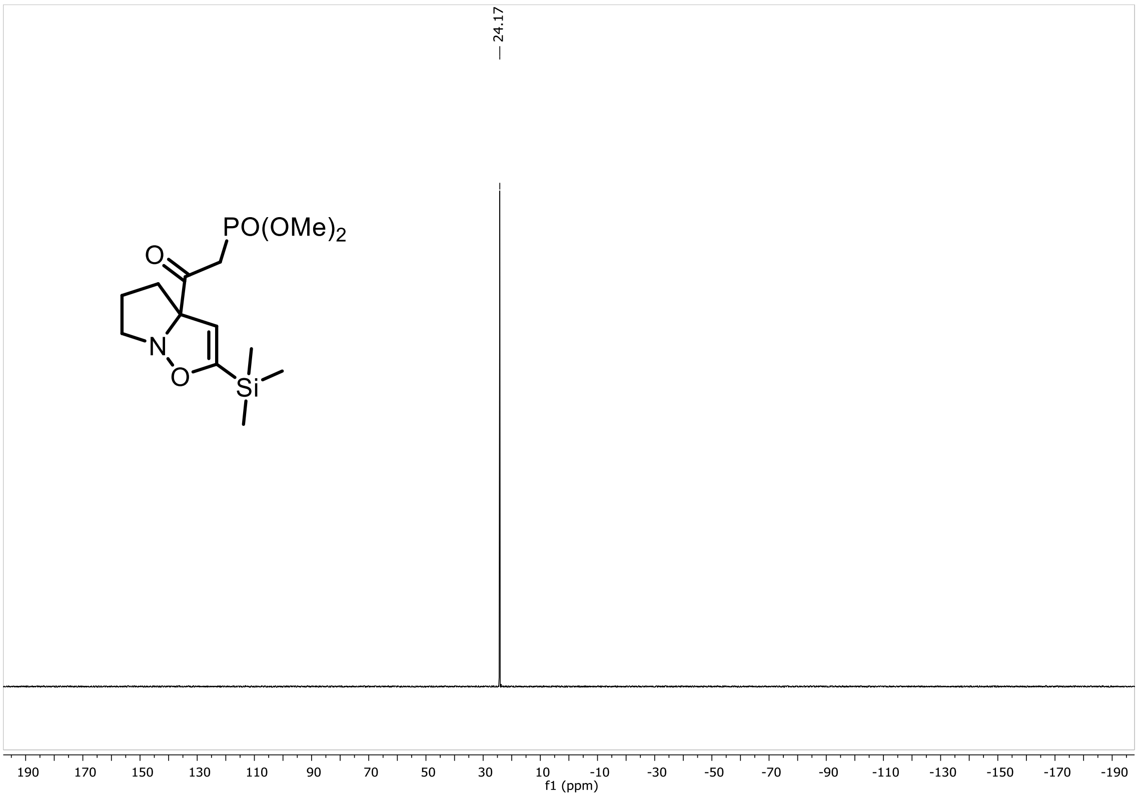

^{1}H NMR Spectra for Compound 4s

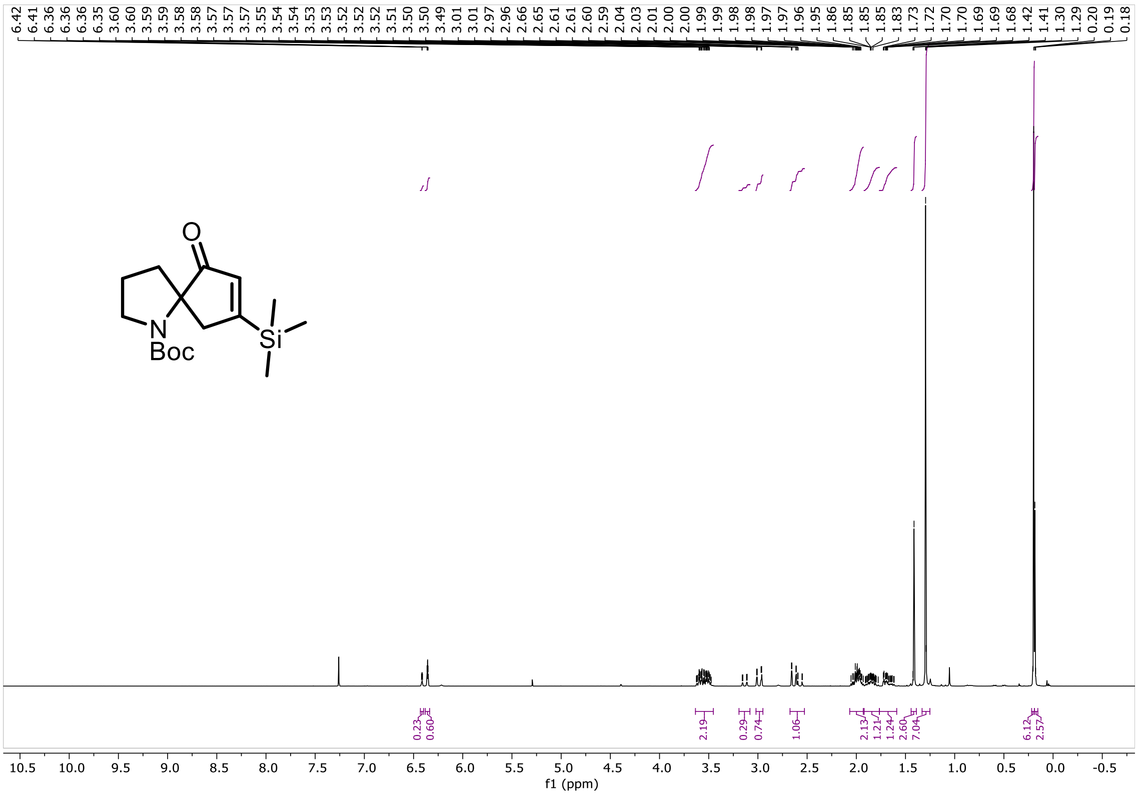

^{13}C NMR Spectra for Compound 4s

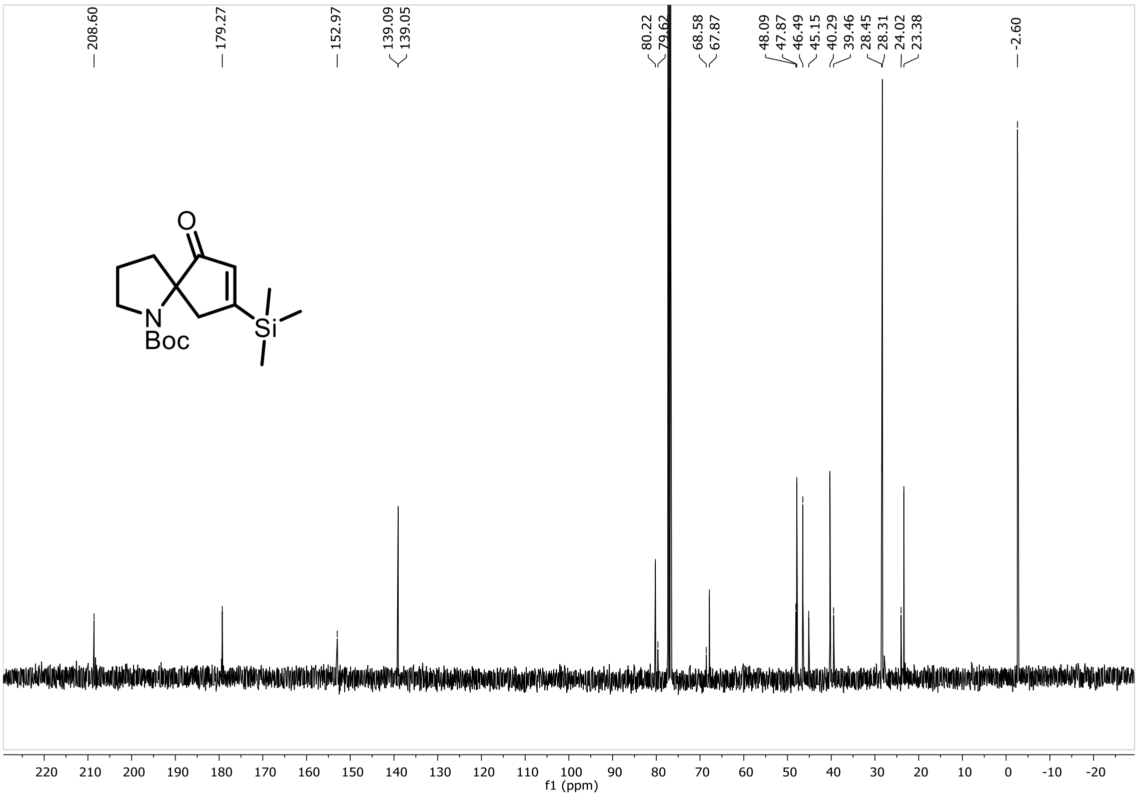

^{1}H NMR Spectra for Compound 5

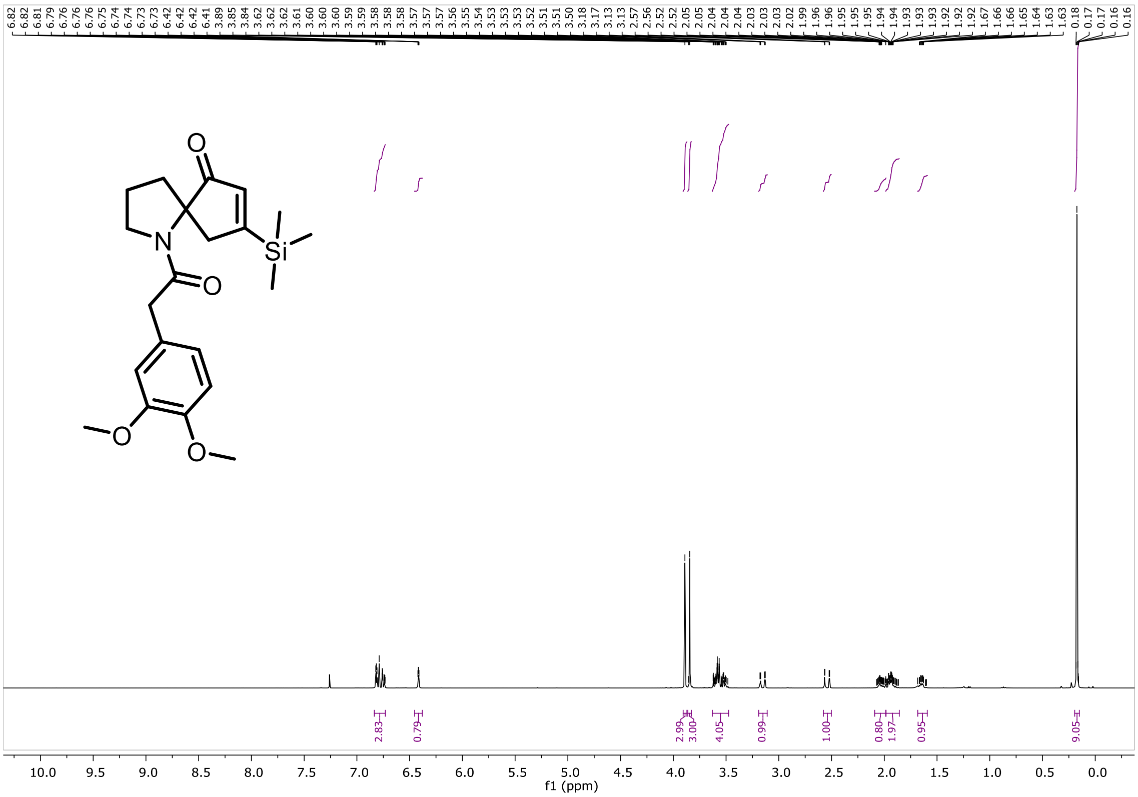

^{13}C NMR Spectra for Compound 5

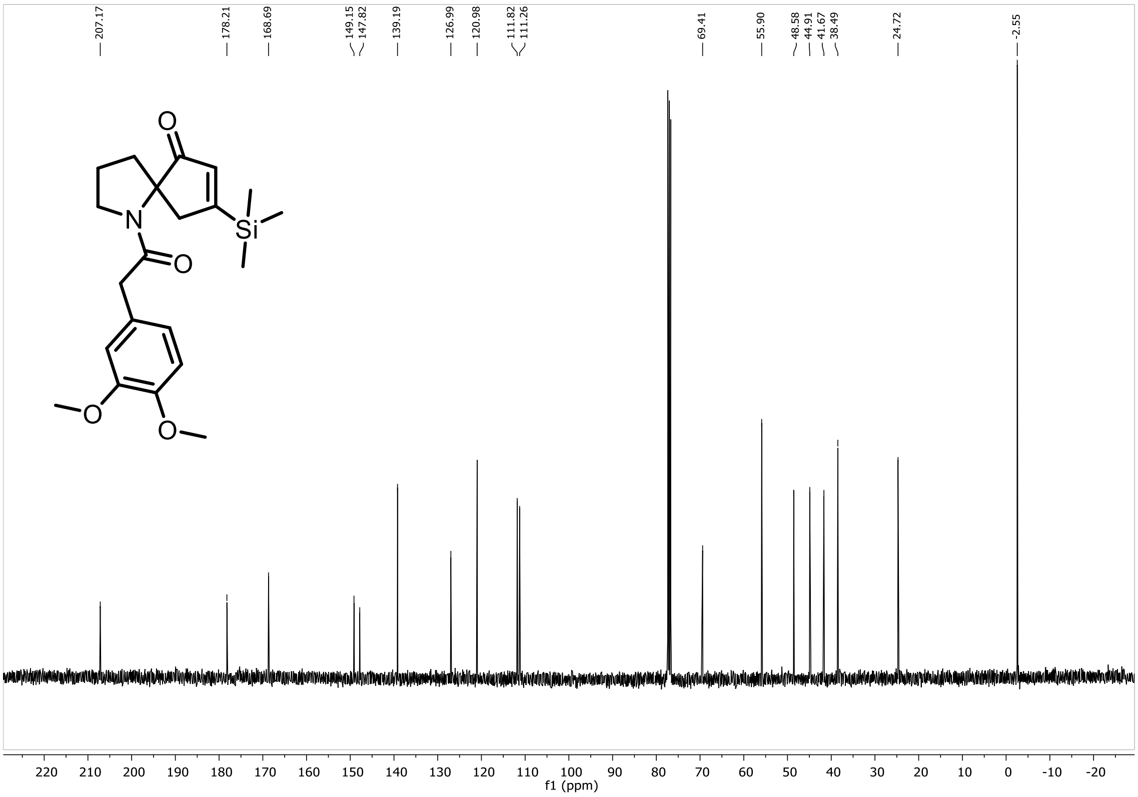

^{1}H NMR Spectra for Compound S15

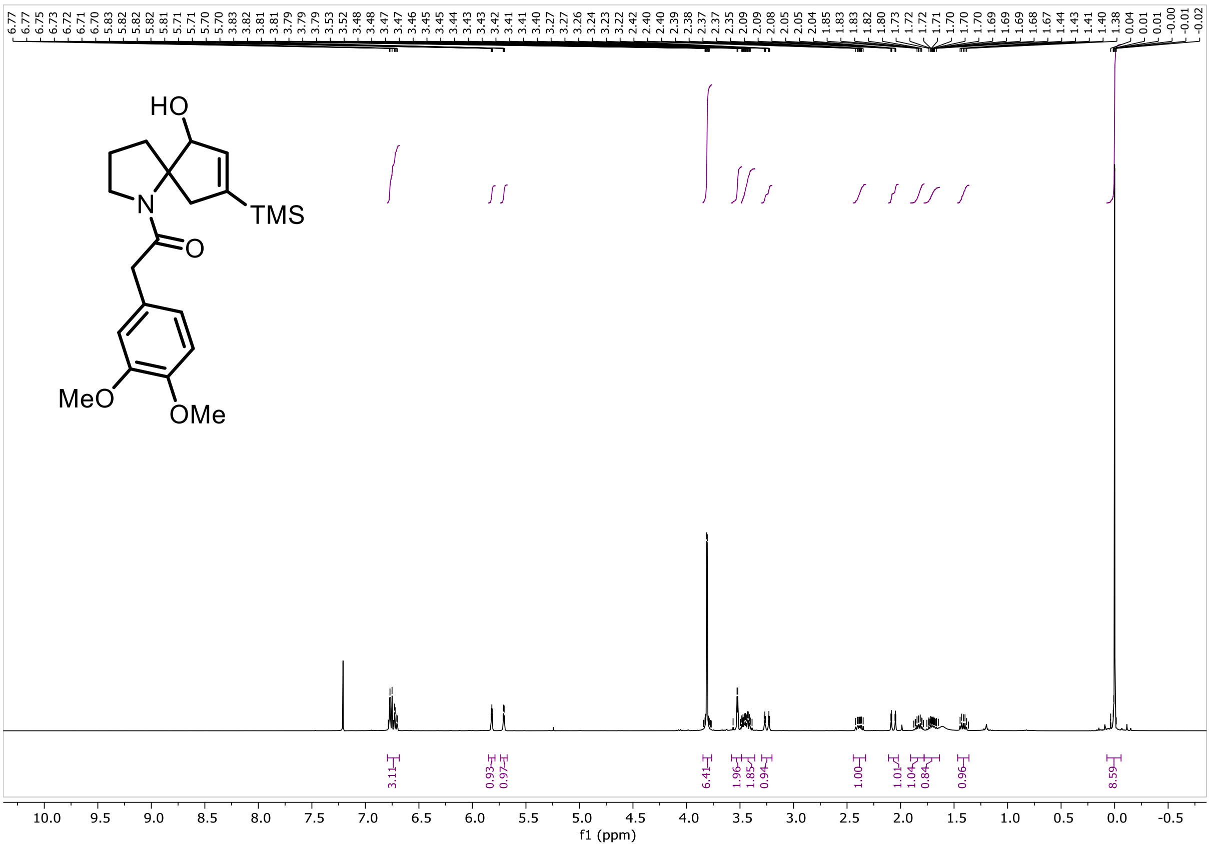

^{13}C NMR Spectra for Compound S15

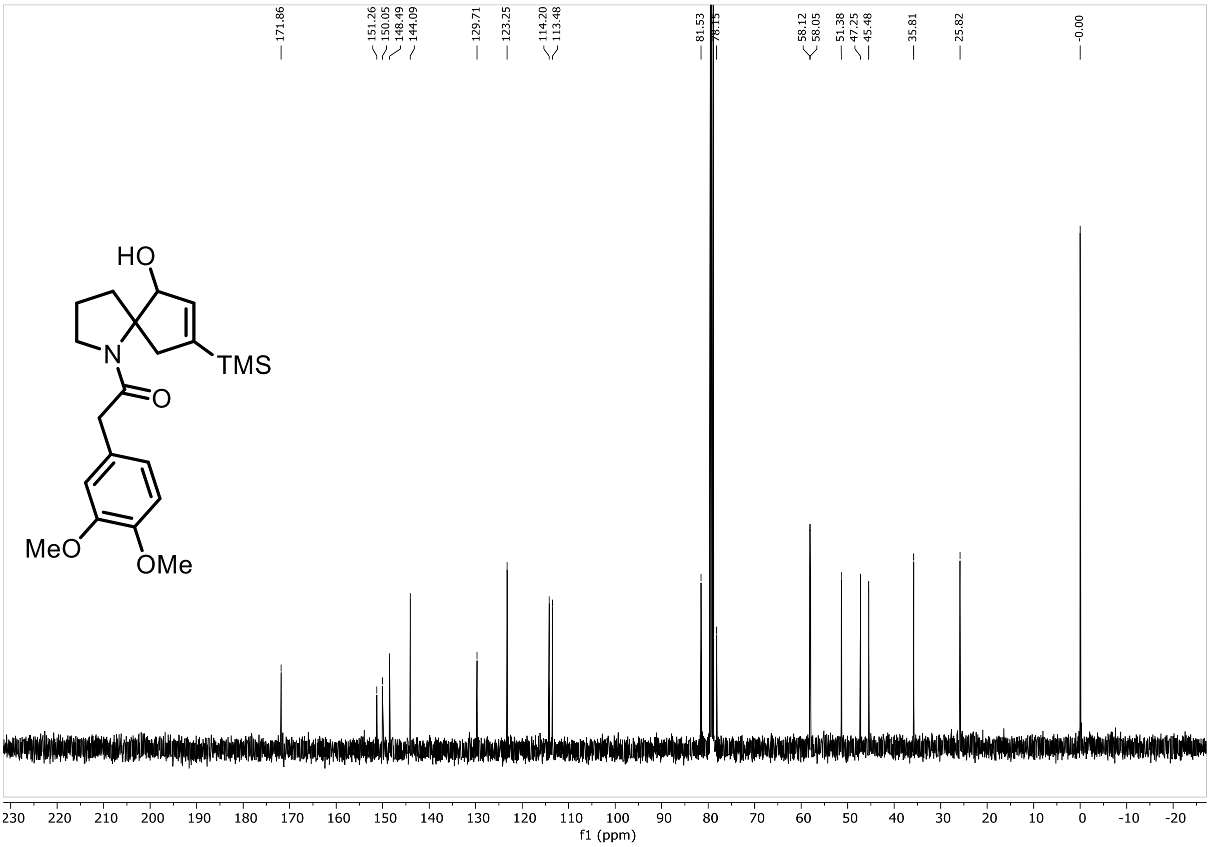

^{1}H NMR Spectra for Compound 6

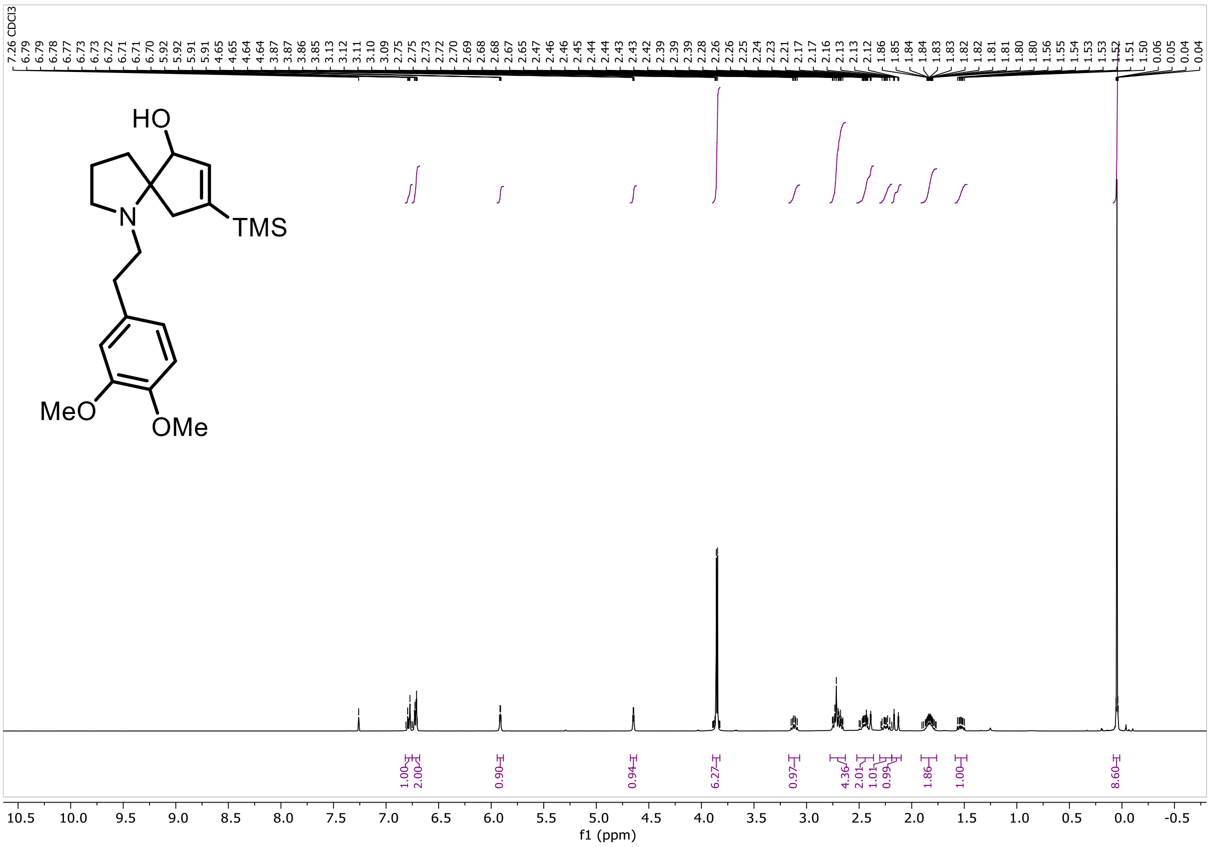

^{13}C NMR Spectra for Compound 6

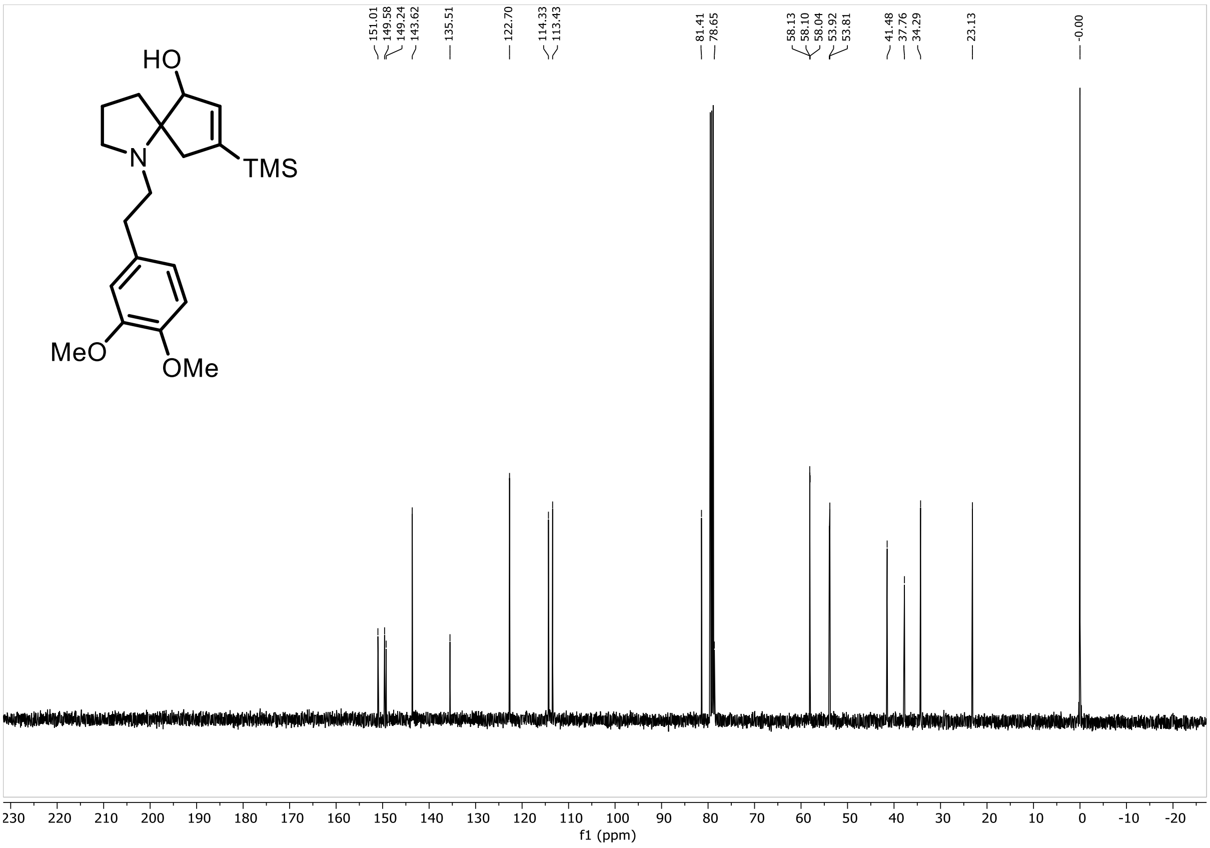

^{1}H NMR Spectra for Compound 7

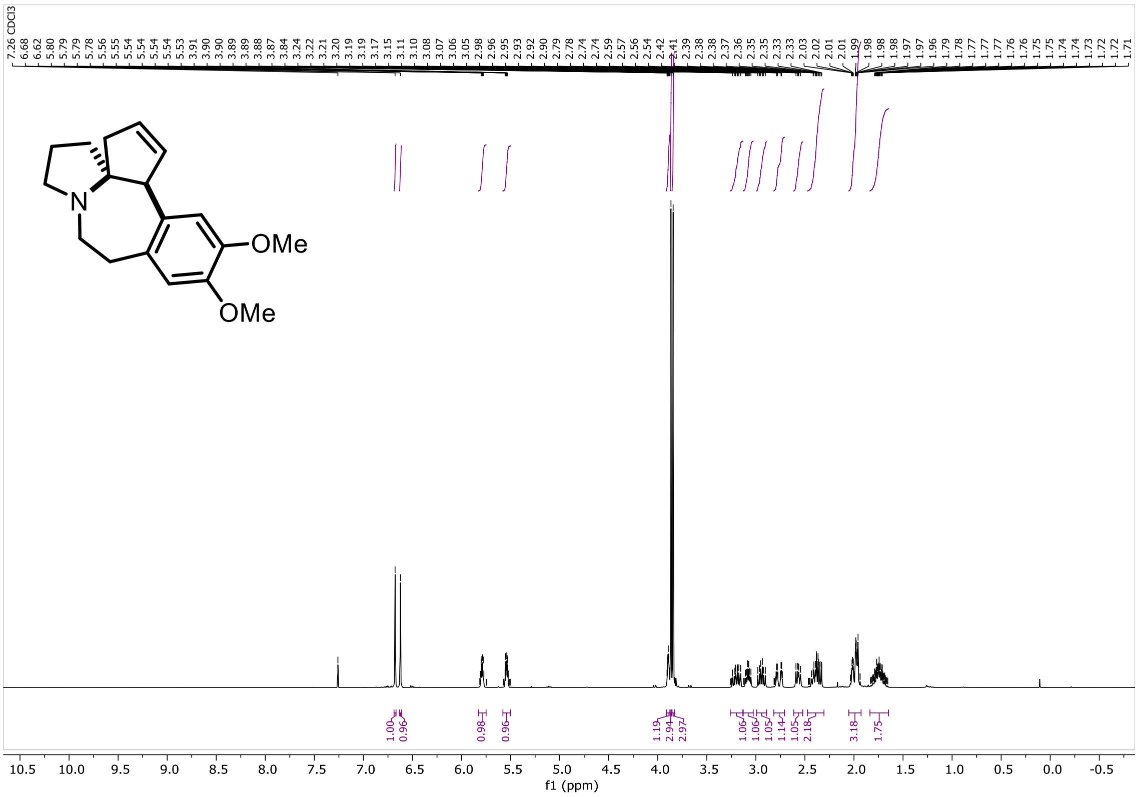

^{13}C NMR Spectra for Compound 7

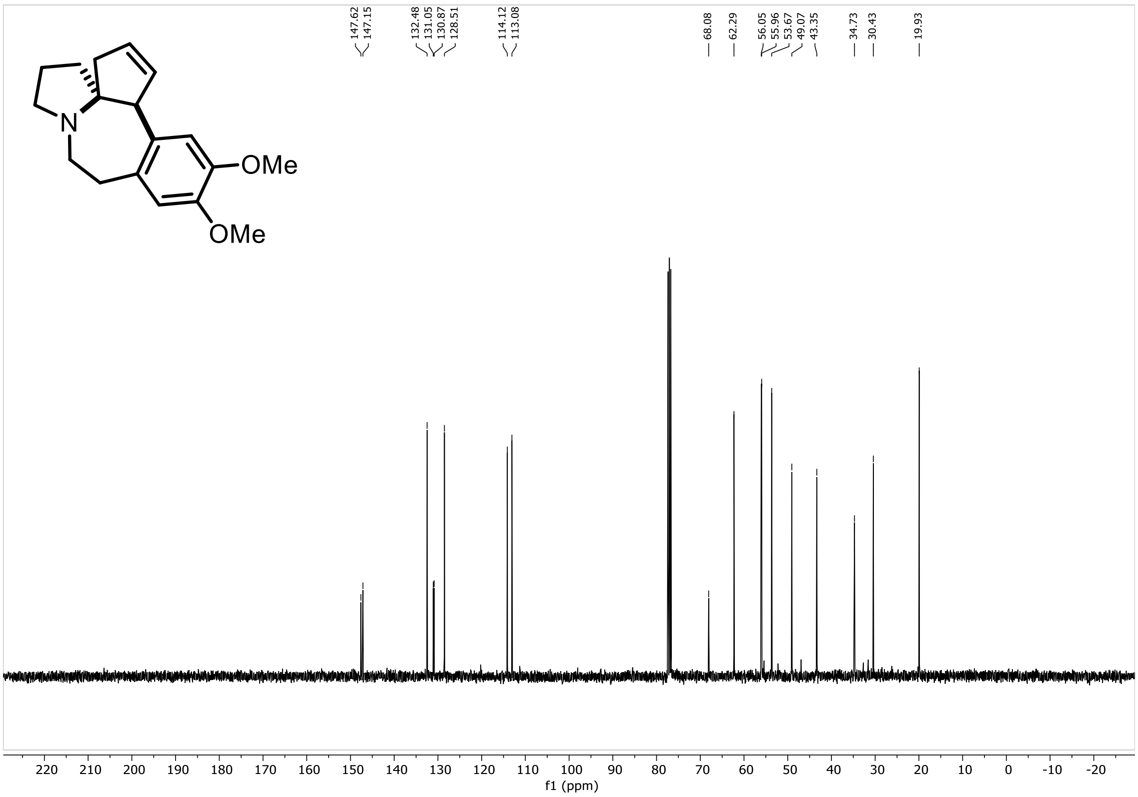

References

(1) S. I. Murahashi, H. Mitsui, T. Shiota, T. Tsuda and S. Watanabe, *J. Org. Chem.*, 1990, **55**, 1736–1744.

(2) W. W. Zajac, T. R. Walters and M. G. Darcy, *J. Org. Chem.*, 1988, **53**, 5856–5860.

(3) S. I. Murahashi and Y. Imada, *Chem. Rev.*, 2019, **119**, 4684–4716.

(4) K. M. Maloney and J. Y. L. Chung, *J. Org. Chem.*, 2009, **74**, 7574–7576.

(5) R. Madsen, C. Roberts and B. Fraser-Reid, *J. Org. Chem.*, 1995, **60**, 7920–7926.

(6) T. Imaoka, O. Iwamoto, K. I. Noguchi and K. Nagasawa, *Angew. Chemie - Int. Ed.*, 2009, **48**, 3799–3801.

(7) J. Peng and D. L. J. Clive, *Org. Lett.*, 2007, **9**, 2939–2941.

(8) D. V. Gribkov, S. J. Pastine, M. Schnürch and D. Sames, *J. Am. Chem. Soc.*, 2007, **129**, 11750–11755.

(9) D. P. Affron and J. A. Bull, *European J. Org. Chem.*, 2016, 139–149.

(10) D. Antermite, D. P. Affron and J. A. Bull, *Org. Lett.*, 2018, **20**, 3948–3952.

(11) V. K. Aggarwal, C. J. Astle, H. Iding, B. Wirz and M. Rogers-Evans, *Tetrahedron Lett.*, 2005, **46**, 945–947.

(12) F. Prause, J. Kaldun, B. Arensmeyer, B. Wennemann, B. Fröhlich, D. Scharnagel and M. Breuning, *Synth.*, 2015, **47**, 575–586.

(13) S. D. Taylor, C. C. Kotoris, A. N. Dinaut and M. J. Chen, *Tetrahedron*, 1998, **54**, 1691–1714.

(14) S. Ganapathy, B. B. V. Soma Sekhar, S. M. Cairns, K. Akutagawa and W. G. Bentrude, *J. Am. Chem. Soc.*, 1999, **121**, 2085–2096.

(15) D. W. G. Wone, J. A. Rowley, A. W. Garofalo and C. E. Berkman, *Bioorganic Med. Chem.*, 2006, **14**, 67–76.

(16) S. Chen, Y. Ruan, J. D. Brown, C. M. Hadad and J. D. Badjić, *J. Am. Chem. Soc.*, 2014, **136**, 17337–17342.

(17) H. Wang, L. Huang, X. Cao, D. Liang and A. Y. Peng, *Org. Biomol. Chem.*, 2017, **15**, 7396–7403.

(18) Z. Zhao and P. S. Mariano, *Tetrahedron*, 2006, **62**, 7266–7273.

(19) a) N. Isono and M. Mori, *J. Org. Chem*, 1995, **60**, 115–119; b) N. Y. Kuznetsov, G. D. Kolomnikova, V. N. Khrustalev, D. G. Golovanov and Y. N. Bubnov, *European J. Org. Chem.*, 2008, 5647–5655.
